# Supplementary material for: Annexin A7 enhances TIA1 axonal trafficking to counteract pathological aggregation in neurons
Source: EMBO J. 2025 Nov 3;44(24):7477–512. doi: 10.1038/s44318-025-00609-8 (PMC12706091; doi:10.1038/s44318-025-00609-8)
Supplement: Supplementary file 1 — Appendix [file 44318_2025_609_MOESM1_ESM.pdf]

Appendix for

Annexin A7 Enhances TIA1 Axonal Trafficking to Counteract Pathological Aggregation in Neurons

Yu Feng*, Tongshu Luan*, Zhenda Zhang, Wei Wang, Yuanyuan Chu, Sijia Wan, Xiaorong Pan, Jie Li, Yifan Liu, Yaqian Xu, Kun Dou, and Tong Wang#

* Equal contribution from these authors.

# Corresponding author Email: wangtong@shanghaitech.edu.cn

Table of Content:

Appendix Figure S1 **page 2**

Appendix Figure S2 **page 3**

Appendix Figure S3 **page 4**

Appendix Figure S4 **page 5**

Appendix for exact *P* values **page 6**

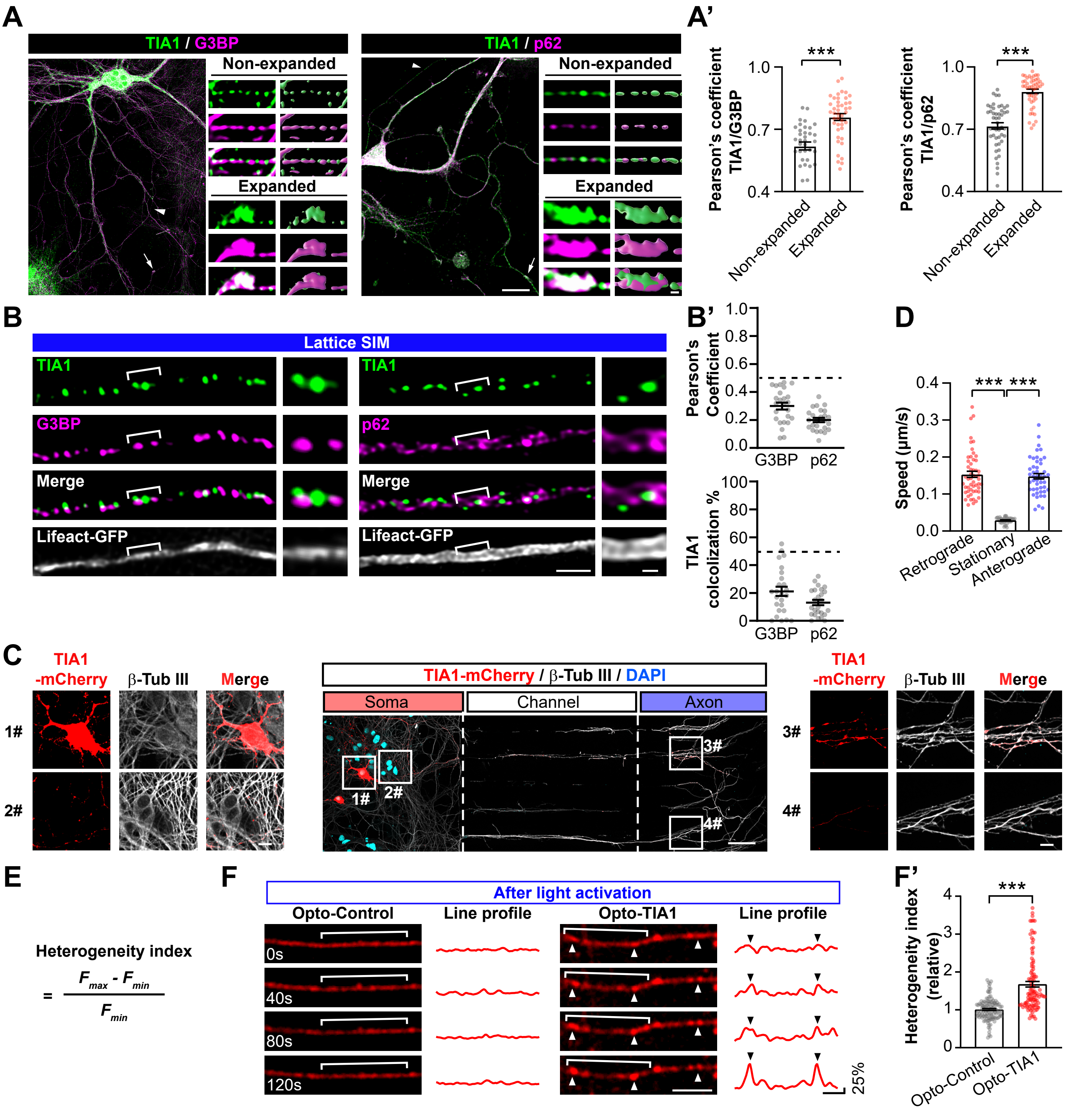

$$\text{Heterogeneity index} = \frac{F_{max} - F_{min}}{F_{min}}$$

Appendix Figure S1: Related to Fig. 1 and Fig. EV1.

(A) Representative images showing the distribution of endogenous TIA1 with G3BP or SQSTM1/p62 in DIV8 cultured rat hippocampal neurons, with Imaris rendering in the right panels. Arrowheads indicate unexpanded axon segments; arrows indicate expanded segments. Scale bars = 20 µm (left), 1 µm (right). **(A')** Pearson's coefficient of TIA1 with G3BP or SQSTM1/p62 in unexpanded and expanded axon segments (For G3BP: *n* = 36 and 43 axons from 3 biological replicates; For p62: *n* = 48 and 47 axons from 3 biological replicates). **(B)** Lattice SIM images showing the distribution of endogenous TIA1 with G3BP or SQSTM1/p62 in axons. Scale bar = 1 µm (left), 0.2 µm (right). **(B')** Quantification of Pearson's coefficient (top) and ratio (bottom) of TIA1 co-localization with G3BP or p62 in axons (*n* = 25 axons from 3 biological replicates). **(C)** Transfection efficiency in neurons cultured in microfluidic devices. Scale bars = 50 µm (middle), 10 µm (sides). **(D)** Quantification of trafficking speeds of TIA1 granules (related to Fig. 1D; *n* =47 axons, from 4 biological replicates). **(E)** Equation of the heterogeneity index. **(F)** Time-lapse images showing intensity changes induced by blue light in axons expressing Opto-Control or Opto-TIA1. Intensity profiles in the bracketed regions are shown on the right. Arrowheads indicate light-induced Opto-TIA1 granules. Scale bars = 10 µm (left), 5 µm (right); y-axis = 25%. **(F')** Quantification of **(F)** showing heterogeneity changes induced by blue light exposure for Opto-Control and Opto-TIA1 (*n* = 118,103 axons from 4 biological replicates). Data represent mean ± SEM; two-tailed unpaired *t*-test in **(A')** ; one-way ANOVA in **(D)** ; one sample *t*-test in **(F')** ; ***P<0.001. See appendix for exact *P* values.

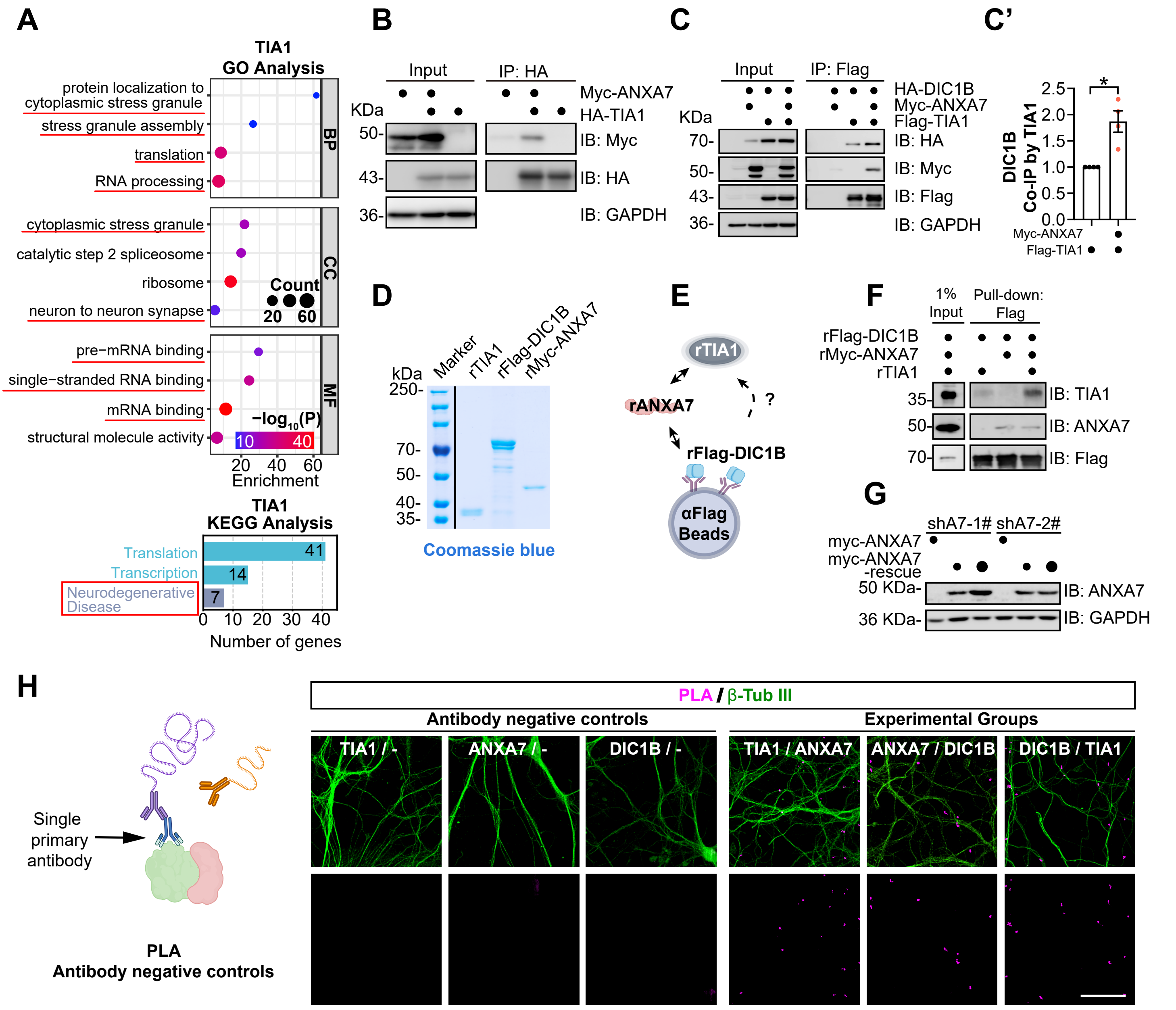

Appendix Figure S2: Related to Fig. 2 and Fig. EV2.

(A) GO and KEGG enrichment pathway analysis for proteins pulled down by GST-TIA1 from P14 rat brain lysates. **(B)** Co-IP of Myc-ANXA7 and HA-TIA1 co-expressed in HEK293T cells, showing interaction. **(C)** Co-IP showing Myc-ANXA7 and HA-DIC1B interact with Flag-TIA1 in HEK293T cells. **(C')** Quantification of **(C)** (n = 4 biological replicates, data represent mean ± SEM, one sample t-test, P = 0.0241). **(D)** Coomassie blue staining of an SDS-PAGE gel showing the purity of recombinant TIA1 (rTIA1), rFlag-DIC1B and rMyc-ANXA7 proteins expressed in *E. Coli*. **(E)** *In vitro* protein pull-down assay schematic, related to Fig. 2G and Appendix Fig. S2F. **(F)** Purified rMyc-ANXA7 protein enhances rTIA1 and rFlag-DIC1B interaction, shown by increased rTIA1 pulled down by rFlag-DIC1B. **(G)** Western blot analysis detecting Myc-ANXA7 levels in HEK293T cells transfected with shANXA7 (shA7) and either Myc-ANXA7 wild type or shRNA-rescue. 2 µg (large dots) or 1 µg (small dots) of Myc-A7-rescue plasmid were used for transfection. **(H)** Left: chematic illustrating antibody negative controls for PLA pairs. Right: PLA signals in negative controls and experimental groups using the indicated antibodies. Scale bar = 50 µm.

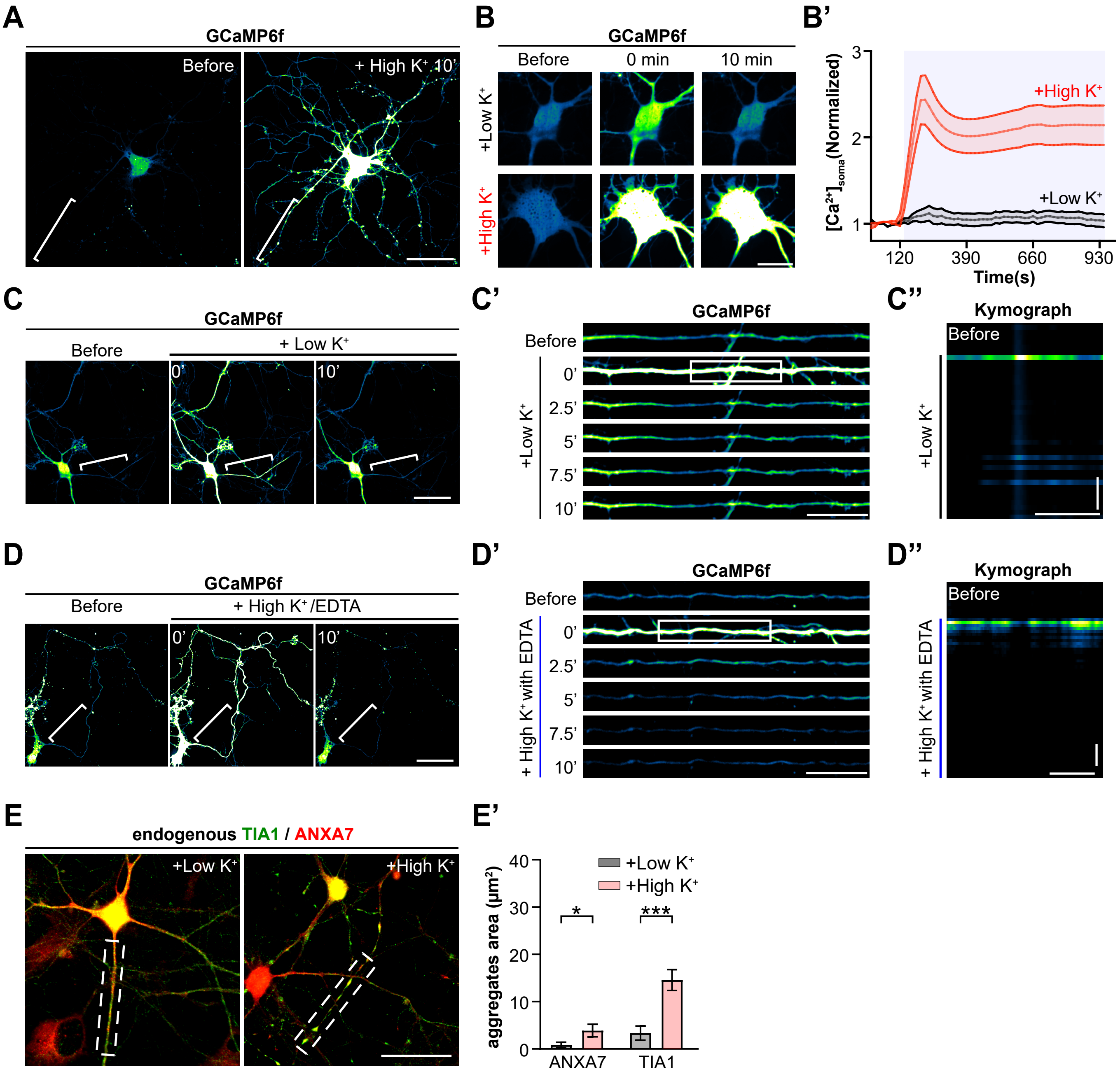

Appendix Figure S3: Related to Fig. 4.

(A) Live-imaging of DIV13 rat hippocampal neurons expressing GCaMP6f, bracketed regaions are shown in (Fig. **4A**), Scale bar = 50 µm. **(B)** Representative live-images displaying somatic Ca^{2+} concentrations ($[Ca^{2+}]_{soma}$) before and after the addition of either 5.6 mM KCl (Low K^+) or 56 mM KCl (High K^+) at the indicated time. Scale bar = 20 µm. **(B')** Quantification of the data in **(B)** ("Low K^+" n = 18 neurons, "High K^+" n = 21 neurons, from 3 biological replicates). **(C–C'')** Time-lapse images of DIV13 cultured rat hippocampal neurons expressing GCaMP6f,showing intracellular Ca^{2+} intensity changes in the axon after addition of 5.6 mM KCl (+Low K^+). Scale bar = 50 µm. Bracketed axonal regions are enlarged in **(C')**. Scale bar =20 µm. **(C'')** Kymographs of the axon segment boxed in **(C')**. Scale bar = 10 µm; y-axis = 100 s. **(D–D'')** Time-lapse images of DIV13 cultured rat hippocampal neurons expressing GCaMP6f, showing intracellular Ca^{2+} intensity changes in axons after addition 56 mM KCl with 0.5 mM EDTA (+High K^+/EDTA). Scale bar = 50 µm. Bracketed axonal regions are enlarged in **(D')**. Scale bar = 20 µm. **(D'')** Kymographs of the axon segment boxed in **(D')**. Scale bar = 10 µm; y-axis =120 s. **(E)** Uncropped confocal images showing endogenous TIA1 and ANXA7 distribution in axons of DIV12 cultured rat hippocampal neurons after 10 min low or high K^+ stimulation. Boxed regions are magnified in Fig. 4G. Scale bar = 50 µm. **(E')** Quantification of **(E)**, showing the total area of TIA1 or ANXA7 granules per 100 µm axon (Low K^+, n =48 axons, High K^+, n = 37 axons, from 3 biological replicates). Data represent mean ± SEM, unpaired t-test, $*P < 0.05$, $***P < 0.001$. See appendix for exact P values.

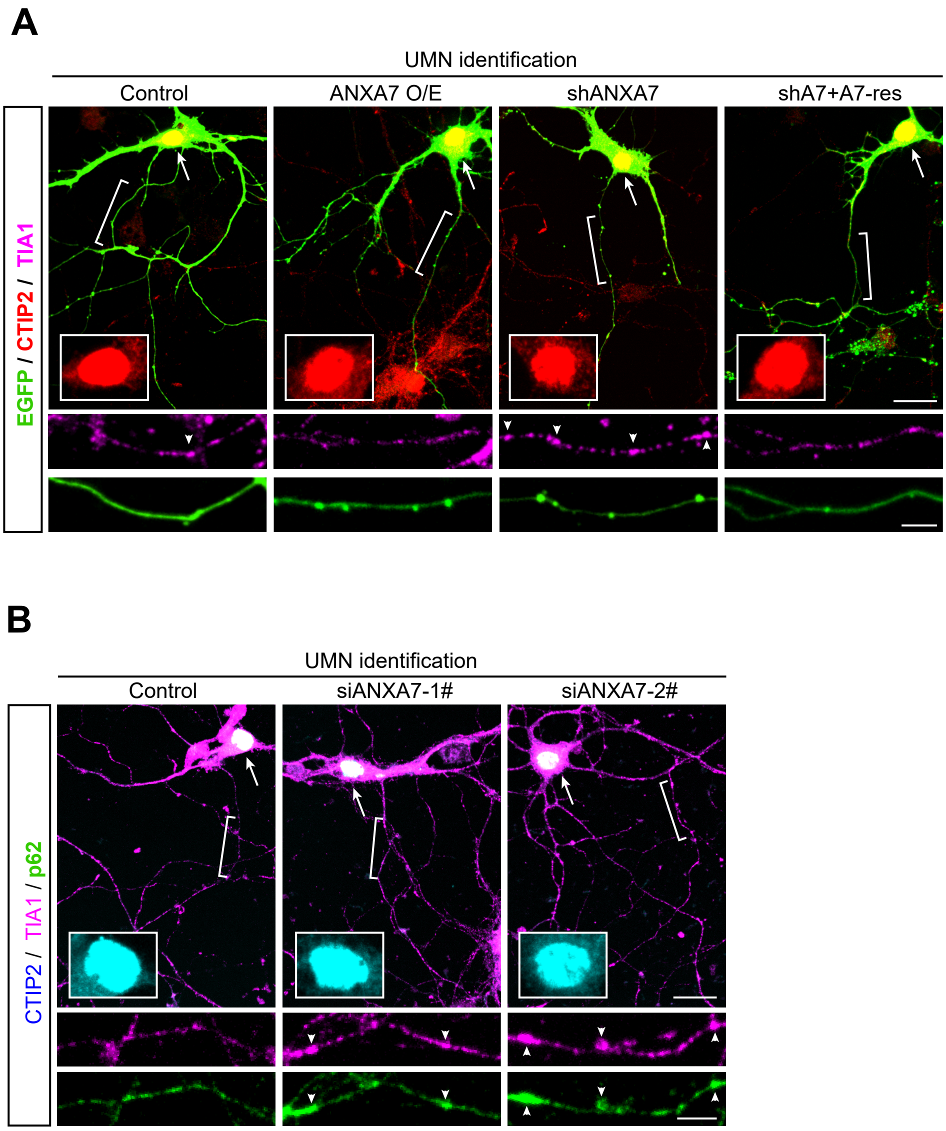

Appendix Figure S4: Related to Fig. 6 and Fig. EV4.

(A) Uncropped images corresponding to Fig. 6A, showing identification of CTIP2-positive UMNs in cultured cortical neurons. Insets show zoomed views of CTIP2 staining (red) in the cell bodies indicated by arrowheads. Bracketed regions are enlarged below. Scale bars= 20 µm (top), 5 µm (bottom).

(B) Uncropped images corresponding to Fig. 6F, showing identification of CTIP2-positive UMNs in cultured cortical neurons. Insets show zoomed views of CTIP2 staining (blue) in the cell bodies indicated by arrowheads. Bracketed regions are enlarged below. Scale bars= 20 µm (top), 5 µm (bottom).

Appendix for exact *P* values.

Figure Number	Adjusted *P*	Significance
**Figure 1B'**		
TIA1^{+} RNPs vs. All RNPs	<0.0001	****
**Figure 1D''**		
Anterograde vs. Retrograde	<0.0001	****
**Figure 1G'**		
Control vs. shDIC1B-1#	0.0044	**
Control vs. shDIC1B-2#	0.0011	**
**Figure 1H''**		
+Hex vs. -Hex	<0.0001	****
**Figure 2G'**		
Myc-ANXA7 vs. TIA1	0.0088	**
TIA1 vs. TIA1 + Myc-ANXA7	0.0463	*
**Figure 2H'**		
Control vs. Myc-ANXA7	0.0267	*
Control vs. shANXA7	0.0474	*
**Figure 2J' and Figure 2J''**		
(1) vs. (2)	0.0266	**
(1) vs. (3)	0.7464	ns
(1) vs. (4)	<0.0001	****
(2) vs. (4)	0.0901	ns
**Figure 2K'**		
Control vs. siANXA7-1#	<0.0001	****
Control vs. siANXA7-2#	<0.0001	****
siA7-2# vs. siA7-2#+A7-res	<0.0001	****
**Figure 3B'**		
0mM vs. 1mM	0.0062	**
0mM vs. 5mM	<0.0001	****
0mM vs. 10mM	<0.0001	****
**Figure 3C'**		
Control vs. +5% PEG	0.0355	*
Control vs. 1mM Ca^{2+}	<0.0001	****
**Figure 3E'**		
density -Ca^{2+} vs. +Ca^{2+}	0.0004	***

total area -Ca^{2+} vs. +Ca^{2+}	0.007	**
**Figure 3H'**		
binding beads -Ca^{2+} vs. +Ca^{2+}	0.0007	***
**Figure 3I'-I''**		
area -Ca^{2+} vs. +Ca^{2+}	0.0181	*
density -Ca^{2+} vs. +Ca^{2+}	0.0023	**
**Figure 4B'**		
Before vs. + High K^+	<0.0001	****
**Figure 4C''**		
Before vs. High K^+	<0.0001	****
Before vs. High K^+ + EDTA	0.9642	ns
**Figure 4D''**		
Before vs. High K^+	<0.0001	****
Before vs. High K^+ + EDTA	0.7917	ns
**Figure 4E''**		
Before vs. + High K^+	0.0106	*
**Figure 4F**		
ANXA7 Low K^+ vs. High K^+	<0.0001	****
TIA1 Low K^+ vs. High K^+	<0.0001	****
**Figure 4G'**		
ANXA7 Low K^+ vs. High K^+	0.0098	**
TIA1 Low K^+ vs. High K^+	<0.0001	****
**Figure 4I'**		
Before vs. Flux	0.0001	***
Flux vs. After	<0.0001	****
**Figure 4J'**		
**ANXA7-mCherry:**		
Before vs. Flux	<0.0001	****
Flux vs. After	<0.0001	****
**TIA1-mCherry:**		
Before vs. Flux	<0.0001	****
Flux vs. After	<0.0001	****
**Figure 5C'**		
Anterograde vs. Retrograde	<0.0001	****
Anterograde vs. Stationary	<0.0001	****

**Figure 5D'**		
LAMP1 vs. EEA1	<0.0001	****
LAMP1 vs. DCP1A	<0.0001	****
LAMP1 vs. LC3	0.0205	*
**Figure 5F''**		
***Ryk*:**		
shANXA7 vs. Control	0.0111	*
***Neurod2*:**		
shANXA7 vs. Control	0.0094	**
***Car7*:**		
shANXA7 vs. Control	0.0271	*
**Figure 5G'**		
**Hippocampal neurons:**		
Control vs. shANXA7	0.024	*
Control vs. shANXA7 + ANXA7-res	0.875	ns
shANXA7 vs. shANXA7 + ANXA7-res	0.0057	**
**Cortical neurons:**		
Control vs. shANXA7	0.0075	**
Control vs. shANXA7 + ANXA7-res	0.7923	ns
shANXA7 vs. shANXA7 + ANXA7-res	0.0436	*
**Figure 6A'**		
**HN:**		
A7 O/E vs. Control	0.0097	**
shA7 vs. Control	0.0347	*
shA7 + A7-res vs. shA7	<0.0001	****
**UMN:**		
A7 O/E vs. Control	0.032	*
shA7 vs. Control	0.005	**
shA7 + A7-res vs. shA7	<0.0001	****
**Figure 6B'**		
Ctrl vs. siANXA7	0.0343	*
Ctrl vs. ANXA7 O/E	0.0412	*
**Figure 6C'**		
Control vs. ANXA7 O/E	0.0004	***
Control vs. shANXA7	<0.0001	****

**Figure 6D'**		
siANXA7 vs. siCtrl	<0.0001	****
**Figure 6E'**		
Ctrl vs. shA7-1#	<0.0001	****
Ctrl vs. shA7-2#	<0.0001	****
**Figure 6F'**		
Ctrl vs. siA7-1#	<0.0001	****
Ctrl vs. siA7-2#	<0.0001	****
**Figure 6G'**		
Ctrl vs. shA7-1#	0.0399	*
Ctrl vs. shA7-2#	0.0128	*
**Figure 6G''**		
Ctrl vs. shA7-1#	0.0269	*
Ctrl vs. shA7-2#	0.0377	*
**Figure 6G'''**		
Ctrl vs. shA7-1#	0.0005	***
Ctrl vs. shA7-2#	0.014	*
**Figure 7B'**		
Control vs. shA7-3#	<0.0001	****
Control vs. shA7-4#	0.003	**
Control vs. shA7-4#-res	0.9079	ns
shA7-4#-res vs. Control	0.8887	ns
shA7-4#-res vs. shA7-3#	0.0011	**
shA7-4#-res vs. shA7-4#	0.0467	*
**Figure 7D'**		
Control vs. shA7-3#	<0.0001	****
Control vs. shA7-4#	<0.0001	****
Control vs. shA7-4#-res	0.007	**
shA7-4#-res vs. Control	0.0067	**
shA7-4#-res vs. shA7-3#	<0.0001	****
shA7-4#-res vs. shA7-4#	<0.0001	****
**Figure 7E'**		
Control vs. shA7-3#	<0.0001	****
Control vs. shA7-4#	<0.0001	****
Control vs. shA7-4#-res	0.2097	ns

shA7-4#-res vs. Control	0.2037	ns
shA7-4#-res vs. shA7-3#	0.0067	**
shA7-4#-res vs. shA7-4#	0.0027	**
**Figure 7F'**		
Control vs. shA7-3#	<0.0001	****
Control vs. shA7-4#	<0.0001	****
Control vs. shA7-4#-res	0.3894	ns
shA7-4#-res vs. Control	0.3399	ns
shA7-4#-res vs. shA7-3#	0.0029	**
shA7-4#-res vs. shA7-4#	0.0021	**
**Figure 7G'**		
Control vs. shA7-3#	<0.0001	****
Control vs. shA7-4#	<0.0001	****
Control vs. shA7-4#-res	0.9204	ns
shA7-4#-res vs. Control	0.9028	ns
shA7-4#-res vs. shA7-3#	<0.0001	****
shA7-4#-res vs. shA7-4#	<0.0001	****
**Figure EV1G'**		
DIC1B vs. LAMP1	<0.0001	****
DIC1B vs. LC3	<0.0001	****
DIC1B vs. Rab5	<0.0001	****
**Figure EV1G''**		
DIC1B vs. LAMP1	0.001	**
DIC1B vs. LC3	<0.0001	****
DIC1B vs. Rab5	<0.0001	****
**Figure EV1G'''**		
DIC1B vs. LAMP1	<0.0001	****
DIC1B vs. LC3	<0.0001	****
DIC1B vs. Rab5	<0.0001	****
**Figure EV1I'**		
+Nocodazole vs. Control	<0.0001	****
**Figure EV2F'**		
GFP-N1 vs. A7-GFP	0.0456	*
GFP-N1 vs. shA7 1#	0.0141	*
GFP-N1 vs. shA7 2#	0.0002	***

**Figure EV2G' and Figure EV2G''**		
(1) vs. (2)	0.0022	**
(1) vs. (3)	0.1245	ns
(1) vs. (4)	<0.0001	****
(4) vs. (1)	<0.0001	****
(4) vs. (2)	0.5087	ns
(4) vs. (3)	0.0097	**
**Figure EV2H'**		
siANXA7 vs. Control	<0.0001	****
**Figure EV2I'**		
siANXA7 vs. Control	<0.0001	****
**Figure EV3A'**		
ANXA7 1 µM vs. 5 µM	0.1732	ns
ANXA7 1 µM vs. 10 µM	<0.0001	****
**Figure EV3A''**		
0% PEG vs. 5% PEG	0.0161	*
0% PEG vs. 10% PEG	<0.0001	****
0% PEG vs. 15% PEG	<0.0001	****
**Figure EV3B'**		
0 mM vs. 100 mM	0.6173	ns
0 mM vs. 300 mM	0.9426	ns
0 mM vs. 500 mM	0.985	ns
**Figure EV3C'**		
0 mM vs. 1 mM	0.9885	ns
0 mM vs. 2 mM	0.6982	ns
0 mM vs. 5 mM	0.961	ns
0 mM vs. 10 mM	0.2348	ns
**Figure EV3F'**		
Control vs. %5 PEG	<0.0001	****
**Figure EV3F''**		
Control vs. %5 PEG	0.3117	ns
**Figure EV3G'**		
0 mM vs. 1 mM	0.9959	ns
0 mM vs. 5 mM	0.9992	ns
0 mM vs. 10 mM	0.2817	ns

**Figure EV4B**		
ANXA7 O/E vs. Control	0.0037	**
shANXA7 vs. Control	0.0401	*
shA7+A7-res vs. shANXA7	0.0399	*
**Figure EV4C'**		
Control vs. shANXA7	<0.0001	****
Control vs. ANXA7 O/E	0.0002	***
**Figure EV4D**		
Control vs. ANXA7 O/E	0.0369	*
Control vs. shANXA7	0.0381	*
**Figure EV4E'**		
Control vs. shANXA7	0.015	*
Control vs. ANXA7 O/E	0.9992	ns
shANXA7 vs. ANXA7 O/E	0.0191	*
**Figure EV4F'**		
shANXA7-1# vs. Ctrl	0.0011	**
shANXA7-2# vs. Ctrl	0.0003	***
**Figure EV4F''**		
Ctrl vs. shANXA7-1#	<0.0001	****
Ctrl vs. shANXA7-2#	<0.0001	****
**Figure EV4G'**		
Ctrl vs. shANXA7-1#	<0.0001	****
Ctrl vs. shANXA7-2#	<0.0001	****
**Figure EV5C'**		
Control vs. shA7-3#	0.0335	*
Control vs. shA7-4#	0.0046	**
**Appendix Figure S1A'-G3BP**		
Non-expanded vs. expanded	<0.0001	****
**Appendix Figure S1A'-p62**		
Non-expanded vs. expanded	<0.0001	****
**Appendix Figure S1D**		
Stationary vs. Retrograde	<0.0001	****
Stationary vs. Anterograde	<0.0001	****
**Appendix Figure S1F'**		
Control vs. TIA1	<0.0001	****

**Appendix Figure S2C'**		
-Myc-A7 vs. +Myc-A7	0.0241	*
**Appendix Figure S3E'**		
ANXA7 Low K^+ vs. High K^+	0.0243	*
TIA1 Low K^+ vs. High K^+	<0.0001	****
