# Supplementary material for: Annexin A7 enhances TIA1 axonal trafficking to counteract pathological aggregation in neurons
Source: EMBO J. 2025 Nov 3;44(24):7477–512. doi: 10.1038/s44318-025-00609-8 (PMC12706091; doi:10.1038/s44318-025-00609-8)
Supplement: Supplementary file 8 — Movie EV1 [file 44318_2025_609_MOESM8_ESM.zip › EMBOJ-2024-119578_Movie EV1/Movie EV1.docx]

**Movie EV1. Co-transport of TIA1 granules and RNA along the axon.**

DIV8 hippocampal neurons were transfected with EGFP-TIA1 (green) and CY5-UTP (magenta). Time-lapse dual-color confocal microscopy imaging shows the co-transport of TIA1 granules (green) and RNA (magenta), composing the TIA1-containing RNPs along the axons. The region of interest (ROI) is magnified in the right panels. Directions relative to the soma are marked below the magnifications. Scale bar: 20 µm (left), 10 µm (right). Related to Fig. 1B.
