# Supplementary material for: Annexin A7 enhances TIA1 axonal trafficking to counteract pathological aggregation in neurons
Source: EMBO J. 2025 Nov 3;44(24):7477–512. doi: 10.1038/s44318-025-00609-8 (PMC12706091; doi:10.1038/s44318-025-00609-8)
Supplement: Supplementary file 9 — Movie EV2 [file 44318_2025_609_MOESM9_ESM.zip › EMBOJ-2024-119578_Movie EV2/Movie EV2.docx]

**Movie EV2. Most TIA1 granules undergo retrograde trafficking in axons.**

DIV8 rat hippocampal neurons cultured in the microfluidic device expressing EGFP-N1 (green) and TIA1-mCherry (magenta) were observed under time-lapse confocal microscopy. Live-imaging shows the directional trafficking of TIA1 granules (magenta) in the axon. The bracketed axonal region is amplified in the bottom panel, with different moving TIA1 granules indicated with arrowheads of different colors. Scale bar: 50 µm (top), 15 µm (bottom). Related to Fig. 1D.
