# Supplementary material for: Annexin A7 enhances TIA1 axonal trafficking to counteract pathological aggregation in neurons
Source: EMBO J. 2025 Nov 3;44(24):7477–512. doi: 10.1038/s44318-025-00609-8 (PMC12706091; doi:10.1038/s44318-025-00609-8)
Supplement: Supplementary file 10 — Movie EV3 [file 44318_2025_609_MOESM10_ESM.zip › EMBOJ-2024-119578_Movie EV3/Movie EV3.docx]

**Movie EV3. TIA1 granules are not co-transported with retrograde membranous organelles in axons.**

DIV8-9 rat hippocampal neurons expressing fluorescently tagged TIA1 (green) were labelled for retrograde organelles: signaling endosomes (CTB), synaptic vesicle (SV)-related vesicles (BoNT/A-Hc), and lysosomes (LysoTracker) in microfluidic devices; mitochondria (MitoTracker) and early endosomes (EGFP-Rab5) in dish cultures. Time-lapse confocal microscopy shows the movement of TIA1 granules (green) with each of the retrograde organelles (magenta) in the axon. Scale bar: 5 µm. Related to Fig. 1E and EV1C-F.
