# Supplementary material for: Annexin A7 enhances TIA1 axonal trafficking to counteract pathological aggregation in neurons
Source: EMBO J. 2025 Nov 3;44(24):7477–512. doi: 10.1038/s44318-025-00609-8 (PMC12706091; doi:10.1038/s44318-025-00609-8)
Supplement: Supplementary file 11 — Movie EV4 [file 44318_2025_609_MOESM11_ESM.zip › EMBOJ-2024-119578_Movie EV4/Movie EV4.docx]

**Movie EV4. TIA1 granules are co-transported with dynein subunit DIC1B.**

In DIV8 rat hippocampal neurons cultured in the microfluidic device expressing EGFP-TIA1 and DIC1B-mRFP, time-lapse confocal microscopy shows the co-transport of TIA1 granules (green) and DIC1B (magenta) in the axon. The green hollow arrowheads indicate TIA1 granules, and white hollow arrowheads indicate TIA1 co-transported with DIC1B. Scale bar: 5 µm. Related to Fig. 1F.
