# Supplementary material for: Annexin A7 enhances TIA1 axonal trafficking to counteract pathological aggregation in neurons
Source: EMBO J. 2025 Nov 3;44(24):7477–512. doi: 10.1038/s44318-025-00609-8 (PMC12706091; doi:10.1038/s44318-025-00609-8)
Supplement: Supplementary file 12 — Movie EV5 [file 44318_2025_609_MOESM12_ESM.zip › EMBOJ-2024-119578_Movie EV5/Movie EV5.docx]

**Movie EV5. Formation of light-induced Opto-TIA1 granules in axons.**

DIV9 rat hippocampal neurons expressing either Opto-Control or Opto-TIA1 were activated with blue light while time-lapse images were acquired. Representative live images show the formation of Opto-TIA1 granules (bottom) compared to Opto-Control (top). Triangles indicate the light-induced Opto-TIA1 granules. Scale bar=10 µm. Related to Appendix Fig. S1F.
