# Supplementary material for: Annexin A7 enhances TIA1 axonal trafficking to counteract pathological aggregation in neurons
Source: EMBO J. 2025 Nov 3;44(24):7477–512. doi: 10.1038/s44318-025-00609-8 (PMC12706091; doi:10.1038/s44318-025-00609-8)
Supplement: Supplementary file 13 — Movie EV6 [file 44318_2025_609_MOESM13_ESM.zip › EMBOJ-2024-119578_Movie EV6/Movie EV6.docx]

**Movie EV6. Light-induced Opto-TIA1 granules undergo rapid retrograde trafficking and fusion in axons.**

DIV9 rat hippocampal neurons expressing Opto-TIA1 were activated with blue light, and time-lapse images were acquired throughout the process. Representative live images, taken approximately 19 minutes after the start of blue light activation, show the retrograde trafficking and fusion of newly-formed Opto-TIA1 granules in the axon. Hollow triangles indicate the movement and fusion of Opto-TIA1 granules. Scale bar = 10 µm. Related to Fig. 1I’.
