# Supplementary material for: Annexin A7 enhances TIA1 axonal trafficking to counteract pathological aggregation in neurons
Source: EMBO J. 2025 Nov 3;44(24):7477–512. doi: 10.1038/s44318-025-00609-8 (PMC12706091; doi:10.1038/s44318-025-00609-8)
Supplement: Supplementary file 14 — Movie EV7 [file 44318_2025_609_MOESM14_ESM.zip › EMBOJ-2024-119578_Movie EV7/Movie EV7.docx]

**Movie EV7. Opto-TIA1 granules co-transport with ANXA7 granules in axon.**

DIV9 rat hippocampal neurons co-expressing Opto-TIA1 and ANXA7-EGFP were activated by blue light while time-lapse images were acquired. Representative time-lapse images show the highly correlated retrograde trafficking of Opto-TIA1 (red) and ANXA7 granules (green) in the axon. Arrows indicate the co-trafficking and fusing processes of Opto-TIA1 and ANXA7-EGFP granules. Scale bar = 2 µm. Related to Fig. 2F.
