# Supplementary material for: Annexin A7 enhances TIA1 axonal trafficking to counteract pathological aggregation in neurons
Source: EMBO J. 2025 Nov 3;44(24):7477–512. doi: 10.1038/s44318-025-00609-8 (PMC12706091; doi:10.1038/s44318-025-00609-8)
Supplement: Supplementary file 15 — Movie EV8 [file 44318_2025_609_MOESM15_ESM.zip › EMBOJ-2024-119578_Movie EV8/Movie EV8.docx]

**Movie EV8. Time-lapse SIM images showing the co-transported TIA1 and ANXA7 granules in the axon.**

Axon trafficking of DIV9 rat hippocampal neurons co-expressing EGFP-TIA1 and ANXA7-mCherry visualized by time-lapse dual-color SIM. The co-trafficking of ANXA7-mCherry (red) and EGFP-TIA1 (green) granules in the retrograde direction are indicated with white arrowheads. Scale bar = 5 µm. Related to Fig. EV2D.
