# Supplementary material for: Annexin A7 enhances TIA1 axonal trafficking to counteract pathological aggregation in neurons
Source: EMBO J. 2025 Nov 3;44(24):7477–512. doi: 10.1038/s44318-025-00609-8 (PMC12706091; doi:10.1038/s44318-025-00609-8)
Supplement: Supplementary file 16 — Movie EV9 [file 44318_2025_609_MOESM16_ESM.zip › EMBOJ-2024-119578_Movie EV9/Movie EV9.docx]

**Movie EV9. High K^+^ depolarization induces Ca^2+^ elevation in neurons.**

In DIV13 neurons expressing GCaMP6f, time-lapse confocal images were acquired to capture the changes in intracellular Ca^2+^ levels induced by the addition of high K^+^ buffer. The yellow background indicates the duration of high K^+^ stimulus, and the bracketed ROI is magnified in the lower panel. Scale bar = 50 µm (top) and 20 µm (bottom). Related to Fig.4A and Appendix Fig. S3A.
