# Supplementary material for: Annexin A7 enhances TIA1 axonal trafficking to counteract pathological aggregation in neurons
Source: EMBO J. 2025 Nov 3;44(24):7477–512. doi: 10.1038/s44318-025-00609-8 (PMC12706091; doi:10.1038/s44318-025-00609-8)
Supplement: Supplementary file 17 — Movie EV10 [file 44318_2025_609_MOESM17_ESM.zip › EMBOJ-2024-119578_Movie EV10/Movie EV10.docx]

**Movie EV10. Ca^2+^ elevation hot spots colocalize with ANXA7 aggregates in axon.**

In axons of DIV13 neurons expressing GCaMP6f and ANXA7-mCherry, dual-color time-lapse images were acquired to show the formation of Ca^2+^ hot spots and the aggregation of ANXA7-mCherry in response to the addition of high K^+^ buffer. The yellow background indicates the duration of high K^+^ stimulus, and the white arrows represent hot spots. Scale bar = 10 µm. Related to Fig. 4E.
