# Supplementary material for: Annexin A7 enhances TIA1 axonal trafficking to counteract pathological aggregation in neurons
Source: EMBO J. 2025 Nov 3;44(24):7477–512. doi: 10.1038/s44318-025-00609-8 (PMC12706091; doi:10.1038/s44318-025-00609-8)
Supplement: Supplementary file 18 — Movie EV11 [file 44318_2025_609_MOESM18_ESM.zip › EMBOJ-2024-119578_Movie EV11/Movie EV11.docx]

**Movie EV11. Flux-induced transient Ca^2+^ elevation in axons of neurons cultured in AoC.**

In axons of DIV8 neurons expressing GCaMP6f and ANXA7-mCherry or TIA1-mCherry, dual-color time-lapse images were acquired to show the formation of Ca^2+^ hot spots and the aggregation of ANXA7-mCherry or TIA1-mCherry in response to mechanical stress. The orange background indicates the duration of fluid flow. Scale bar = 10 µm. Related to Fig. 4J.
