# Supplementary material for: Annexin A7 enhances TIA1 axonal trafficking to counteract pathological aggregation in neurons
Source: EMBO J. 2025 Nov 3;44(24):7477–512. doi: 10.1038/s44318-025-00609-8 (PMC12706091; doi:10.1038/s44318-025-00609-8)
Supplement: Supplementary file 19 — Movie EV12 [file 44318_2025_609_MOESM19_ESM.zip › EMBOJ-2024-119578_Movie EV12/Movie EV12.docx]

**Movie EV12. RNA-select labelled RNPs retrograde axon trafficking in neurons cultured in microfluidic device.**

DIV8 rat hippocampal neurons cultured in the microfluidic device and labeled with RNA-select in axon chamber. Time-lapse confocal imaging captured the directional movement of RNA granules (green) within axons, with individual moving granules indicated by arrowheads of different colors. Scale bar: 15 µm. Related to Fig. 5C.
