# Supplementary material for: Annexin A7 enhances TIA1 axonal trafficking to counteract pathological aggregation in neurons
Source: EMBO J. 2025 Nov 3;44(24):7477–512. doi: 10.1038/s44318-025-00609-8 (PMC12706091; doi:10.1038/s44318-025-00609-8)
Supplement: Supplementary file 20 — Movie EV13 [file 44318_2025_609_MOESM20_ESM.zip › EMBOJ-2024-119578_Movie EV13/Movie EV13.docx]

**Movie EV13. ANXA7 expression affects the dynamics of TIA1 granules in axons.**

In DIV8 rat hippocampal neurons, EGFP-TIA1 was co-transfected with either Myc-ANXA7 (ANXA7 OE) or siANXA7 (ANXA7 KD), and then an axon-segment FRAP assay was conducted to show the dynamics of EGFP-TIA1 granules over a long segment of axon shafts. The representative FRAP movie depicts the intensity recovery of EGFP-TIA1, demonstrating the molecular dynamics of TIA1 in axons. Scale bar = 10 μm. Related to Fig. 6B.
