# Supplementary material for: Annexin A7 enhances TIA1 axonal trafficking to counteract pathological aggregation in neurons
Source: EMBO J. 2025 Nov 3;44(24):7477–512. doi: 10.1038/s44318-025-00609-8 (PMC12706091; doi:10.1038/s44318-025-00609-8)
Supplement: Supplementary file 21 — Movie EV14 [file 44318_2025_609_MOESM21_ESM.zip › EMBOJ-2024-119578_Movie EV14/Movie EV14.docx]

**Movie EV14. ANXA7 expression level affects the trafficking efficiency of TIA1 granules in axons.**

DIV8 rat hippocampal neurons expressing TIA1-mCherry were co-transfected with either ANXA7-EGFP (ANXA7 OE) or shANXA7 (ANXA7 KD). Representative time-lapse confocal images depict the axon trafficking of TIA1 granules in the indicated groups. Different TIA1 granules are indicated with different coloured hollow arrows. Scale bar = 5 µm. Related to Fig. 6C.
