# Supplementary material for: Annexin A7 enhances TIA1 axonal trafficking to counteract pathological aggregation in neurons
Source: EMBO J. 2025 Nov 3;44(24):7477–512. doi: 10.1038/s44318-025-00609-8 (PMC12706091; doi:10.1038/s44318-025-00609-8)
Supplement: Supplementary file 22 — Movie EV15 [file 44318_2025_609_MOESM22_ESM.zip › EMBOJ-2024-119578_Movie EV15/Movie EV15.docx]

**Movie EV15. Down-regulation of ANXA7 leads to the formation of TIA1 aggregates in axons.**

In DIV8 rat hippocampal neurons, EGFP-TIA1 was co-transfected with either control siRNA (siControl) or siANXA7, and then a FRAP assay was conducted on large EGFP-TIA1 granules to examine the dynamics of EGFP-TIA1 molecules focally. The representative FRAP movie depicts the intensity recovery of EGFP-TIA1, demonstrating the molecular dynamics of TIA1 molecules within these large condensates. Scale bar = 2 μm. Related to Fig. 6D.
