# Supplementary figures and images for: Annexin A7 enhances TIA1 axonal trafficking to counteract pathological aggregation in neurons (part 1 of 5)
Source: EMBO J. 2025 Nov 3;44(24):7477–512. doi: 10.1038/s44318-025-00609-8 (PMC12706091; doi:10.1038/s44318-025-00609-8)

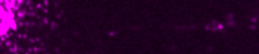

Supplement: Supplementary file 23 — Source datad Fig. 1 [file 44318_2025_609_MOESM23_ESM.zip › EMBOJ-2024-119578_SourceDataForFigure1/1B/CY5-UTP.tif]

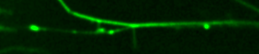

Supplement: Supplementary file 23 — Source datad Fig. 1 [file 44318_2025_609_MOESM23_ESM.zip › EMBOJ-2024-119578_SourceDataForFigure1/1B/GFP-TIA1.tif]

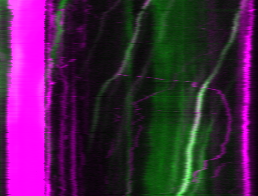

Supplement: Supplementary file 23 — Source datad Fig. 1 [file 44318_2025_609_MOESM23_ESM.zip › EMBOJ-2024-119578_SourceDataForFigure1/1B/Kymograph-Composite.tif]

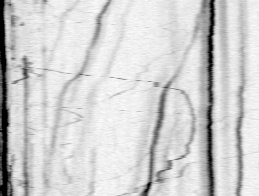

Supplement: Supplementary file 23 — Source datad Fig. 1 [file 44318_2025_609_MOESM23_ESM.zip › EMBOJ-2024-119578_SourceDataForFigure1/1B/Kymograph-CY5-UTP.tif]

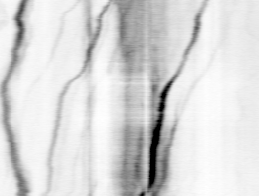

Supplement: Supplementary file 23 — Source datad Fig. 1 [file 44318_2025_609_MOESM23_ESM.zip › EMBOJ-2024-119578_SourceDataForFigure1/1B/Kymograph-GFP-TIA1.tif]

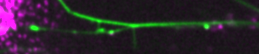

Supplement: Supplementary file 23 — Source datad Fig. 1 [file 44318_2025_609_MOESM23_ESM.zip › EMBOJ-2024-119578_SourceDataForFigure1/1B/Overlay.tif]

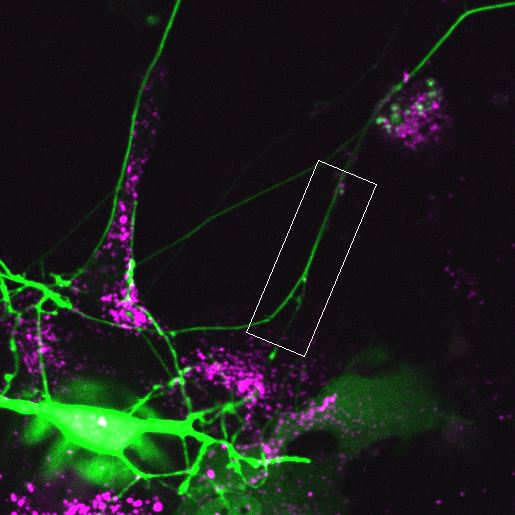

Supplement: Supplementary file 23 — Source datad Fig. 1 [file 44318_2025_609_MOESM23_ESM.zip › EMBOJ-2024-119578_SourceDataForFigure1/1B/TIA1 and CY5-UTP.tif]

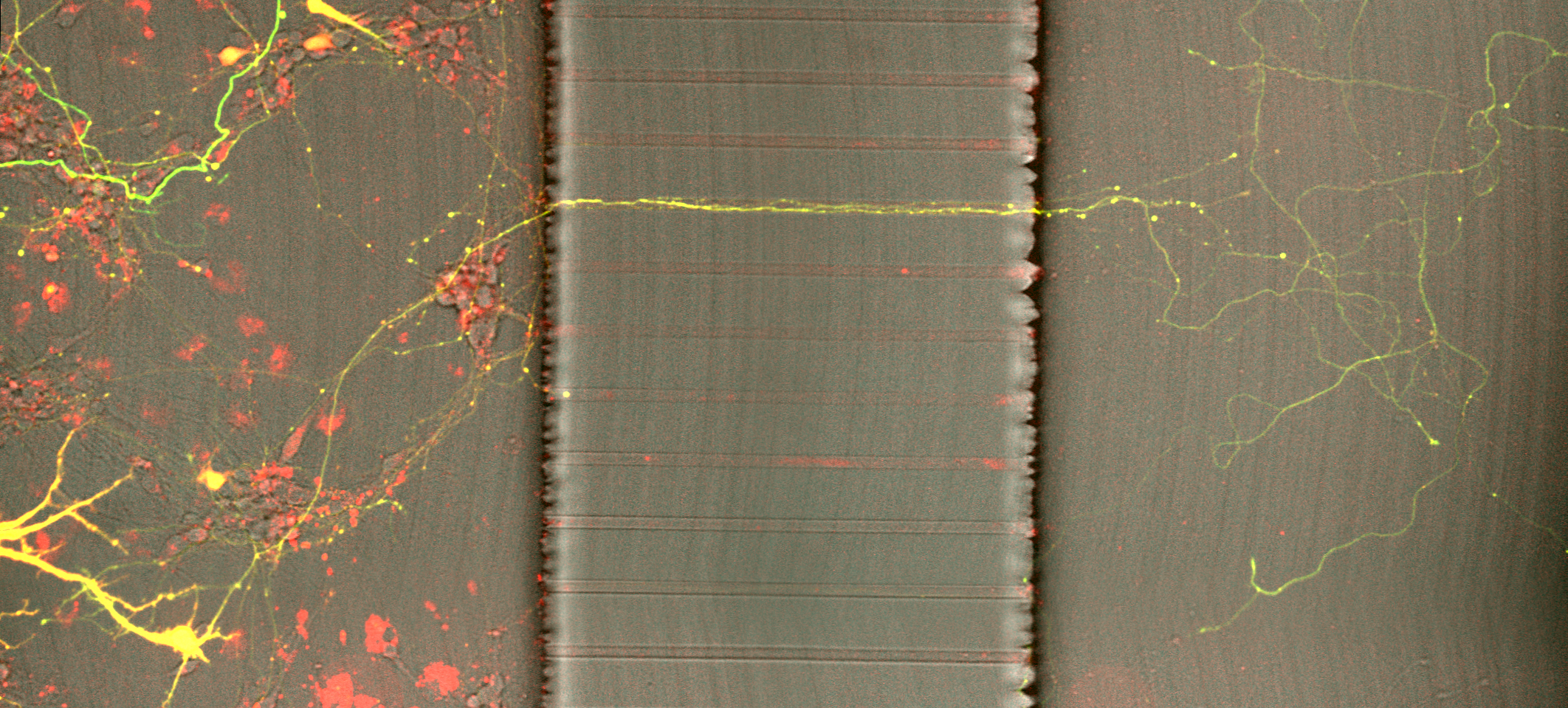

Supplement: Supplementary file 23 — Source datad Fig. 1 [file 44318_2025_609_MOESM23_ESM.zip › EMBOJ-2024-119578_SourceDataForFigure1/1C/TIA1-mCherry and EGFP-N1 in device.tif]

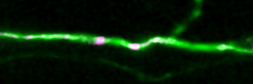

Supplement: Supplementary file 23 — Source datad Fig. 1 [file 44318_2025_609_MOESM23_ESM.zip › EMBOJ-2024-119578_SourceDataForFigure1/1D/Overlay.tif]

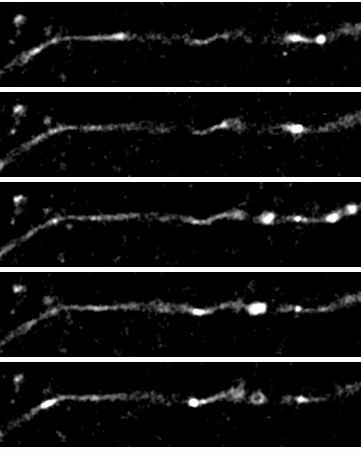

Supplement: Supplementary file 23 — Source datad Fig. 1 [file 44318_2025_609_MOESM23_ESM.zip › EMBOJ-2024-119578_SourceDataForFigure1/1D/TIA-mCherry Montage.tif]

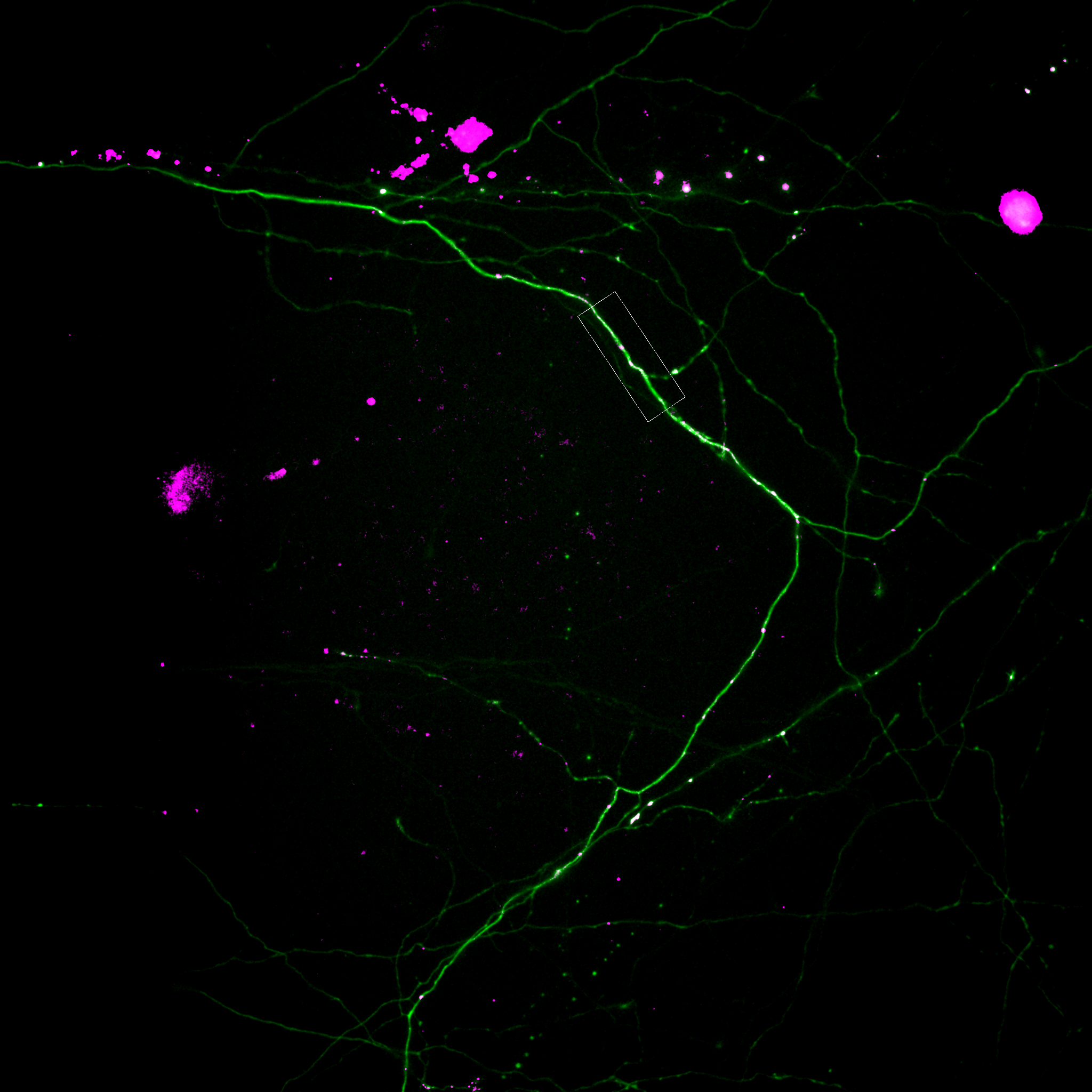

Supplement: Supplementary file 23 — Source datad Fig. 1 [file 44318_2025_609_MOESM23_ESM.zip › EMBOJ-2024-119578_SourceDataForFigure1/1D/TIA1-mCherry and EGFP.tif]

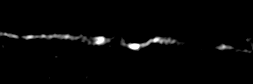

Supplement: Supplementary file 23 — Source datad Fig. 1 [file 44318_2025_609_MOESM23_ESM.zip › EMBOJ-2024-119578_SourceDataForFigure1/1D/TIA1-mCherry.tif]

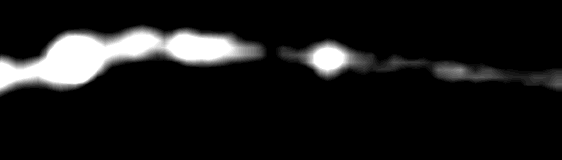

Supplement: Supplementary file 23 — Source datad Fig. 1 [file 44318_2025_609_MOESM23_ESM.zip › EMBOJ-2024-119578_SourceDataForFigure1/1E/0s_ROI_GFP-TIA1.tif]

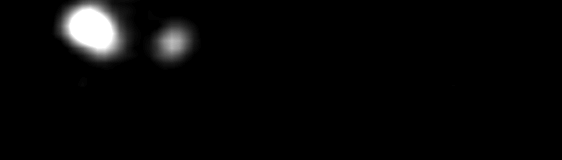

Supplement: Supplementary file 23 — Source datad Fig. 1 [file 44318_2025_609_MOESM23_ESM.zip › EMBOJ-2024-119578_SourceDataForFigure1/1E/0s_ROI_LysoTracker.tif]

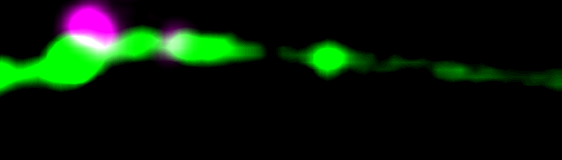

Supplement: Supplementary file 23 — Source datad Fig. 1 [file 44318_2025_609_MOESM23_ESM.zip › EMBOJ-2024-119578_SourceDataForFigure1/1E/0s_ROI_Merge.tif]

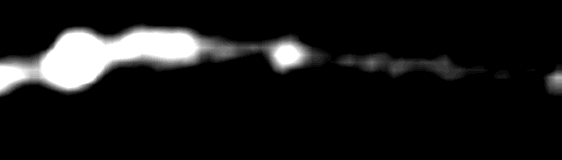

Supplement: Supplementary file 23 — Source datad Fig. 1 [file 44318_2025_609_MOESM23_ESM.zip › EMBOJ-2024-119578_SourceDataForFigure1/1E/20s_ROI_GFP-TIA1.tif]

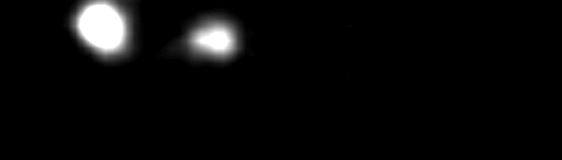

Supplement: Supplementary file 23 — Source datad Fig. 1 [file 44318_2025_609_MOESM23_ESM.zip › EMBOJ-2024-119578_SourceDataForFigure1/1E/20s_ROI_LysoTracker.tif]

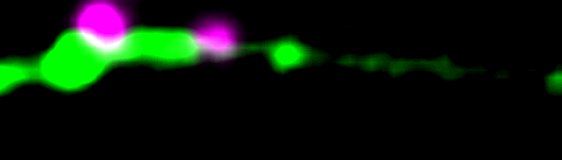

Supplement: Supplementary file 23 — Source datad Fig. 1 [file 44318_2025_609_MOESM23_ESM.zip › EMBOJ-2024-119578_SourceDataForFigure1/1E/20s_ROI_Merge.tif]

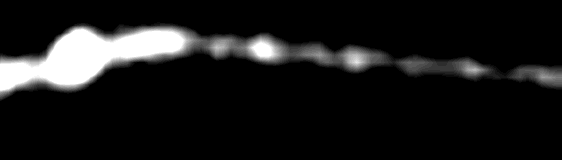

Supplement: Supplementary file 23 — Source datad Fig. 1 [file 44318_2025_609_MOESM23_ESM.zip › EMBOJ-2024-119578_SourceDataForFigure1/1E/40s_ROI_GFP-TIA1.tif]

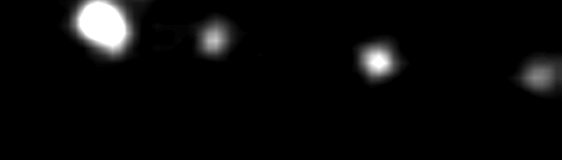

Supplement: Supplementary file 23 — Source datad Fig. 1 [file 44318_2025_609_MOESM23_ESM.zip › EMBOJ-2024-119578_SourceDataForFigure1/1E/40s_ROI_LysoTracker.tif]

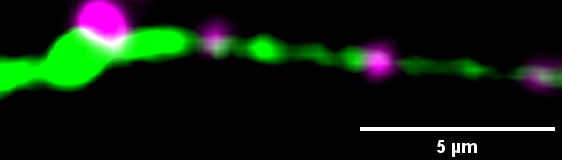

Supplement: Supplementary file 23 — Source datad Fig. 1 [file 44318_2025_609_MOESM23_ESM.zip › EMBOJ-2024-119578_SourceDataForFigure1/1E/40s_ROI_Merge.tif]

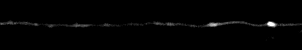

Supplement: Supplementary file 23 — Source datad Fig. 1 [file 44318_2025_609_MOESM23_ESM.zip › EMBOJ-2024-119578_SourceDataForFigure1/1F/DIC1B-mRFP 0s.tif]

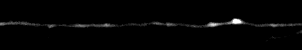

Supplement: Supplementary file 23 — Source datad Fig. 1 [file 44318_2025_609_MOESM23_ESM.zip › EMBOJ-2024-119578_SourceDataForFigure1/1F/DIC1B-mRFP 32s.tif]

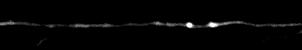

Supplement: Supplementary file 23 — Source datad Fig. 1 [file 44318_2025_609_MOESM23_ESM.zip › EMBOJ-2024-119578_SourceDataForFigure1/1F/DIC1B-mRFP 64s.tif]

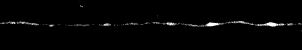

Supplement: Supplementary file 23 — Source datad Fig. 1 [file 44318_2025_609_MOESM23_ESM.zip › EMBOJ-2024-119578_SourceDataForFigure1/1F/EGFP-TIA1 0s.tif]

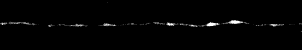

Supplement: Supplementary file 23 — Source datad Fig. 1 [file 44318_2025_609_MOESM23_ESM.zip › EMBOJ-2024-119578_SourceDataForFigure1/1F/EGFP-TIA1 32s.tif]

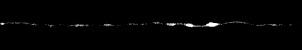

Supplement: Supplementary file 23 — Source datad Fig. 1 [file 44318_2025_609_MOESM23_ESM.zip › EMBOJ-2024-119578_SourceDataForFigure1/1F/EGFP-TIA1 64s.tif]

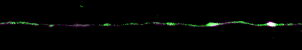

Supplement: Supplementary file 23 — Source datad Fig. 1 [file 44318_2025_609_MOESM23_ESM.zip › EMBOJ-2024-119578_SourceDataForFigure1/1F/Merge 0s.tif]

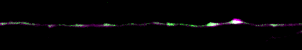

Supplement: Supplementary file 23 — Source datad Fig. 1 [file 44318_2025_609_MOESM23_ESM.zip › EMBOJ-2024-119578_SourceDataForFigure1/1F/Merge 32s.tif]

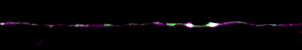

Supplement: Supplementary file 23 — Source datad Fig. 1 [file 44318_2025_609_MOESM23_ESM.zip › EMBOJ-2024-119578_SourceDataForFigure1/1F/Merge 64s.tif]

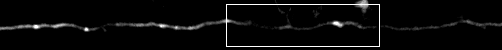

Supplement: Supplementary file 23 — Source datad Fig. 1 [file 44318_2025_609_MOESM23_ESM.zip › EMBOJ-2024-119578_SourceDataForFigure1/1G/Control 0s.tif]

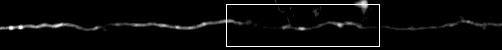

Supplement: Supplementary file 23 — Source datad Fig. 1 [file 44318_2025_609_MOESM23_ESM.zip › EMBOJ-2024-119578_SourceDataForFigure1/1G/Control 30s.tif]

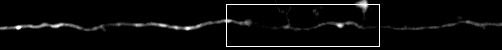

Supplement: Supplementary file 23 — Source datad Fig. 1 [file 44318_2025_609_MOESM23_ESM.zip › EMBOJ-2024-119578_SourceDataForFigure1/1G/Control 60s.tif]

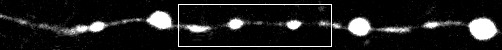

Supplement: Supplementary file 23 — Source datad Fig. 1 [file 44318_2025_609_MOESM23_ESM.zip › EMBOJ-2024-119578_SourceDataForFigure1/1G/shDIC1B-1# 0s.tif]

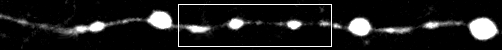

Supplement: Supplementary file 23 — Source datad Fig. 1 [file 44318_2025_609_MOESM23_ESM.zip › EMBOJ-2024-119578_SourceDataForFigure1/1G/shDIC1B-1# 30s.tif]

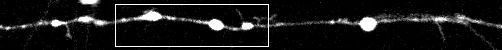

Supplement: Supplementary file 23 — Source datad Fig. 1 [file 44318_2025_609_MOESM23_ESM.zip › EMBOJ-2024-119578_SourceDataForFigure1/1G/shDIC1B-2# 0s.tif]

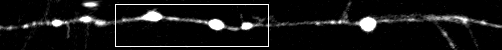

Supplement: Supplementary file 23 — Source datad Fig. 1 [file 44318_2025_609_MOESM23_ESM.zip › EMBOJ-2024-119578_SourceDataForFigure1/1G/shDIC1B-2# 60s.tif]

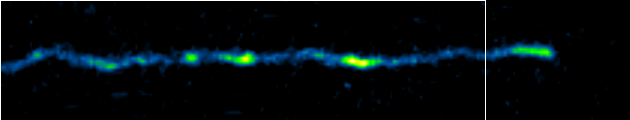

Supplement: Supplementary file 23 — Source datad Fig. 1 [file 44318_2025_609_MOESM23_ESM.zip › EMBOJ-2024-119578_SourceDataForFigure1/1H/+5% 1,6-Hex 0s.tif]

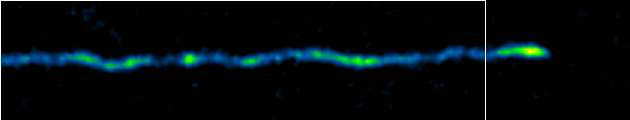

Supplement: Supplementary file 23 — Source datad Fig. 1 [file 44318_2025_609_MOESM23_ESM.zip › EMBOJ-2024-119578_SourceDataForFigure1/1H/+5% 1,6-Hex 40s.tif]

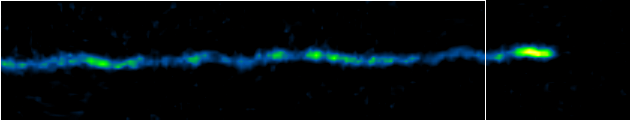

Supplement: Supplementary file 23 — Source datad Fig. 1 [file 44318_2025_609_MOESM23_ESM.zip › EMBOJ-2024-119578_SourceDataForFigure1/1H/+5% 1,6-Hex 80s.tif]

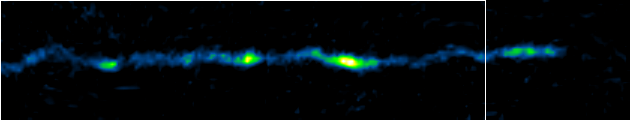

Supplement: Supplementary file 23 — Source datad Fig. 1 [file 44318_2025_609_MOESM23_ESM.zip › EMBOJ-2024-119578_SourceDataForFigure1/1H/before.tif]

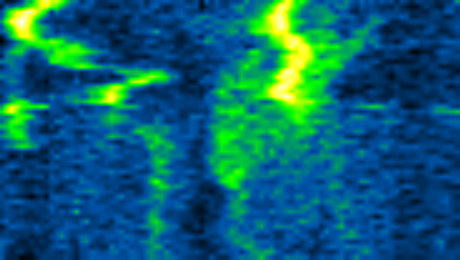

Supplement: Supplementary file 23 — Source datad Fig. 1 [file 44318_2025_609_MOESM23_ESM.zip › EMBOJ-2024-119578_SourceDataForFigure1/1H/Kymograph.tif]

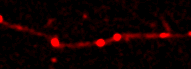

Supplement: Supplementary file 23 — Source datad Fig. 1 [file 44318_2025_609_MOESM23_ESM.zip › EMBOJ-2024-119578_SourceDataForFigure1/1I'/After 0s_raw-ROI.tif]

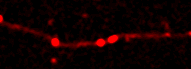

Supplement: Supplementary file 23 — Source datad Fig. 1 [file 44318_2025_609_MOESM23_ESM.zip › EMBOJ-2024-119578_SourceDataForFigure1/1I'/After 19s_raw-ROI.tif]

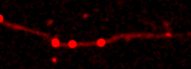

Supplement: Supplementary file 23 — Source datad Fig. 1 [file 44318_2025_609_MOESM23_ESM.zip › EMBOJ-2024-119578_SourceDataForFigure1/1I'/After 37s_raw-ROI.tif]

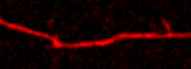

Supplement: Supplementary file 23 — Source datad Fig. 1 [file 44318_2025_609_MOESM23_ESM.zip › EMBOJ-2024-119578_SourceDataForFigure1/1I'/Before_raw-ROI.tif]

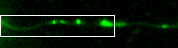

Supplement: Supplementary file 24 — Source data Fig. 2 [file 44318_2025_609_MOESM24_ESM.zip › EMBOJ-2024-119578_SourceDataForFigure2/2F/0' ANXA7-EGFP.tif]

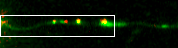

Supplement: Supplementary file 24 — Source data Fig. 2 [file 44318_2025_609_MOESM24_ESM.zip › EMBOJ-2024-119578_SourceDataForFigure2/2F/0' Merge.tif]

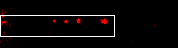

Supplement: Supplementary file 24 — Source data Fig. 2 [file 44318_2025_609_MOESM24_ESM.zip › EMBOJ-2024-119578_SourceDataForFigure2/2F/0' Opto-TIA1.tif]

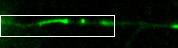

Supplement: Supplementary file 24 — Source data Fig. 2 [file 44318_2025_609_MOESM24_ESM.zip › EMBOJ-2024-119578_SourceDataForFigure2/2F/10' ANXA7-EGFP.tif]

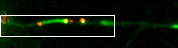

Supplement: Supplementary file 24 — Source data Fig. 2 [file 44318_2025_609_MOESM24_ESM.zip › EMBOJ-2024-119578_SourceDataForFigure2/2F/10' Merge.tif]

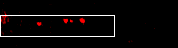

Supplement: Supplementary file 24 — Source data Fig. 2 [file 44318_2025_609_MOESM24_ESM.zip › EMBOJ-2024-119578_SourceDataForFigure2/2F/10' Opto-TIA1.tif]

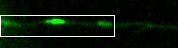

Supplement: Supplementary file 24 — Source data Fig. 2 [file 44318_2025_609_MOESM24_ESM.zip › EMBOJ-2024-119578_SourceDataForFigure2/2F/20' ANXA7-EGFP.tif]

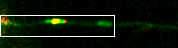

Supplement: Supplementary file 24 — Source data Fig. 2 [file 44318_2025_609_MOESM24_ESM.zip › EMBOJ-2024-119578_SourceDataForFigure2/2F/20' Merge.tif]

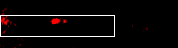

Supplement: Supplementary file 24 — Source data Fig. 2 [file 44318_2025_609_MOESM24_ESM.zip › EMBOJ-2024-119578_SourceDataForFigure2/2F/20' Opto-TIA1.tif]

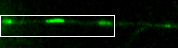

Supplement: Supplementary file 24 — Source data Fig. 2 [file 44318_2025_609_MOESM24_ESM.zip › EMBOJ-2024-119578_SourceDataForFigure2/2F/30' ANXA7-EGFP.tif]

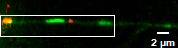

Supplement: Supplementary file 24 — Source data Fig. 2 [file 44318_2025_609_MOESM24_ESM.zip › EMBOJ-2024-119578_SourceDataForFigure2/2F/30' Merge.tif]

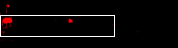

Supplement: Supplementary file 24 — Source data Fig. 2 [file 44318_2025_609_MOESM24_ESM.zip › EMBOJ-2024-119578_SourceDataForFigure2/2F/30' Opto-TIA1.tif]

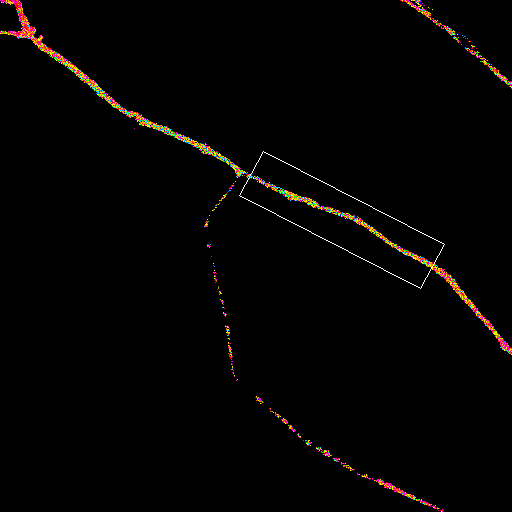

Supplement: Supplementary file 24 — Source data Fig. 2 [file 44318_2025_609_MOESM24_ESM.zip › EMBOJ-2024-119578_SourceDataForFigure2/2J/GFP-TIA1+DIC1B-mRFP+siA7+A7-res.tif]

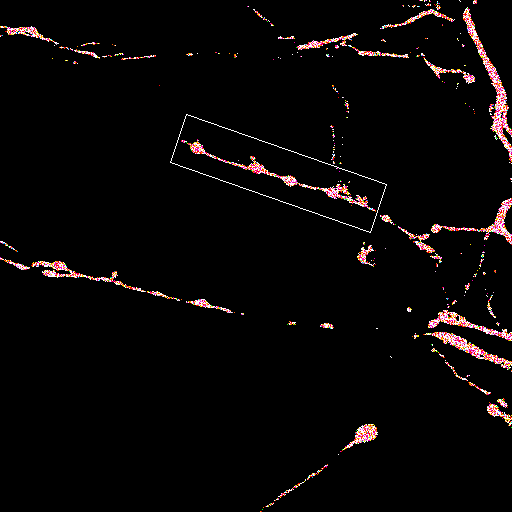

Supplement: Supplementary file 24 — Source data Fig. 2 [file 44318_2025_609_MOESM24_ESM.zip › EMBOJ-2024-119578_SourceDataForFigure2/2J/GFP-TIA1+DIC1B-mRFP+siA7.tif]

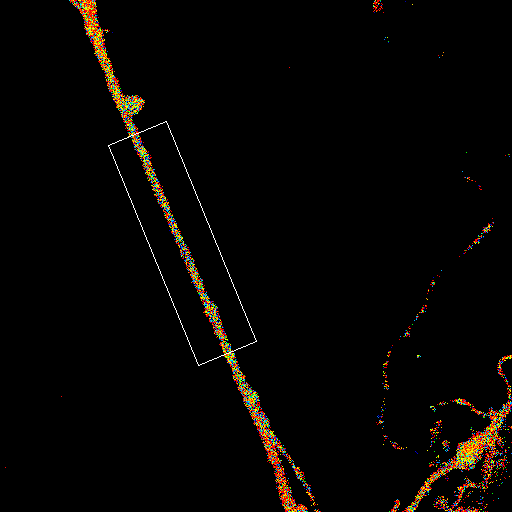

Supplement: Supplementary file 24 — Source data Fig. 2 [file 44318_2025_609_MOESM24_ESM.zip › EMBOJ-2024-119578_SourceDataForFigure2/2J/GFP-TIA1+DIC1B-mRFP.tif]

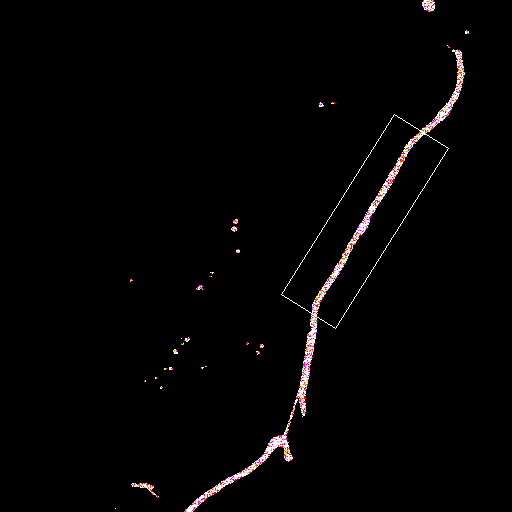

Supplement: Supplementary file 24 — Source data Fig. 2 [file 44318_2025_609_MOESM24_ESM.zip › EMBOJ-2024-119578_SourceDataForFigure2/2J/GFP-TIA1.tif]

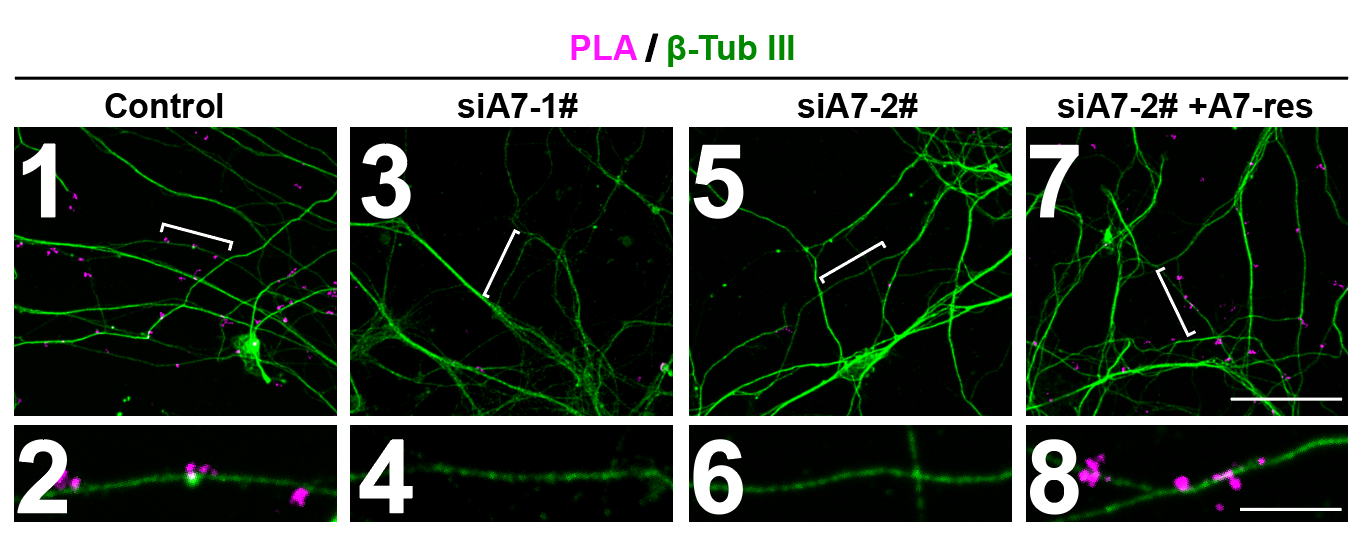

Supplement: Supplementary file 24 — Source data Fig. 2 [file 44318_2025_609_MOESM24_ESM.zip › EMBOJ-2024-119578_SourceDataForFigure2/2K/0-Fig. 2K.tif]

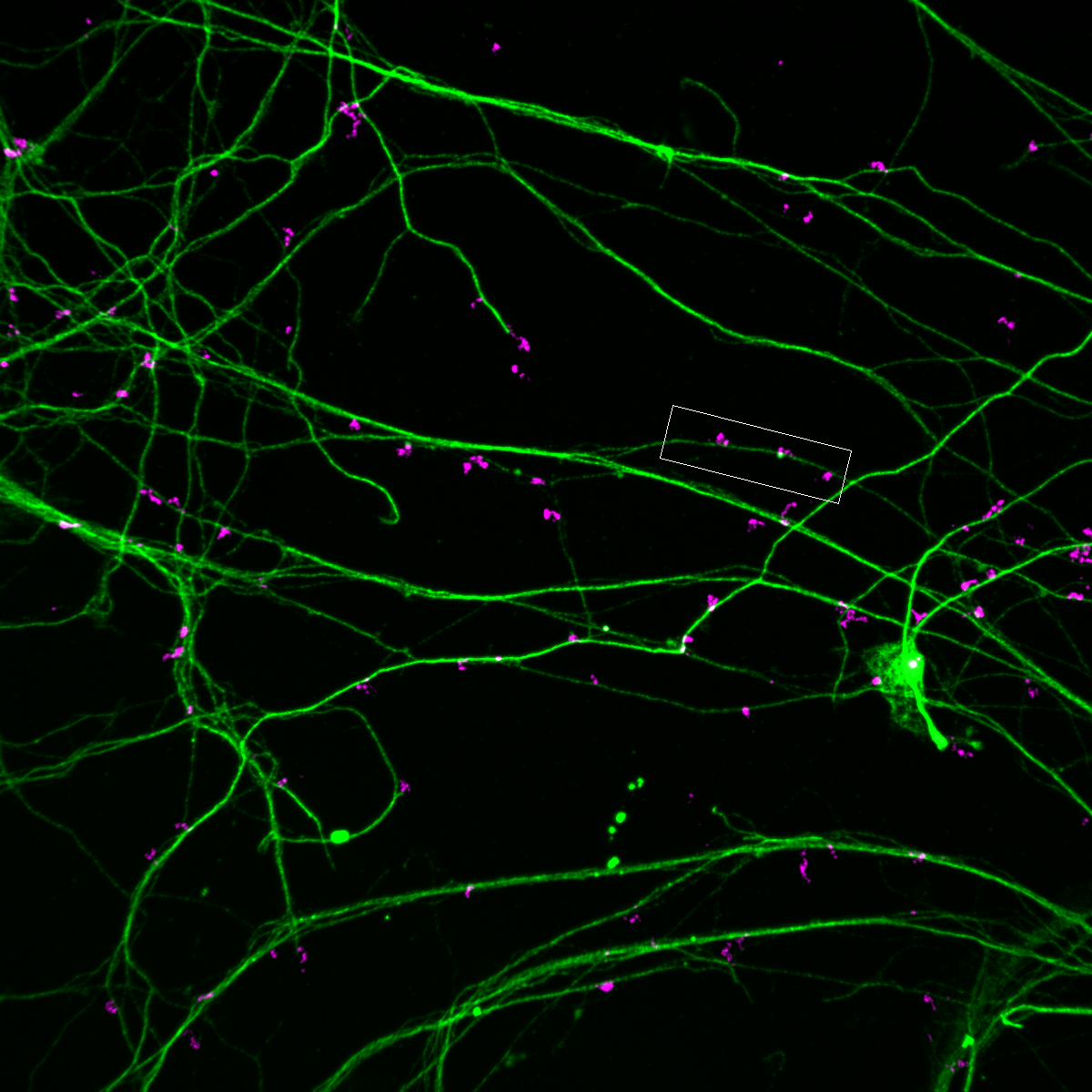

Supplement: Supplementary file 24 — Source data Fig. 2 [file 44318_2025_609_MOESM24_ESM.zip › EMBOJ-2024-119578_SourceDataForFigure2/2K/1-Control.tif]

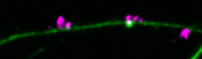

Supplement: Supplementary file 24 — Source data Fig. 2 [file 44318_2025_609_MOESM24_ESM.zip › EMBOJ-2024-119578_SourceDataForFigure2/2K/2-Control-ROI.tif]

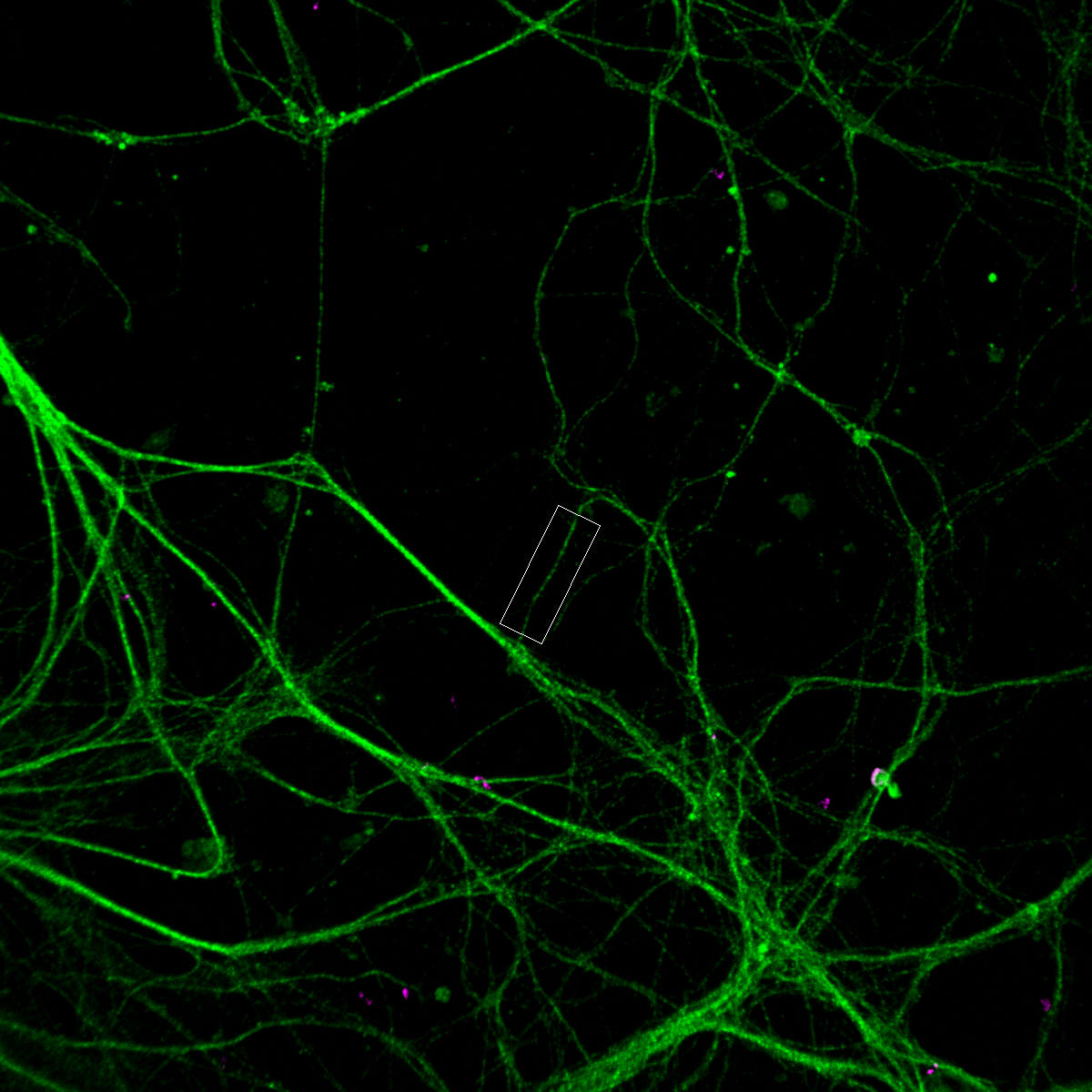

Supplement: Supplementary file 24 — Source data Fig. 2 [file 44318_2025_609_MOESM24_ESM.zip › EMBOJ-2024-119578_SourceDataForFigure2/2K/3-siANXA7-1#.tif]

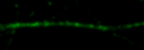

Supplement: Supplementary file 24 — Source data Fig. 2 [file 44318_2025_609_MOESM24_ESM.zip › EMBOJ-2024-119578_SourceDataForFigure2/2K/4-siANXA7-1#-ROI.tif]

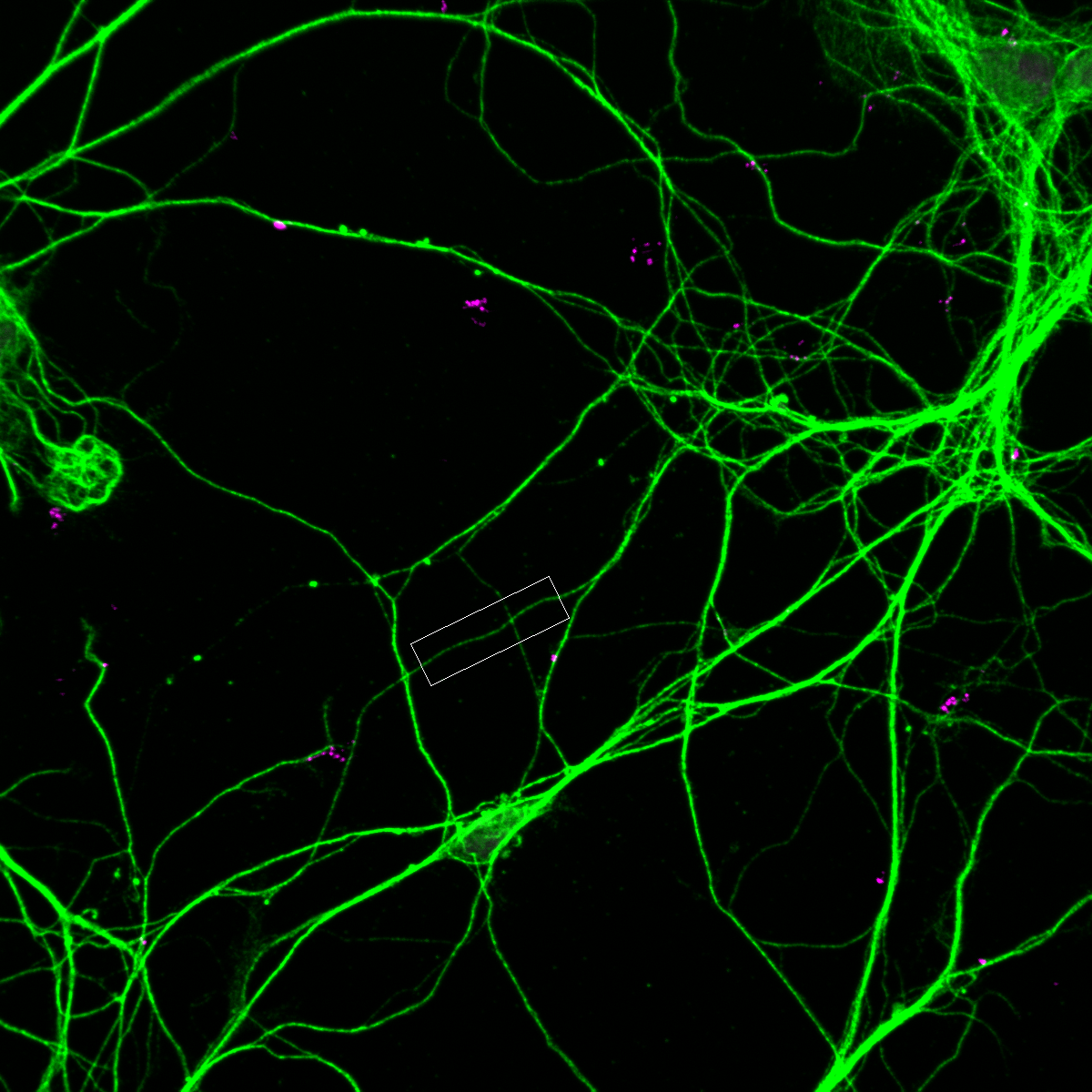

Supplement: Supplementary file 24 — Source data Fig. 2 [file 44318_2025_609_MOESM24_ESM.zip › EMBOJ-2024-119578_SourceDataForFigure2/2K/5-siANXA7-2#.tif]

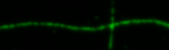

Supplement: Supplementary file 24 — Source data Fig. 2 [file 44318_2025_609_MOESM24_ESM.zip › EMBOJ-2024-119578_SourceDataForFigure2/2K/6-siANXA7-2#-ROI.tif]

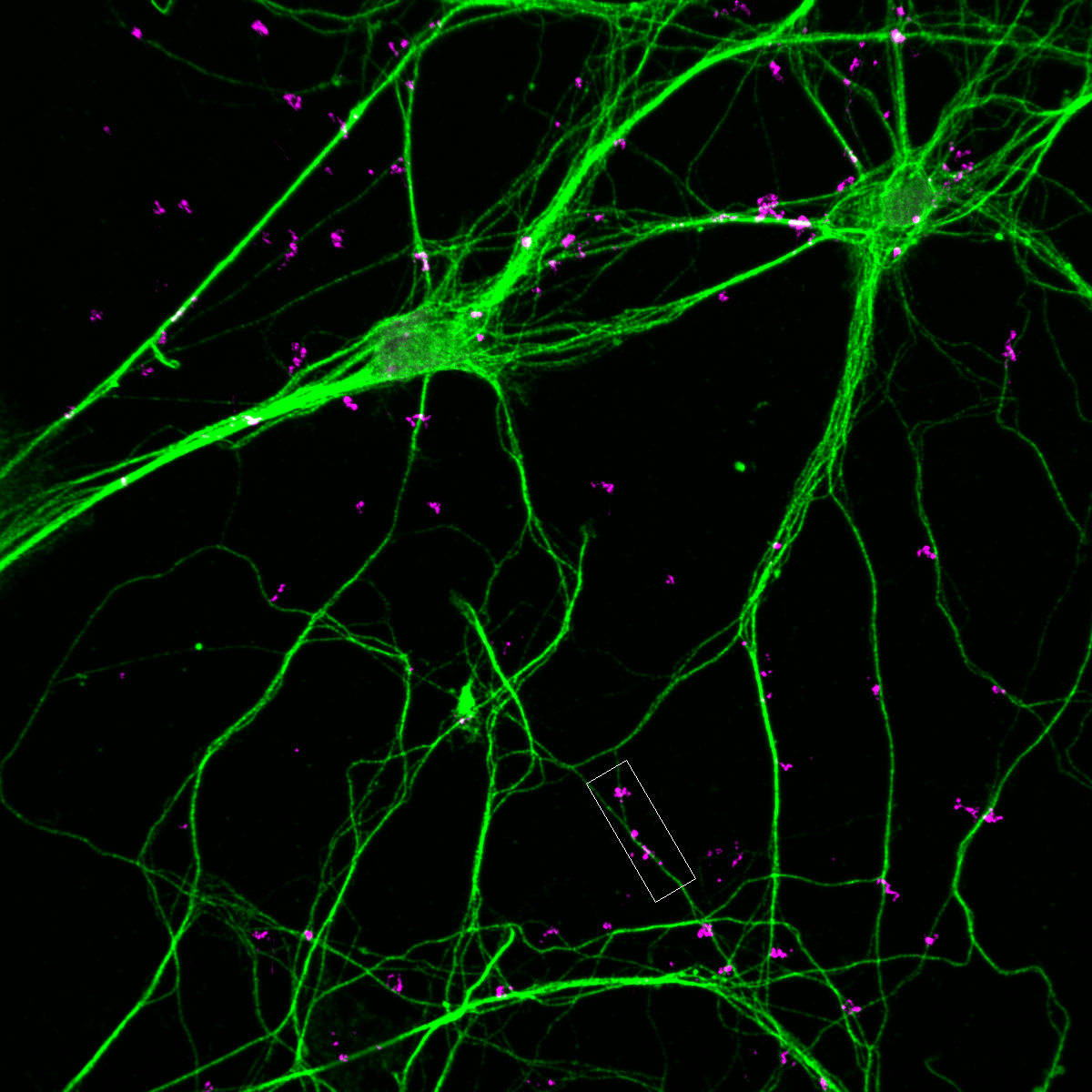

Supplement: Supplementary file 24 — Source data Fig. 2 [file 44318_2025_609_MOESM24_ESM.zip › EMBOJ-2024-119578_SourceDataForFigure2/2K/7-siA7-2#+A7-res.tif]

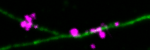

Supplement: Supplementary file 24 — Source data Fig. 2 [file 44318_2025_609_MOESM24_ESM.zip › EMBOJ-2024-119578_SourceDataForFigure2/2K/8-siA7-2#+A7-res-ROI.tif]

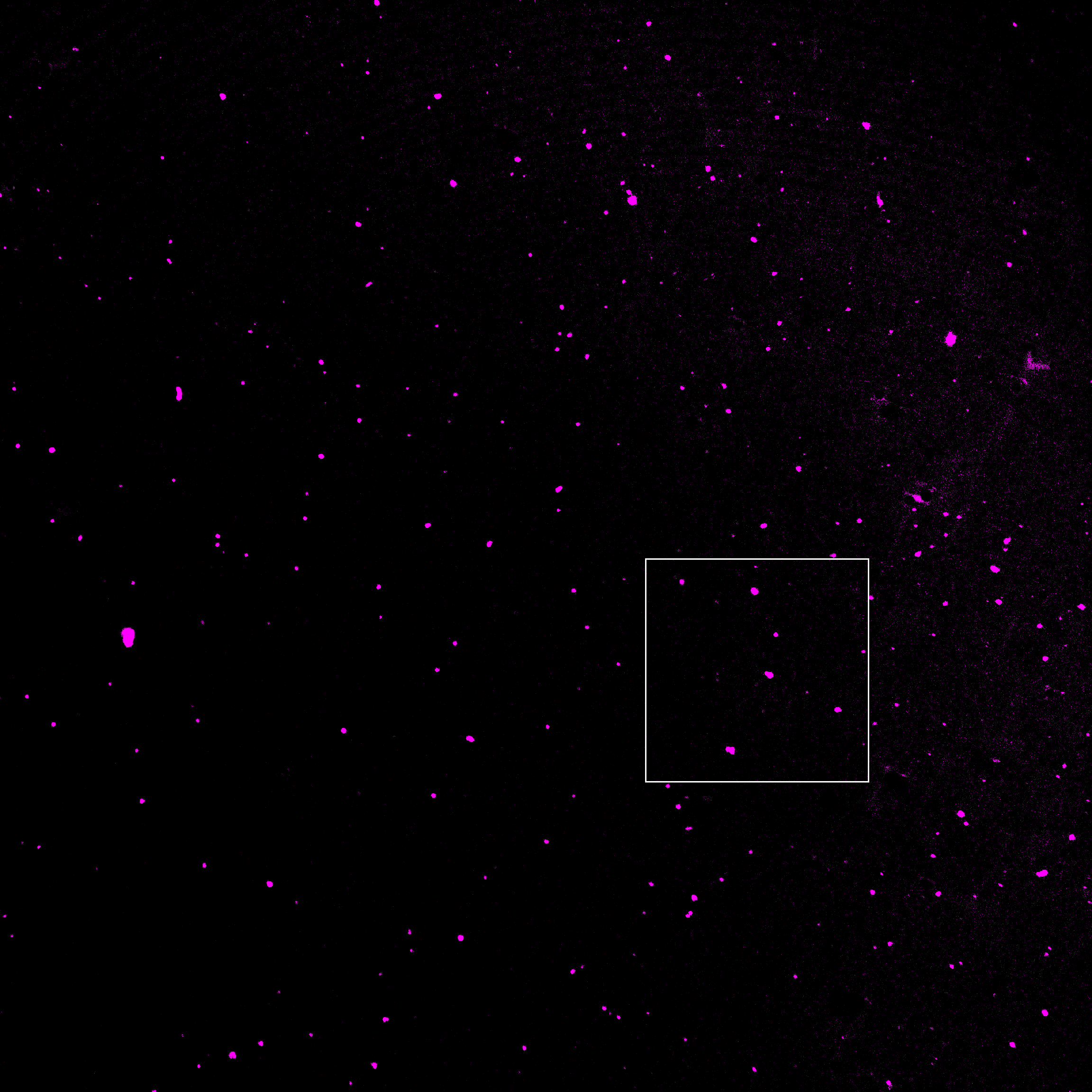

Supplement: Supplementary file 25 — Source data Fig. 3 [file 44318_2025_609_MOESM25_ESM.zip › EMBOJ-2024-119578_SourceDataForFigure3/3C/1mM Ca.tif]

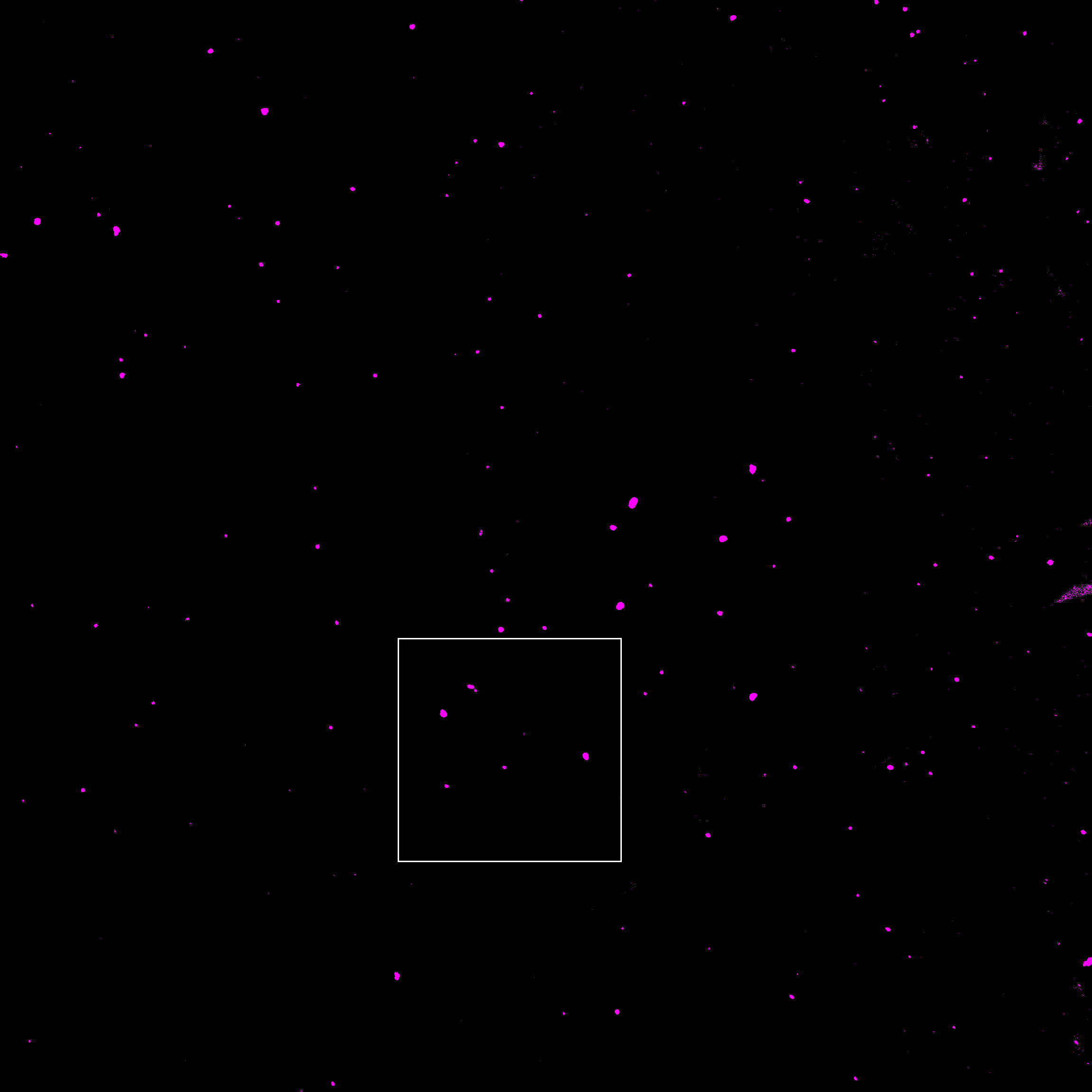

Supplement: Supplementary file 25 — Source data Fig. 3 [file 44318_2025_609_MOESM25_ESM.zip › EMBOJ-2024-119578_SourceDataForFigure3/3C/5% PEG.tif]

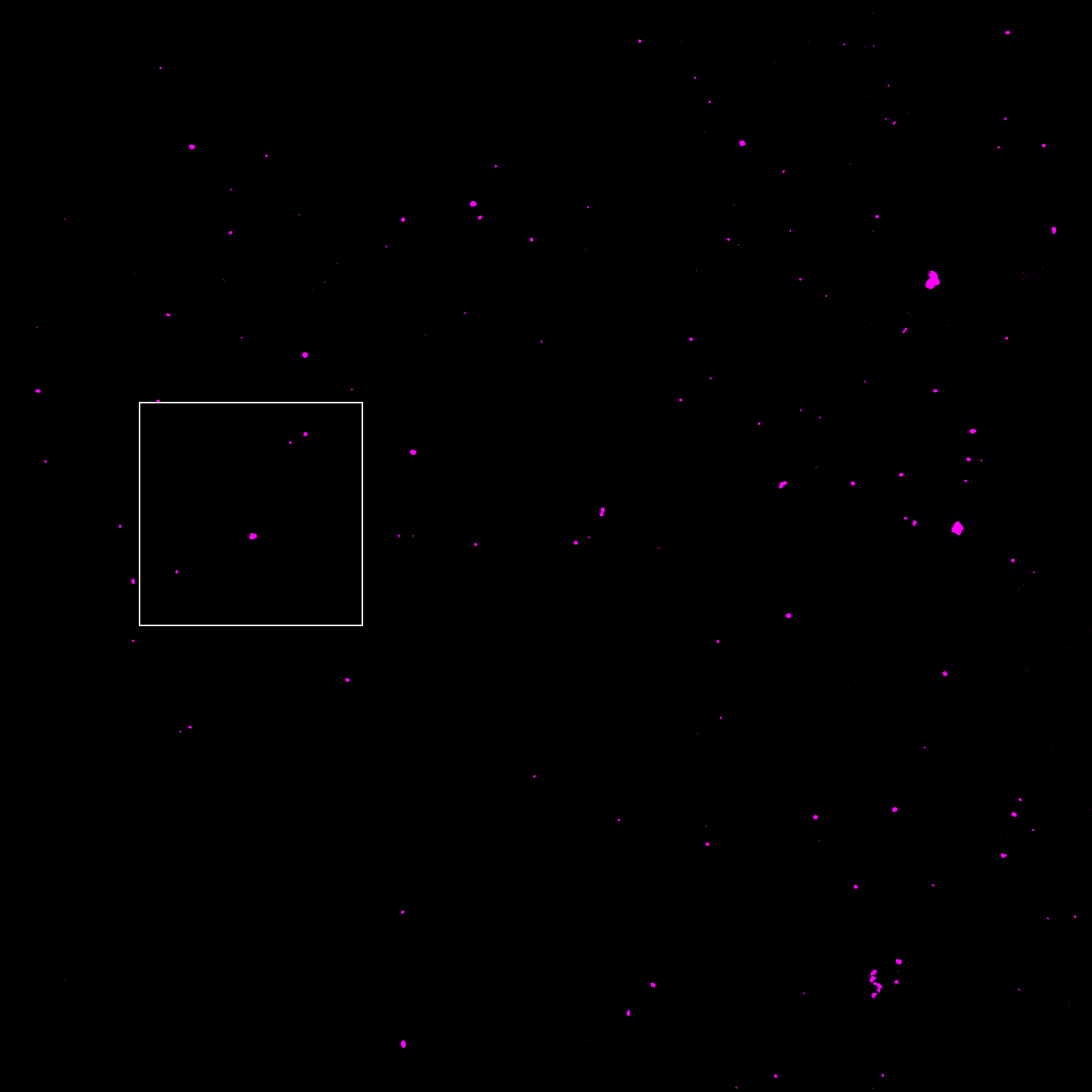

Supplement: Supplementary file 25 — Source data Fig. 3 [file 44318_2025_609_MOESM25_ESM.zip › EMBOJ-2024-119578_SourceDataForFigure3/3C/control.tif]

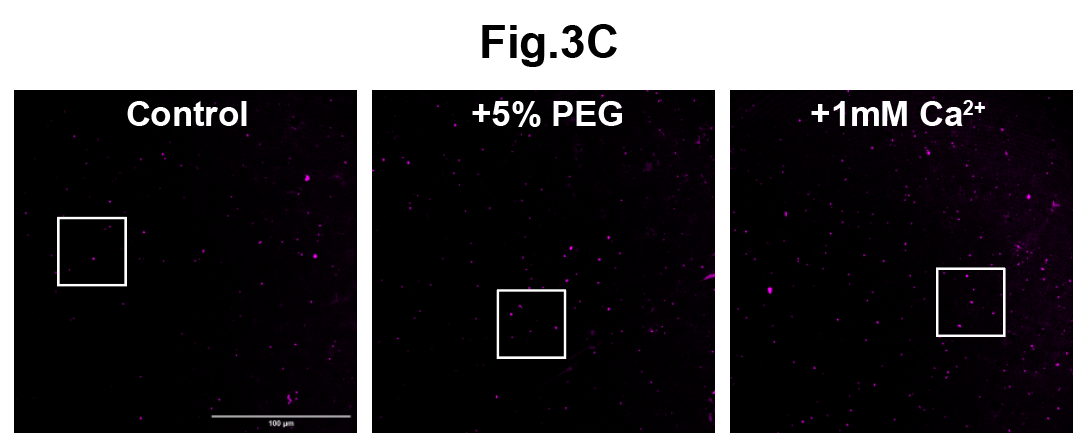

Supplement: Supplementary file 25 — Source data Fig. 3 [file 44318_2025_609_MOESM25_ESM.zip › EMBOJ-2024-119578_SourceDataForFigure3/3C/Fig.3C.tif]

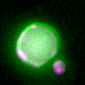

Supplement: Supplementary file 25 — Source data Fig. 3 [file 44318_2025_609_MOESM25_ESM.zip › EMBOJ-2024-119578_SourceDataForFigure3/3D/ANXA7-647 and TIA1-488 merge.tif]

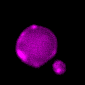

Supplement: Supplementary file 25 — Source data Fig. 3 [file 44318_2025_609_MOESM25_ESM.zip › EMBOJ-2024-119578_SourceDataForFigure3/3D/ANXA7-647.tif]

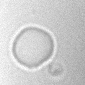

Supplement: Supplementary file 25 — Source data Fig. 3 [file 44318_2025_609_MOESM25_ESM.zip › EMBOJ-2024-119578_SourceDataForFigure3/3D/BF.tif]

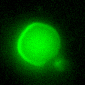

Supplement: Supplementary file 25 — Source data Fig. 3 [file 44318_2025_609_MOESM25_ESM.zip › EMBOJ-2024-119578_SourceDataForFigure3/3D/TIA1-488.tif]

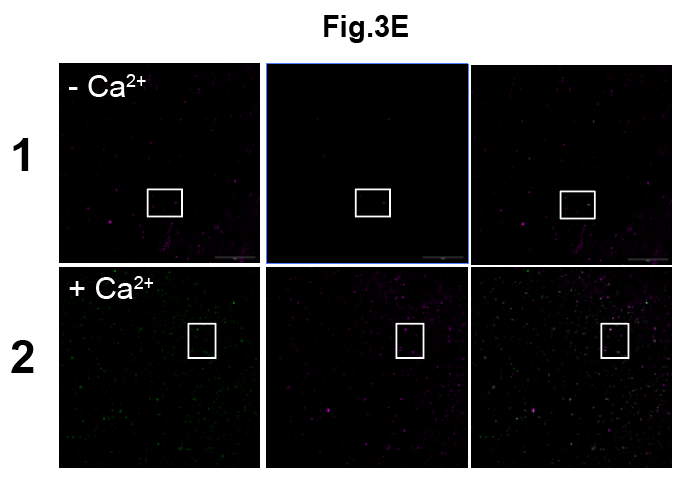

Supplement: Supplementary file 25 — Source data Fig. 3 [file 44318_2025_609_MOESM25_ESM.zip › EMBOJ-2024-119578_SourceDataForFigure3/3E/0-Fig.3E.tif]

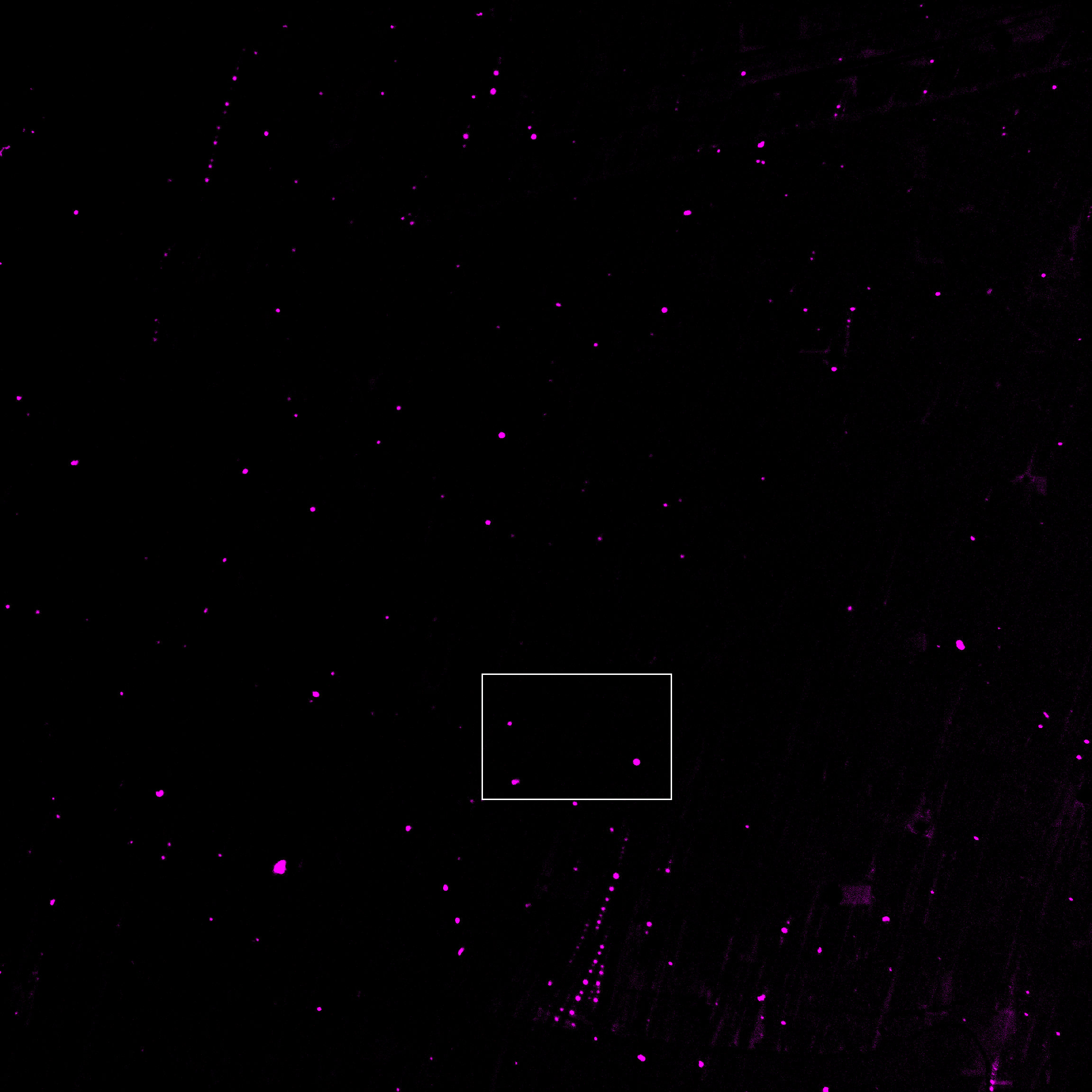

Supplement: Supplementary file 25 — Source data Fig. 3 [file 44318_2025_609_MOESM25_ESM.zip › EMBOJ-2024-119578_SourceDataForFigure3/3E/1-ANXA7-647.tif]

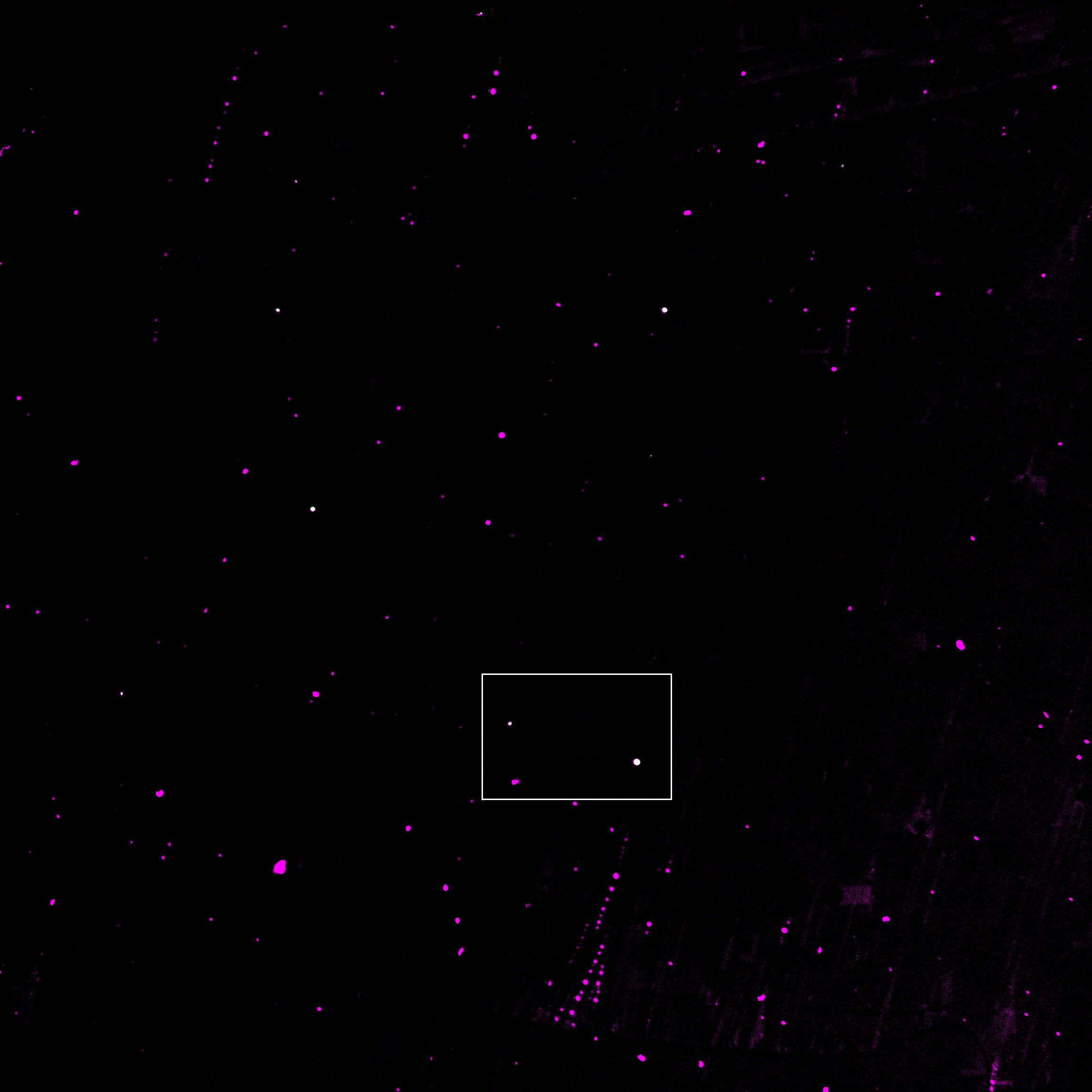

Supplement: Supplementary file 25 — Source data Fig. 3 [file 44318_2025_609_MOESM25_ESM.zip › EMBOJ-2024-119578_SourceDataForFigure3/3E/1-Merge.tif]

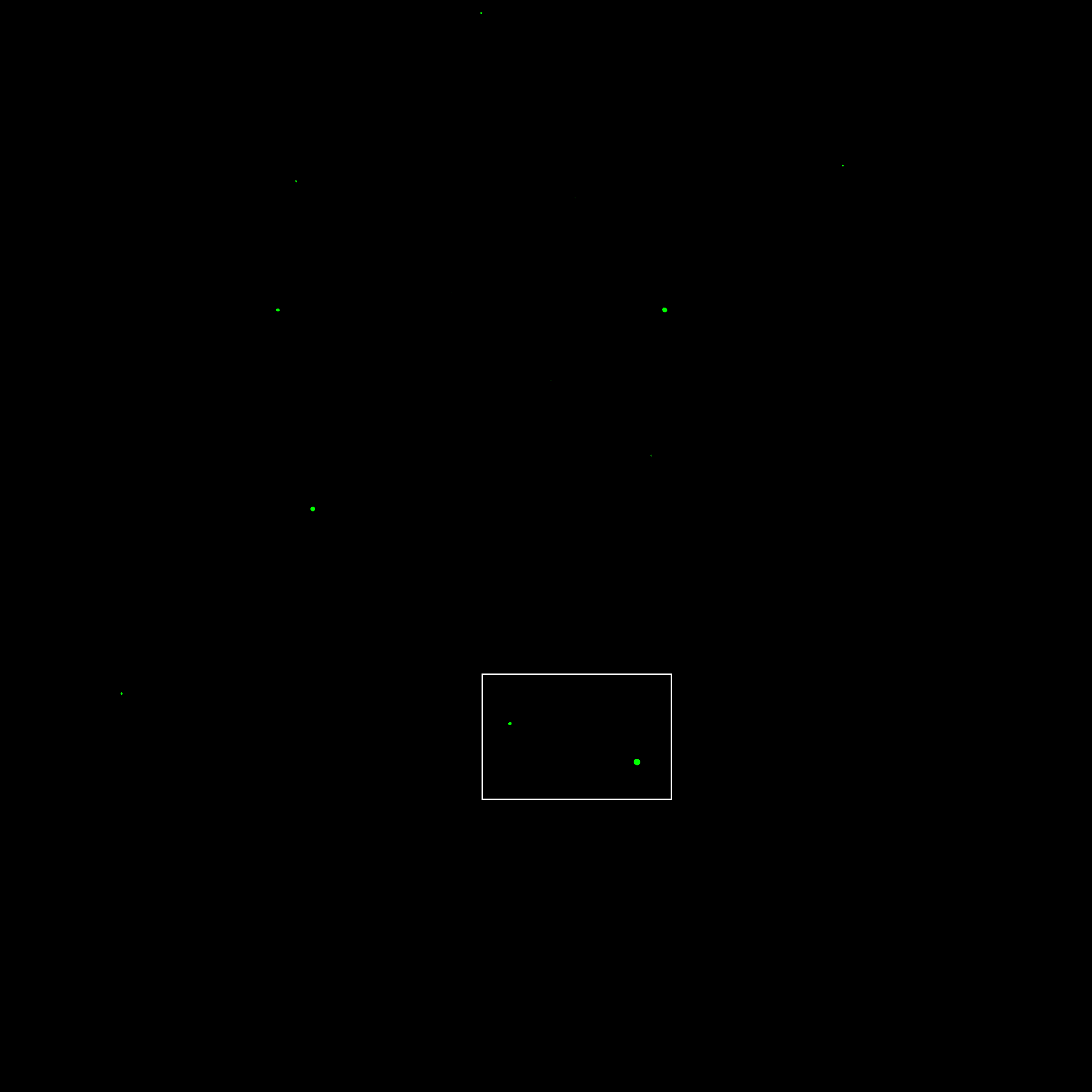

Supplement: Supplementary file 25 — Source data Fig. 3 [file 44318_2025_609_MOESM25_ESM.zip › EMBOJ-2024-119578_SourceDataForFigure3/3E/1-TIA1-488.tif]

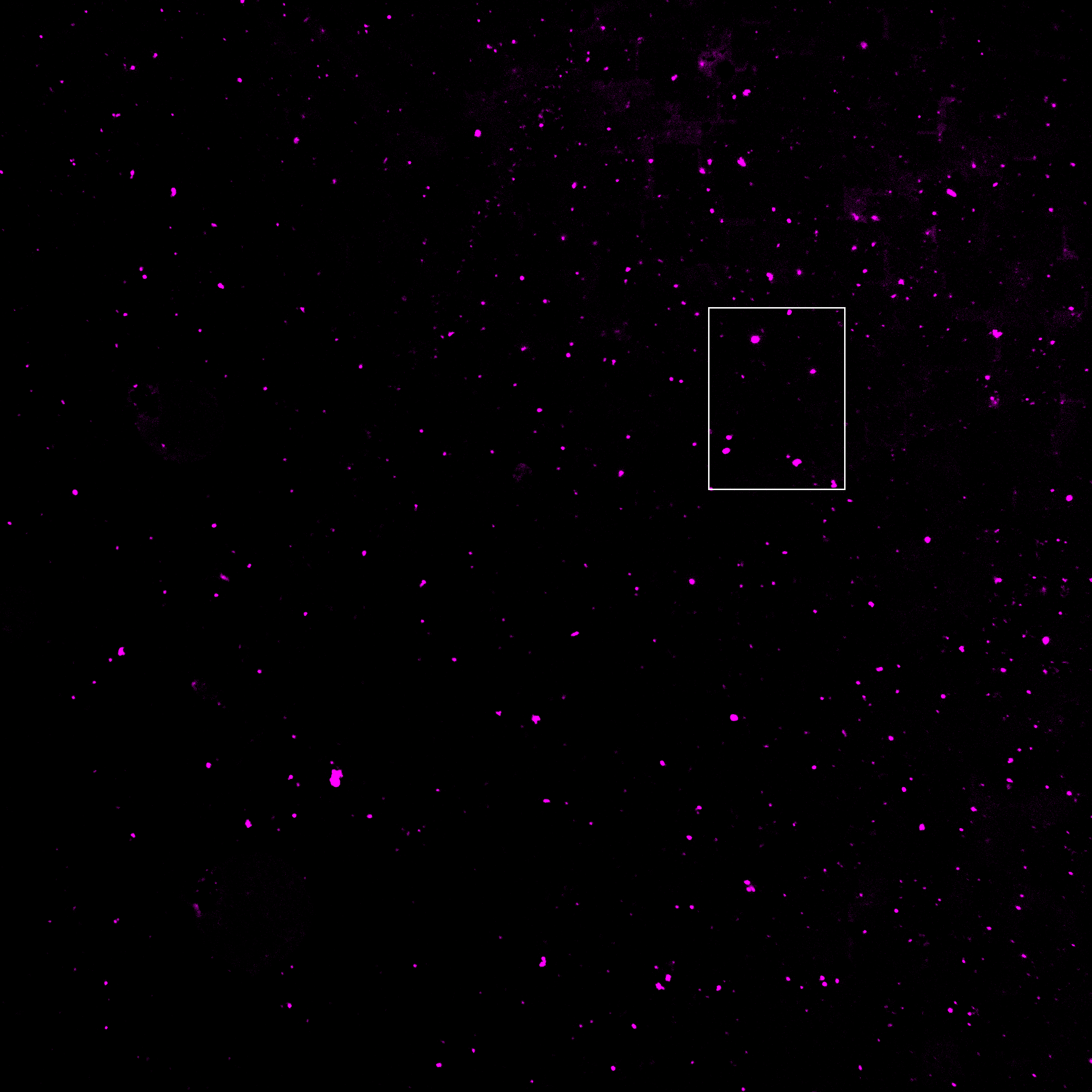

Supplement: Supplementary file 25 — Source data Fig. 3 [file 44318_2025_609_MOESM25_ESM.zip › EMBOJ-2024-119578_SourceDataForFigure3/3E/2-ANXA7-647.tif]

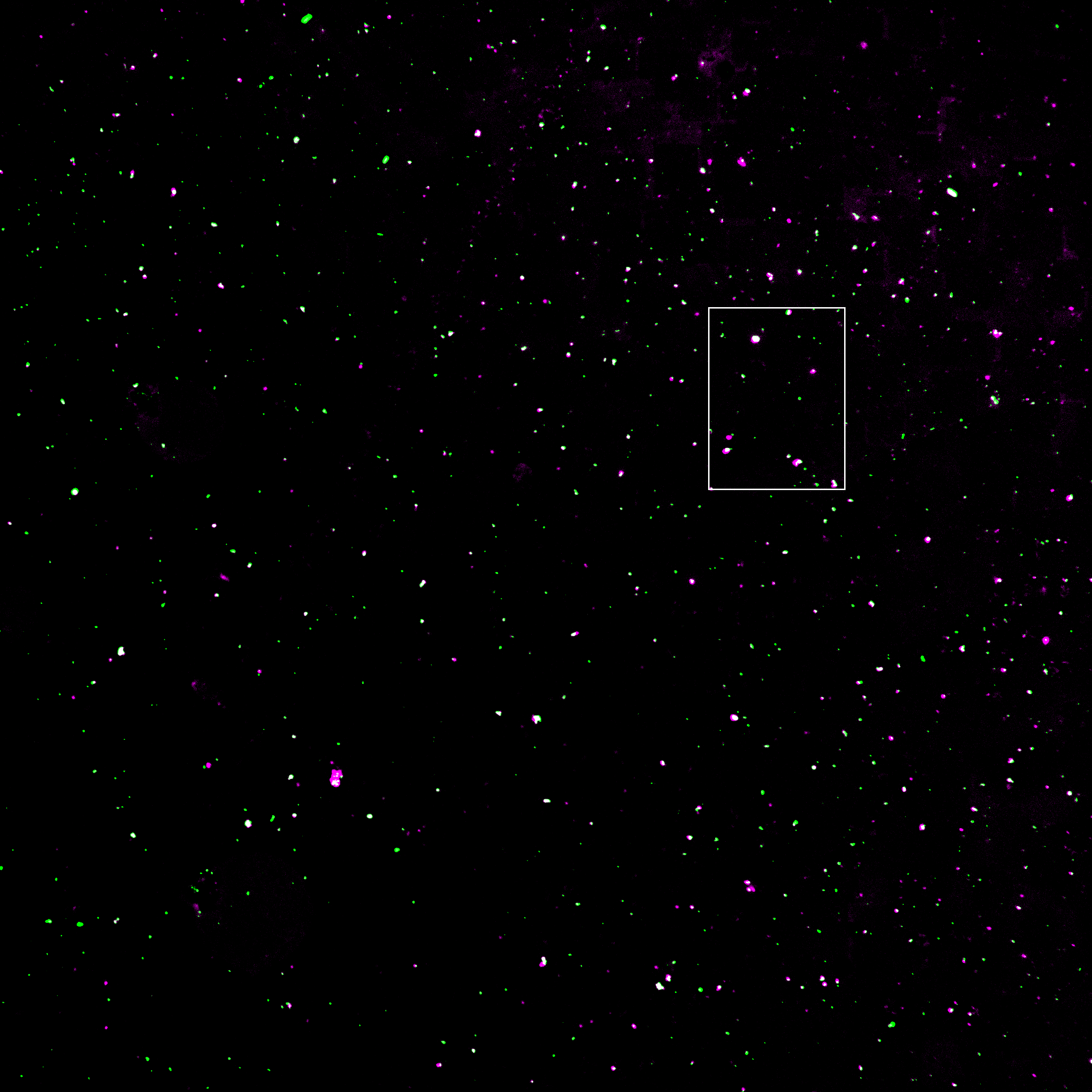

Supplement: Supplementary file 25 — Source data Fig. 3 [file 44318_2025_609_MOESM25_ESM.zip › EMBOJ-2024-119578_SourceDataForFigure3/3E/2-Merge.tif]

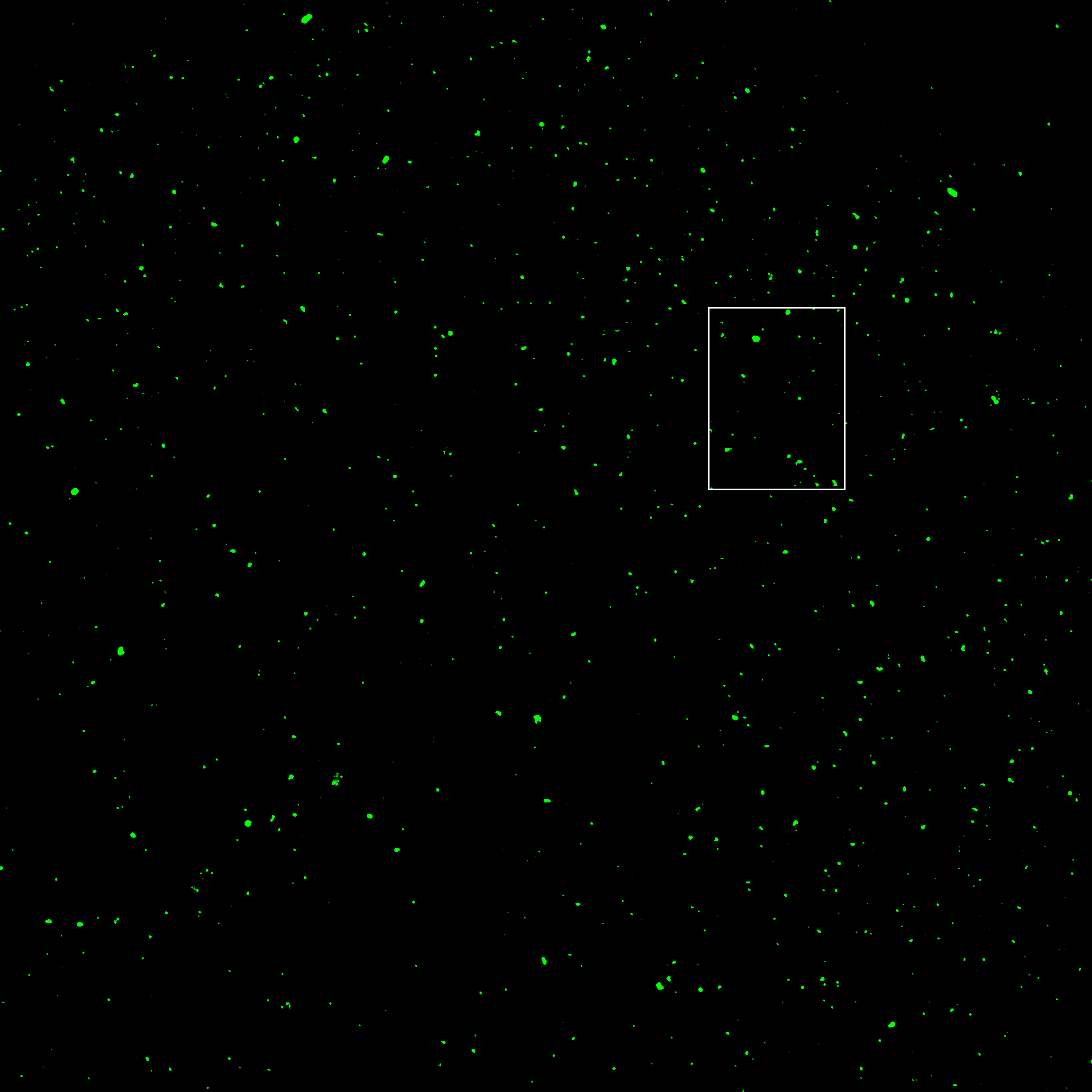

Supplement: Supplementary file 25 — Source data Fig. 3 [file 44318_2025_609_MOESM25_ESM.zip › EMBOJ-2024-119578_SourceDataForFigure3/3E/2-TIA1-488.tif]

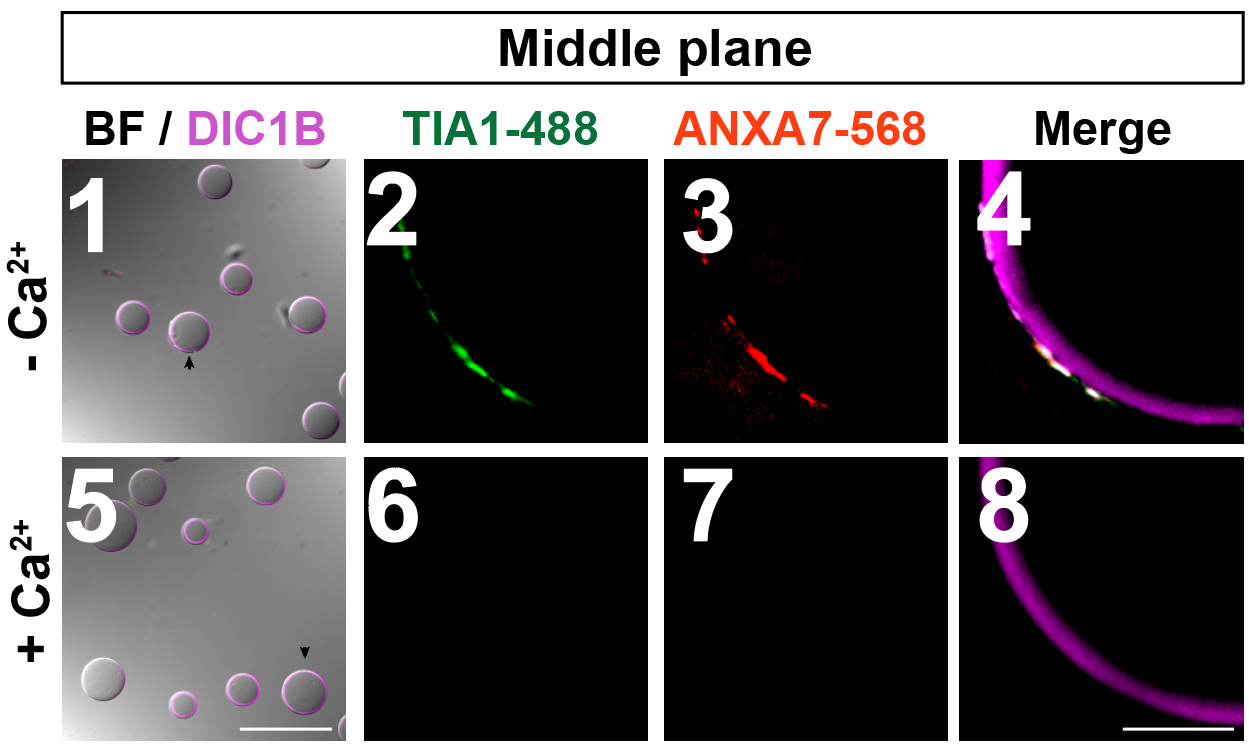

Supplement: Supplementary file 25 — Source data Fig. 3 [file 44318_2025_609_MOESM25_ESM.zip › EMBOJ-2024-119578_SourceDataForFigure3/3H/0-Fig. 3H.tif]

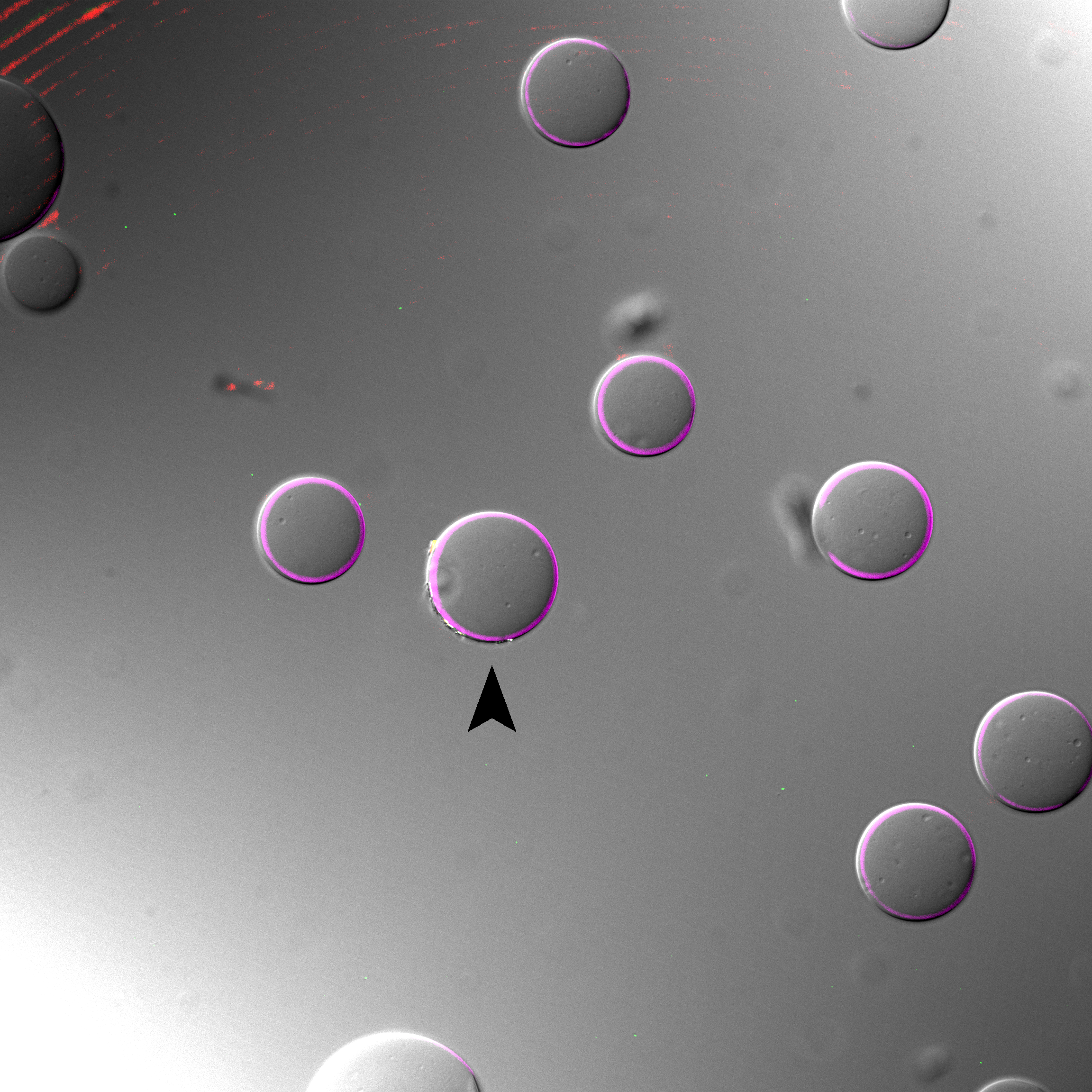

Supplement: Supplementary file 25 — Source data Fig. 3 [file 44318_2025_609_MOESM25_ESM.zip › EMBOJ-2024-119578_SourceDataForFigure3/3H/1-BF.tif]

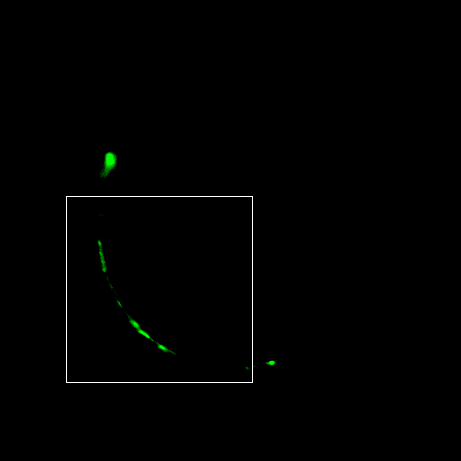

Supplement: Supplementary file 25 — Source data Fig. 3 [file 44318_2025_609_MOESM25_ESM.zip › EMBOJ-2024-119578_SourceDataForFigure3/3H/2-TIA1-488.tif]

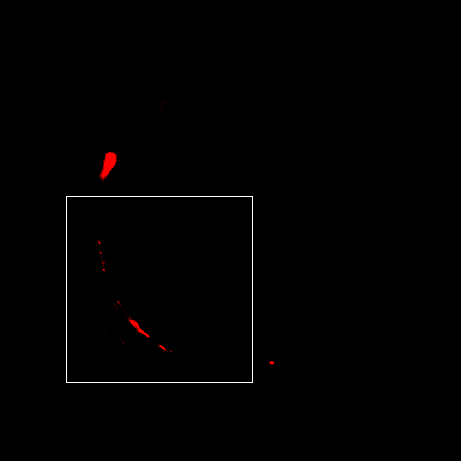

Supplement: Supplementary file 25 — Source data Fig. 3 [file 44318_2025_609_MOESM25_ESM.zip › EMBOJ-2024-119578_SourceDataForFigure3/3H/3-ANXA7-568.tif]

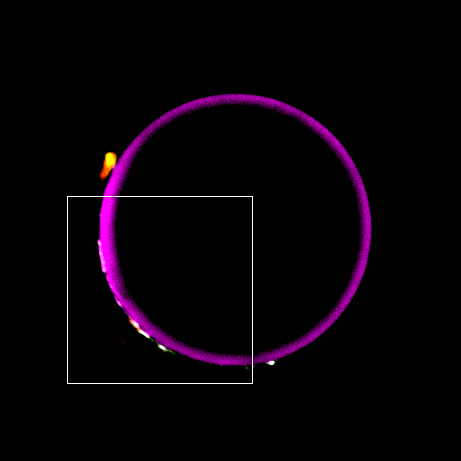

Supplement: Supplementary file 25 — Source data Fig. 3 [file 44318_2025_609_MOESM25_ESM.zip › EMBOJ-2024-119578_SourceDataForFigure3/3H/4-Merge.tif]

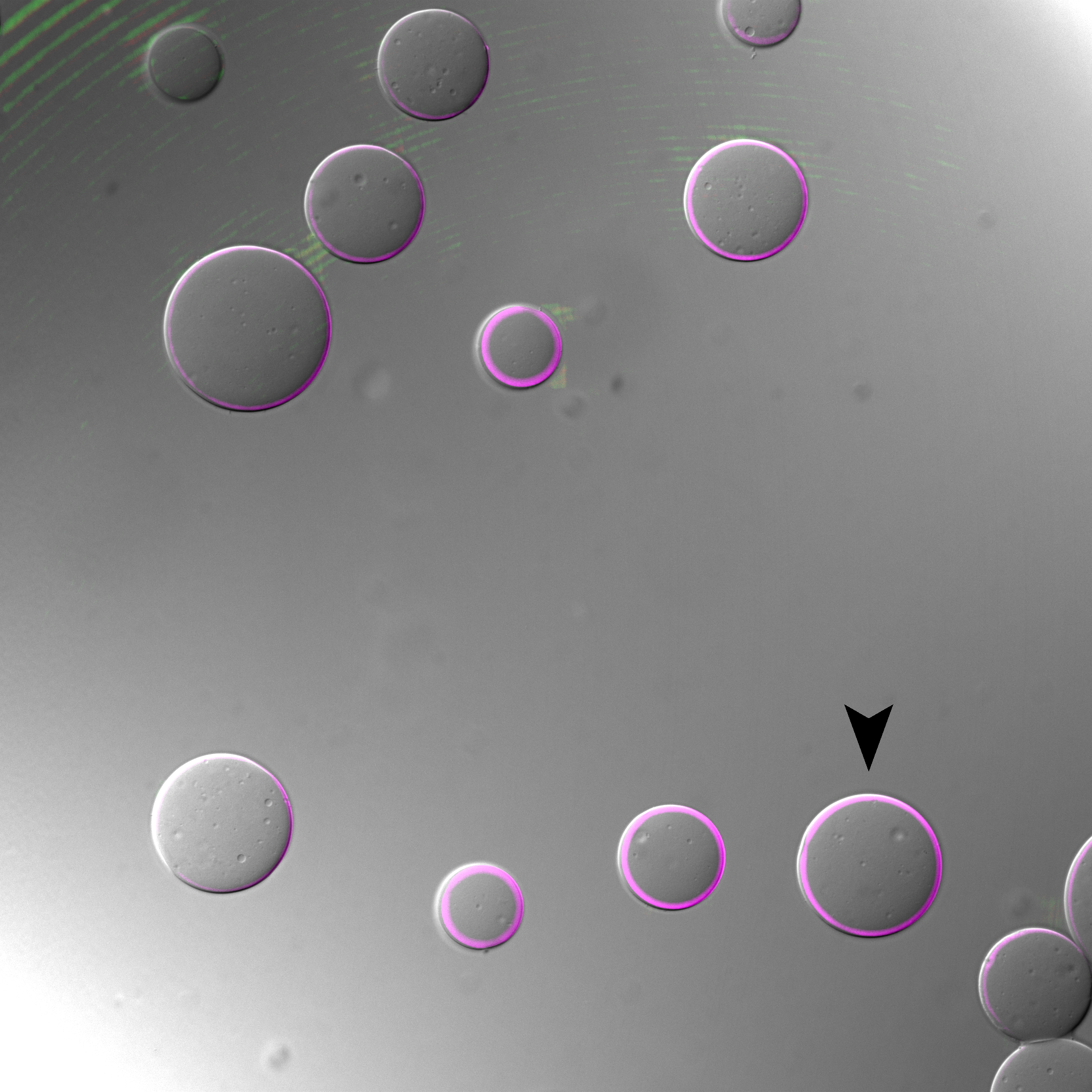

Supplement: Supplementary file 25 — Source data Fig. 3 [file 44318_2025_609_MOESM25_ESM.zip › EMBOJ-2024-119578_SourceDataForFigure3/3H/5-BF.tif]

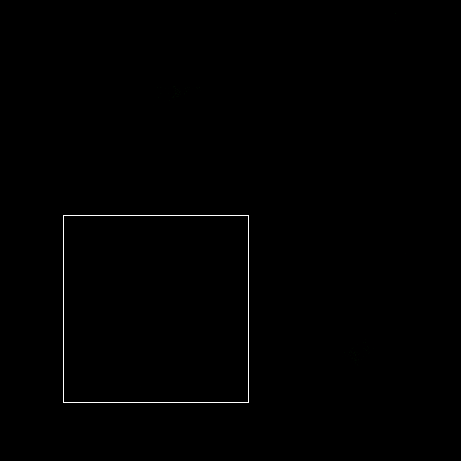

Supplement: Supplementary file 25 — Source data Fig. 3 [file 44318_2025_609_MOESM25_ESM.zip › EMBOJ-2024-119578_SourceDataForFigure3/3H/6-TIA1-488.tif]

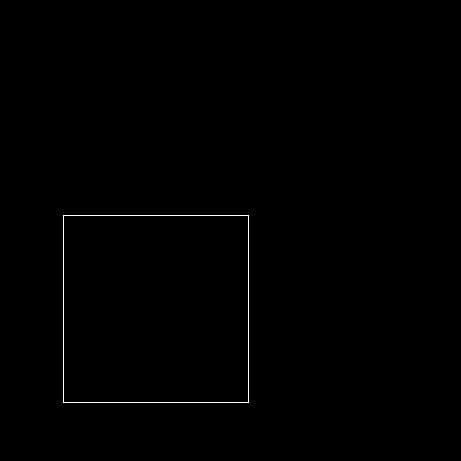

Supplement: Supplementary file 25 — Source data Fig. 3 [file 44318_2025_609_MOESM25_ESM.zip › EMBOJ-2024-119578_SourceDataForFigure3/3H/7-ANXA7-568.tif]

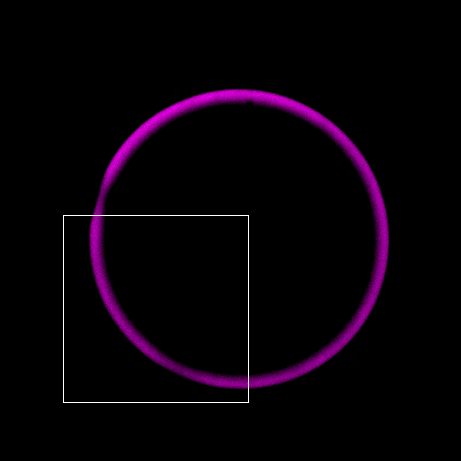

Supplement: Supplementary file 25 — Source data Fig. 3 [file 44318_2025_609_MOESM25_ESM.zip › EMBOJ-2024-119578_SourceDataForFigure3/3H/8-Merge.tif]

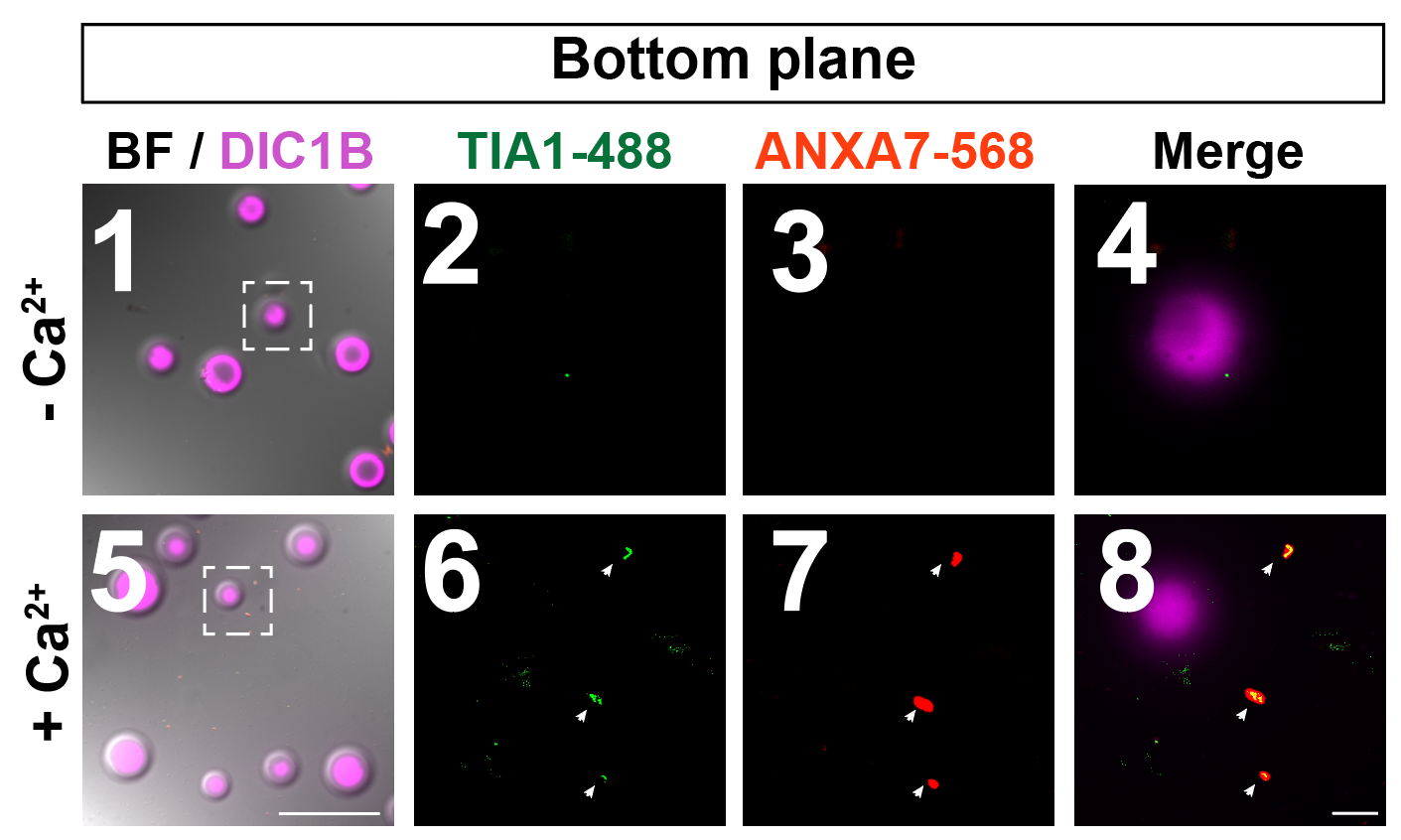

Supplement: Supplementary file 25 — Source data Fig. 3 [file 44318_2025_609_MOESM25_ESM.zip › EMBOJ-2024-119578_SourceDataForFigure3/3I/0-Fig. 3I.tif]

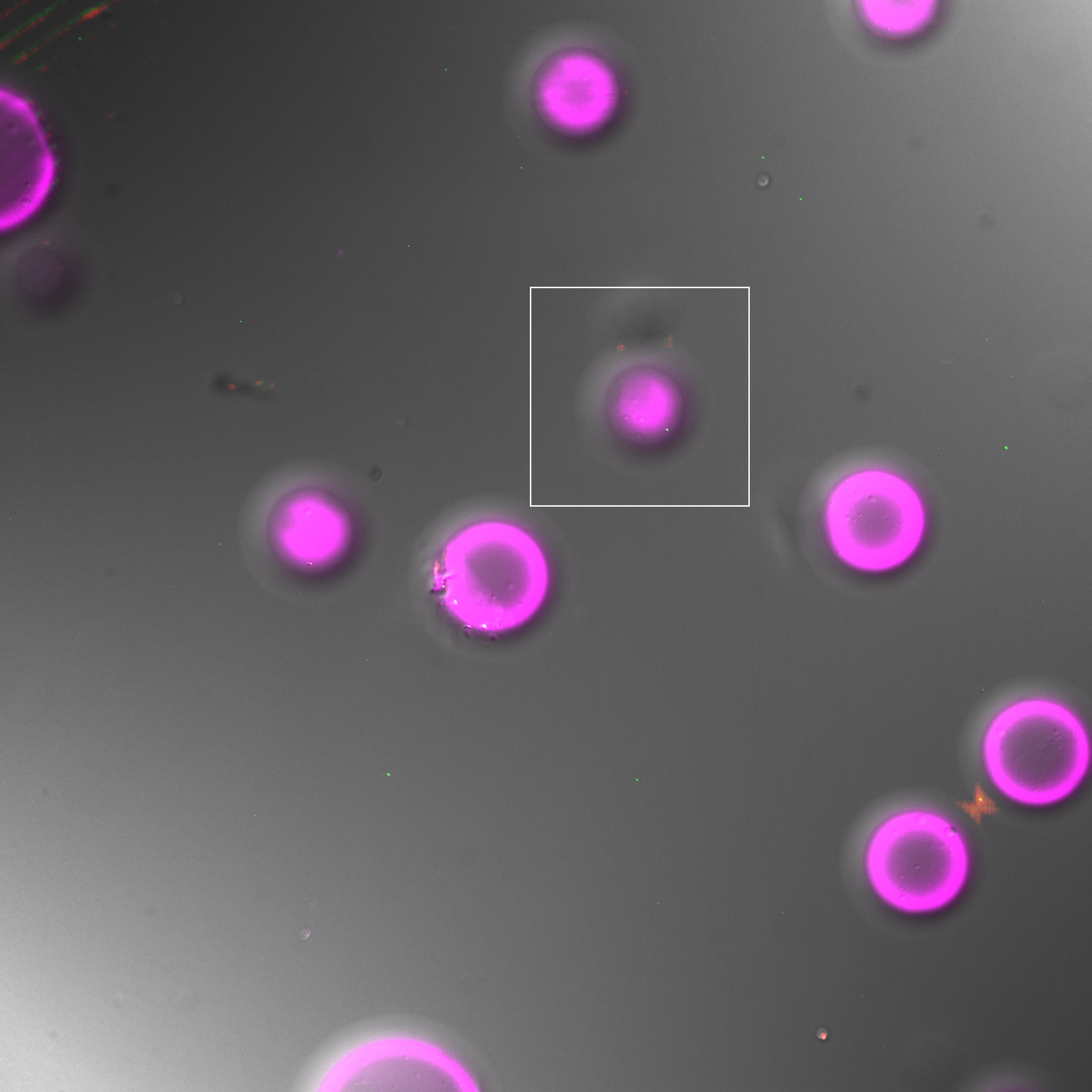

Supplement: Supplementary file 25 — Source data Fig. 3 [file 44318_2025_609_MOESM25_ESM.zip › EMBOJ-2024-119578_SourceDataForFigure3/3I/1-BF.tif]

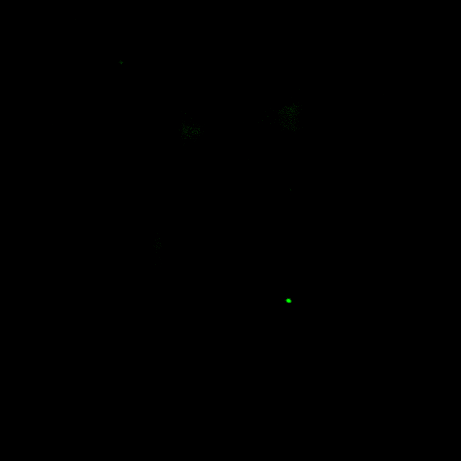

Supplement: Supplementary file 25 — Source data Fig. 3 [file 44318_2025_609_MOESM25_ESM.zip › EMBOJ-2024-119578_SourceDataForFigure3/3I/2-TIA1-488.tif]

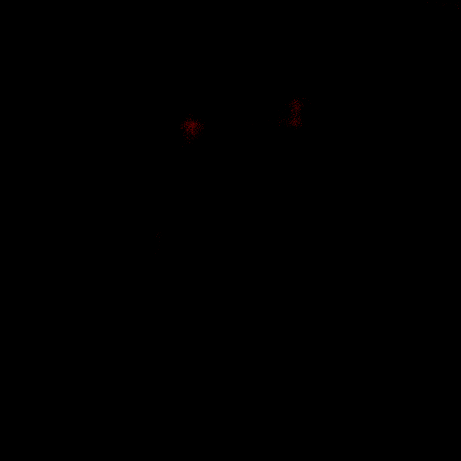

Supplement: Supplementary file 25 — Source data Fig. 3 [file 44318_2025_609_MOESM25_ESM.zip › EMBOJ-2024-119578_SourceDataForFigure3/3I/3-ANXA7-568.tif]

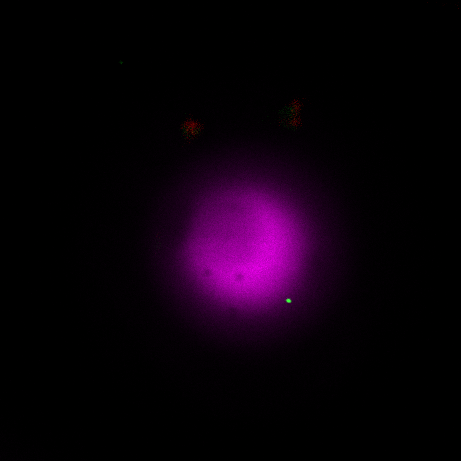

Supplement: Supplementary file 25 — Source data Fig. 3 [file 44318_2025_609_MOESM25_ESM.zip › EMBOJ-2024-119578_SourceDataForFigure3/3I/4-Merge.tif]
