# Supplementary figures and images for: Annexin A7 enhances TIA1 axonal trafficking to counteract pathological aggregation in neurons (part 2 of 5)
Source: EMBO J. 2025 Nov 3;44(24):7477–512. doi: 10.1038/s44318-025-00609-8 (PMC12706091; doi:10.1038/s44318-025-00609-8)

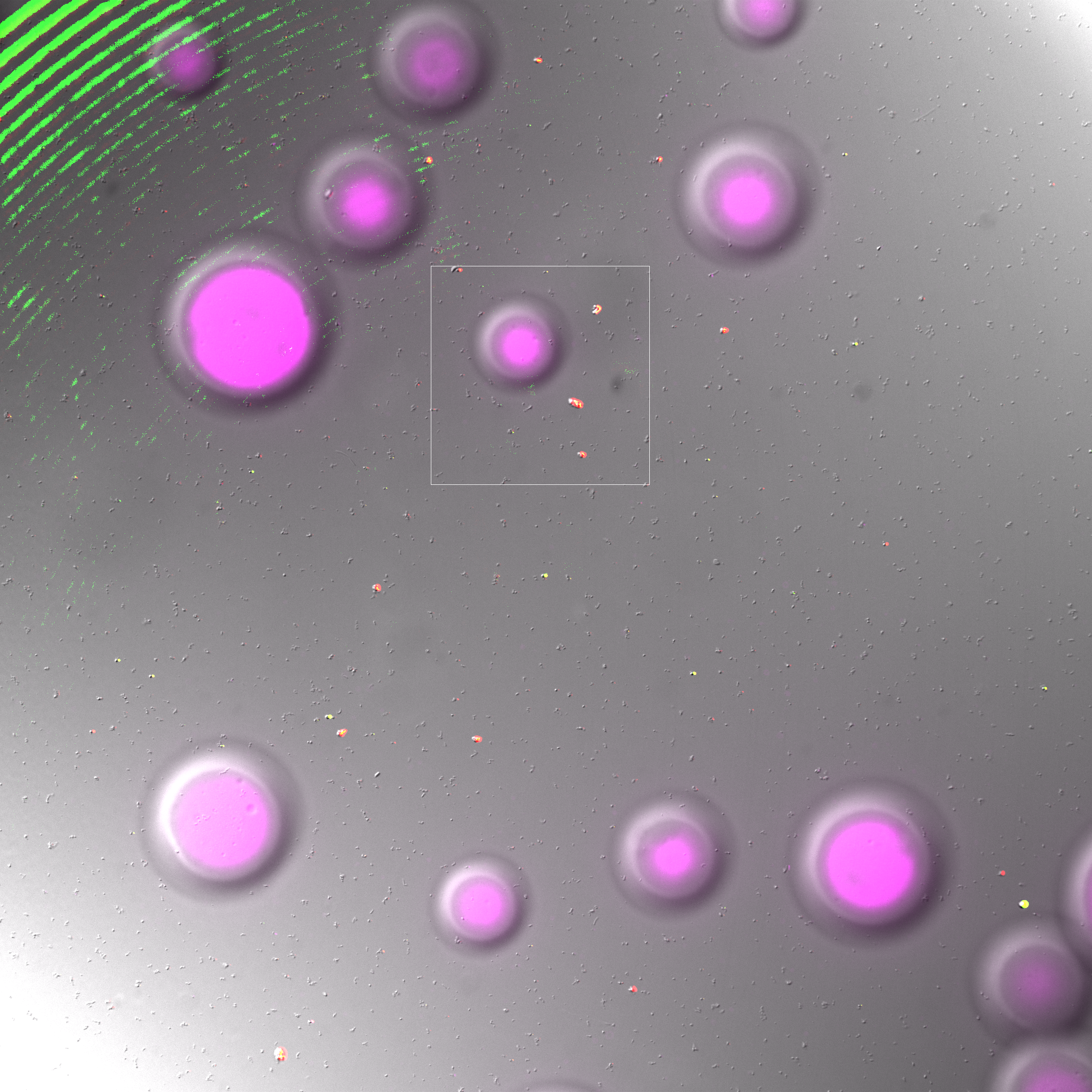

Supplement: Supplementary file 25 — Source data Fig. 3 [file 44318_2025_609_MOESM25_ESM.zip › EMBOJ-2024-119578_SourceDataForFigure3/3I/5-BF.tif]

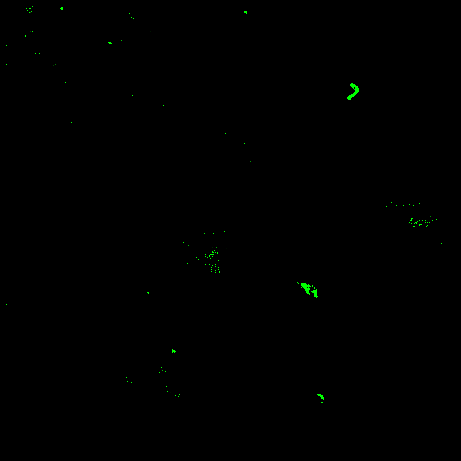

Supplement: Supplementary file 25 — Source data Fig. 3 [file 44318_2025_609_MOESM25_ESM.zip › EMBOJ-2024-119578_SourceDataForFigure3/3I/6-TIA1-488.tif]

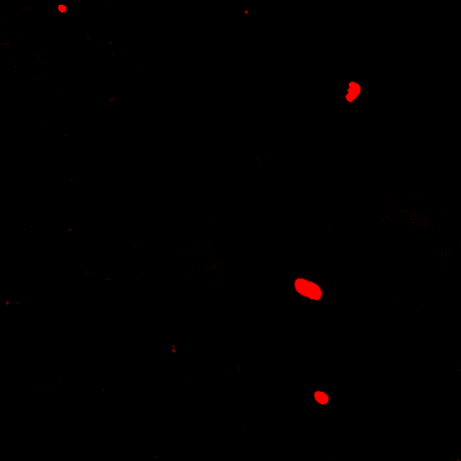

Supplement: Supplementary file 25 — Source data Fig. 3 [file 44318_2025_609_MOESM25_ESM.zip › EMBOJ-2024-119578_SourceDataForFigure3/3I/7-ANXA7-568.tif]

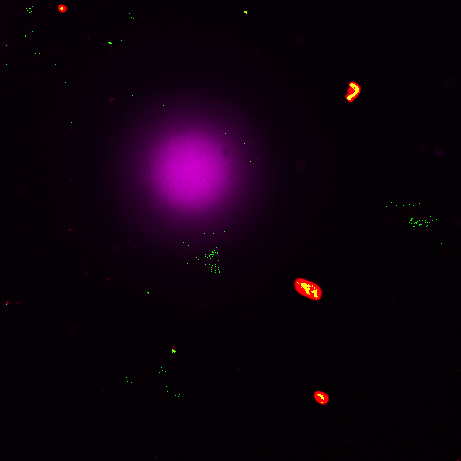

Supplement: Supplementary file 25 — Source data Fig. 3 [file 44318_2025_609_MOESM25_ESM.zip › EMBOJ-2024-119578_SourceDataForFigure3/3I/8-Merge.tif]

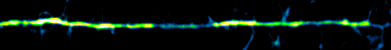

Supplement: Supplementary file 26 — Source data Fig. 4 [file 44318_2025_609_MOESM26_ESM.zip › EMBOJ-2024-119578_SourceDataForFigure4/4A/+High K 0min.tif]

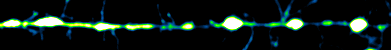

Supplement: Supplementary file 26 — Source data Fig. 4 [file 44318_2025_609_MOESM26_ESM.zip › EMBOJ-2024-119578_SourceDataForFigure4/4A/+High K 10min.tif]

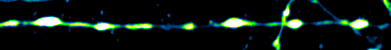

Supplement: Supplementary file 26 — Source data Fig. 4 [file 44318_2025_609_MOESM26_ESM.zip › EMBOJ-2024-119578_SourceDataForFigure4/4A/+High K 2min30s.tif]

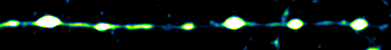

Supplement: Supplementary file 26 — Source data Fig. 4 [file 44318_2025_609_MOESM26_ESM.zip › EMBOJ-2024-119578_SourceDataForFigure4/4A/+High K 5min.tif]

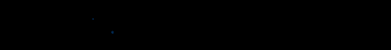

Supplement: Supplementary file 26 — Source data Fig. 4 [file 44318_2025_609_MOESM26_ESM.zip › EMBOJ-2024-119578_SourceDataForFigure4/4A/Before.tif]

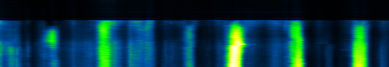

Supplement: Supplementary file 26 — Source data Fig. 4 [file 44318_2025_609_MOESM26_ESM.zip › EMBOJ-2024-119578_SourceDataForFigure4/4B/Kymograph.tif]

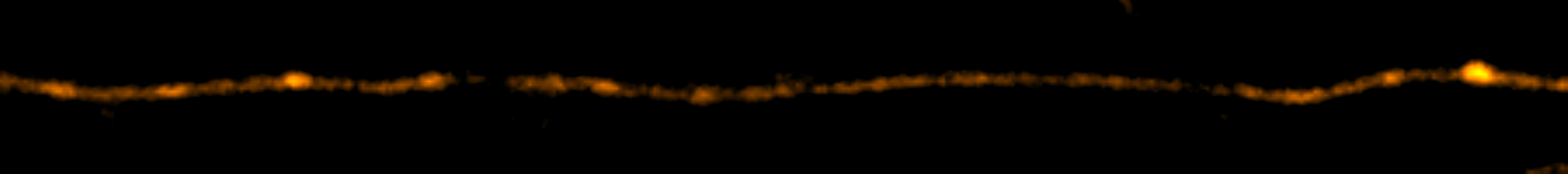

Supplement: Supplementary file 26 — Source data Fig. 4 [file 44318_2025_609_MOESM26_ESM.zip › EMBOJ-2024-119578_SourceDataForFigure4/4C/+High K/+High K 0min.tif]

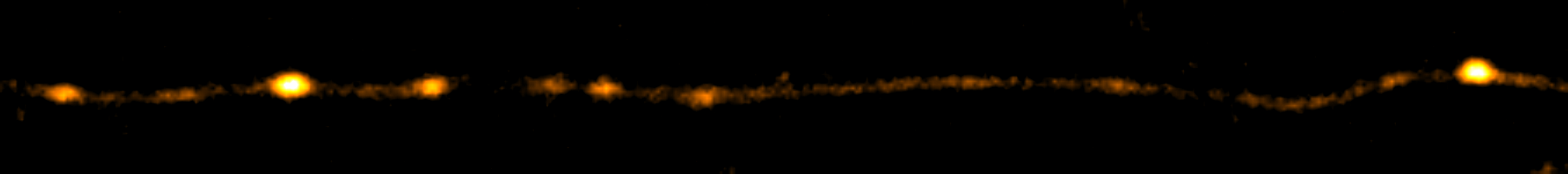

Supplement: Supplementary file 26 — Source data Fig. 4 [file 44318_2025_609_MOESM26_ESM.zip › EMBOJ-2024-119578_SourceDataForFigure4/4C/+High K/+High K 4min.tif]

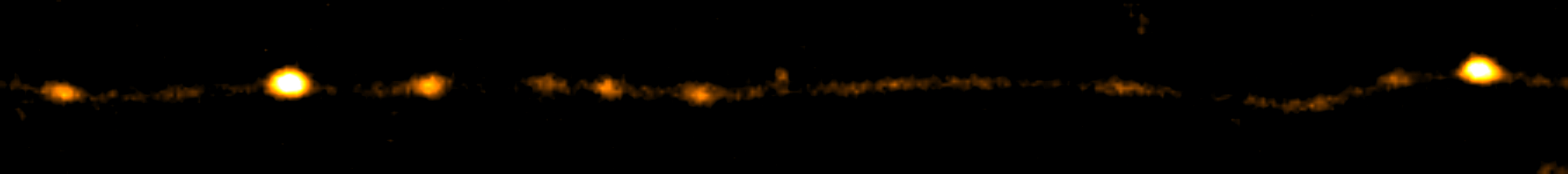

Supplement: Supplementary file 26 — Source data Fig. 4 [file 44318_2025_609_MOESM26_ESM.zip › EMBOJ-2024-119578_SourceDataForFigure4/4C/+High K/+High K 6min.tif]

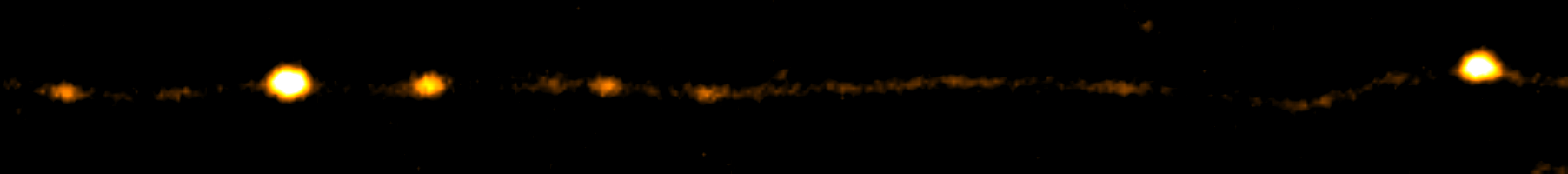

Supplement: Supplementary file 26 — Source data Fig. 4 [file 44318_2025_609_MOESM26_ESM.zip › EMBOJ-2024-119578_SourceDataForFigure4/4C/+High K/+High K 8min.tif]

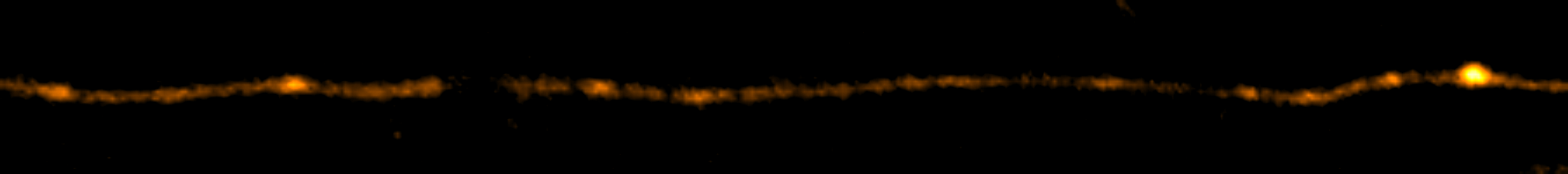

Supplement: Supplementary file 26 — Source data Fig. 4 [file 44318_2025_609_MOESM26_ESM.zip › EMBOJ-2024-119578_SourceDataForFigure4/4C/+High K/Before.tif]

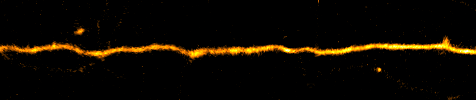

Supplement: Supplementary file 26 — Source data Fig. 4 [file 44318_2025_609_MOESM26_ESM.zip › EMBOJ-2024-119578_SourceDataForFigure4/4C/+High K with EDTA/+High K with EDTA 0min.tif]

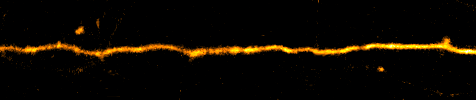

Supplement: Supplementary file 26 — Source data Fig. 4 [file 44318_2025_609_MOESM26_ESM.zip › EMBOJ-2024-119578_SourceDataForFigure4/4C/+High K with EDTA/+High K with EDTA 4min.tif]

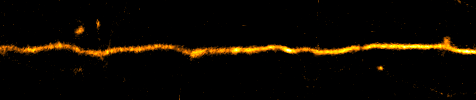

Supplement: Supplementary file 26 — Source data Fig. 4 [file 44318_2025_609_MOESM26_ESM.zip › EMBOJ-2024-119578_SourceDataForFigure4/4C/+High K with EDTA/+High K with EDTA 6min.tif]

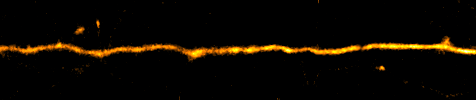

Supplement: Supplementary file 26 — Source data Fig. 4 [file 44318_2025_609_MOESM26_ESM.zip › EMBOJ-2024-119578_SourceDataForFigure4/4C/+High K with EDTA/+High K with EDTA 8min.tif]

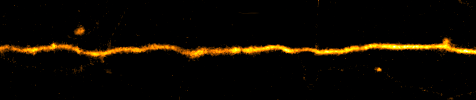

Supplement: Supplementary file 26 — Source data Fig. 4 [file 44318_2025_609_MOESM26_ESM.zip › EMBOJ-2024-119578_SourceDataForFigure4/4C/+High K with EDTA/Before.tif]

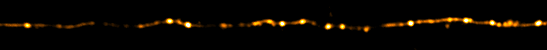

Supplement: Supplementary file 26 — Source data Fig. 4 [file 44318_2025_609_MOESM26_ESM.zip › EMBOJ-2024-119578_SourceDataForFigure4/4D/+High K/+High K 0min.tif]

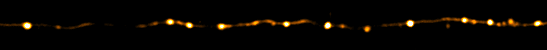

Supplement: Supplementary file 26 — Source data Fig. 4 [file 44318_2025_609_MOESM26_ESM.zip › EMBOJ-2024-119578_SourceDataForFigure4/4D/+High K/+High K 10min.tif]

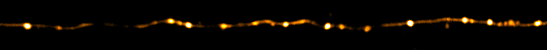

Supplement: Supplementary file 26 — Source data Fig. 4 [file 44318_2025_609_MOESM26_ESM.zip › EMBOJ-2024-119578_SourceDataForFigure4/4D/+High K/+High K 5min.tif]

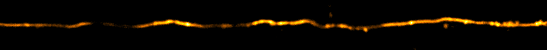

Supplement: Supplementary file 26 — Source data Fig. 4 [file 44318_2025_609_MOESM26_ESM.zip › EMBOJ-2024-119578_SourceDataForFigure4/4D/+High K/Before.tif]

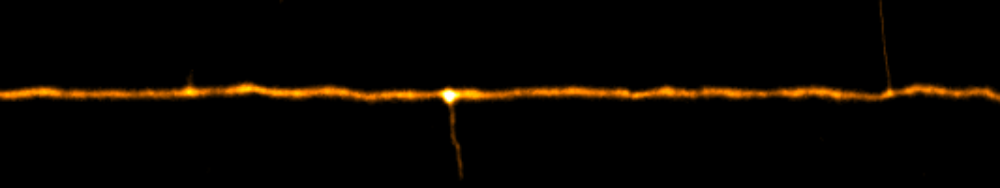

Supplement: Supplementary file 26 — Source data Fig. 4 [file 44318_2025_609_MOESM26_ESM.zip › EMBOJ-2024-119578_SourceDataForFigure4/4D/+High K with EDTA/+High K with EDTA 0min.tif]

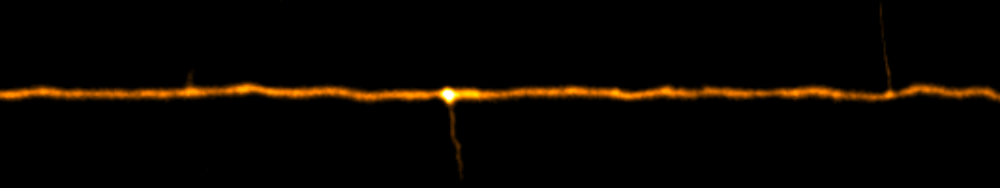

Supplement: Supplementary file 26 — Source data Fig. 4 [file 44318_2025_609_MOESM26_ESM.zip › EMBOJ-2024-119578_SourceDataForFigure4/4D/+High K with EDTA/+High K with EDTA 10min.tif]

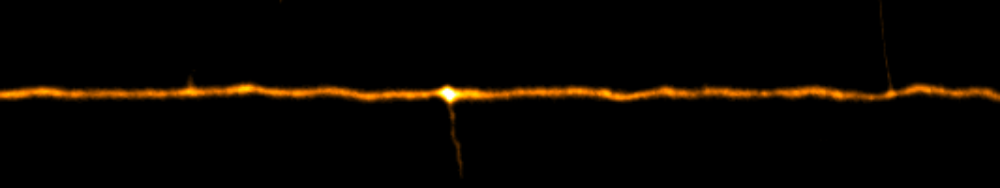

Supplement: Supplementary file 26 — Source data Fig. 4 [file 44318_2025_609_MOESM26_ESM.zip › EMBOJ-2024-119578_SourceDataForFigure4/4D/+High K with EDTA/+High K with EDTA 5min.tif]

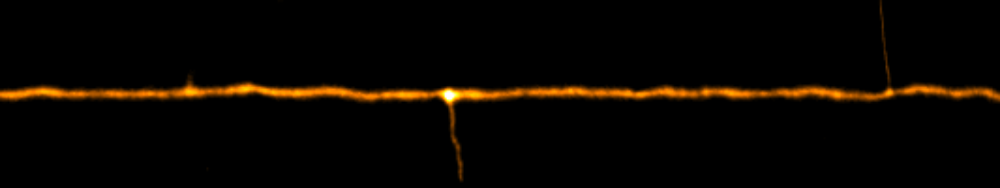

Supplement: Supplementary file 26 — Source data Fig. 4 [file 44318_2025_609_MOESM26_ESM.zip › EMBOJ-2024-119578_SourceDataForFigure4/4D/+High K with EDTA/Before.tif]

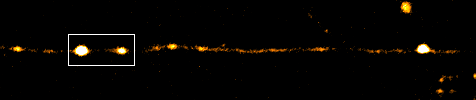

Supplement: Supplementary file 26 — Source data Fig. 4 [file 44318_2025_609_MOESM26_ESM.zip › EMBOJ-2024-119578_SourceDataForFigure4/4E/+High K 8min ANXA7-mCherry.tif]

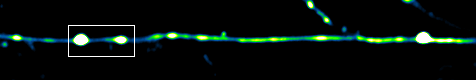

Supplement: Supplementary file 26 — Source data Fig. 4 [file 44318_2025_609_MOESM26_ESM.zip › EMBOJ-2024-119578_SourceDataForFigure4/4E/+High K 8min GCaMP6f.tif]

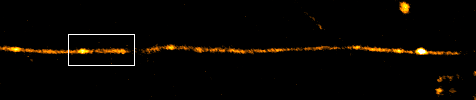

Supplement: Supplementary file 26 — Source data Fig. 4 [file 44318_2025_609_MOESM26_ESM.zip › EMBOJ-2024-119578_SourceDataForFigure4/4E/Before ANXA7-mCherry.tif]

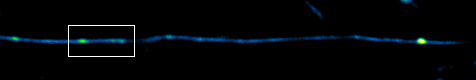

Supplement: Supplementary file 26 — Source data Fig. 4 [file 44318_2025_609_MOESM26_ESM.zip › EMBOJ-2024-119578_SourceDataForFigure4/4E/Before GCaMP6f.tif]

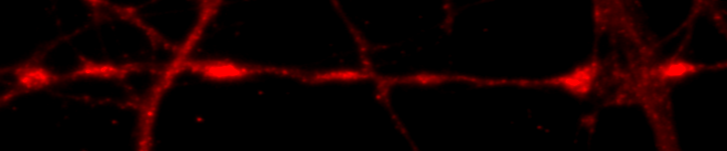

Supplement: Supplementary file 26 — Source data Fig. 4 [file 44318_2025_609_MOESM26_ESM.zip › EMBOJ-2024-119578_SourceDataForFigure4/4G/High K-ANXA7.tif]

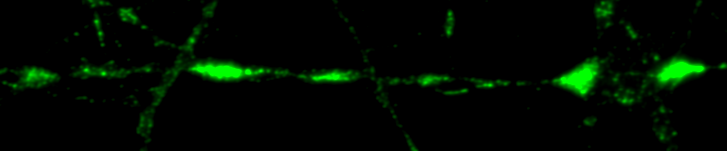

Supplement: Supplementary file 26 — Source data Fig. 4 [file 44318_2025_609_MOESM26_ESM.zip › EMBOJ-2024-119578_SourceDataForFigure4/4G/High K-TIA1.tif]

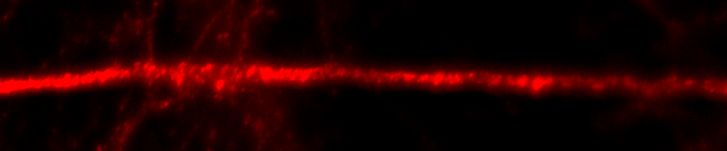

Supplement: Supplementary file 26 — Source data Fig. 4 [file 44318_2025_609_MOESM26_ESM.zip › EMBOJ-2024-119578_SourceDataForFigure4/4G/Low K-ANXA7.tif]

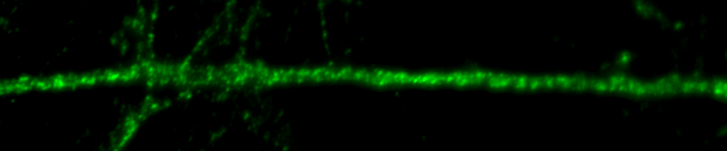

Supplement: Supplementary file 26 — Source data Fig. 4 [file 44318_2025_609_MOESM26_ESM.zip › EMBOJ-2024-119578_SourceDataForFigure4/4G/Low K-TIA1.tif]

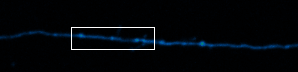

Supplement: Supplementary file 26 — Source data Fig. 4 [file 44318_2025_609_MOESM26_ESM.zip › EMBOJ-2024-119578_SourceDataForFigure4/4I/GCaMP6f.tif]

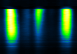

Supplement: Supplementary file 26 — Source data Fig. 4 [file 44318_2025_609_MOESM26_ESM.zip › EMBOJ-2024-119578_SourceDataForFigure4/4I/Kymograph.tif]

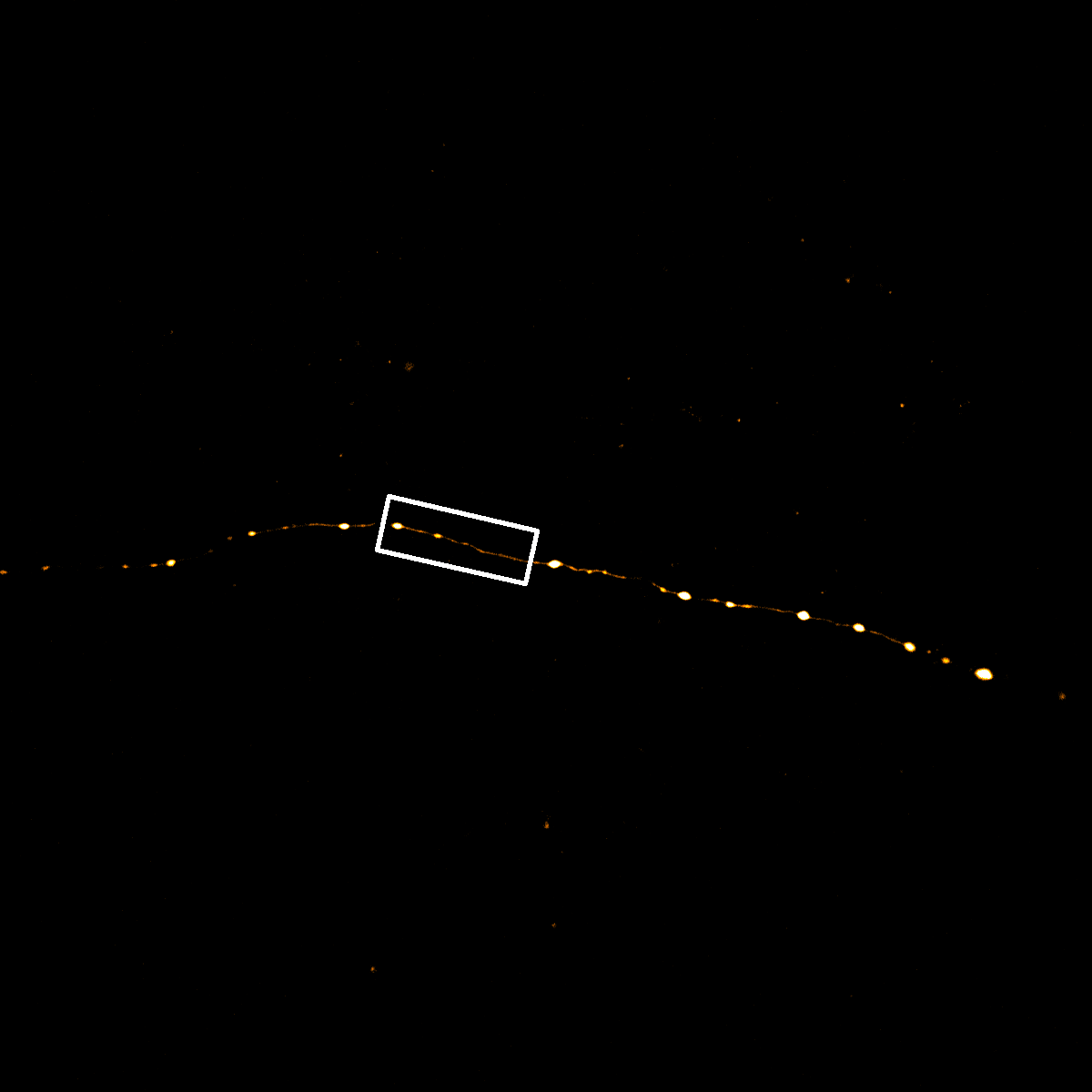

Supplement: Supplementary file 26 — Source data Fig. 4 [file 44318_2025_609_MOESM26_ESM.zip › EMBOJ-2024-119578_SourceDataForFigure4/4J/ANXA7 and GCaMP6f/ANXA7-After.tif]

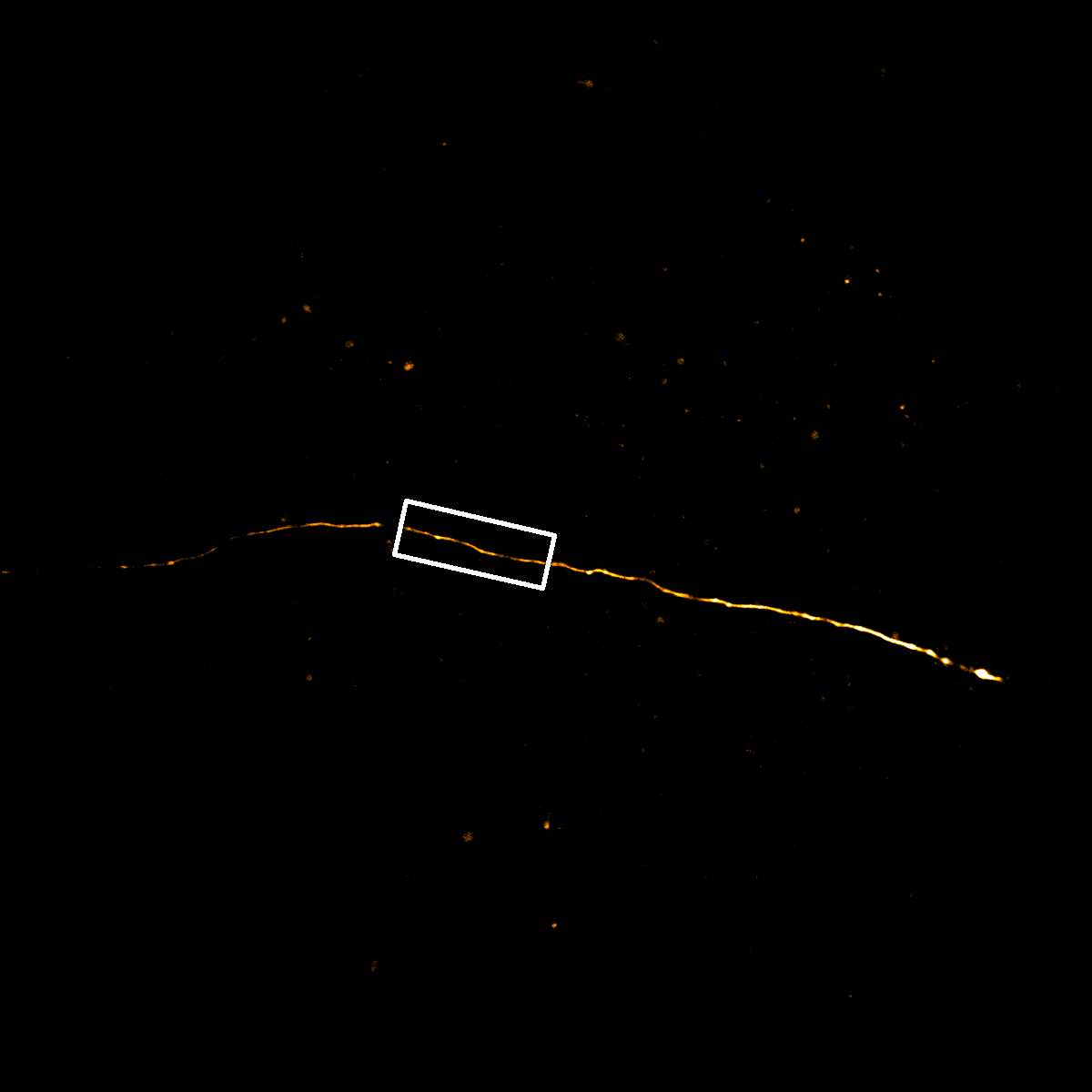

Supplement: Supplementary file 26 — Source data Fig. 4 [file 44318_2025_609_MOESM26_ESM.zip › EMBOJ-2024-119578_SourceDataForFigure4/4J/ANXA7 and GCaMP6f/ANXA7-Before.tif]

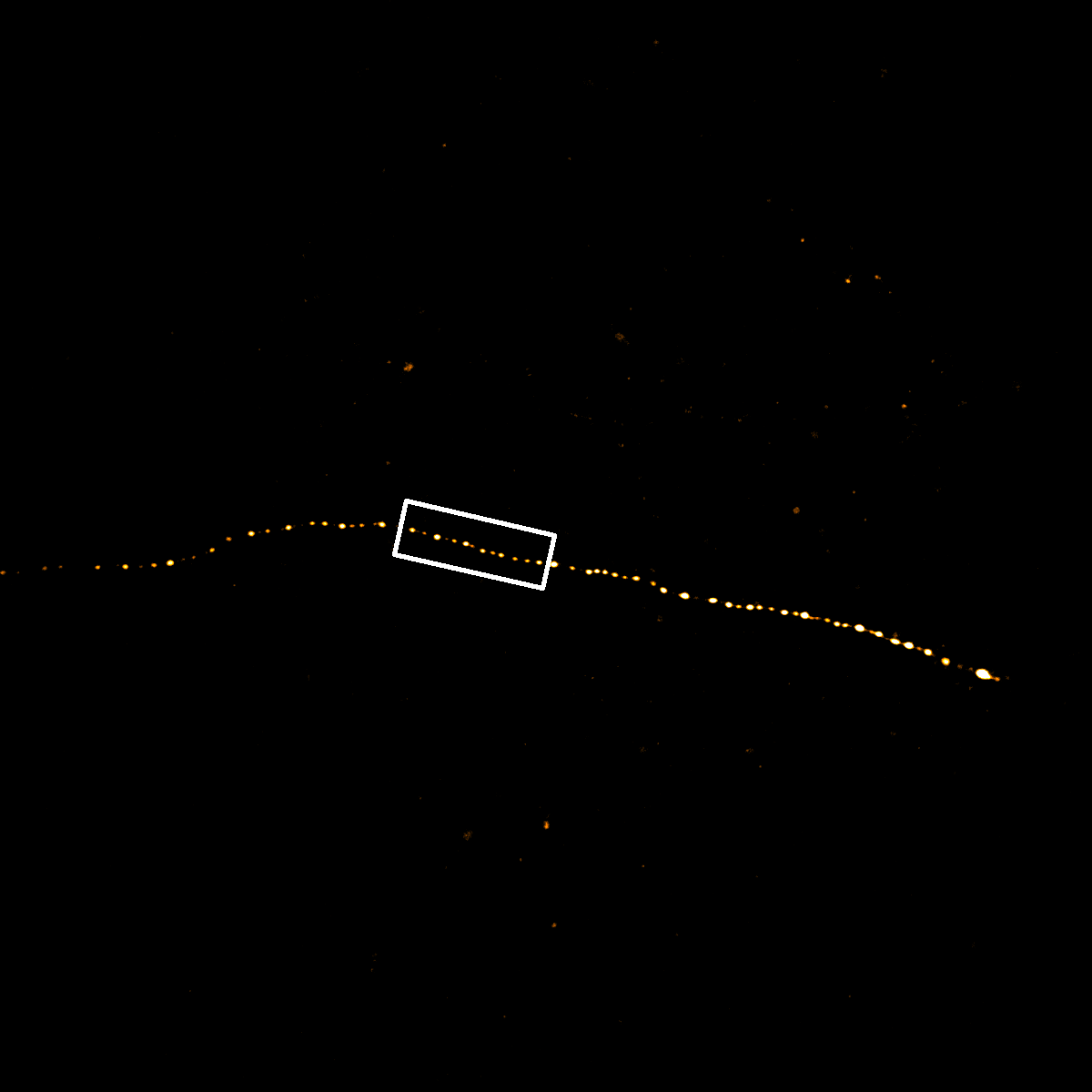

Supplement: Supplementary file 26 — Source data Fig. 4 [file 44318_2025_609_MOESM26_ESM.zip › EMBOJ-2024-119578_SourceDataForFigure4/4J/ANXA7 and GCaMP6f/ANXA7-Flux.tif]

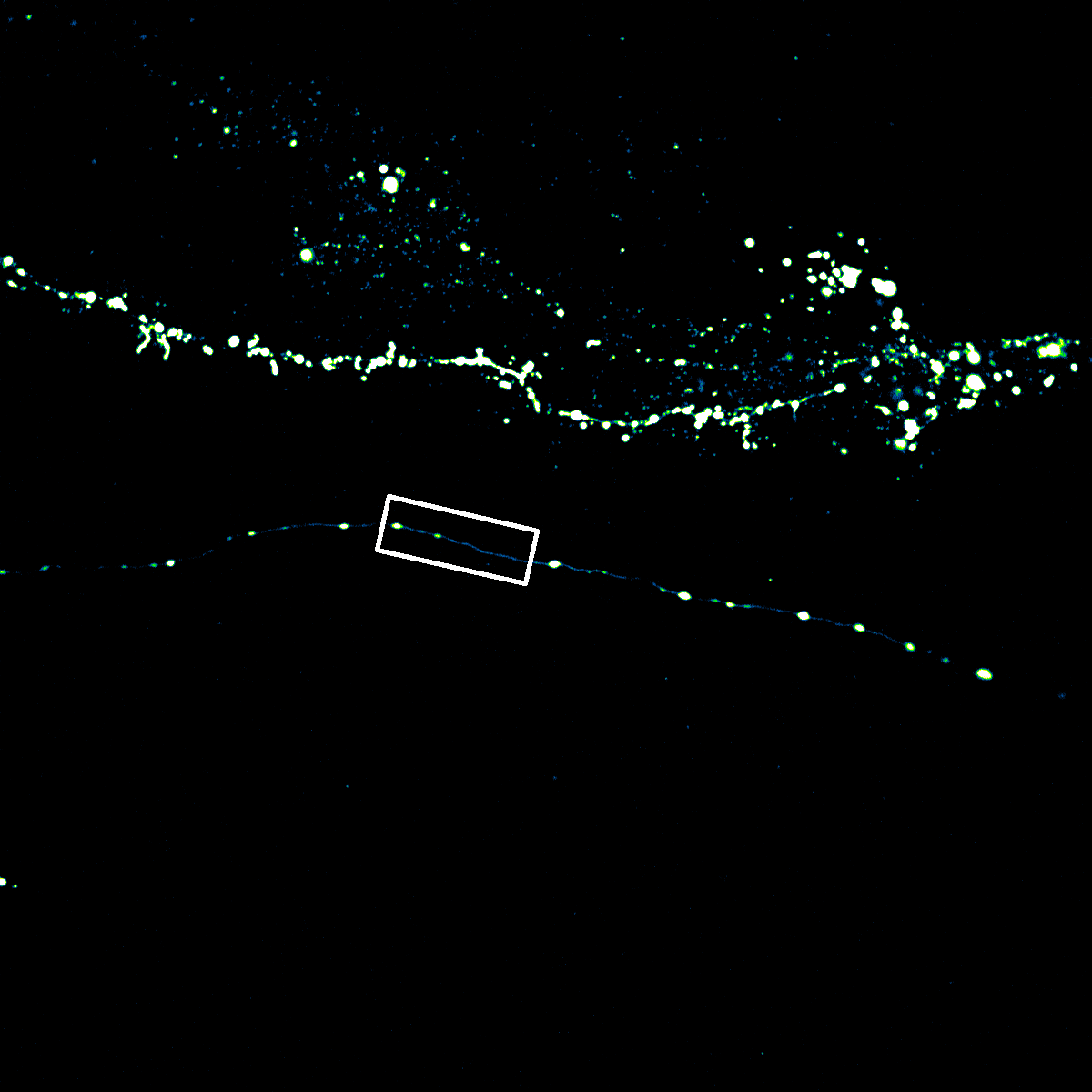

Supplement: Supplementary file 26 — Source data Fig. 4 [file 44318_2025_609_MOESM26_ESM.zip › EMBOJ-2024-119578_SourceDataForFigure4/4J/ANXA7 and GCaMP6f/GCaMP6f-After.tif]

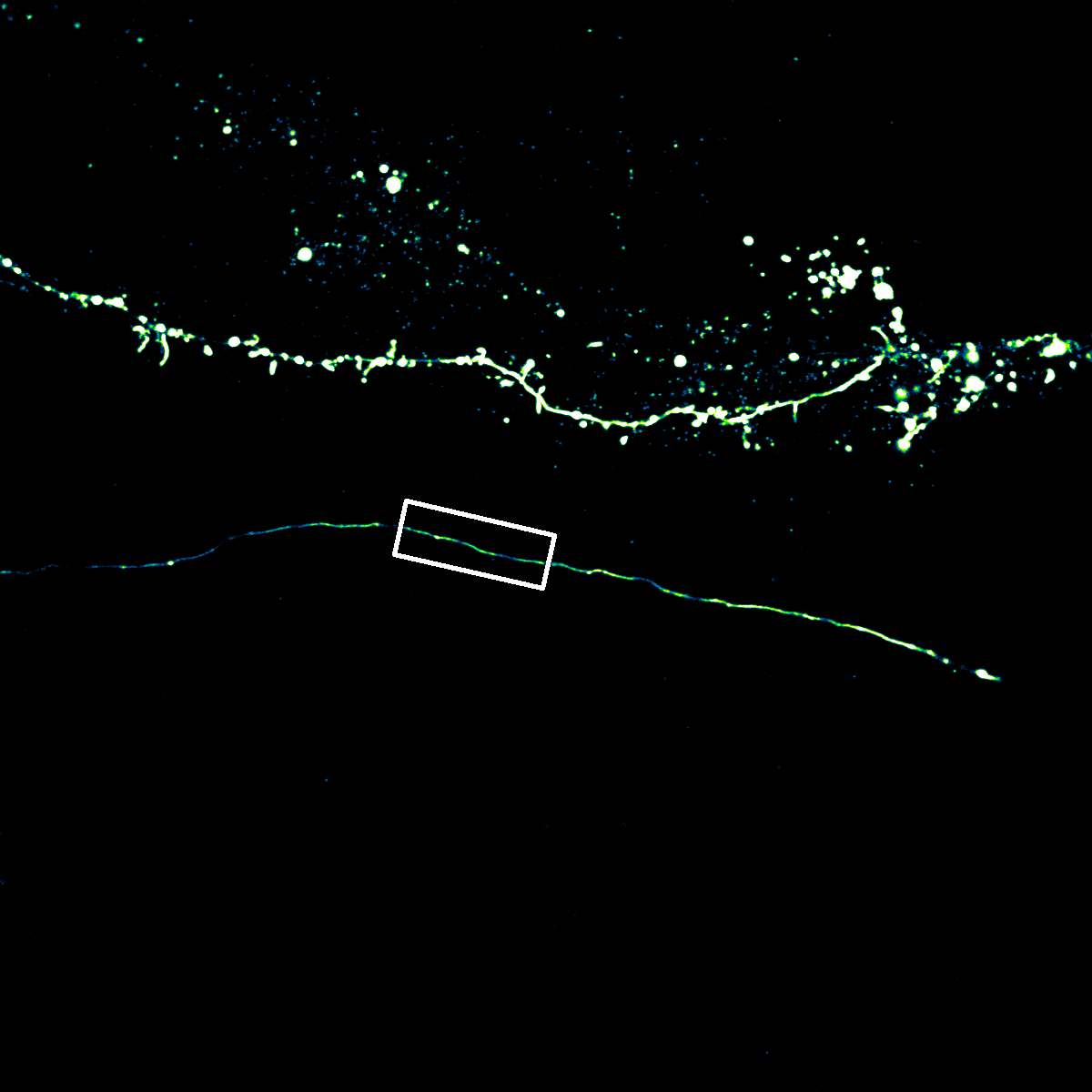

Supplement: Supplementary file 26 — Source data Fig. 4 [file 44318_2025_609_MOESM26_ESM.zip › EMBOJ-2024-119578_SourceDataForFigure4/4J/ANXA7 and GCaMP6f/GCaMP6f-Before.tif]

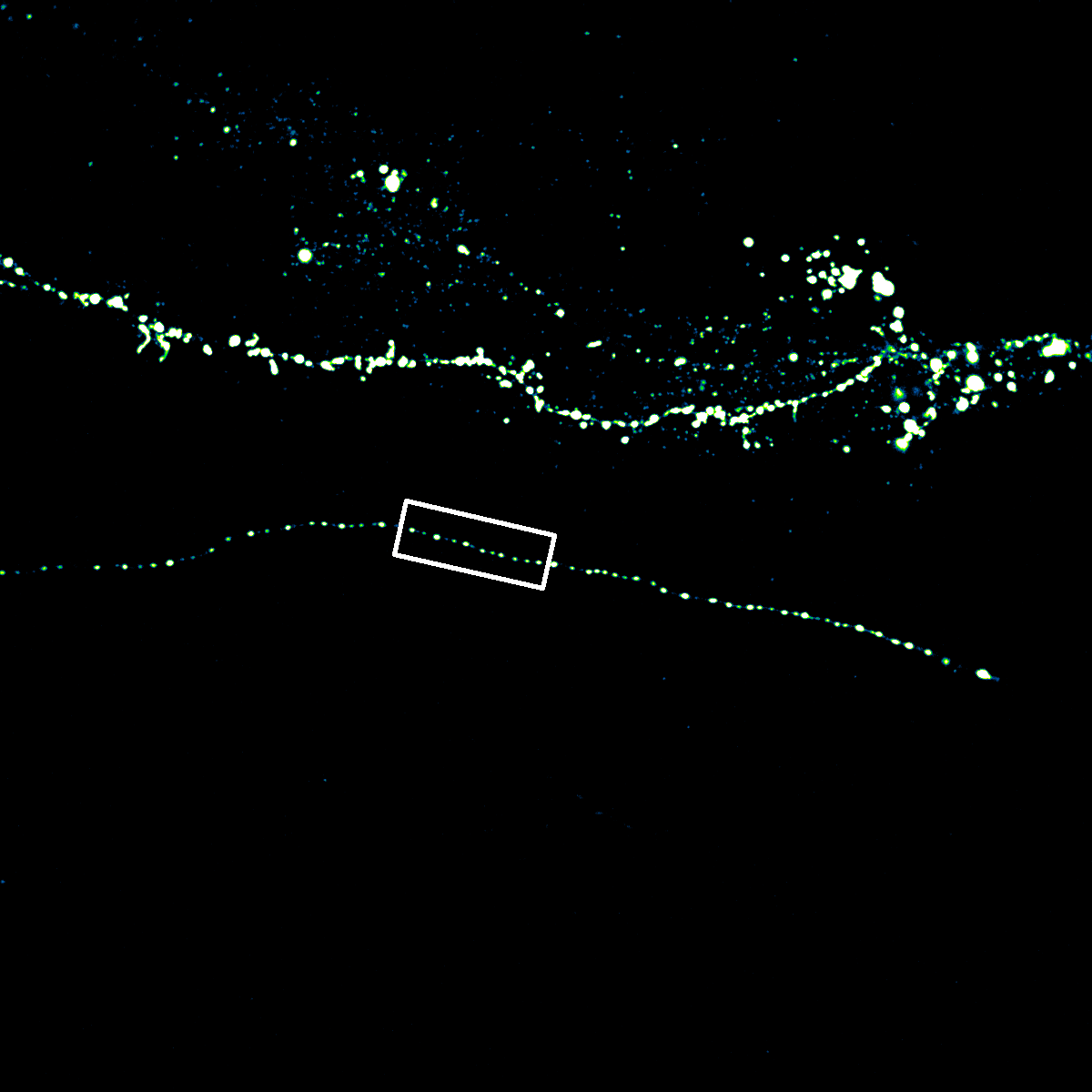

Supplement: Supplementary file 26 — Source data Fig. 4 [file 44318_2025_609_MOESM26_ESM.zip › EMBOJ-2024-119578_SourceDataForFigure4/4J/ANXA7 and GCaMP6f/GCaMP6f-Flux.tif]

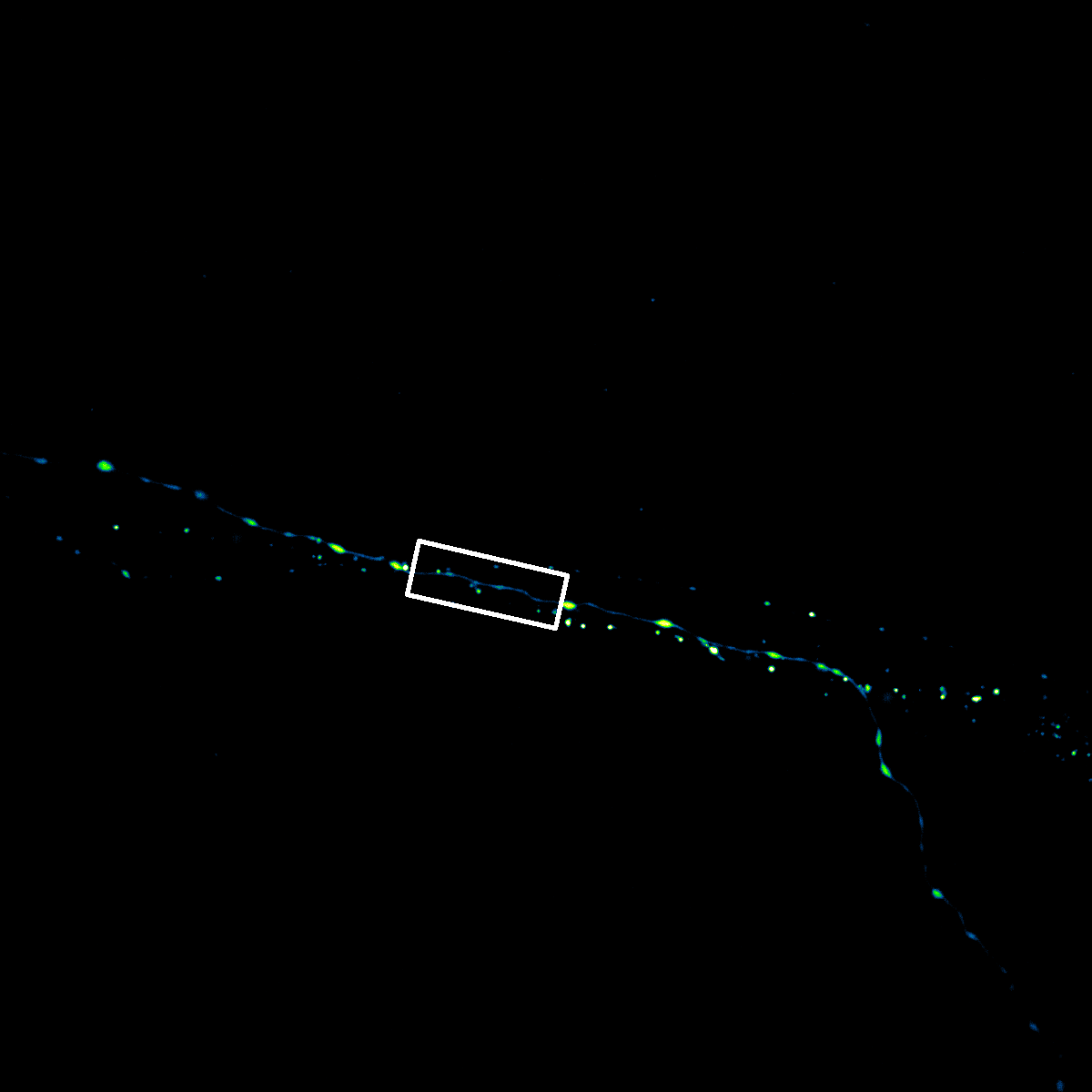

Supplement: Supplementary file 26 — Source data Fig. 4 [file 44318_2025_609_MOESM26_ESM.zip › EMBOJ-2024-119578_SourceDataForFigure4/4J/TIA1 and GCaMP6f/GCaMP6f-After.tif]

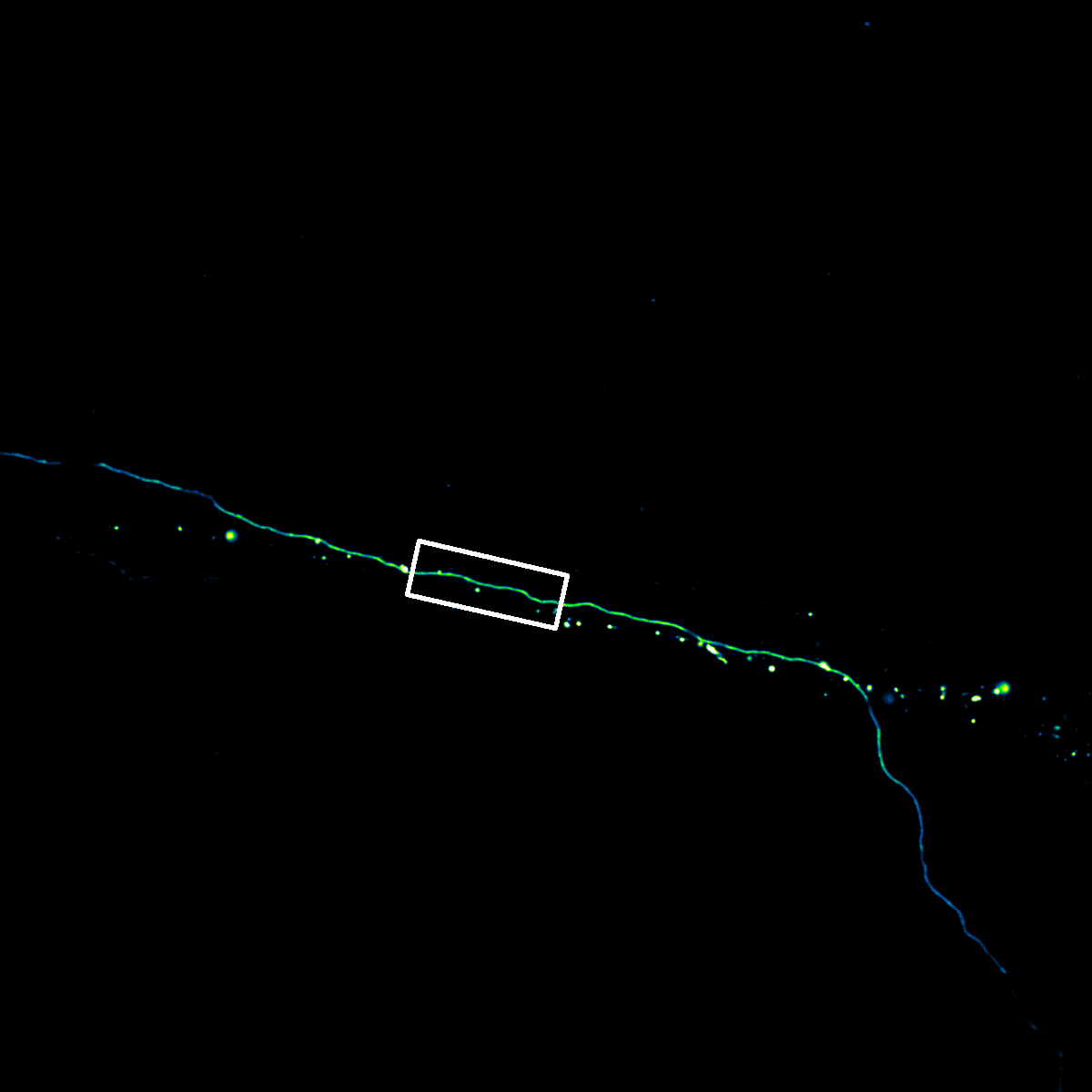

Supplement: Supplementary file 26 — Source data Fig. 4 [file 44318_2025_609_MOESM26_ESM.zip › EMBOJ-2024-119578_SourceDataForFigure4/4J/TIA1 and GCaMP6f/GCaMP6f-Before.tif]

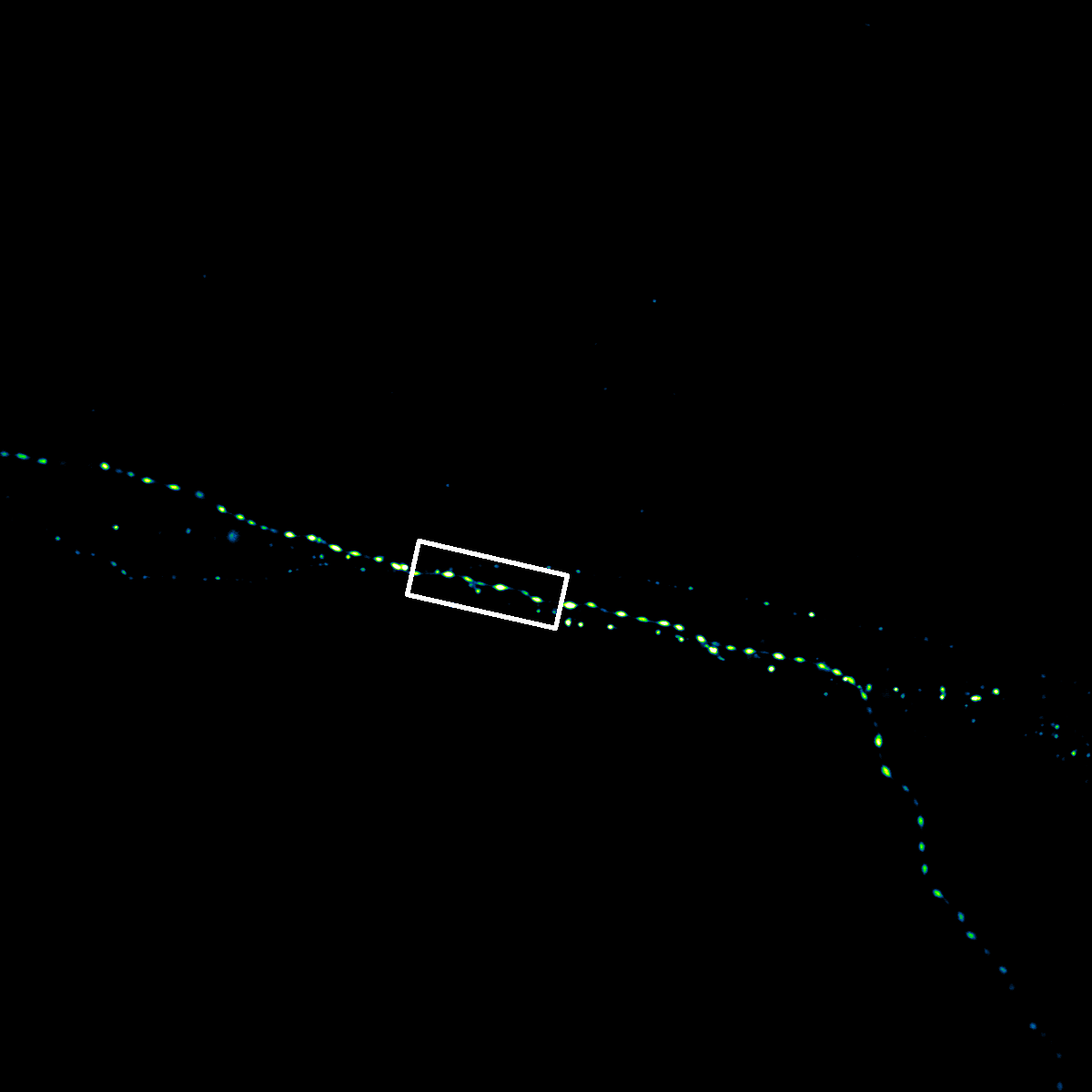

Supplement: Supplementary file 26 — Source data Fig. 4 [file 44318_2025_609_MOESM26_ESM.zip › EMBOJ-2024-119578_SourceDataForFigure4/4J/TIA1 and GCaMP6f/GCaMP6f-Flux.tif]

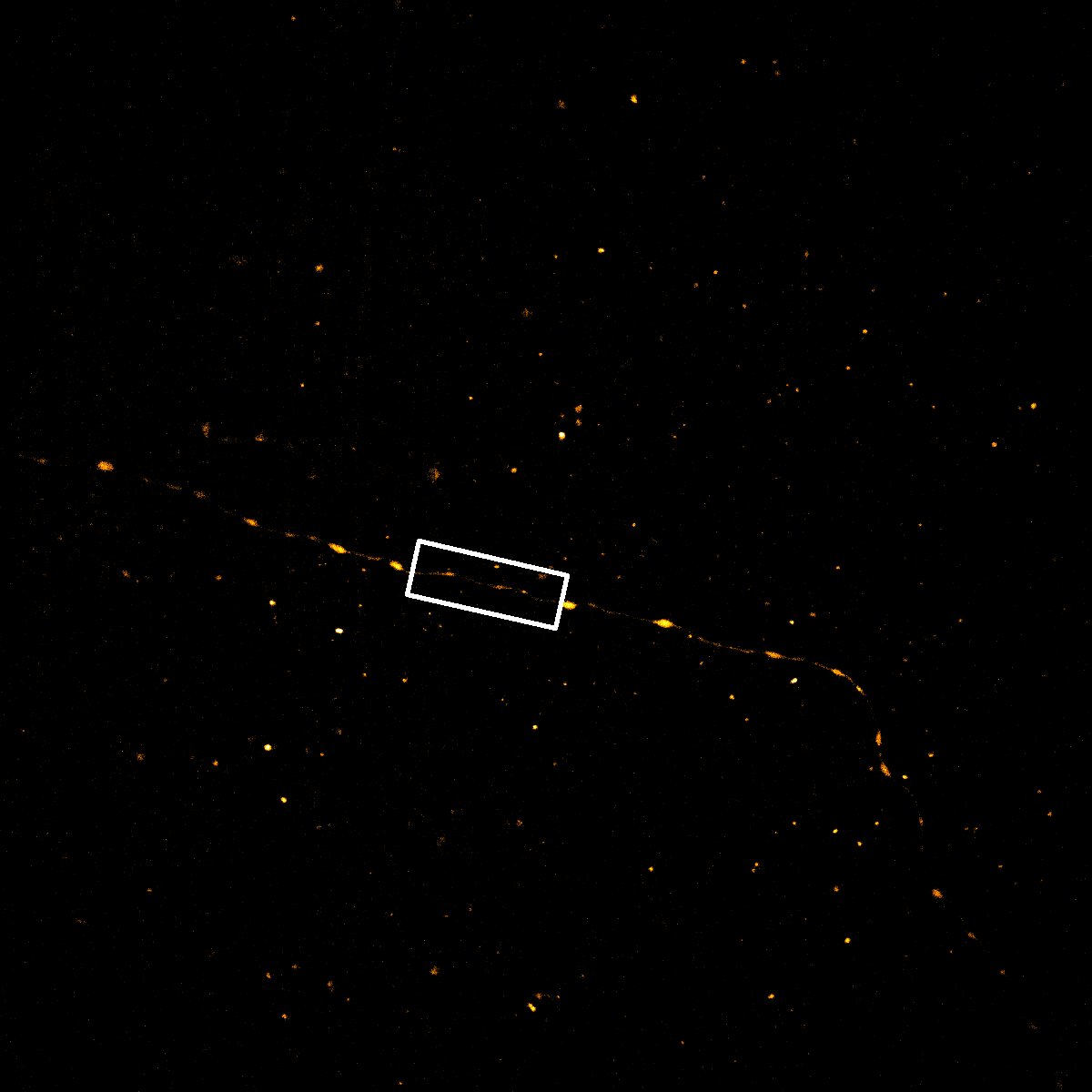

Supplement: Supplementary file 26 — Source data Fig. 4 [file 44318_2025_609_MOESM26_ESM.zip › EMBOJ-2024-119578_SourceDataForFigure4/4J/TIA1 and GCaMP6f/TIA1-After.tif]

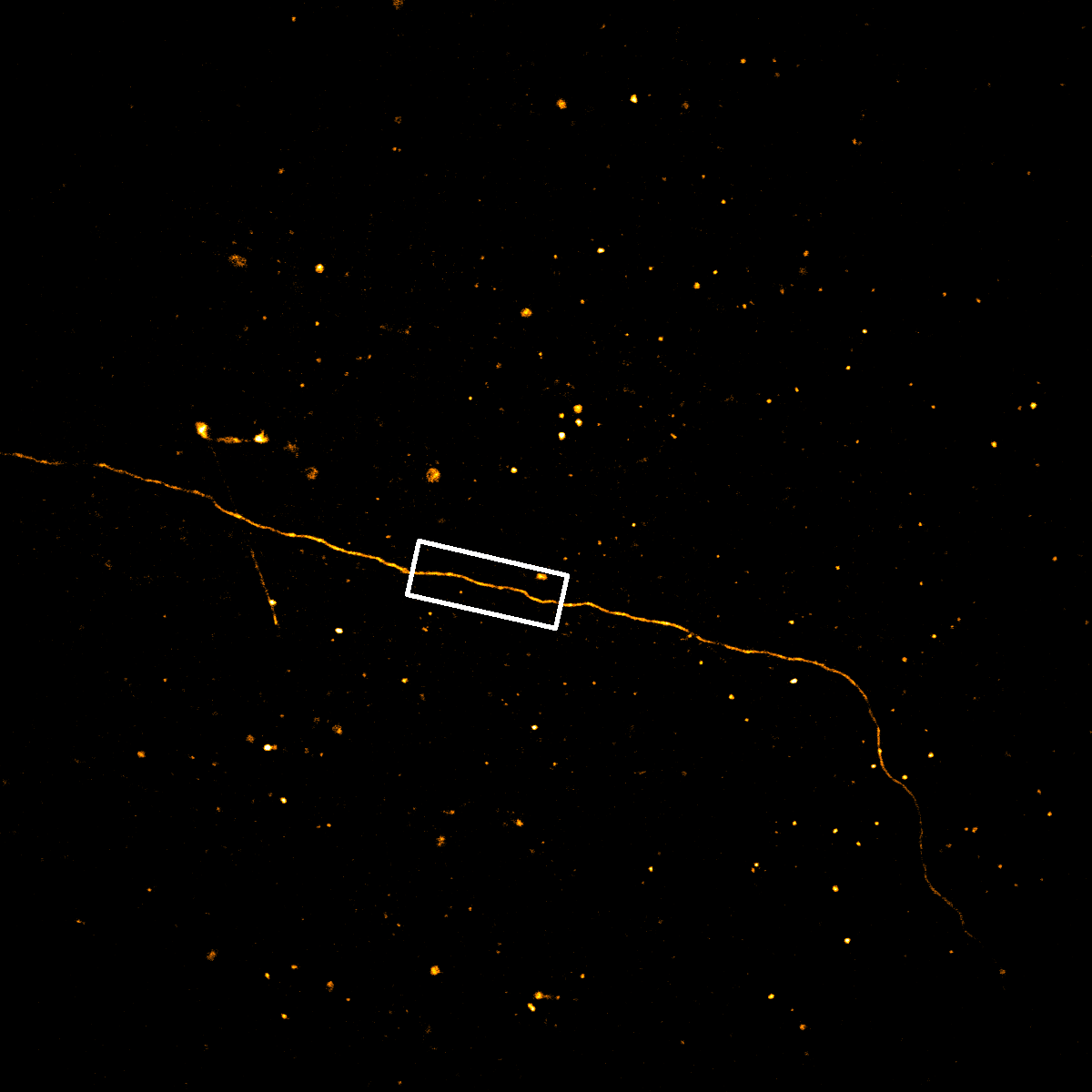

Supplement: Supplementary file 26 — Source data Fig. 4 [file 44318_2025_609_MOESM26_ESM.zip › EMBOJ-2024-119578_SourceDataForFigure4/4J/TIA1 and GCaMP6f/TIA1-Before.tif]

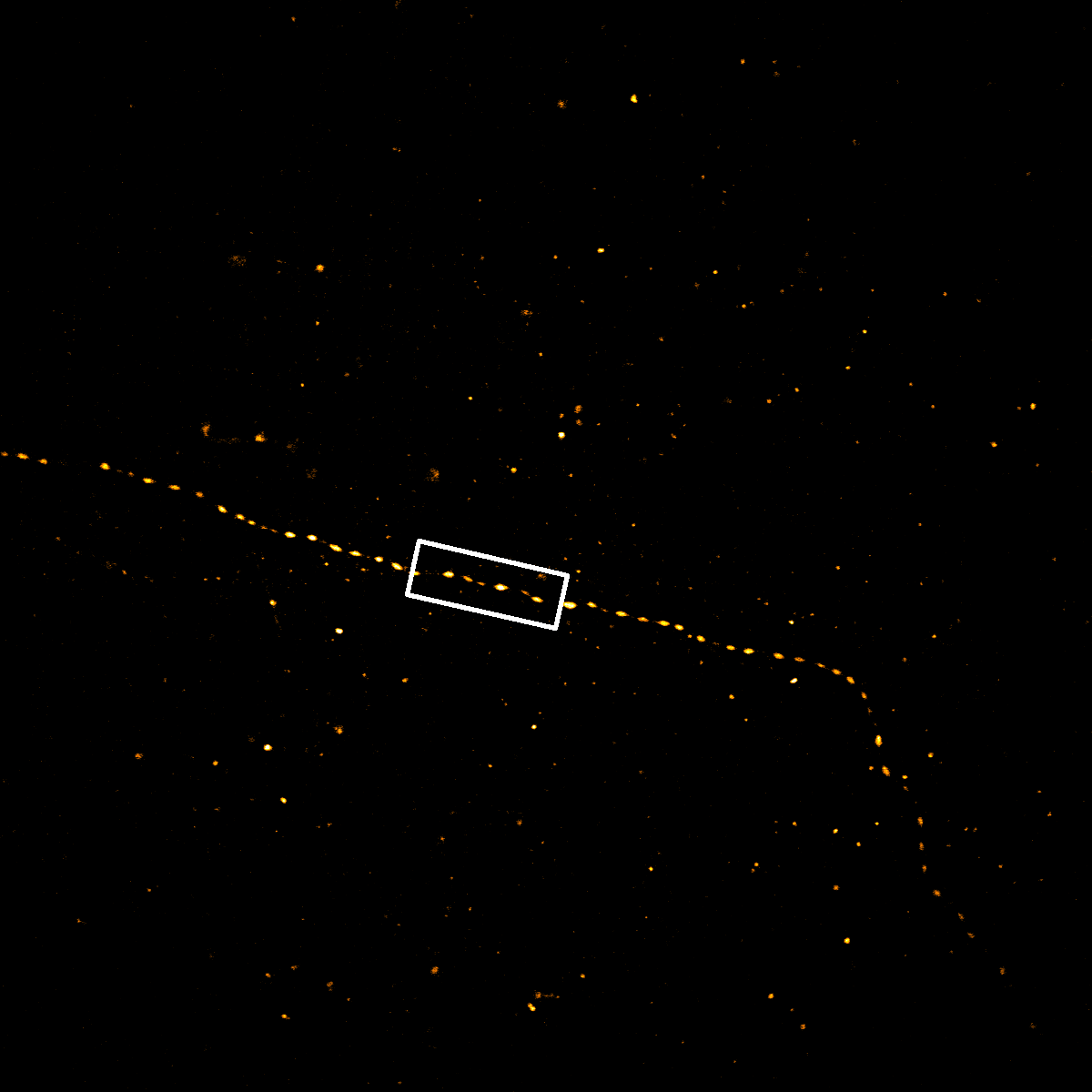

Supplement: Supplementary file 26 — Source data Fig. 4 [file 44318_2025_609_MOESM26_ESM.zip › EMBOJ-2024-119578_SourceDataForFigure4/4J/TIA1 and GCaMP6f/TIA1-Flux.tif]

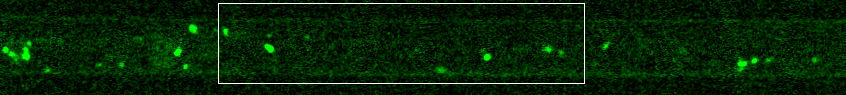

Supplement: Supplementary file 27 — Source data Fig. 5 [file 44318_2025_609_MOESM27_ESM.zip › EMBOJ-2024-119578_SourceDataForFigure5/5C/0s.tif]

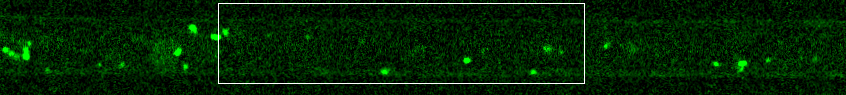

Supplement: Supplementary file 27 — Source data Fig. 5 [file 44318_2025_609_MOESM27_ESM.zip › EMBOJ-2024-119578_SourceDataForFigure5/5C/30s.tif]

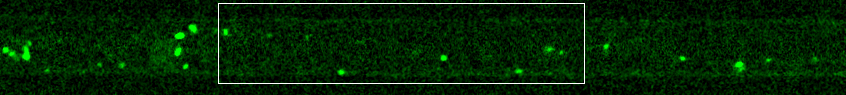

Supplement: Supplementary file 27 — Source data Fig. 5 [file 44318_2025_609_MOESM27_ESM.zip › EMBOJ-2024-119578_SourceDataForFigure5/5C/60s.tif]

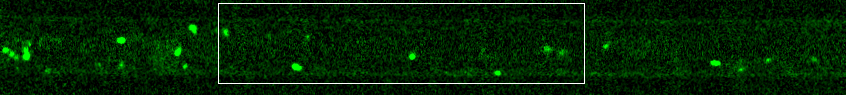

Supplement: Supplementary file 27 — Source data Fig. 5 [file 44318_2025_609_MOESM27_ESM.zip › EMBOJ-2024-119578_SourceDataForFigure5/5C/90s.tif]

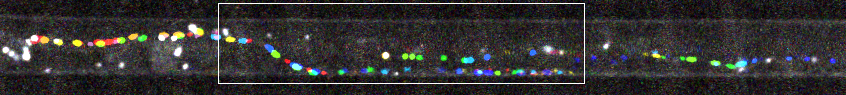

Supplement: Supplementary file 27 — Source data Fig. 5 [file 44318_2025_609_MOESM27_ESM.zip › EMBOJ-2024-119578_SourceDataForFigure5/5C/time-projection.tif]

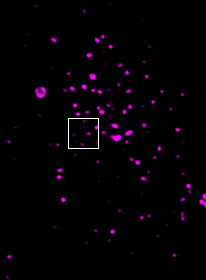

Supplement: Supplementary file 27 — Source data Fig. 5 [file 44318_2025_609_MOESM27_ESM.zip › EMBOJ-2024-119578_SourceDataForFigure5/5D/1_EEA1 only.tif]

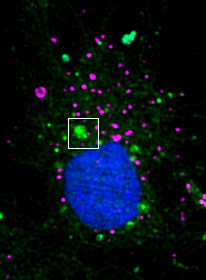

Supplement: Supplementary file 27 — Source data Fig. 5 [file 44318_2025_609_MOESM27_ESM.zip › EMBOJ-2024-119578_SourceDataForFigure5/5D/1_EEA1 with RNA and DAPI.tif]

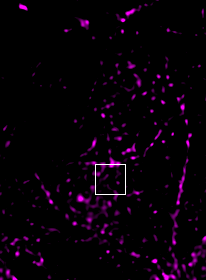

Supplement: Supplementary file 27 — Source data Fig. 5 [file 44318_2025_609_MOESM27_ESM.zip › EMBOJ-2024-119578_SourceDataForFigure5/5D/2_DCP1A only.tif]

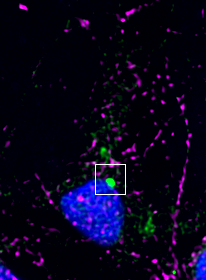

Supplement: Supplementary file 27 — Source data Fig. 5 [file 44318_2025_609_MOESM27_ESM.zip › EMBOJ-2024-119578_SourceDataForFigure5/5D/2_DCP1A with RNA and DAPI.tif]

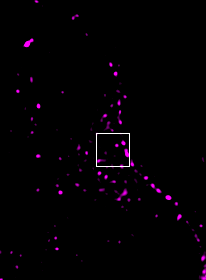

Supplement: Supplementary file 27 — Source data Fig. 5 [file 44318_2025_609_MOESM27_ESM.zip › EMBOJ-2024-119578_SourceDataForFigure5/5D/3_LC3 only.tif]

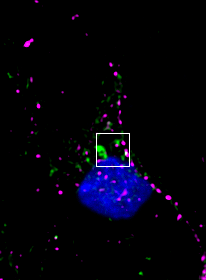

Supplement: Supplementary file 27 — Source data Fig. 5 [file 44318_2025_609_MOESM27_ESM.zip › EMBOJ-2024-119578_SourceDataForFigure5/5D/3_LC3 with RNA and DAPI.tif]

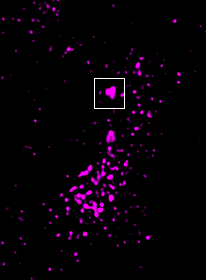

Supplement: Supplementary file 27 — Source data Fig. 5 [file 44318_2025_609_MOESM27_ESM.zip › EMBOJ-2024-119578_SourceDataForFigure5/5D/4_LAMP1 only.tif]

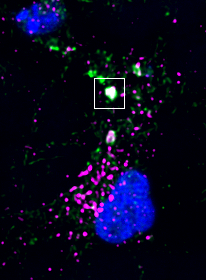

Supplement: Supplementary file 27 — Source data Fig. 5 [file 44318_2025_609_MOESM27_ESM.zip › EMBOJ-2024-119578_SourceDataForFigure5/5D/4_LAMP1 with RNA and DAPI.tif]

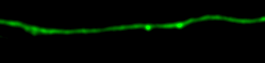

Supplement: Supplementary file 27 — Source data Fig. 5 [file 44318_2025_609_MOESM27_ESM.zip › EMBOJ-2024-119578_SourceDataForFigure5/5G/1_Hippocampal neurons_Control_GFP.tif]

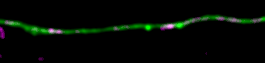

Supplement: Supplementary file 27 — Source data Fig. 5 [file 44318_2025_609_MOESM27_ESM.zip › EMBOJ-2024-119578_SourceDataForFigure5/5G/1_Hippocampal neurons_Control_Merge.tif]

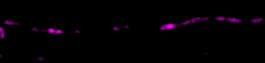

Supplement: Supplementary file 27 — Source data Fig. 5 [file 44318_2025_609_MOESM27_ESM.zip › EMBOJ-2024-119578_SourceDataForFigure5/5G/1_Hippocampal neurons_Control_Ryk mRNA smFISH.tif]

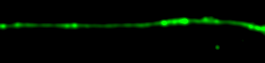

Supplement: Supplementary file 27 — Source data Fig. 5 [file 44318_2025_609_MOESM27_ESM.zip › EMBOJ-2024-119578_SourceDataForFigure5/5G/2_Hippocampal neurons_shANXA7_GFP.tif]

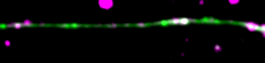

Supplement: Supplementary file 27 — Source data Fig. 5 [file 44318_2025_609_MOESM27_ESM.zip › EMBOJ-2024-119578_SourceDataForFigure5/5G/2_Hippocampal neurons_shANXA7_Merge.tif]

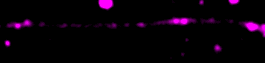

Supplement: Supplementary file 27 — Source data Fig. 5 [file 44318_2025_609_MOESM27_ESM.zip › EMBOJ-2024-119578_SourceDataForFigure5/5G/2_Hippocampal neurons_shANXA7_Ryk mRNA smFISH.tif]

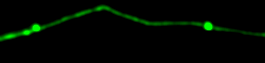

Supplement: Supplementary file 27 — Source data Fig. 5 [file 44318_2025_609_MOESM27_ESM.zip › EMBOJ-2024-119578_SourceDataForFigure5/5G/3_Hippocampal neurons_shANXA7 + ANXA7-res_GFP.tif]

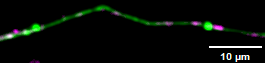

Supplement: Supplementary file 27 — Source data Fig. 5 [file 44318_2025_609_MOESM27_ESM.zip › EMBOJ-2024-119578_SourceDataForFigure5/5G/3_Hippocampal neurons_shANXA7 + ANXA7-res_Merge.tif]

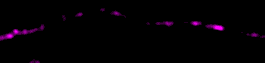

Supplement: Supplementary file 27 — Source data Fig. 5 [file 44318_2025_609_MOESM27_ESM.zip › EMBOJ-2024-119578_SourceDataForFigure5/5G/3_Hippocampal neurons_shANXA7 + ANXA7-res_Ryk mRNA smFISH.tif]

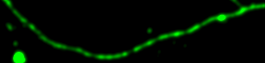

Supplement: Supplementary file 27 — Source data Fig. 5 [file 44318_2025_609_MOESM27_ESM.zip › EMBOJ-2024-119578_SourceDataForFigure5/5G/4_Cortical neurons_Control_GFP.tif]

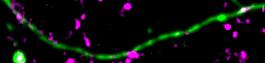

Supplement: Supplementary file 27 — Source data Fig. 5 [file 44318_2025_609_MOESM27_ESM.zip › EMBOJ-2024-119578_SourceDataForFigure5/5G/4_Cortical neurons_Control_Merge.tif]

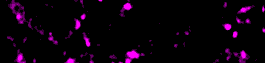

Supplement: Supplementary file 27 — Source data Fig. 5 [file 44318_2025_609_MOESM27_ESM.zip › EMBOJ-2024-119578_SourceDataForFigure5/5G/4_Cortical neurons_Control_Ryk mRNA smFISH.tif]

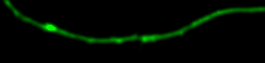

Supplement: Supplementary file 27 — Source data Fig. 5 [file 44318_2025_609_MOESM27_ESM.zip › EMBOJ-2024-119578_SourceDataForFigure5/5G/5_Cortical neurons_shANXA7_GFP.tif]

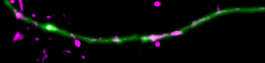

Supplement: Supplementary file 27 — Source data Fig. 5 [file 44318_2025_609_MOESM27_ESM.zip › EMBOJ-2024-119578_SourceDataForFigure5/5G/5_Cortical neurons_shANXA7_Merge.tif]

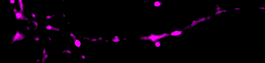

Supplement: Supplementary file 27 — Source data Fig. 5 [file 44318_2025_609_MOESM27_ESM.zip › EMBOJ-2024-119578_SourceDataForFigure5/5G/5_Cortical neurons_shANXA7_Ryk mRNA smFISH.tif]

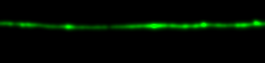

Supplement: Supplementary file 27 — Source data Fig. 5 [file 44318_2025_609_MOESM27_ESM.zip › EMBOJ-2024-119578_SourceDataForFigure5/5G/6_Cortical neurons_shANXA7 + ANXA7-res_GFP.tif]

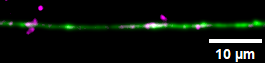

Supplement: Supplementary file 27 — Source data Fig. 5 [file 44318_2025_609_MOESM27_ESM.zip › EMBOJ-2024-119578_SourceDataForFigure5/5G/6_Cortical neurons_shANXA7 + ANXA7-res_Merge.tif]

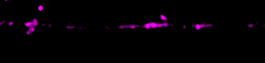

Supplement: Supplementary file 27 — Source data Fig. 5 [file 44318_2025_609_MOESM27_ESM.zip › EMBOJ-2024-119578_SourceDataForFigure5/5G/6_Cortical neurons_shANXA7 + ANXA7-res_Ryk mRNA smFISH.tif]

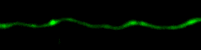

Supplement: Supplementary file 28 — Source data Fig. 6 [file 44318_2025_609_MOESM28_ESM.zip › EMBOJ-2024-119578_SourceDataForFigure6/6A/1_HN_Control_EGFP.tif]

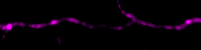

Supplement: Supplementary file 28 — Source data Fig. 6 [file 44318_2025_609_MOESM28_ESM.zip › EMBOJ-2024-119578_SourceDataForFigure6/6A/1_HN_Control_TIA1.tif]

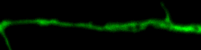

Supplement: Supplementary file 28 — Source data Fig. 6 [file 44318_2025_609_MOESM28_ESM.zip › EMBOJ-2024-119578_SourceDataForFigure6/6A/2_HN_A7 OE_EGFP.tif]

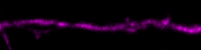

Supplement: Supplementary file 28 — Source data Fig. 6 [file 44318_2025_609_MOESM28_ESM.zip › EMBOJ-2024-119578_SourceDataForFigure6/6A/2_HN_A7 OE_TIA1.tif]

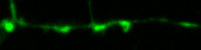

Supplement: Supplementary file 28 — Source data Fig. 6 [file 44318_2025_609_MOESM28_ESM.zip › EMBOJ-2024-119578_SourceDataForFigure6/6A/3_HN_shA7_EGFP.tif]

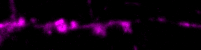

Supplement: Supplementary file 28 — Source data Fig. 6 [file 44318_2025_609_MOESM28_ESM.zip › EMBOJ-2024-119578_SourceDataForFigure6/6A/3_HN_shA7_TIA1.tif]

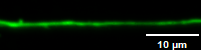

Supplement: Supplementary file 28 — Source data Fig. 6 [file 44318_2025_609_MOESM28_ESM.zip › EMBOJ-2024-119578_SourceDataForFigure6/6A/4_HN_A7-res_EGFP.tif]

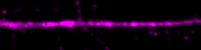

Supplement: Supplementary file 28 — Source data Fig. 6 [file 44318_2025_609_MOESM28_ESM.zip › EMBOJ-2024-119578_SourceDataForFigure6/6A/4_HN_A7-res_TIA1.tif]

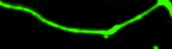

Supplement: Supplementary file 28 — Source data Fig. 6 [file 44318_2025_609_MOESM28_ESM.zip › EMBOJ-2024-119578_SourceDataForFigure6/6A/5_UMN_Control_EGFP.tif]

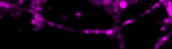

Supplement: Supplementary file 28 — Source data Fig. 6 [file 44318_2025_609_MOESM28_ESM.zip › EMBOJ-2024-119578_SourceDataForFigure6/6A/5_UMN_Control_TIA1.tif]

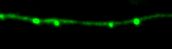

Supplement: Supplementary file 28 — Source data Fig. 6 [file 44318_2025_609_MOESM28_ESM.zip › EMBOJ-2024-119578_SourceDataForFigure6/6A/6_UMN_A7 OE_EGFP.tif]

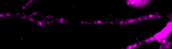

Supplement: Supplementary file 28 — Source data Fig. 6 [file 44318_2025_609_MOESM28_ESM.zip › EMBOJ-2024-119578_SourceDataForFigure6/6A/6_UMN_A7 OE_TIA1.tif]

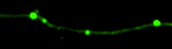

Supplement: Supplementary file 28 — Source data Fig. 6 [file 44318_2025_609_MOESM28_ESM.zip › EMBOJ-2024-119578_SourceDataForFigure6/6A/7_UMN_shA7_EGFP.tif]

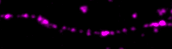

Supplement: Supplementary file 28 — Source data Fig. 6 [file 44318_2025_609_MOESM28_ESM.zip › EMBOJ-2024-119578_SourceDataForFigure6/6A/7_UMN_shA7_TIA1.tif]

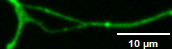

Supplement: Supplementary file 28 — Source data Fig. 6 [file 44318_2025_609_MOESM28_ESM.zip › EMBOJ-2024-119578_SourceDataForFigure6/6A/8_UMN_A7-res_EGFP.tif]

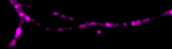

Supplement: Supplementary file 28 — Source data Fig. 6 [file 44318_2025_609_MOESM28_ESM.zip › EMBOJ-2024-119578_SourceDataForFigure6/6A/8_UMN_A7-res_TIA1.tif]

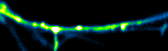

Supplement: Supplementary file 28 — Source data Fig. 6 [file 44318_2025_609_MOESM28_ESM.zip › EMBOJ-2024-119578_SourceDataForFigure6/6B/1_Control_before.tif]

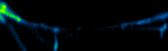

Supplement: Supplementary file 28 — Source data Fig. 6 [file 44318_2025_609_MOESM28_ESM.zip › EMBOJ-2024-119578_SourceDataForFigure6/6B/1_Control_FRAP_0''.tif]

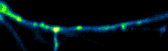

Supplement: Supplementary file 28 — Source data Fig. 6 [file 44318_2025_609_MOESM28_ESM.zip › EMBOJ-2024-119578_SourceDataForFigure6/6B/1_Control_FRAP_30''.tif]
