# Supplementary figures and images for: Annexin A7 enhances TIA1 axonal trafficking to counteract pathological aggregation in neurons (part 3 of 5)
Source: EMBO J. 2025 Nov 3;44(24):7477–512. doi: 10.1038/s44318-025-00609-8 (PMC12706091; doi:10.1038/s44318-025-00609-8)

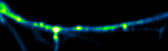

Supplement: Supplementary file 28 — Source data Fig. 6 [file 44318_2025_609_MOESM28_ESM.zip › EMBOJ-2024-119578_SourceDataForFigure6/6B/1_Control_FRAP_90''.tif]

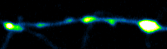

Supplement: Supplementary file 28 — Source data Fig. 6 [file 44318_2025_609_MOESM28_ESM.zip › EMBOJ-2024-119578_SourceDataForFigure6/6B/2_siANXA7_before.tif]

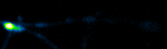

Supplement: Supplementary file 28 — Source data Fig. 6 [file 44318_2025_609_MOESM28_ESM.zip › EMBOJ-2024-119578_SourceDataForFigure6/6B/2_siANXA7_FRAP_0''.tif]

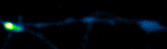

Supplement: Supplementary file 28 — Source data Fig. 6 [file 44318_2025_609_MOESM28_ESM.zip › EMBOJ-2024-119578_SourceDataForFigure6/6B/2_siANXA7_FRAP_30''.tif]

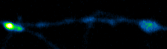

Supplement: Supplementary file 28 — Source data Fig. 6 [file 44318_2025_609_MOESM28_ESM.zip › EMBOJ-2024-119578_SourceDataForFigure6/6B/2_siANXA7_FRAP_90''.tif]

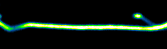

Supplement: Supplementary file 28 — Source data Fig. 6 [file 44318_2025_609_MOESM28_ESM.zip › EMBOJ-2024-119578_SourceDataForFigure6/6B/3_ANXA7 OE_before.tif]

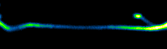

Supplement: Supplementary file 28 — Source data Fig. 6 [file 44318_2025_609_MOESM28_ESM.zip › EMBOJ-2024-119578_SourceDataForFigure6/6B/3_ANXA7 OE_FRAP_0''.tif]

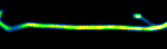

Supplement: Supplementary file 28 — Source data Fig. 6 [file 44318_2025_609_MOESM28_ESM.zip › EMBOJ-2024-119578_SourceDataForFigure6/6B/3_ANXA7 OE_FRAP_30''.tif]

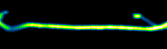

Supplement: Supplementary file 28 — Source data Fig. 6 [file 44318_2025_609_MOESM28_ESM.zip › EMBOJ-2024-119578_SourceDataForFigure6/6B/3_ANXA7 OE_FRAP_90''.tif]

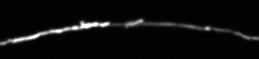

Supplement: Supplementary file 28 — Source data Fig. 6 [file 44318_2025_609_MOESM28_ESM.zip › EMBOJ-2024-119578_SourceDataForFigure6/6C/1_Control_0s.tif]

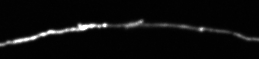

Supplement: Supplementary file 28 — Source data Fig. 6 [file 44318_2025_609_MOESM28_ESM.zip › EMBOJ-2024-119578_SourceDataForFigure6/6C/1_Control_20s.tif]

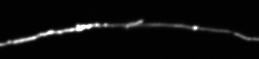

Supplement: Supplementary file 28 — Source data Fig. 6 [file 44318_2025_609_MOESM28_ESM.zip › EMBOJ-2024-119578_SourceDataForFigure6/6C/1_Control_40s.tif]

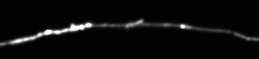

Supplement: Supplementary file 28 — Source data Fig. 6 [file 44318_2025_609_MOESM28_ESM.zip › EMBOJ-2024-119578_SourceDataForFigure6/6C/1_Control_80s.tif]

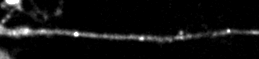

Supplement: Supplementary file 28 — Source data Fig. 6 [file 44318_2025_609_MOESM28_ESM.zip › EMBOJ-2024-119578_SourceDataForFigure6/6C/2_ANXA7 OE_0s.tif]

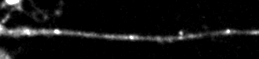

Supplement: Supplementary file 28 — Source data Fig. 6 [file 44318_2025_609_MOESM28_ESM.zip › EMBOJ-2024-119578_SourceDataForFigure6/6C/2_ANXA7 OE_20s.tif]

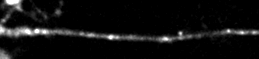

Supplement: Supplementary file 28 — Source data Fig. 6 [file 44318_2025_609_MOESM28_ESM.zip › EMBOJ-2024-119578_SourceDataForFigure6/6C/2_ANXA7 OE_40s.tif]

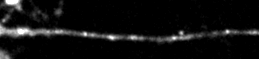

Supplement: Supplementary file 28 — Source data Fig. 6 [file 44318_2025_609_MOESM28_ESM.zip › EMBOJ-2024-119578_SourceDataForFigure6/6C/2_ANXA7 OE_80s.tif]

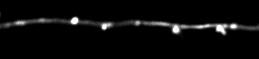

Supplement: Supplementary file 28 — Source data Fig. 6 [file 44318_2025_609_MOESM28_ESM.zip › EMBOJ-2024-119578_SourceDataForFigure6/6C/3_shANXA7_0s.tif]

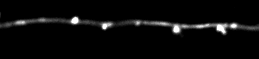

Supplement: Supplementary file 28 — Source data Fig. 6 [file 44318_2025_609_MOESM28_ESM.zip › EMBOJ-2024-119578_SourceDataForFigure6/6C/3_shANXA7_20s.tif]

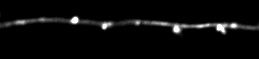

Supplement: Supplementary file 28 — Source data Fig. 6 [file 44318_2025_609_MOESM28_ESM.zip › EMBOJ-2024-119578_SourceDataForFigure6/6C/3_shANXA7_40s.tif]

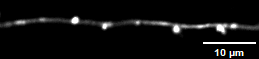

Supplement: Supplementary file 28 — Source data Fig. 6 [file 44318_2025_609_MOESM28_ESM.zip › EMBOJ-2024-119578_SourceDataForFigure6/6C/3_shANXA7_80s.tif]

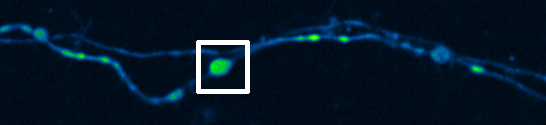

Supplement: Supplementary file 28 — Source data Fig. 6 [file 44318_2025_609_MOESM28_ESM.zip › EMBOJ-2024-119578_SourceDataForFigure6/6D/siANXA7_0''.tif]

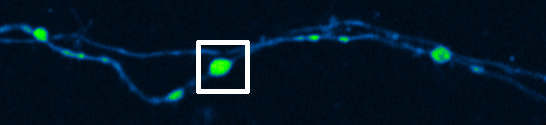

Supplement: Supplementary file 28 — Source data Fig. 6 [file 44318_2025_609_MOESM28_ESM.zip › EMBOJ-2024-119578_SourceDataForFigure6/6D/siANXA7_10''.tif]

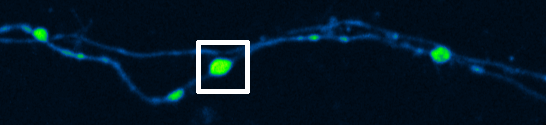

Supplement: Supplementary file 28 — Source data Fig. 6 [file 44318_2025_609_MOESM28_ESM.zip › EMBOJ-2024-119578_SourceDataForFigure6/6D/siANXA7_30''.tif]

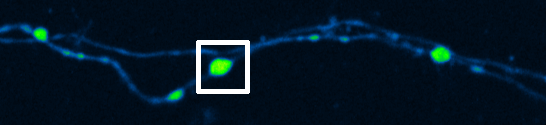

Supplement: Supplementary file 28 — Source data Fig. 6 [file 44318_2025_609_MOESM28_ESM.zip › EMBOJ-2024-119578_SourceDataForFigure6/6D/siANXA7_60''.tif]

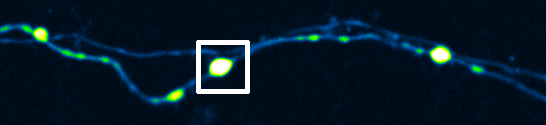

Supplement: Supplementary file 28 — Source data Fig. 6 [file 44318_2025_609_MOESM28_ESM.zip › EMBOJ-2024-119578_SourceDataForFigure6/6D/siANXA7_before.tif]

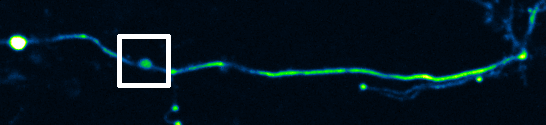

Supplement: Supplementary file 28 — Source data Fig. 6 [file 44318_2025_609_MOESM28_ESM.zip › EMBOJ-2024-119578_SourceDataForFigure6/6D/siControl_0''.tif]

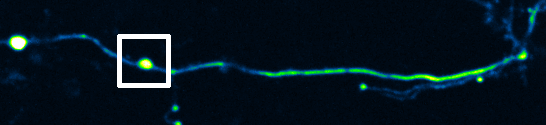

Supplement: Supplementary file 28 — Source data Fig. 6 [file 44318_2025_609_MOESM28_ESM.zip › EMBOJ-2024-119578_SourceDataForFigure6/6D/siControl_10''.tif]

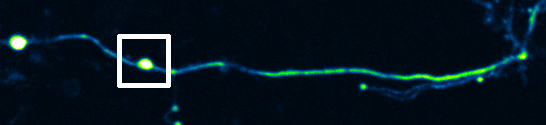

Supplement: Supplementary file 28 — Source data Fig. 6 [file 44318_2025_609_MOESM28_ESM.zip › EMBOJ-2024-119578_SourceDataForFigure6/6D/siControl_30''.tif]

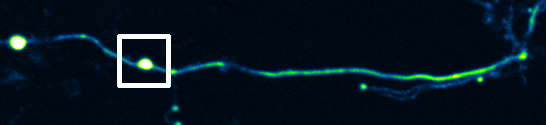

Supplement: Supplementary file 28 — Source data Fig. 6 [file 44318_2025_609_MOESM28_ESM.zip › EMBOJ-2024-119578_SourceDataForFigure6/6D/siControl_60''.tif]

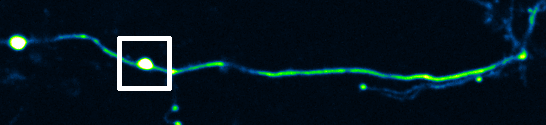

Supplement: Supplementary file 28 — Source data Fig. 6 [file 44318_2025_609_MOESM28_ESM.zip › EMBOJ-2024-119578_SourceDataForFigure6/6D/siControl_before.tif]

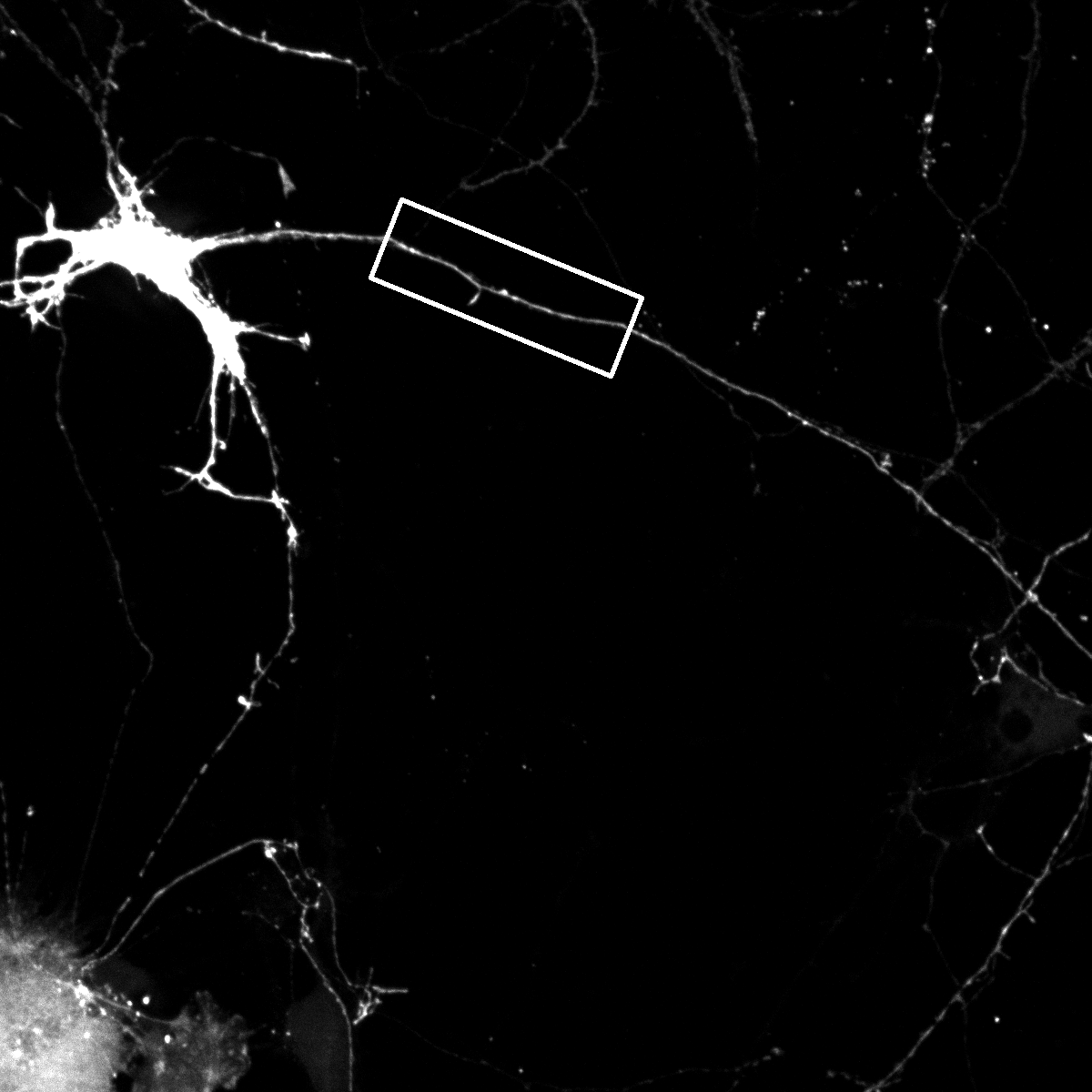

Supplement: Supplementary file 28 — Source data Fig. 6 [file 44318_2025_609_MOESM28_ESM.zip › EMBOJ-2024-119578_SourceDataForFigure6/6E/Control-EGFP.tif]

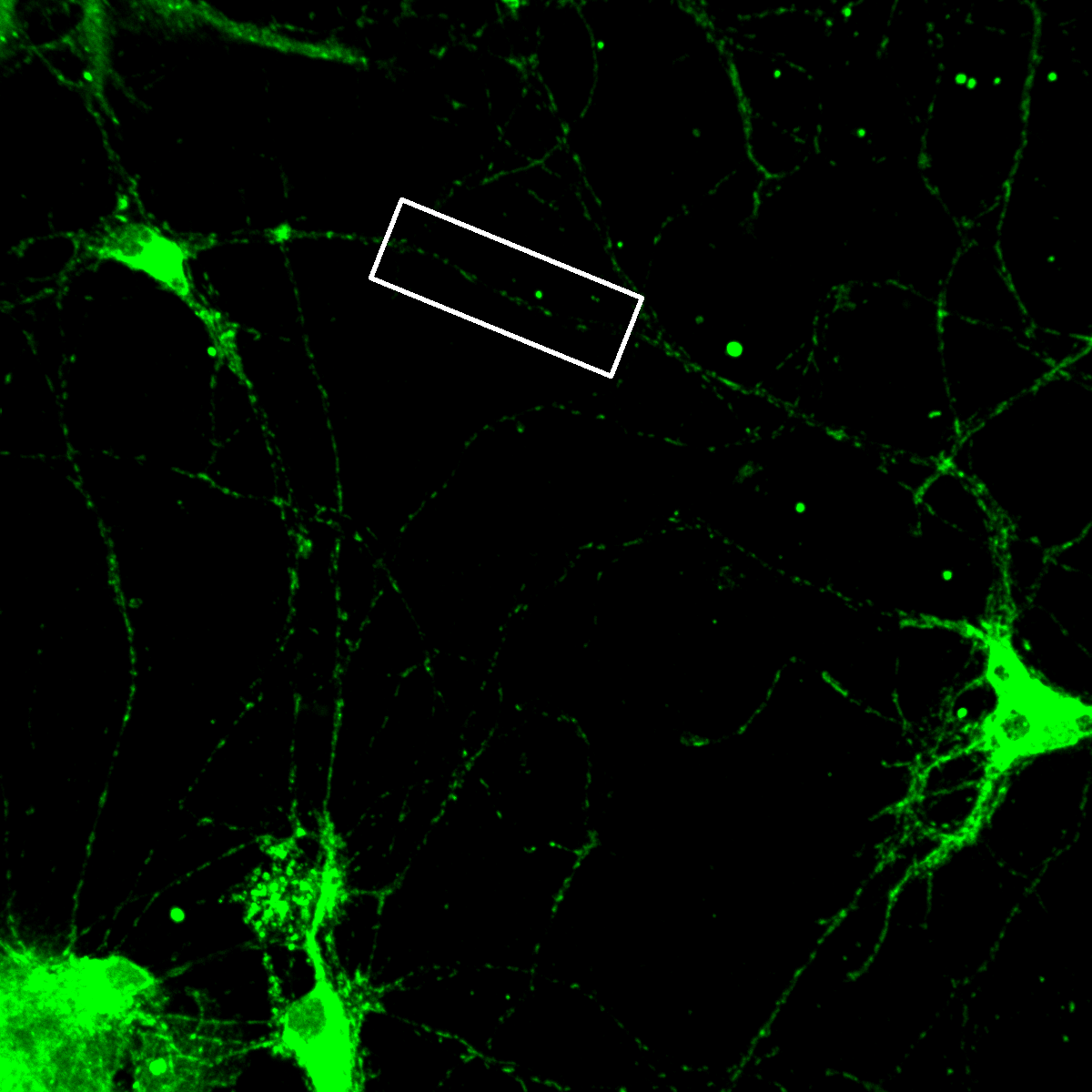

Supplement: Supplementary file 28 — Source data Fig. 6 [file 44318_2025_609_MOESM28_ESM.zip › EMBOJ-2024-119578_SourceDataForFigure6/6E/Control-p62.tif]

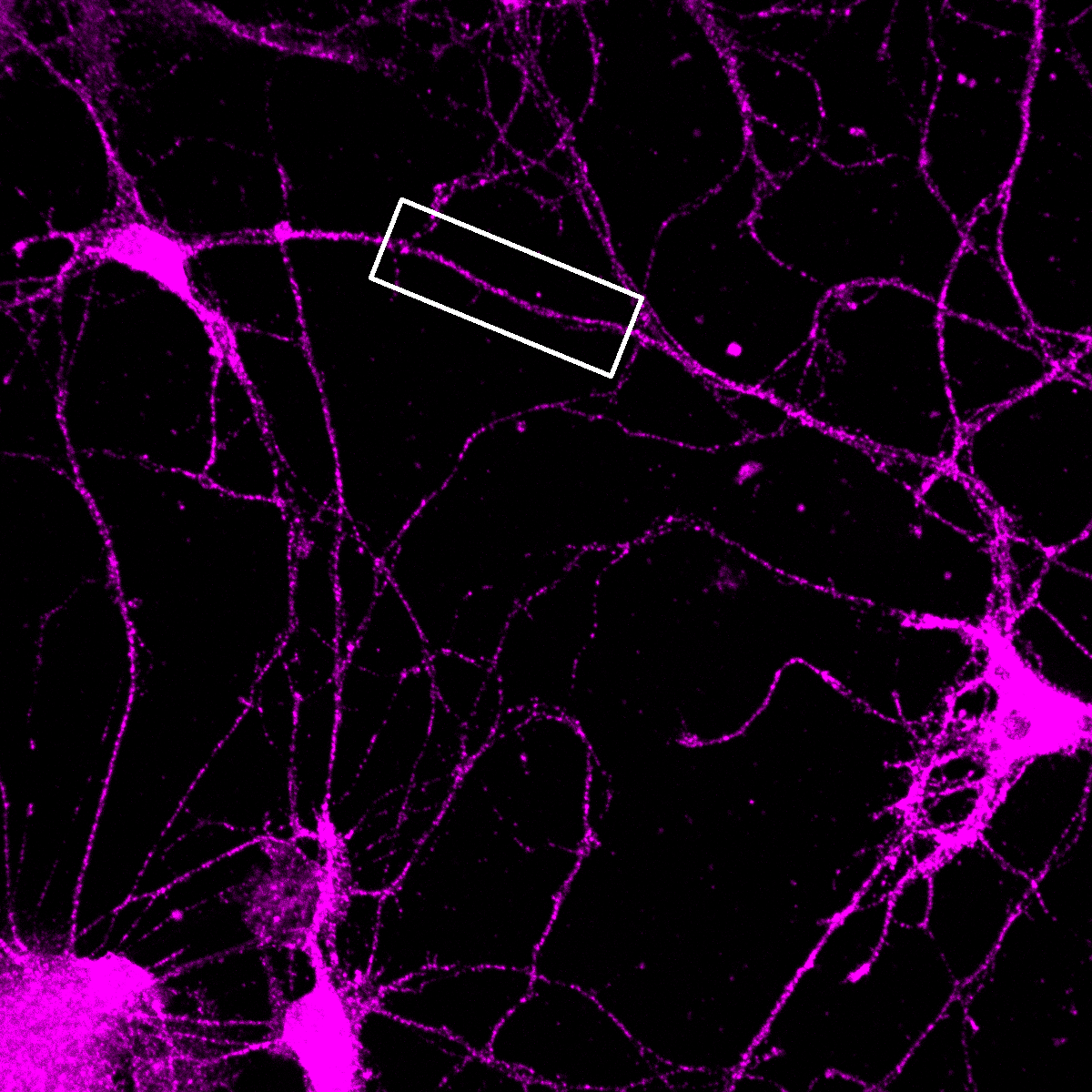

Supplement: Supplementary file 28 — Source data Fig. 6 [file 44318_2025_609_MOESM28_ESM.zip › EMBOJ-2024-119578_SourceDataForFigure6/6E/Control-TIA1.tif]

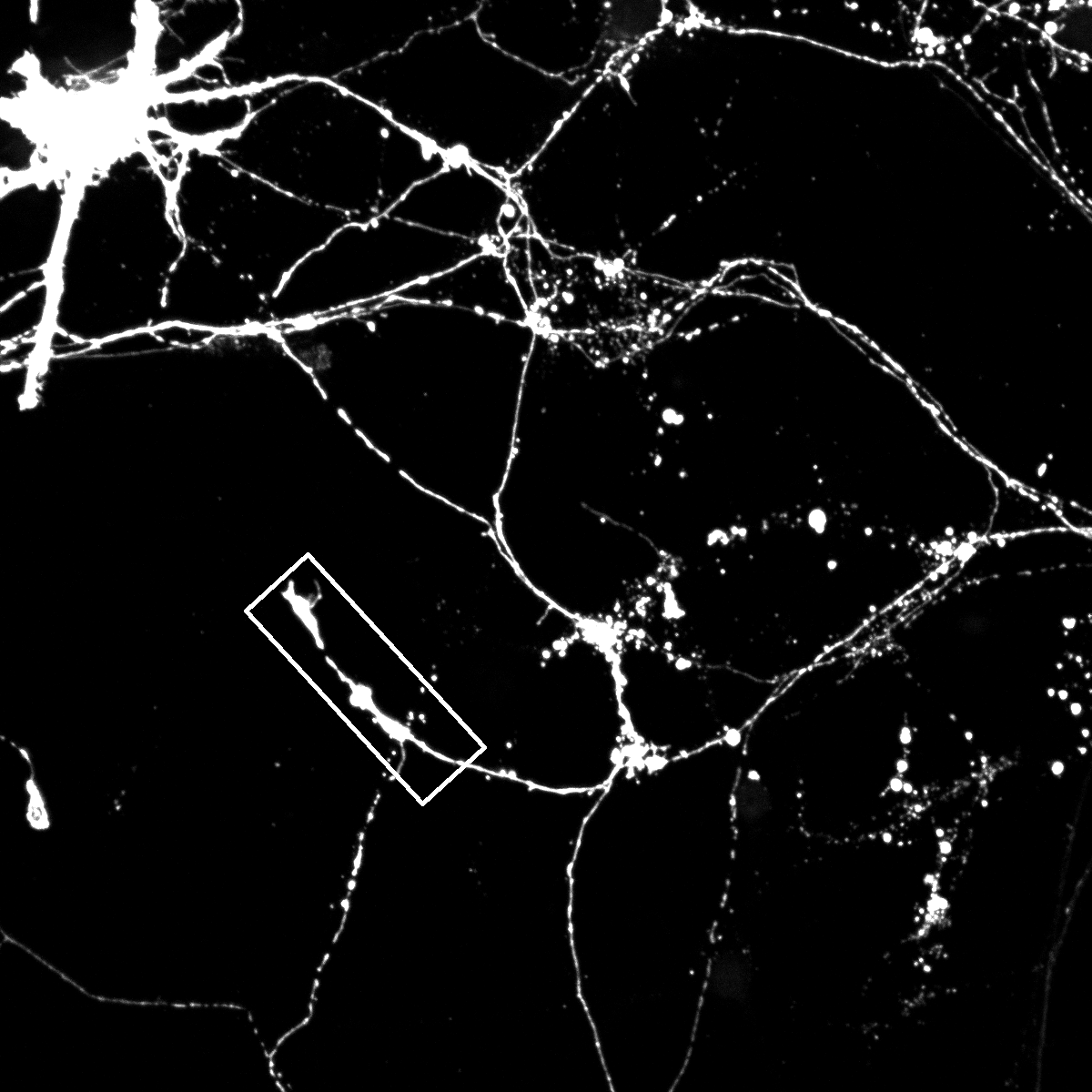

Supplement: Supplementary file 28 — Source data Fig. 6 [file 44318_2025_609_MOESM28_ESM.zip › EMBOJ-2024-119578_SourceDataForFigure6/6E/shANXA7-1#-EGFP.tif]

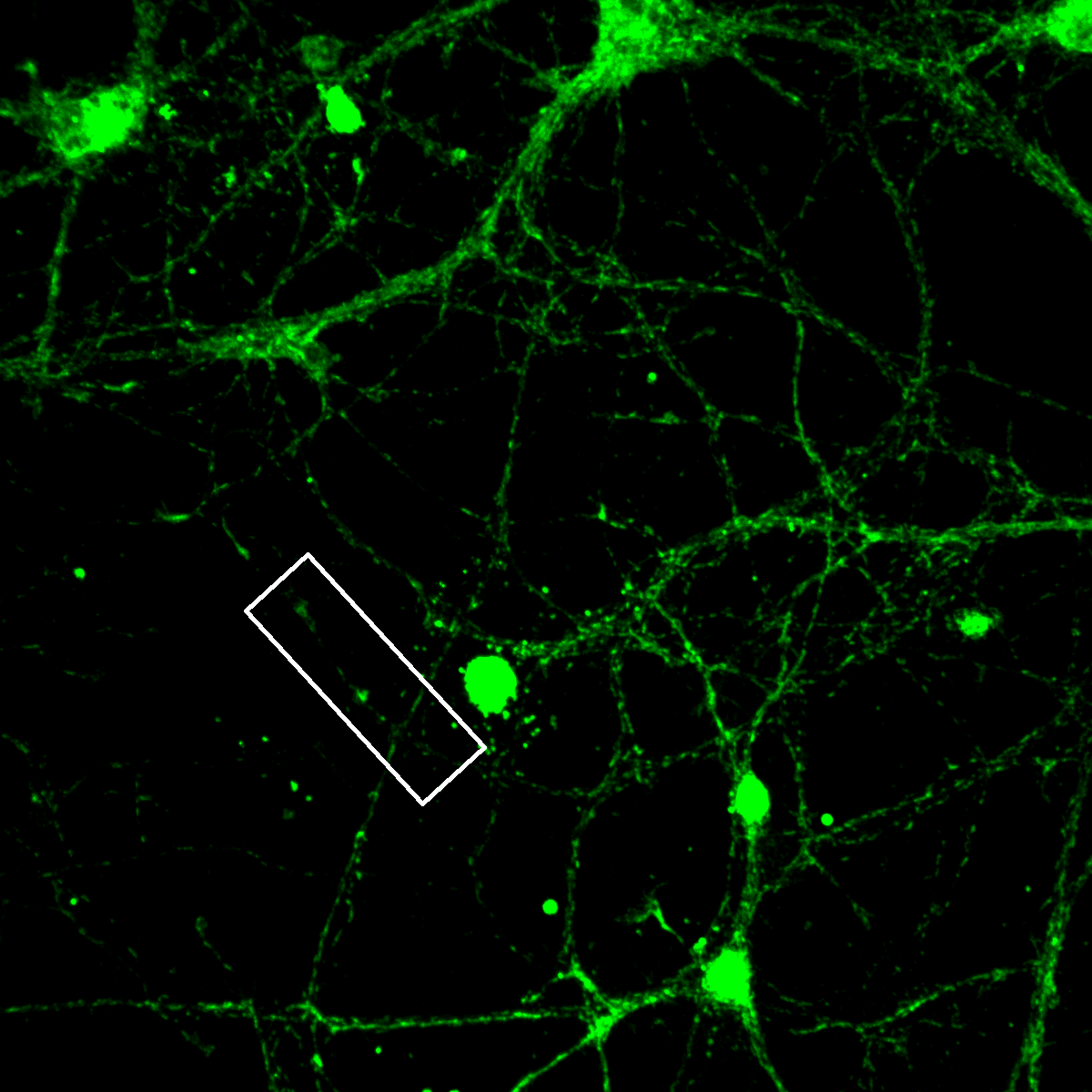

Supplement: Supplementary file 28 — Source data Fig. 6 [file 44318_2025_609_MOESM28_ESM.zip › EMBOJ-2024-119578_SourceDataForFigure6/6E/shANXA7-1#-p62.tif]

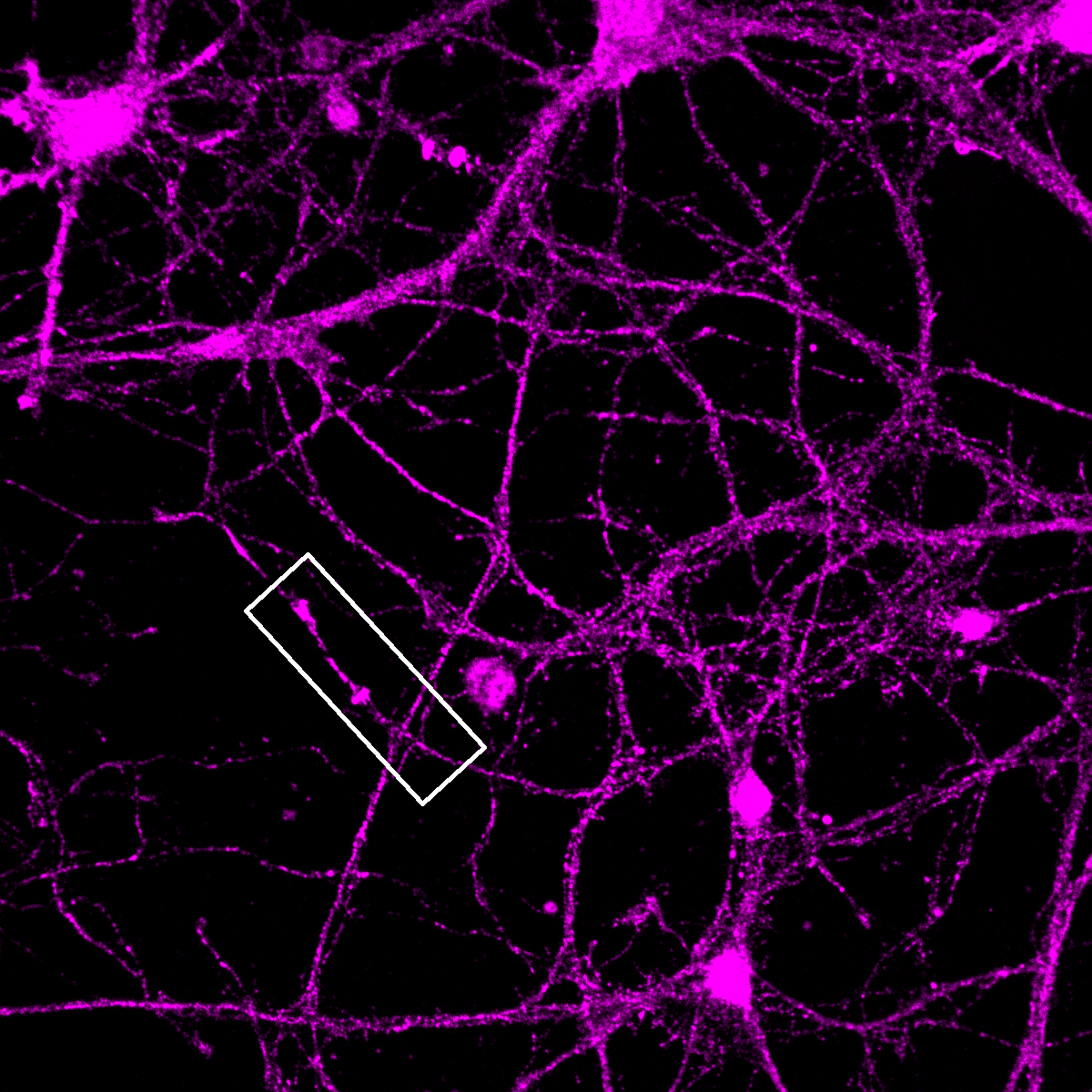

Supplement: Supplementary file 28 — Source data Fig. 6 [file 44318_2025_609_MOESM28_ESM.zip › EMBOJ-2024-119578_SourceDataForFigure6/6E/shANXA7-1#-TIA1.tif]

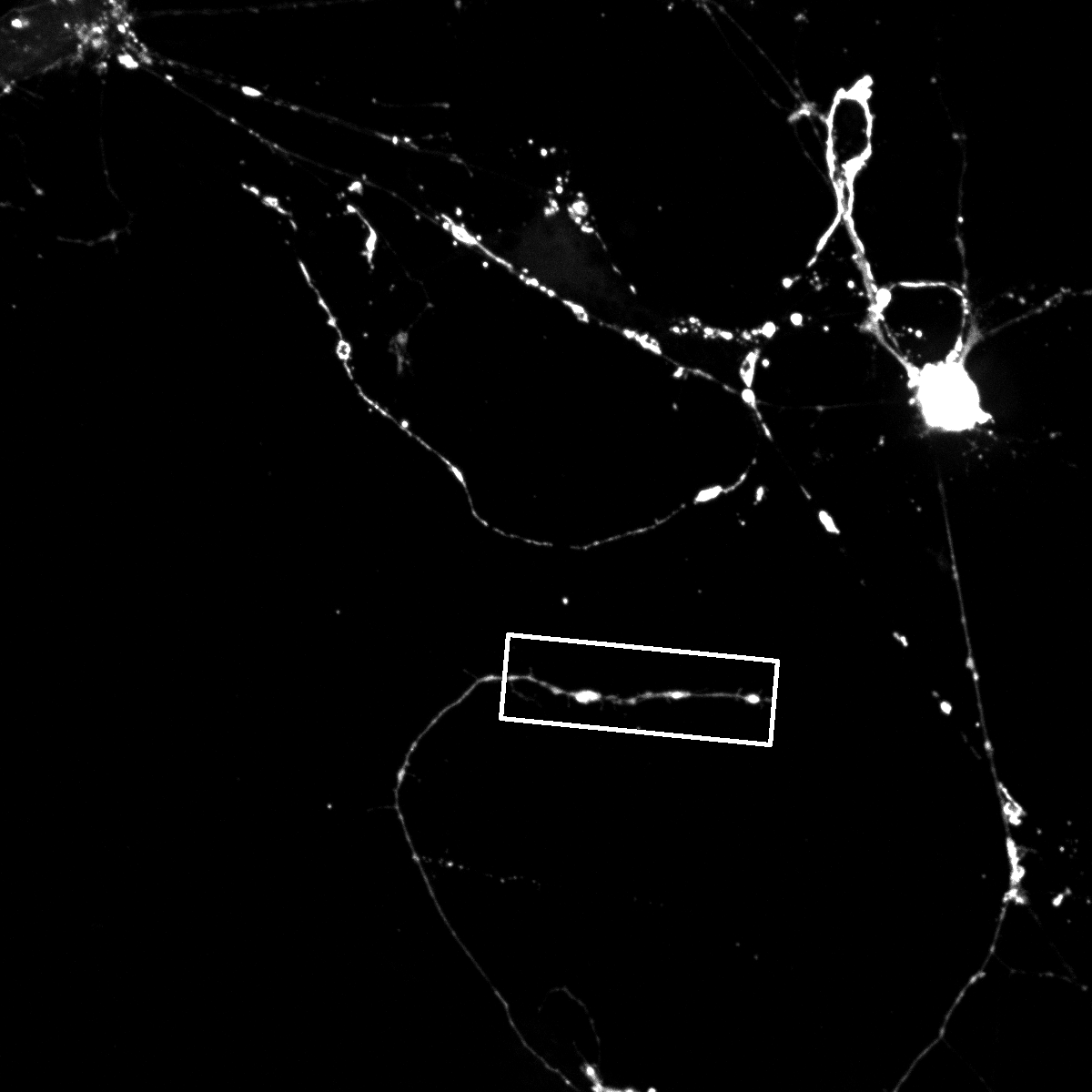

Supplement: Supplementary file 28 — Source data Fig. 6 [file 44318_2025_609_MOESM28_ESM.zip › EMBOJ-2024-119578_SourceDataForFigure6/6E/shANXA7-2#-EGFP.tif]

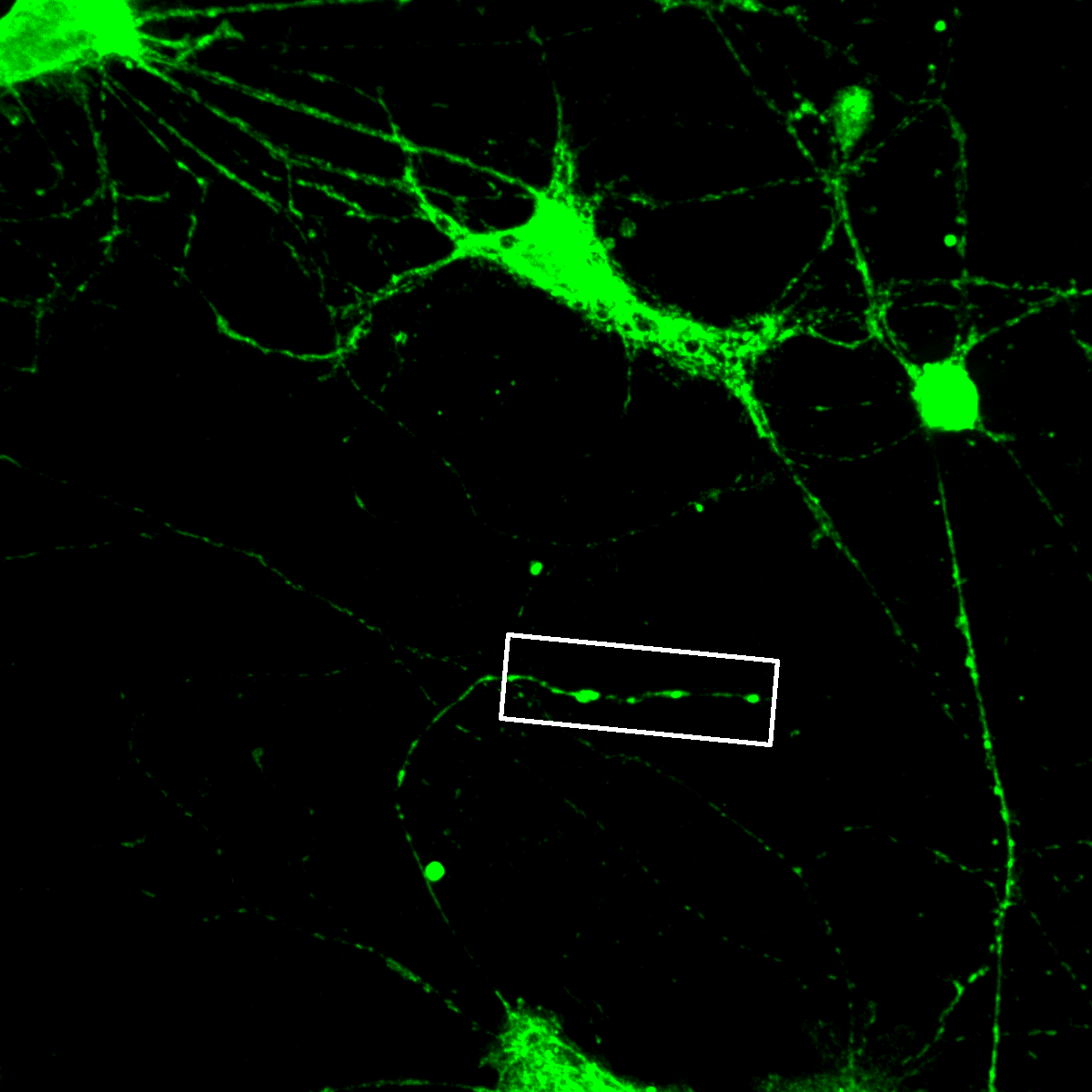

Supplement: Supplementary file 28 — Source data Fig. 6 [file 44318_2025_609_MOESM28_ESM.zip › EMBOJ-2024-119578_SourceDataForFigure6/6E/shANXA7-2#-p62.tif]

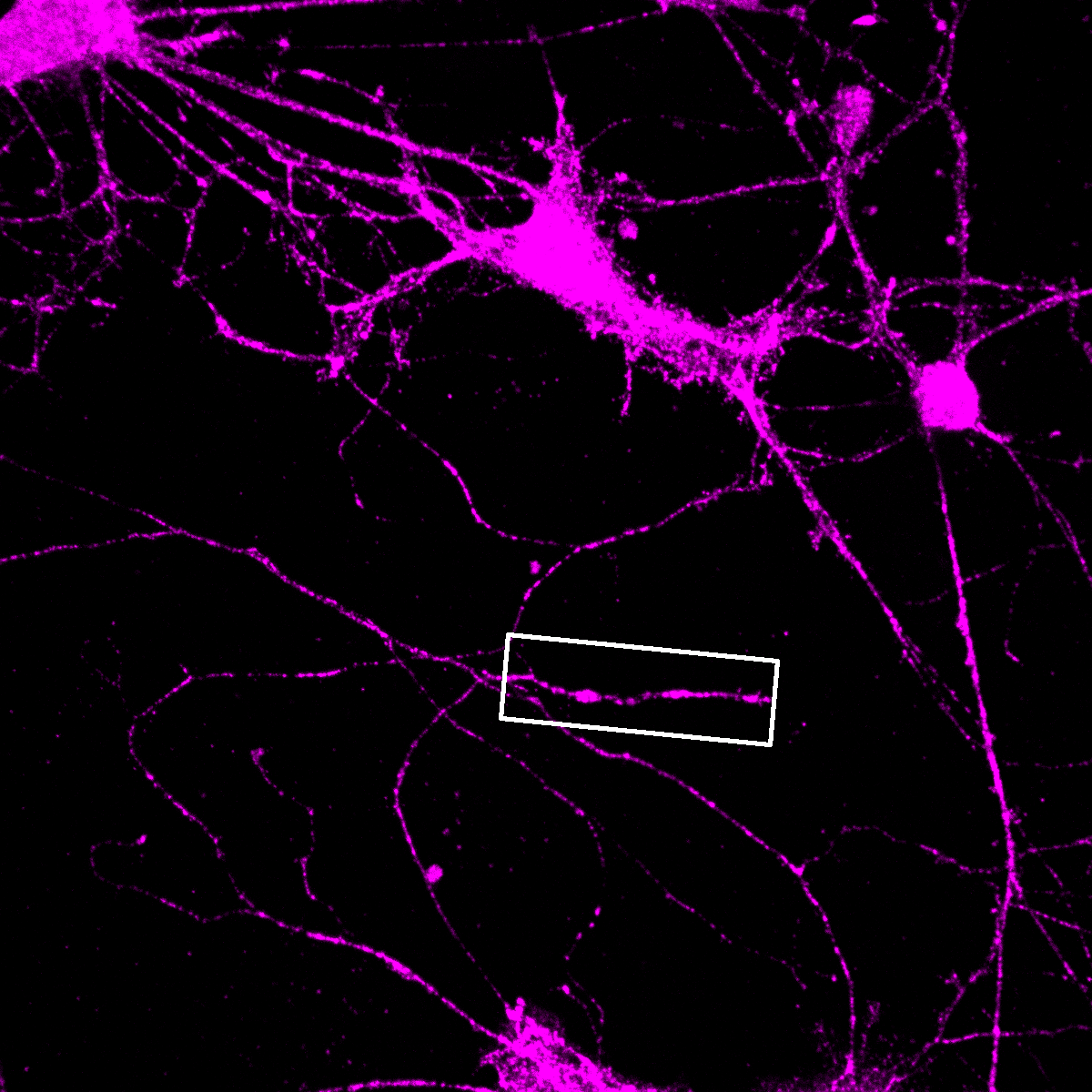

Supplement: Supplementary file 28 — Source data Fig. 6 [file 44318_2025_609_MOESM28_ESM.zip › EMBOJ-2024-119578_SourceDataForFigure6/6E/shANXA7-2#-TIA1.tif]

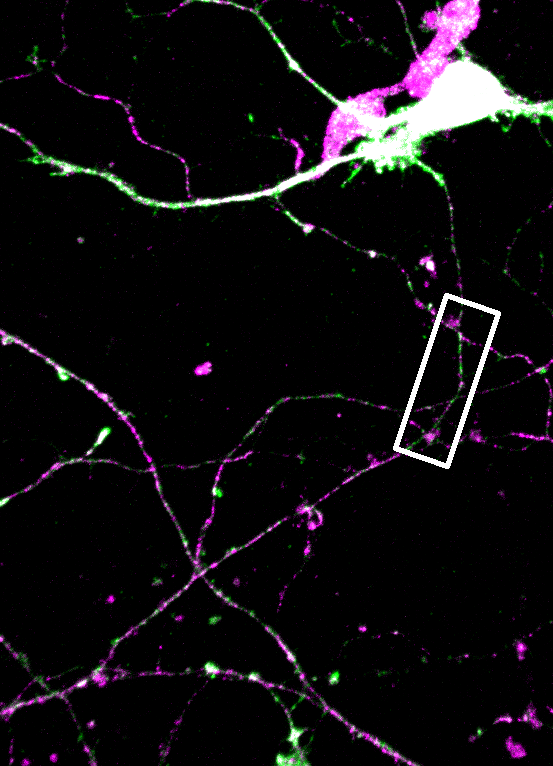

Supplement: Supplementary file 28 — Source data Fig. 6 [file 44318_2025_609_MOESM28_ESM.zip › EMBOJ-2024-119578_SourceDataForFigure6/6F/Control-Merge.tif]

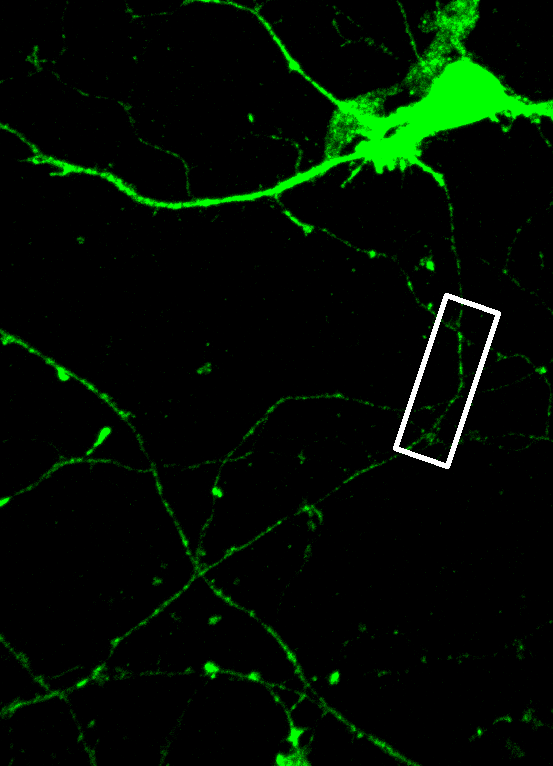

Supplement: Supplementary file 28 — Source data Fig. 6 [file 44318_2025_609_MOESM28_ESM.zip › EMBOJ-2024-119578_SourceDataForFigure6/6F/Control-p62.tif]

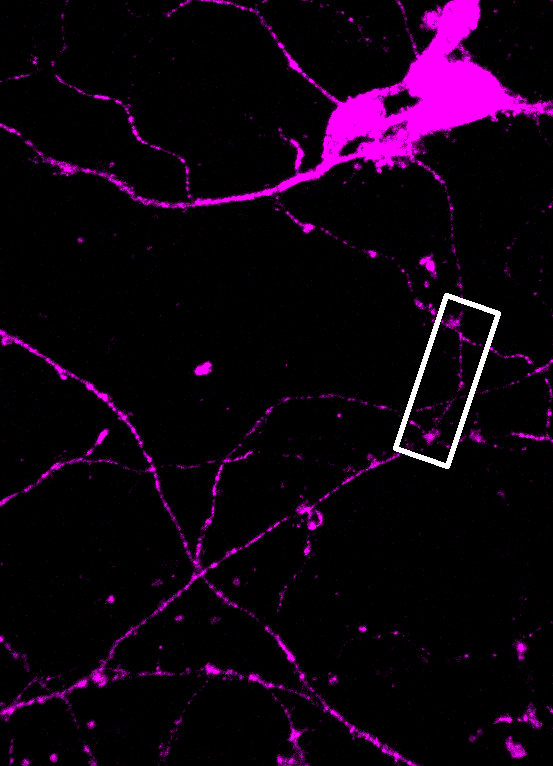

Supplement: Supplementary file 28 — Source data Fig. 6 [file 44318_2025_609_MOESM28_ESM.zip › EMBOJ-2024-119578_SourceDataForFigure6/6F/Control-TIA1.tif]

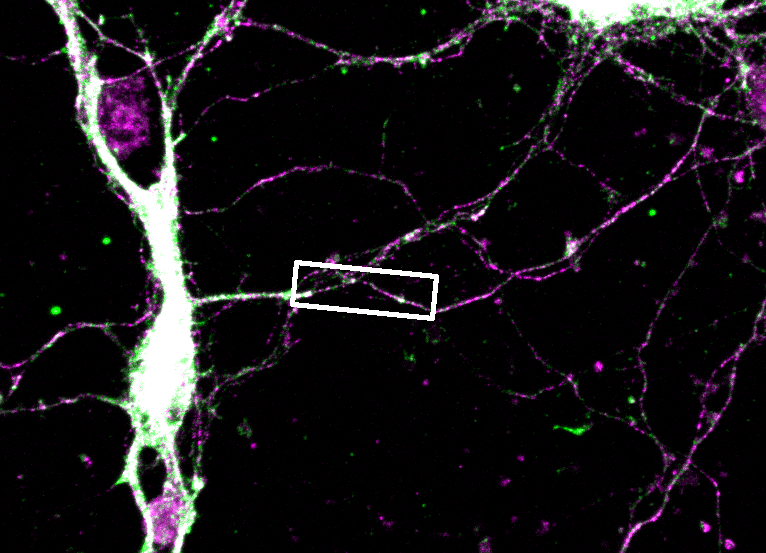

Supplement: Supplementary file 28 — Source data Fig. 6 [file 44318_2025_609_MOESM28_ESM.zip › EMBOJ-2024-119578_SourceDataForFigure6/6F/siANXA7-1#-Merge.tif]

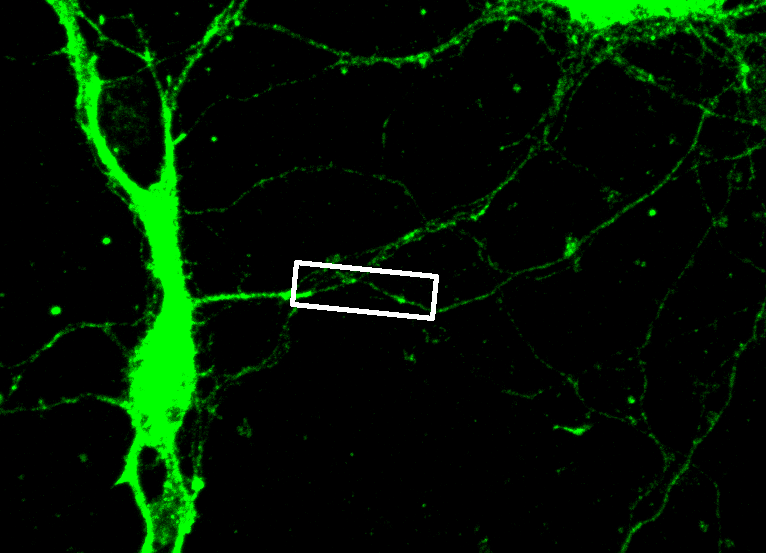

Supplement: Supplementary file 28 — Source data Fig. 6 [file 44318_2025_609_MOESM28_ESM.zip › EMBOJ-2024-119578_SourceDataForFigure6/6F/siANXA7-1#-p62.tif]

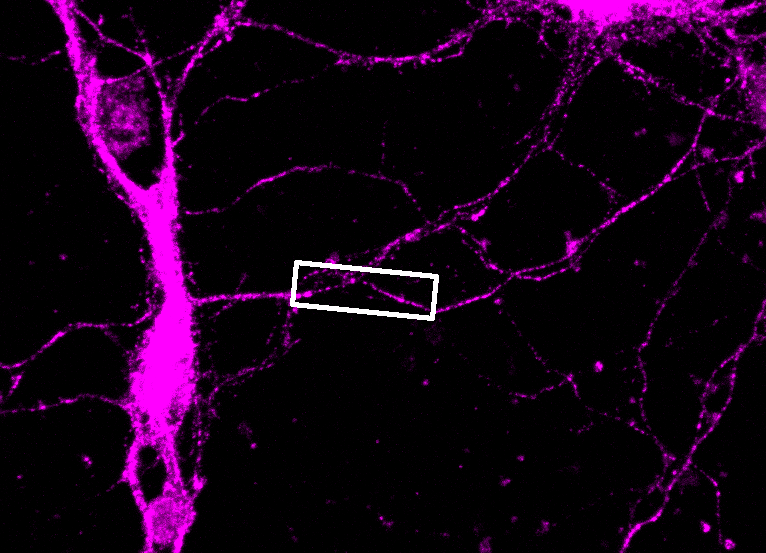

Supplement: Supplementary file 28 — Source data Fig. 6 [file 44318_2025_609_MOESM28_ESM.zip › EMBOJ-2024-119578_SourceDataForFigure6/6F/siANXA7-1#-TIA1.tif]

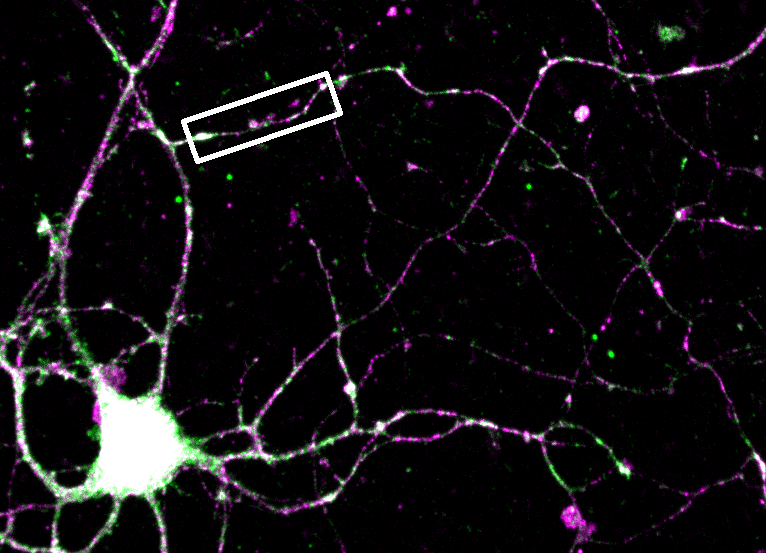

Supplement: Supplementary file 28 — Source data Fig. 6 [file 44318_2025_609_MOESM28_ESM.zip › EMBOJ-2024-119578_SourceDataForFigure6/6F/siANXA7-2#-Merge.tif]

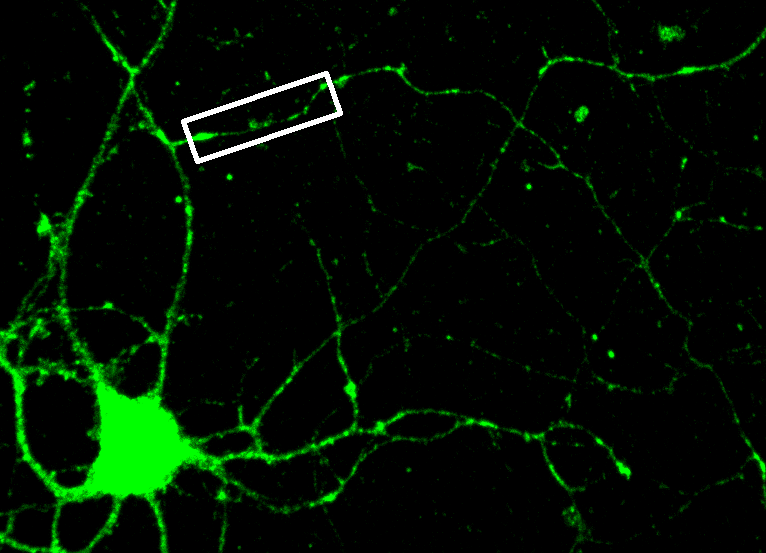

Supplement: Supplementary file 28 — Source data Fig. 6 [file 44318_2025_609_MOESM28_ESM.zip › EMBOJ-2024-119578_SourceDataForFigure6/6F/siANXA7-2#-p62.tif]

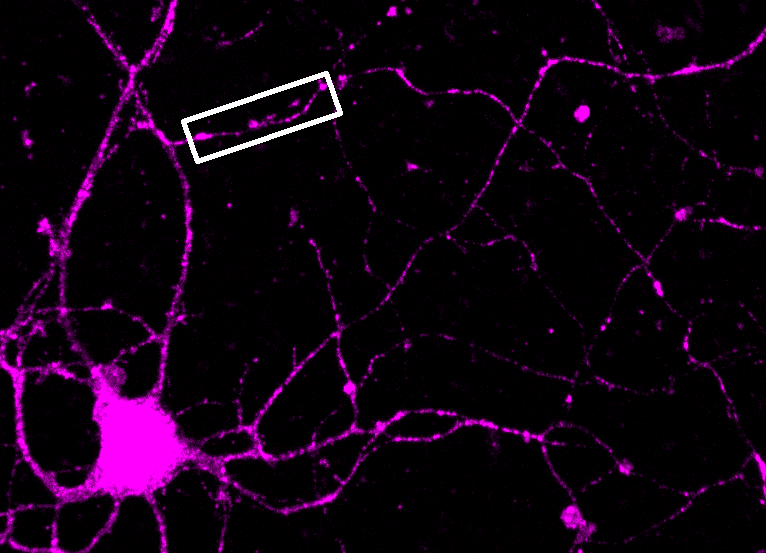

Supplement: Supplementary file 28 — Source data Fig. 6 [file 44318_2025_609_MOESM28_ESM.zip › EMBOJ-2024-119578_SourceDataForFigure6/6F/siANXA7-2#-TIA1.tif]

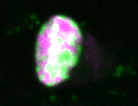

Supplement: Supplementary file 28 — Source data Fig. 6 [file 44318_2025_609_MOESM28_ESM.zip › EMBOJ-2024-119578_SourceDataForFigure6/6G/1_TDP-43 with DAPI_Control.tif]

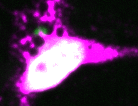

Supplement: Supplementary file 28 — Source data Fig. 6 [file 44318_2025_609_MOESM28_ESM.zip › EMBOJ-2024-119578_SourceDataForFigure6/6G/1_TDP-43 with DAPI_shA7-1#.tif]

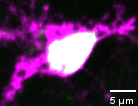

Supplement: Supplementary file 28 — Source data Fig. 6 [file 44318_2025_609_MOESM28_ESM.zip › EMBOJ-2024-119578_SourceDataForFigure6/6G/1_TDP-43 with DAPI_shA7-2#.tif]

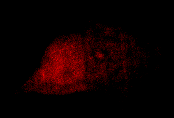

Supplement: Supplementary file 28 — Source data Fig. 6 [file 44318_2025_609_MOESM28_ESM.zip › EMBOJ-2024-119578_SourceDataForFigure6/6G/2_PI_Control.tif]

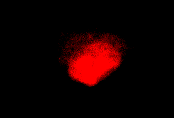

Supplement: Supplementary file 28 — Source data Fig. 6 [file 44318_2025_609_MOESM28_ESM.zip › EMBOJ-2024-119578_SourceDataForFigure6/6G/2_PI_shA7-1#.tif]

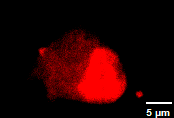

Supplement: Supplementary file 28 — Source data Fig. 6 [file 44318_2025_609_MOESM28_ESM.zip › EMBOJ-2024-119578_SourceDataForFigure6/6G/2_PI_shA7-2#.tif]

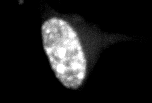

Supplement: Supplementary file 28 — Source data Fig. 6 [file 44318_2025_609_MOESM28_ESM.zip › EMBOJ-2024-119578_SourceDataForFigure6/6G/3_DAPI_Control.tif]

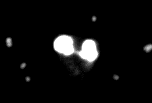

Supplement: Supplementary file 28 — Source data Fig. 6 [file 44318_2025_609_MOESM28_ESM.zip › EMBOJ-2024-119578_SourceDataForFigure6/6G/3_DAPI_shA7-1#.tif]

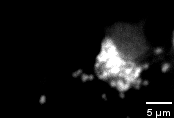

Supplement: Supplementary file 28 — Source data Fig. 6 [file 44318_2025_609_MOESM28_ESM.zip › EMBOJ-2024-119578_SourceDataForFigure6/6G/3_DAPI_shA7-2#.tif]

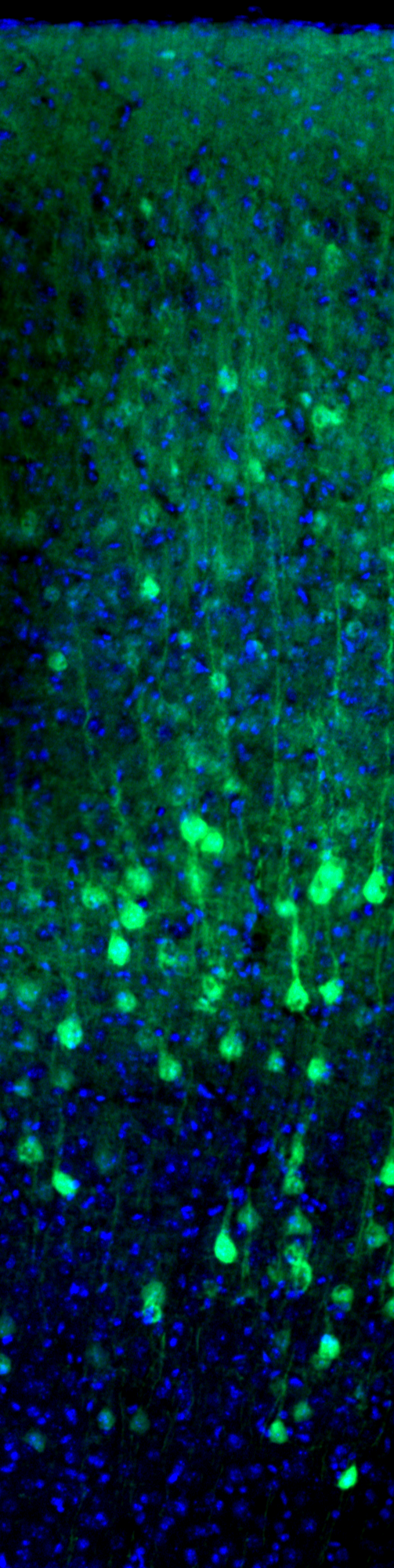

Supplement: Supplementary file 29 — Source data Fig. 7 [file 44318_2025_609_MOESM29_ESM.zip › EMBOJ-2024-119578_SourceDataForFigure7/7D/Control.tif]

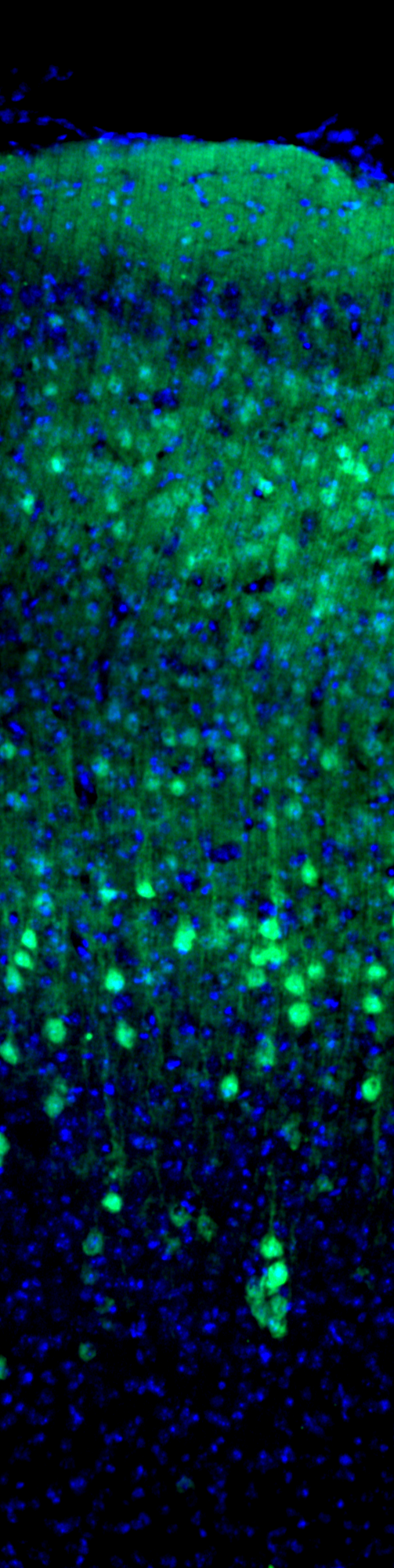

Supplement: Supplementary file 29 — Source data Fig. 7 [file 44318_2025_609_MOESM29_ESM.zip › EMBOJ-2024-119578_SourceDataForFigure7/7D/shA7-3#.tif]

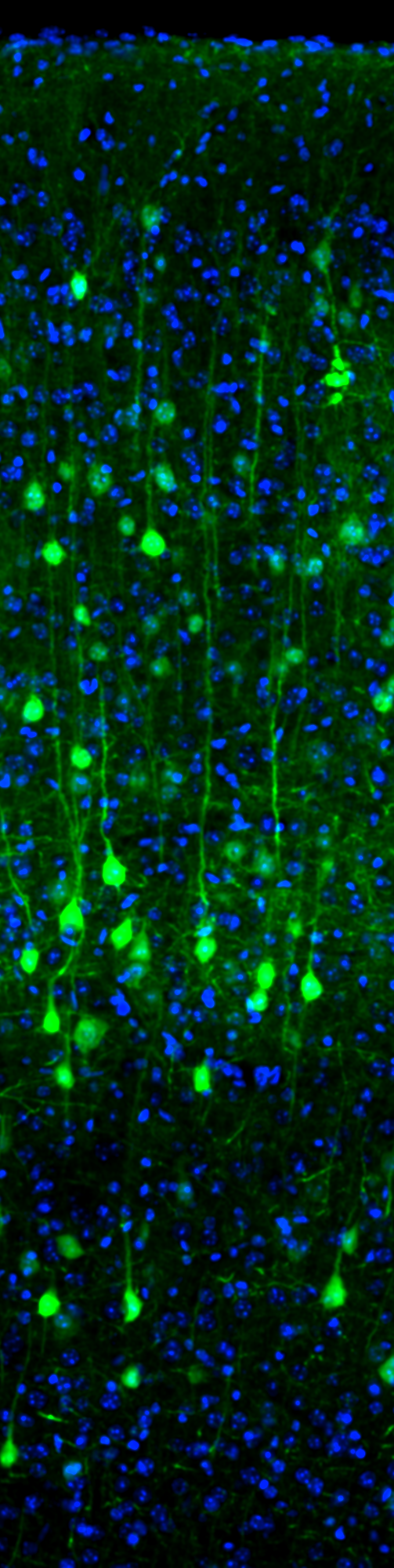

Supplement: Supplementary file 29 — Source data Fig. 7 [file 44318_2025_609_MOESM29_ESM.zip › EMBOJ-2024-119578_SourceDataForFigure7/7D/shA7-4#-res.tif]

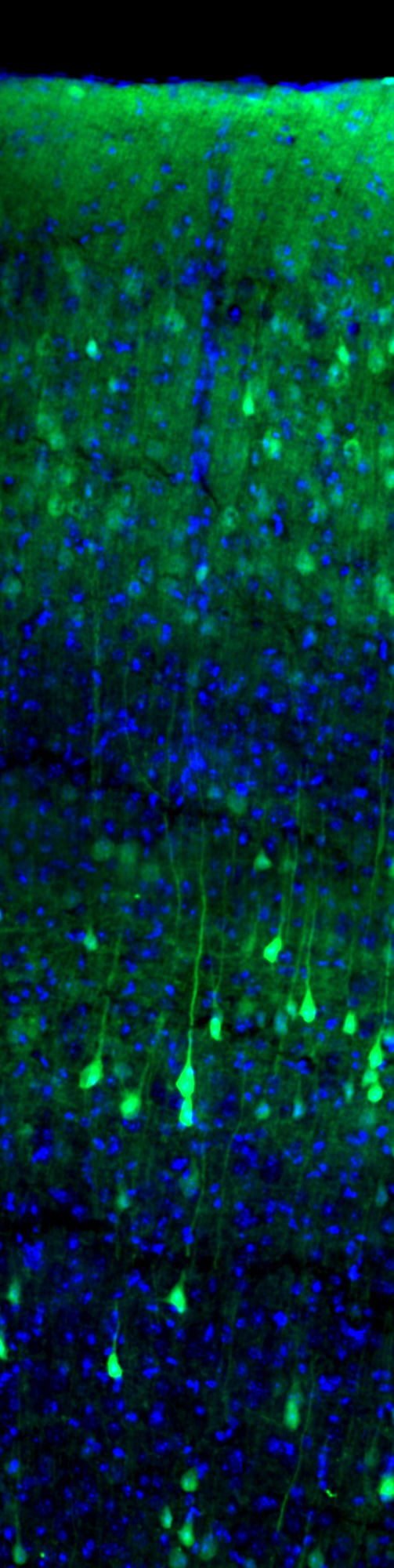

Supplement: Supplementary file 29 — Source data Fig. 7 [file 44318_2025_609_MOESM29_ESM.zip › EMBOJ-2024-119578_SourceDataForFigure7/7D/shA7-4#.tif]

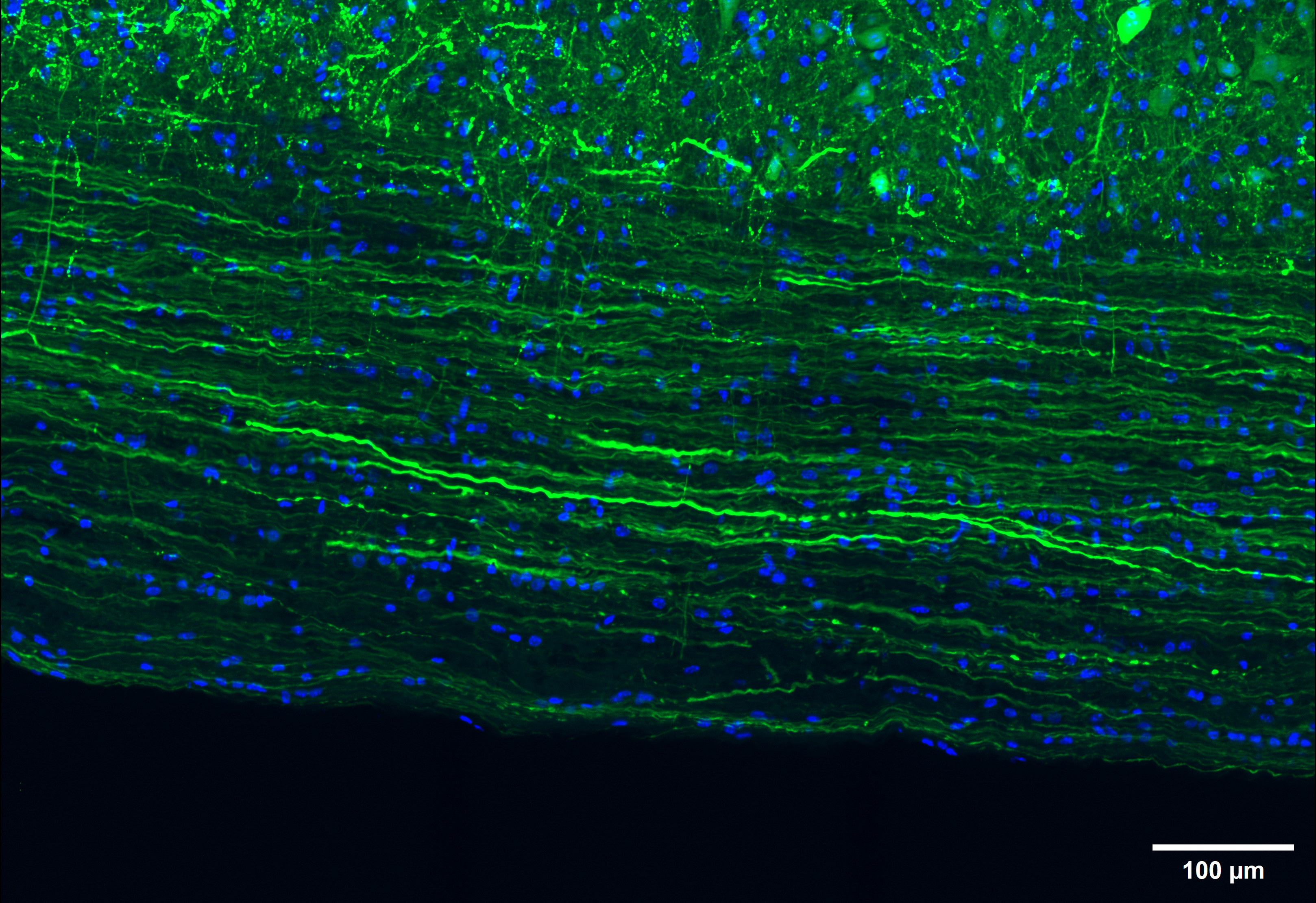

Supplement: Supplementary file 29 — Source data Fig. 7 [file 44318_2025_609_MOESM29_ESM.zip › EMBOJ-2024-119578_SourceDataForFigure7/7E/2_large image for Lateral CST.tif]

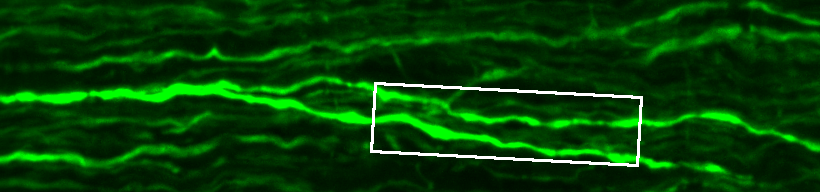

Supplement: Supplementary file 29 — Source data Fig. 7 [file 44318_2025_609_MOESM29_ESM.zip › EMBOJ-2024-119578_SourceDataForFigure7/7E/3_Control.tif]

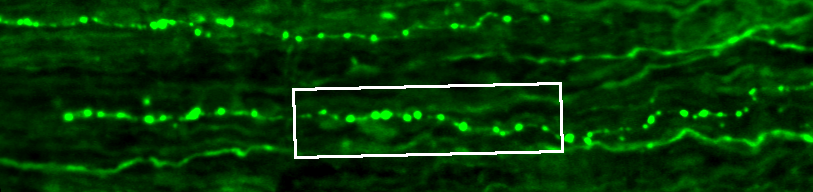

Supplement: Supplementary file 29 — Source data Fig. 7 [file 44318_2025_609_MOESM29_ESM.zip › EMBOJ-2024-119578_SourceDataForFigure7/7E/4_shA7-3#.tif]

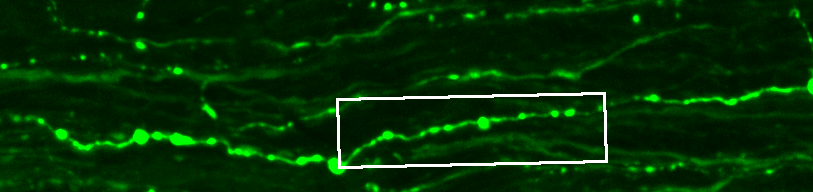

Supplement: Supplementary file 29 — Source data Fig. 7 [file 44318_2025_609_MOESM29_ESM.zip › EMBOJ-2024-119578_SourceDataForFigure7/7E/5_shA7-4#.tif]

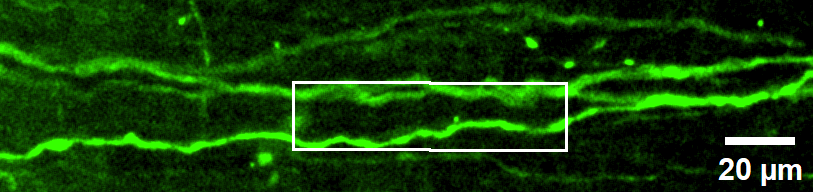

Supplement: Supplementary file 29 — Source data Fig. 7 [file 44318_2025_609_MOESM29_ESM.zip › EMBOJ-2024-119578_SourceDataForFigure7/7E/6_shA7-4#-res.tif]

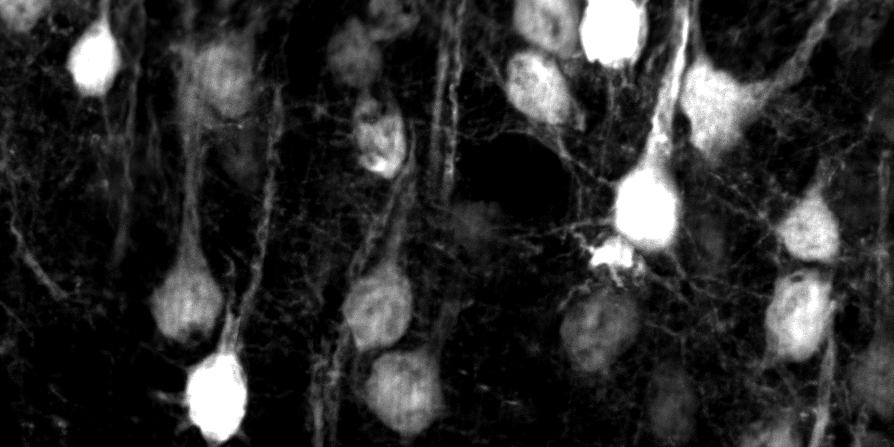

Supplement: Supplementary file 29 — Source data Fig. 7 [file 44318_2025_609_MOESM29_ESM.zip › EMBOJ-2024-119578_SourceDataForFigure7/7F/Control_EGFP.tif]

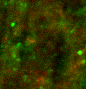

Supplement: Supplementary file 29 — Source data Fig. 7 [file 44318_2025_609_MOESM29_ESM.zip › EMBOJ-2024-119578_SourceDataForFigure7/7F/Control_Merge.tif]

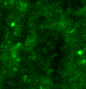

Supplement: Supplementary file 29 — Source data Fig. 7 [file 44318_2025_609_MOESM29_ESM.zip › EMBOJ-2024-119578_SourceDataForFigure7/7F/Control_p62.tif]

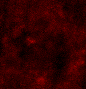

Supplement: Supplementary file 29 — Source data Fig. 7 [file 44318_2025_609_MOESM29_ESM.zip › EMBOJ-2024-119578_SourceDataForFigure7/7F/Control_TIA1.tif]

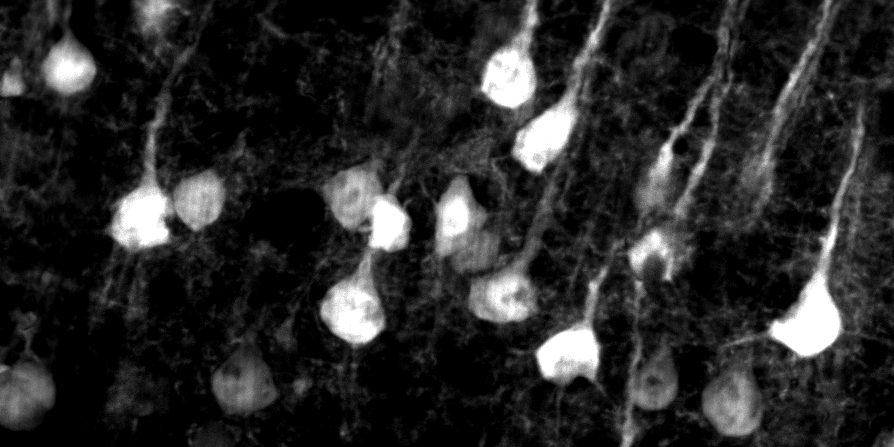

Supplement: Supplementary file 29 — Source data Fig. 7 [file 44318_2025_609_MOESM29_ESM.zip › EMBOJ-2024-119578_SourceDataForFigure7/7F/shA7-3#_EGFP.tif]

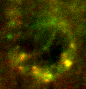

Supplement: Supplementary file 29 — Source data Fig. 7 [file 44318_2025_609_MOESM29_ESM.zip › EMBOJ-2024-119578_SourceDataForFigure7/7F/shA7-3#_Merge.tif]

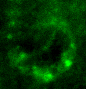

Supplement: Supplementary file 29 — Source data Fig. 7 [file 44318_2025_609_MOESM29_ESM.zip › EMBOJ-2024-119578_SourceDataForFigure7/7F/shA7-3#_p62.tif]

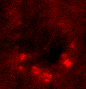

Supplement: Supplementary file 29 — Source data Fig. 7 [file 44318_2025_609_MOESM29_ESM.zip › EMBOJ-2024-119578_SourceDataForFigure7/7F/shA7-3#_TIA1.tif]

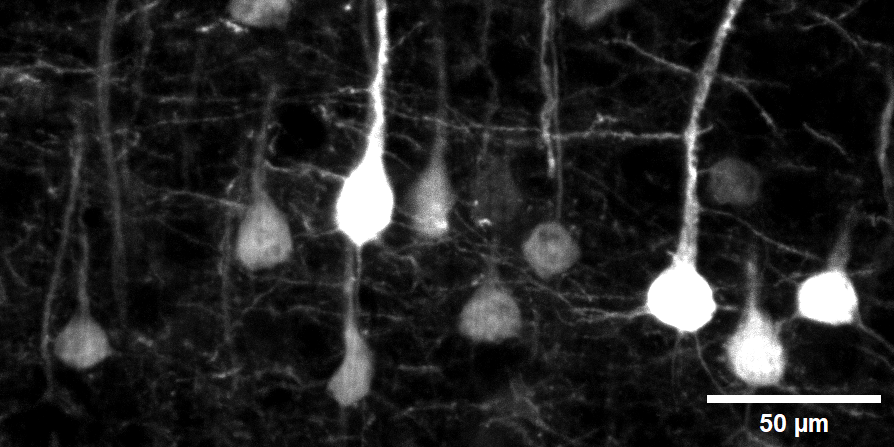

Supplement: Supplementary file 29 — Source data Fig. 7 [file 44318_2025_609_MOESM29_ESM.zip › EMBOJ-2024-119578_SourceDataForFigure7/7F/shA7-4#-res_EGFP.tif]

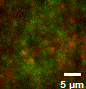

Supplement: Supplementary file 29 — Source data Fig. 7 [file 44318_2025_609_MOESM29_ESM.zip › EMBOJ-2024-119578_SourceDataForFigure7/7F/shA7-4#-res_Merge.tif]

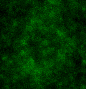

Supplement: Supplementary file 29 — Source data Fig. 7 [file 44318_2025_609_MOESM29_ESM.zip › EMBOJ-2024-119578_SourceDataForFigure7/7F/shA7-4#-res_p62.tif]

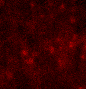

Supplement: Supplementary file 29 — Source data Fig. 7 [file 44318_2025_609_MOESM29_ESM.zip › EMBOJ-2024-119578_SourceDataForFigure7/7F/shA7-4#-res_TIA1.tif]

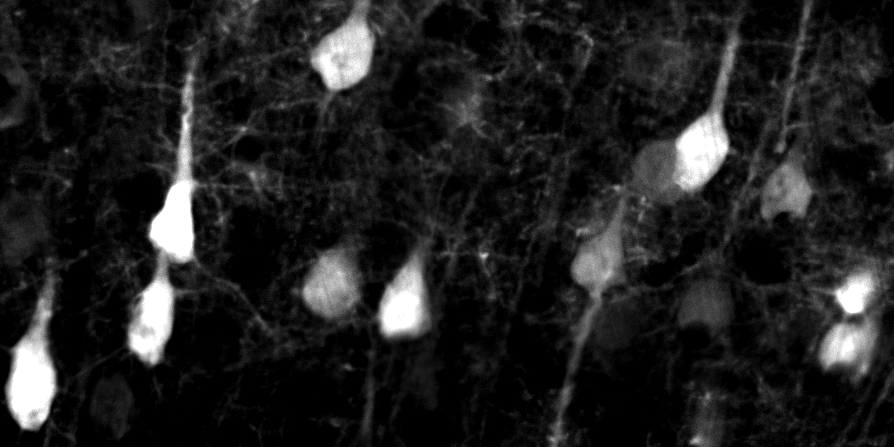

Supplement: Supplementary file 29 — Source data Fig. 7 [file 44318_2025_609_MOESM29_ESM.zip › EMBOJ-2024-119578_SourceDataForFigure7/7F/shA7-4#_EGFP.tif]

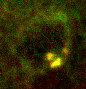

Supplement: Supplementary file 29 — Source data Fig. 7 [file 44318_2025_609_MOESM29_ESM.zip › EMBOJ-2024-119578_SourceDataForFigure7/7F/shA7-4#_Merge.tif]

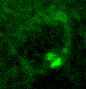

Supplement: Supplementary file 29 — Source data Fig. 7 [file 44318_2025_609_MOESM29_ESM.zip › EMBOJ-2024-119578_SourceDataForFigure7/7F/shA7-4#_p62.tif]

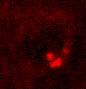

Supplement: Supplementary file 29 — Source data Fig. 7 [file 44318_2025_609_MOESM29_ESM.zip › EMBOJ-2024-119578_SourceDataForFigure7/7F/shA7-4#_TIA1.tif]

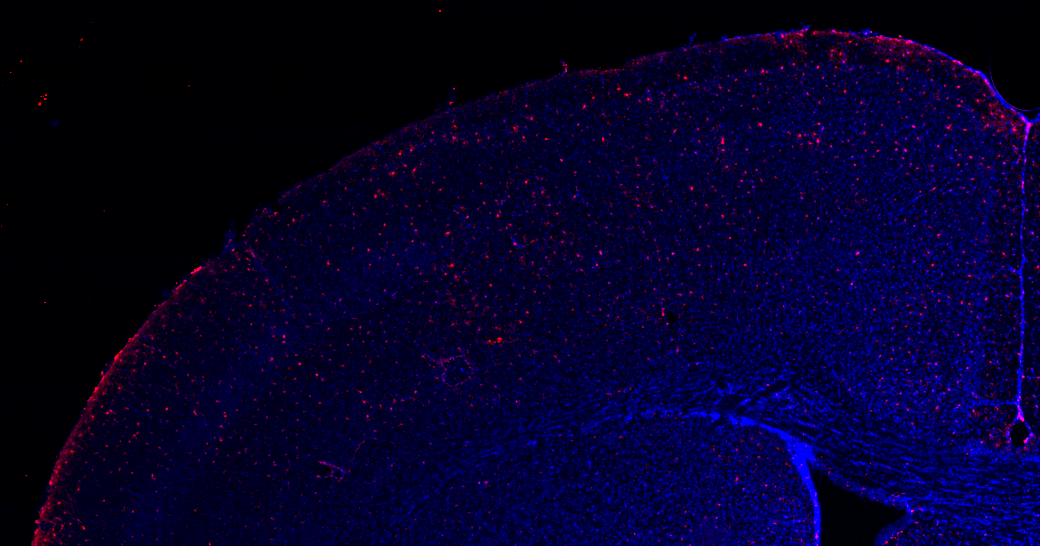

Supplement: Supplementary file 29 — Source data Fig. 7 [file 44318_2025_609_MOESM29_ESM.zip › EMBOJ-2024-119578_SourceDataForFigure7/7G/Control_large image.tif]

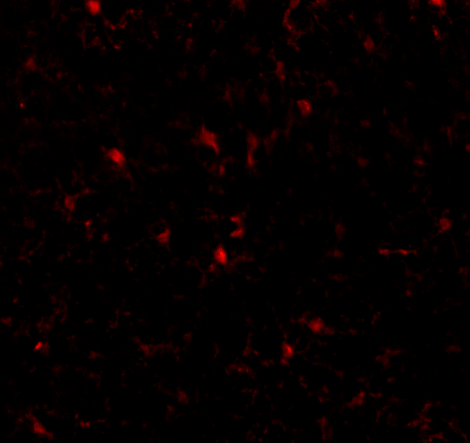

Supplement: Supplementary file 29 — Source data Fig. 7 [file 44318_2025_609_MOESM29_ESM.zip › EMBOJ-2024-119578_SourceDataForFigure7/7G/Control_M1.tif]

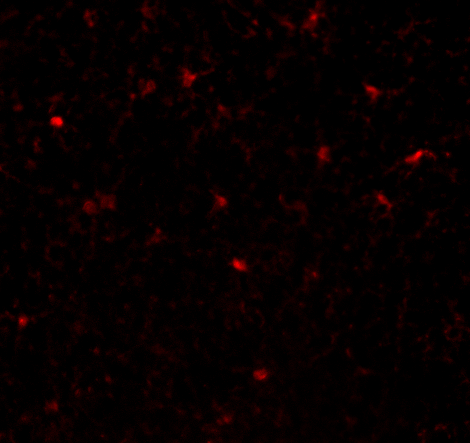

Supplement: Supplementary file 29 — Source data Fig. 7 [file 44318_2025_609_MOESM29_ESM.zip › EMBOJ-2024-119578_SourceDataForFigure7/7G/Control_M2.tif]

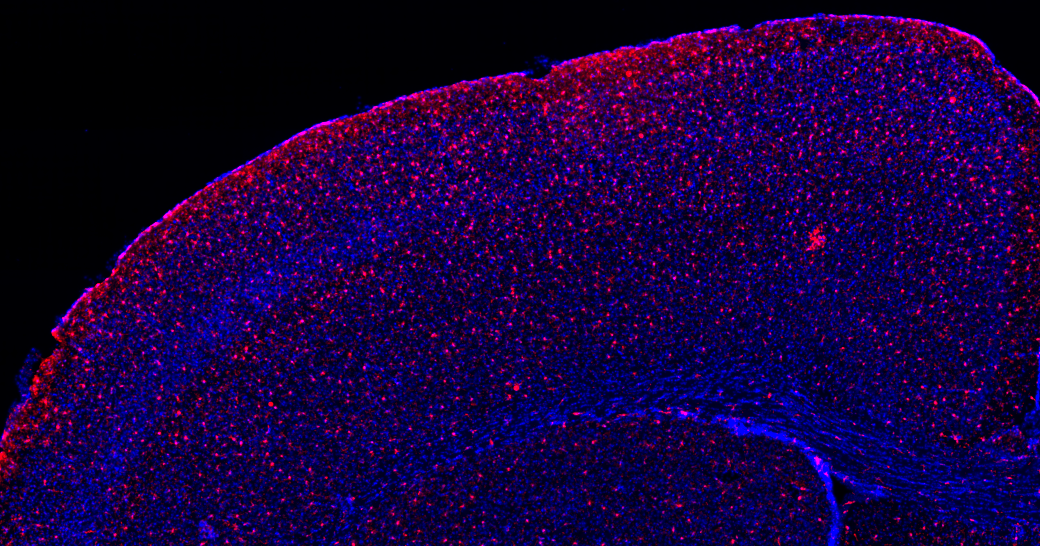

Supplement: Supplementary file 29 — Source data Fig. 7 [file 44318_2025_609_MOESM29_ESM.zip › EMBOJ-2024-119578_SourceDataForFigure7/7G/shA7-3#_large image.tif]

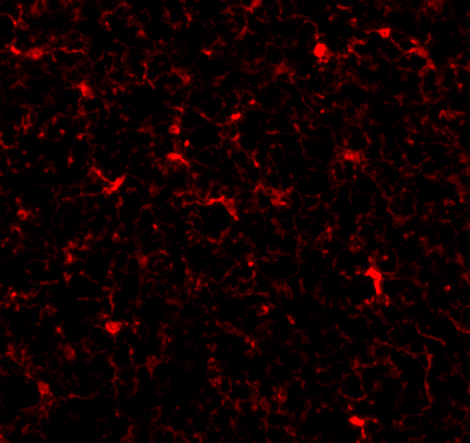

Supplement: Supplementary file 29 — Source data Fig. 7 [file 44318_2025_609_MOESM29_ESM.zip › EMBOJ-2024-119578_SourceDataForFigure7/7G/shA7-3#_M1.tif]

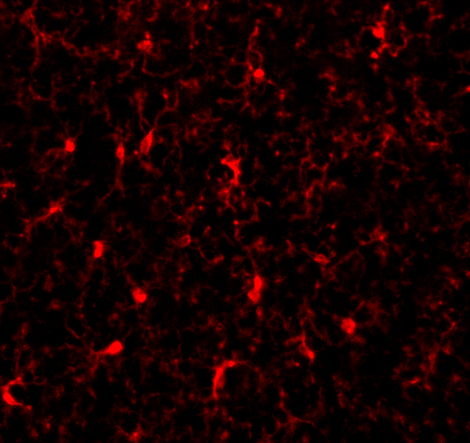

Supplement: Supplementary file 29 — Source data Fig. 7 [file 44318_2025_609_MOESM29_ESM.zip › EMBOJ-2024-119578_SourceDataForFigure7/7G/shA7-3#_M2.tif]

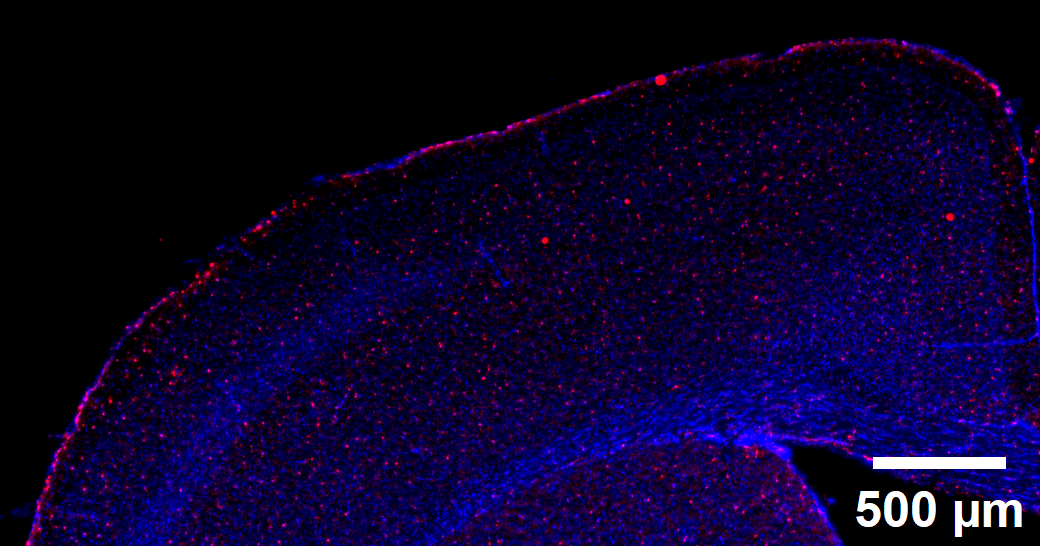

Supplement: Supplementary file 29 — Source data Fig. 7 [file 44318_2025_609_MOESM29_ESM.zip › EMBOJ-2024-119578_SourceDataForFigure7/7G/shA7-4#-res_large image.tif]

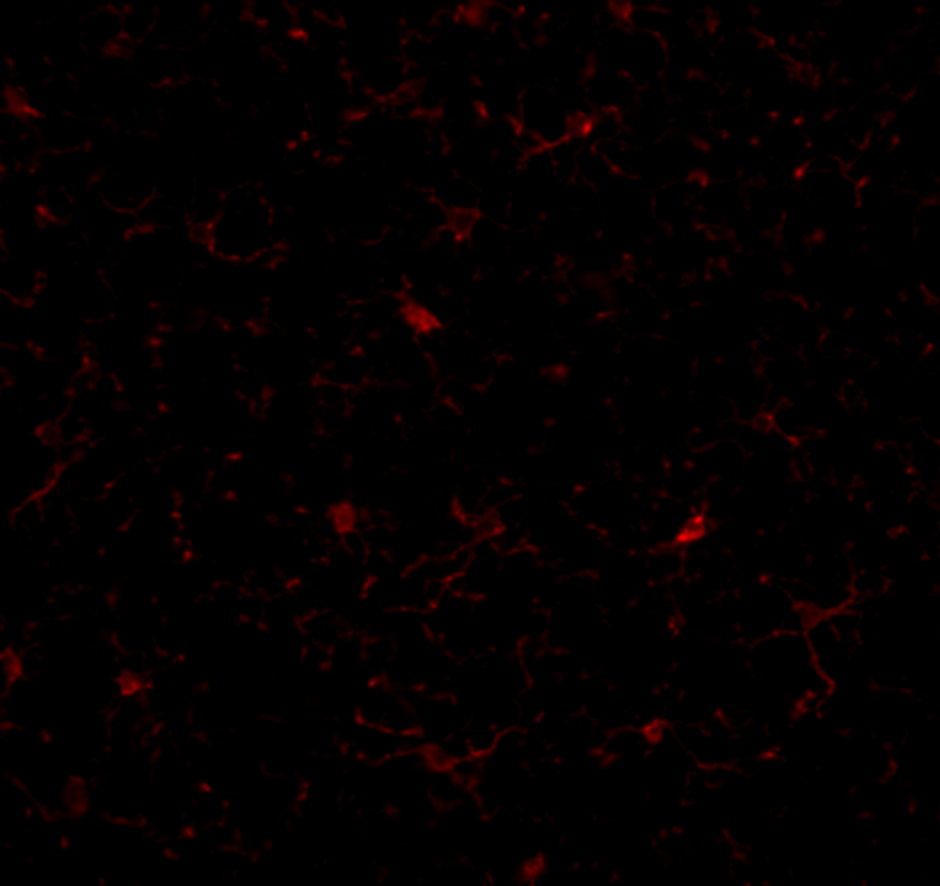

Supplement: Supplementary file 29 — Source data Fig. 7 [file 44318_2025_609_MOESM29_ESM.zip › EMBOJ-2024-119578_SourceDataForFigure7/7G/shA7-4#-res_M1.tif]

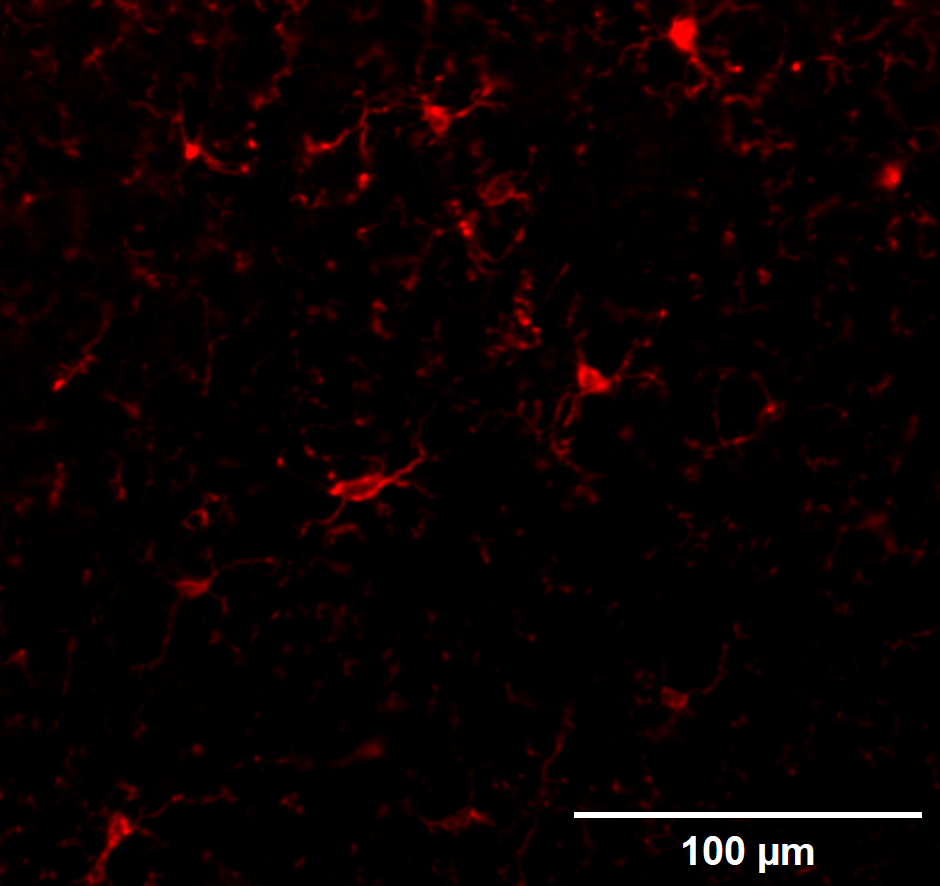

Supplement: Supplementary file 29 — Source data Fig. 7 [file 44318_2025_609_MOESM29_ESM.zip › EMBOJ-2024-119578_SourceDataForFigure7/7G/shA7-4#-res_M2.tif]

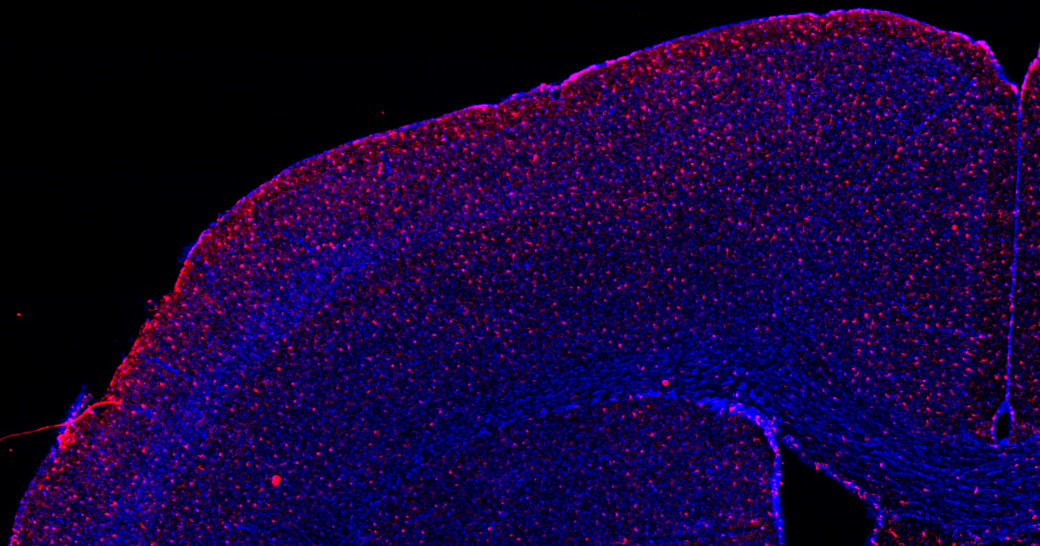

Supplement: Supplementary file 29 — Source data Fig. 7 [file 44318_2025_609_MOESM29_ESM.zip › EMBOJ-2024-119578_SourceDataForFigure7/7G/shA7-4#_large image.tif]

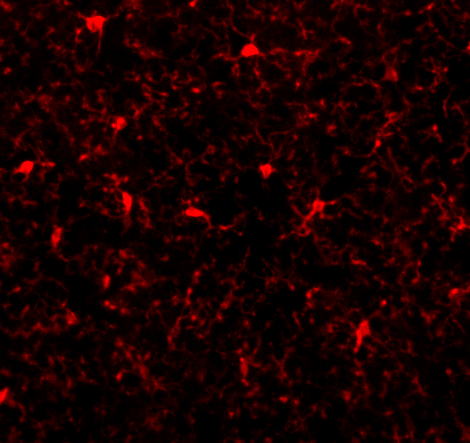

Supplement: Supplementary file 29 — Source data Fig. 7 [file 44318_2025_609_MOESM29_ESM.zip › EMBOJ-2024-119578_SourceDataForFigure7/7G/shA7-4#_M1.tif]

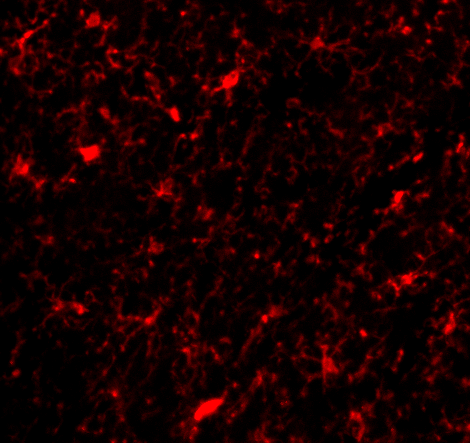

Supplement: Supplementary file 29 — Source data Fig. 7 [file 44318_2025_609_MOESM29_ESM.zip › EMBOJ-2024-119578_SourceDataForFigure7/7G/shA7-4#_M2.tif]

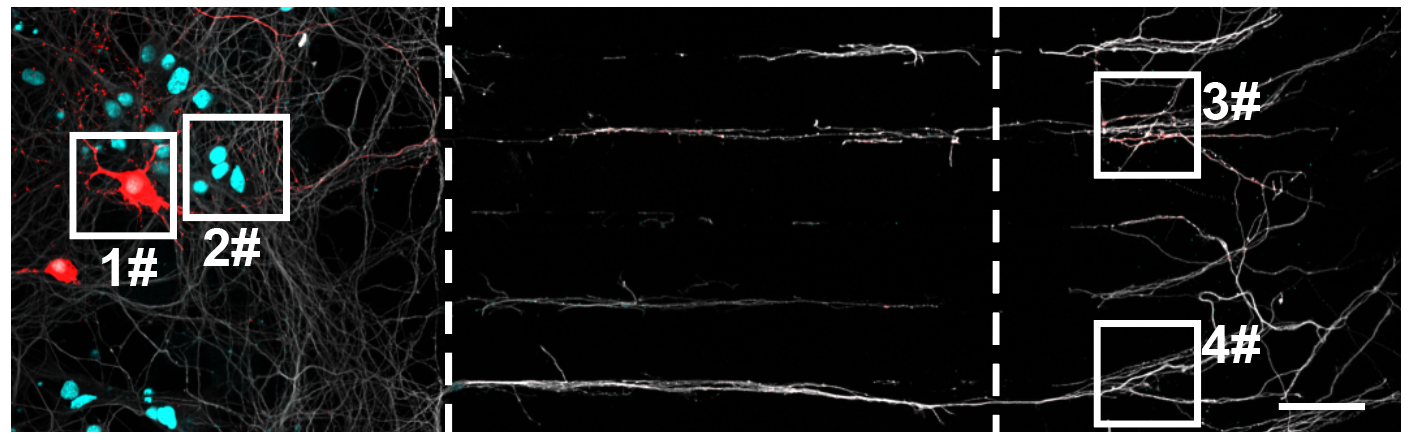

Supplement: Supplementary file 30 — Source Data For Expanded View [file 44318_2025_609_MOESM30_ESM.zip › SourceDataForExpandedView/Appendix figure/Appendix FigS1C/0-0 Appendix Fig. S1C with ROI.tif]

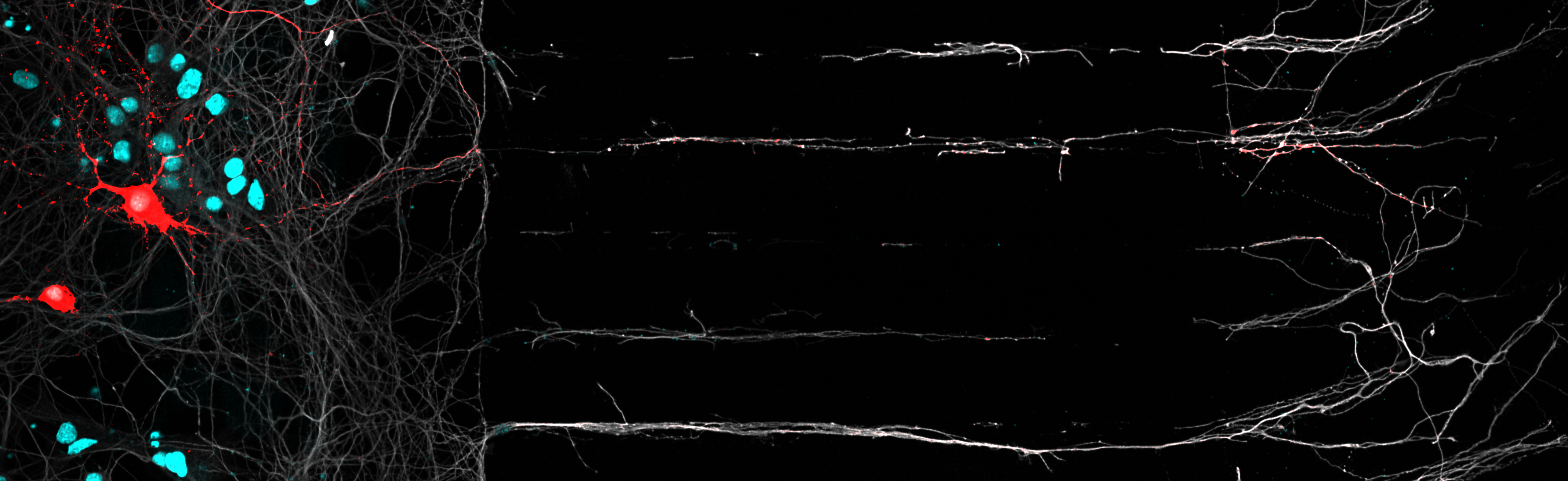

Supplement: Supplementary file 30 — Source Data For Expanded View [file 44318_2025_609_MOESM30_ESM.zip › SourceDataForExpandedView/Appendix figure/Appendix FigS1C/0-1 Appendix Fig. S1C raw.tif]

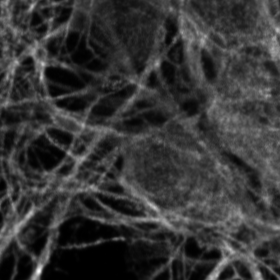

Supplement: Supplementary file 30 — Source Data For Expanded View [file 44318_2025_609_MOESM30_ESM.zip › SourceDataForExpandedView/Appendix figure/Appendix FigS1C/1#-B-Tub III.tif]

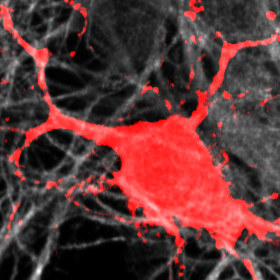

Supplement: Supplementary file 30 — Source Data For Expanded View [file 44318_2025_609_MOESM30_ESM.zip › SourceDataForExpandedView/Appendix figure/Appendix FigS1C/1#-Merge.tif]

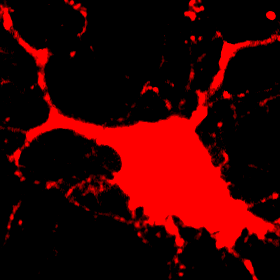

Supplement: Supplementary file 30 — Source Data For Expanded View [file 44318_2025_609_MOESM30_ESM.zip › SourceDataForExpandedView/Appendix figure/Appendix FigS1C/1#-TIA1-mCherry.tif]
