# Supplementary figures and images for: Annexin A7 enhances TIA1 axonal trafficking to counteract pathological aggregation in neurons (part 5 of 5)
Source: EMBO J. 2025 Nov 3;44(24):7477–512. doi: 10.1038/s44318-025-00609-8 (PMC12706091; doi:10.1038/s44318-025-00609-8)

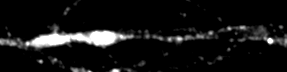

Supplement: Supplementary file 30 — Source Data For Expanded View [file 44318_2025_609_MOESM30_ESM.zip › SourceDataForExpandedView/FigureEV4/EV4G/shANXA7-1#-TIA1.tif]

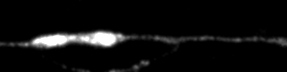

Supplement: Supplementary file 30 — Source Data For Expanded View [file 44318_2025_609_MOESM30_ESM.zip › SourceDataForExpandedView/FigureEV4/EV4G/shANXA7-1#-TDP43.tif]

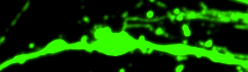

Supplement: Supplementary file 30 — Source Data For Expanded View [file 44318_2025_609_MOESM30_ESM.zip › SourceDataForExpandedView/FigureEV4/EV4G/shANXA7-2#-EGFP.tif]

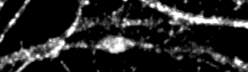

Supplement: Supplementary file 30 — Source Data For Expanded View [file 44318_2025_609_MOESM30_ESM.zip › SourceDataForExpandedView/FigureEV4/EV4G/shANXA7-2#-TIA1.tif]

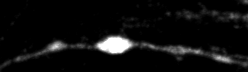

Supplement: Supplementary file 30 — Source Data For Expanded View [file 44318_2025_609_MOESM30_ESM.zip › SourceDataForExpandedView/FigureEV4/EV4G/shANXA7-2#-TDP43.tif]

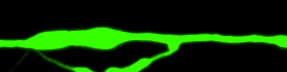

Supplement: Supplementary file 30 — Source Data For Expanded View [file 44318_2025_609_MOESM30_ESM.zip › SourceDataForExpandedView/FigureEV4/EV4G/shANXA7-1#-EGFP.tif]

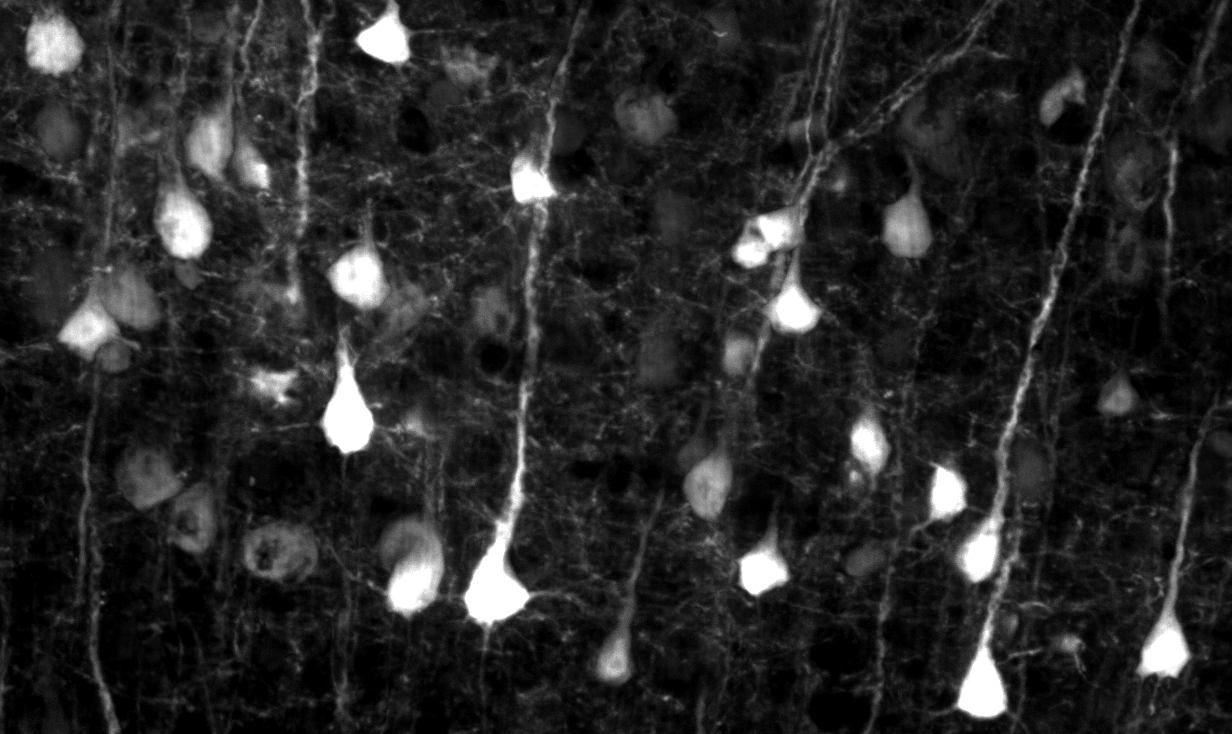

Supplement: Supplementary file 30 — Source Data For Expanded View [file 44318_2025_609_MOESM30_ESM.zip › SourceDataForExpandedView/FigureEV5/EV5C/Control_GFP.tif]

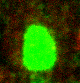

Supplement: Supplementary file 30 — Source Data For Expanded View [file 44318_2025_609_MOESM30_ESM.zip › SourceDataForExpandedView/FigureEV5/EV5C/Control_Merge.tif]

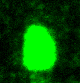

Supplement: Supplementary file 30 — Source Data For Expanded View [file 44318_2025_609_MOESM30_ESM.zip › SourceDataForExpandedView/FigureEV5/EV5C/Control_TDP-43.tif]

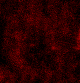

Supplement: Supplementary file 30 — Source Data For Expanded View [file 44318_2025_609_MOESM30_ESM.zip › SourceDataForExpandedView/FigureEV5/EV5C/Control_TIA1.tif]

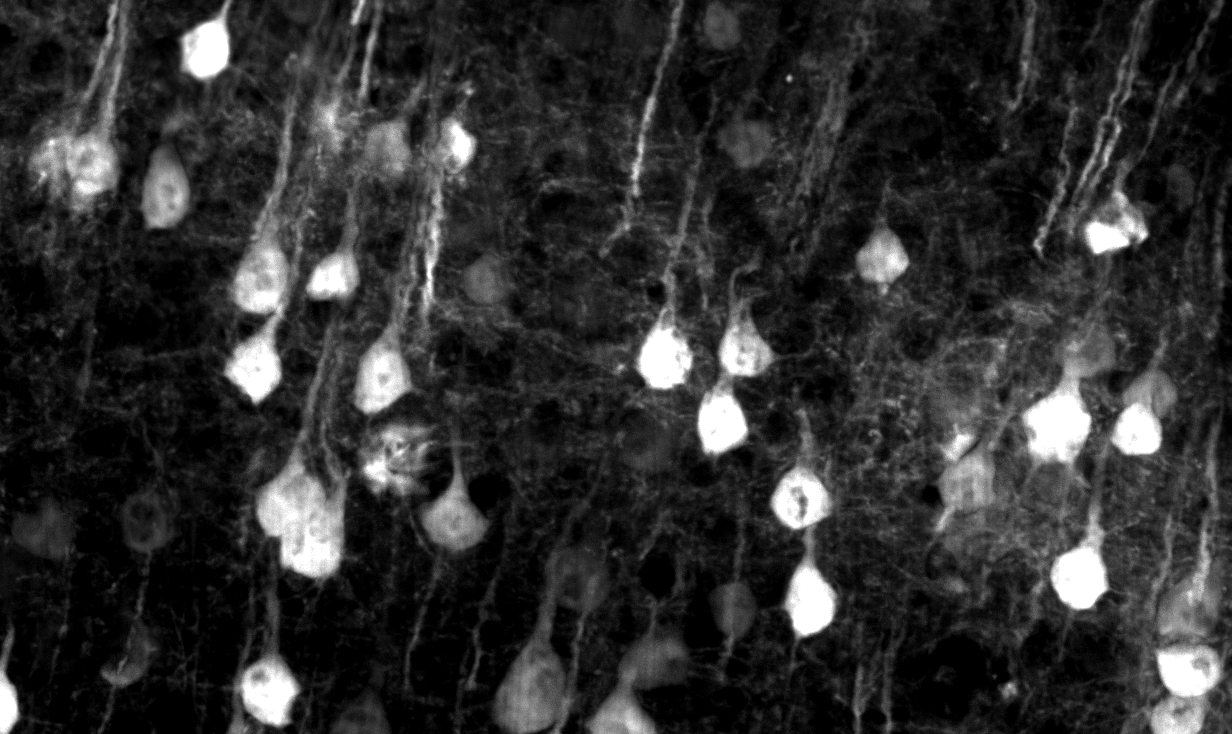

Supplement: Supplementary file 30 — Source Data For Expanded View [file 44318_2025_609_MOESM30_ESM.zip › SourceDataForExpandedView/FigureEV5/EV5C/shA7-3#_GFP.tif]

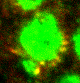

Supplement: Supplementary file 30 — Source Data For Expanded View [file 44318_2025_609_MOESM30_ESM.zip › SourceDataForExpandedView/FigureEV5/EV5C/shA7-3#_Merge.tif]

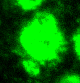

Supplement: Supplementary file 30 — Source Data For Expanded View [file 44318_2025_609_MOESM30_ESM.zip › SourceDataForExpandedView/FigureEV5/EV5C/shA7-3#_TDP-43.tif]

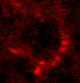

Supplement: Supplementary file 30 — Source Data For Expanded View [file 44318_2025_609_MOESM30_ESM.zip › SourceDataForExpandedView/FigureEV5/EV5C/shA7-3#_TIA1.tif]

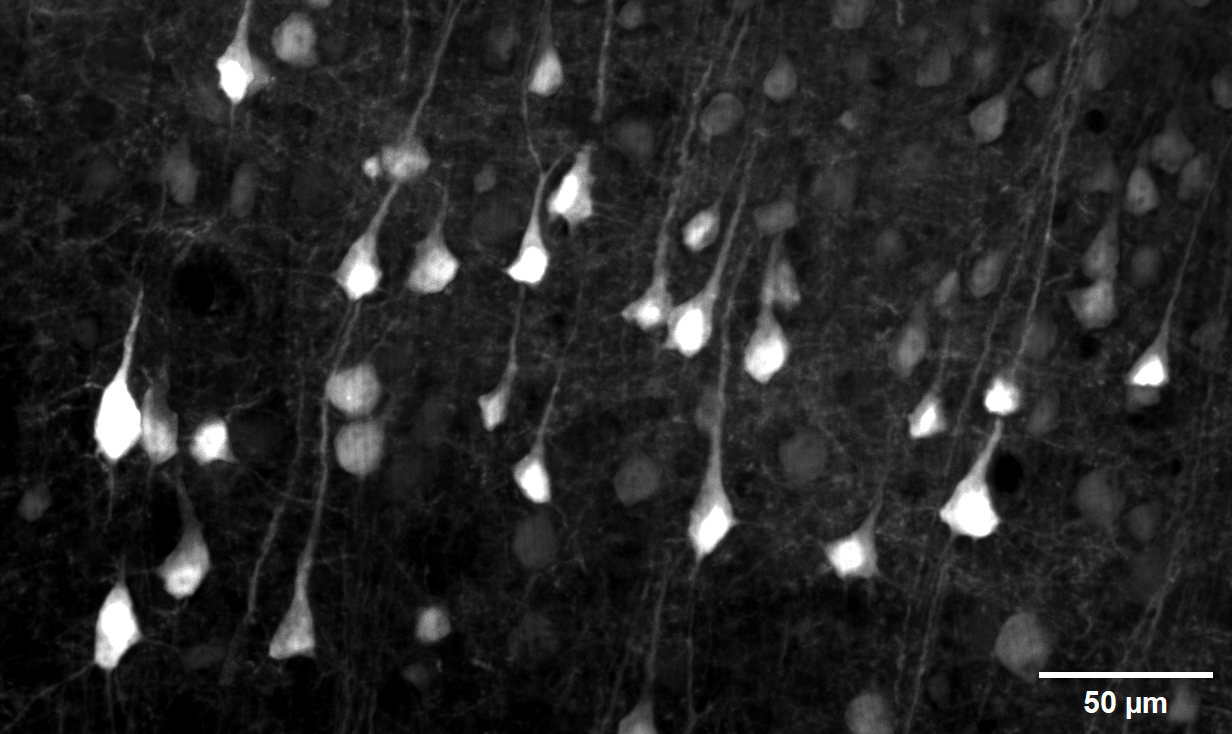

Supplement: Supplementary file 30 — Source Data For Expanded View [file 44318_2025_609_MOESM30_ESM.zip › SourceDataForExpandedView/FigureEV5/EV5C/shA7-4#_GFP.tif]

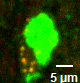

Supplement: Supplementary file 30 — Source Data For Expanded View [file 44318_2025_609_MOESM30_ESM.zip › SourceDataForExpandedView/FigureEV5/EV5C/shA7-4#_Merge.tif]

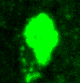

Supplement: Supplementary file 30 — Source Data For Expanded View [file 44318_2025_609_MOESM30_ESM.zip › SourceDataForExpandedView/FigureEV5/EV5C/shA7-4#_TDP-43.tif]

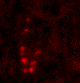

Supplement: Supplementary file 30 — Source Data For Expanded View [file 44318_2025_609_MOESM30_ESM.zip › SourceDataForExpandedView/FigureEV5/EV5C/shA7-4#_TIA1.tif]
