# Supplementary figures and images for: Annexin A7 enhances TIA1 axonal trafficking to counteract pathological aggregation in neurons (part 4 of 5)
Source: EMBO J. 2025 Nov 3;44(24):7477–512. doi: 10.1038/s44318-025-00609-8 (PMC12706091; doi:10.1038/s44318-025-00609-8)

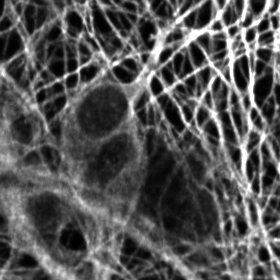

Supplement: Supplementary file 30 — Source Data For Expanded View [file 44318_2025_609_MOESM30_ESM.zip › SourceDataForExpandedView/Appendix figure/Appendix FigS1C/2#-B-Tub III.tif]

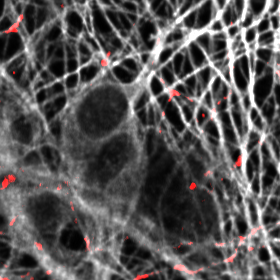

Supplement: Supplementary file 30 — Source Data For Expanded View [file 44318_2025_609_MOESM30_ESM.zip › SourceDataForExpandedView/Appendix figure/Appendix FigS1C/2#-Merge.tif]

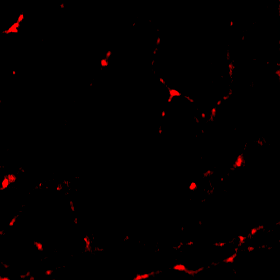

Supplement: Supplementary file 30 — Source Data For Expanded View [file 44318_2025_609_MOESM30_ESM.zip › SourceDataForExpandedView/Appendix figure/Appendix FigS1C/2#-TIA1-mCherry.tif]

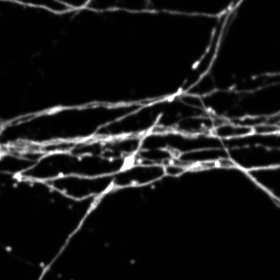

Supplement: Supplementary file 30 — Source Data For Expanded View [file 44318_2025_609_MOESM30_ESM.zip › SourceDataForExpandedView/Appendix figure/Appendix FigS1C/3#-B-Tub III.tif]

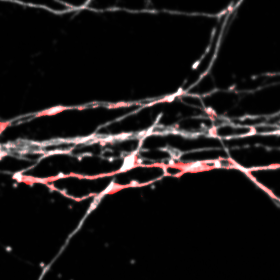

Supplement: Supplementary file 30 — Source Data For Expanded View [file 44318_2025_609_MOESM30_ESM.zip › SourceDataForExpandedView/Appendix figure/Appendix FigS1C/3#-Merge.tif]

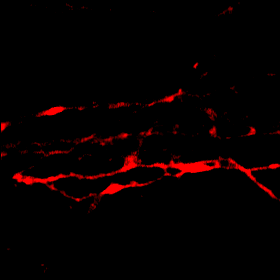

Supplement: Supplementary file 30 — Source Data For Expanded View [file 44318_2025_609_MOESM30_ESM.zip › SourceDataForExpandedView/Appendix figure/Appendix FigS1C/3#-TIA1-mCherry.tif]

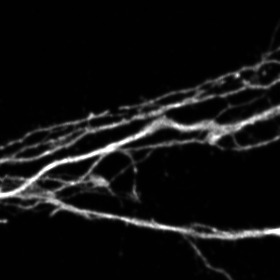

Supplement: Supplementary file 30 — Source Data For Expanded View [file 44318_2025_609_MOESM30_ESM.zip › SourceDataForExpandedView/Appendix figure/Appendix FigS1C/4#-B-Tub III.tif]

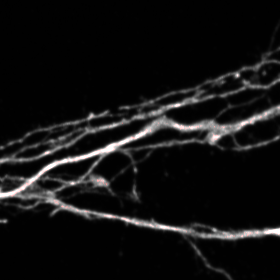

Supplement: Supplementary file 30 — Source Data For Expanded View [file 44318_2025_609_MOESM30_ESM.zip › SourceDataForExpandedView/Appendix figure/Appendix FigS1C/4#-Merge.tif]

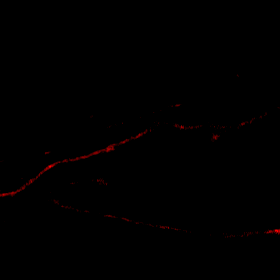

Supplement: Supplementary file 30 — Source Data For Expanded View [file 44318_2025_609_MOESM30_ESM.zip › SourceDataForExpandedView/Appendix figure/Appendix FigS1C/4#-TIA1-mCherry.tif]

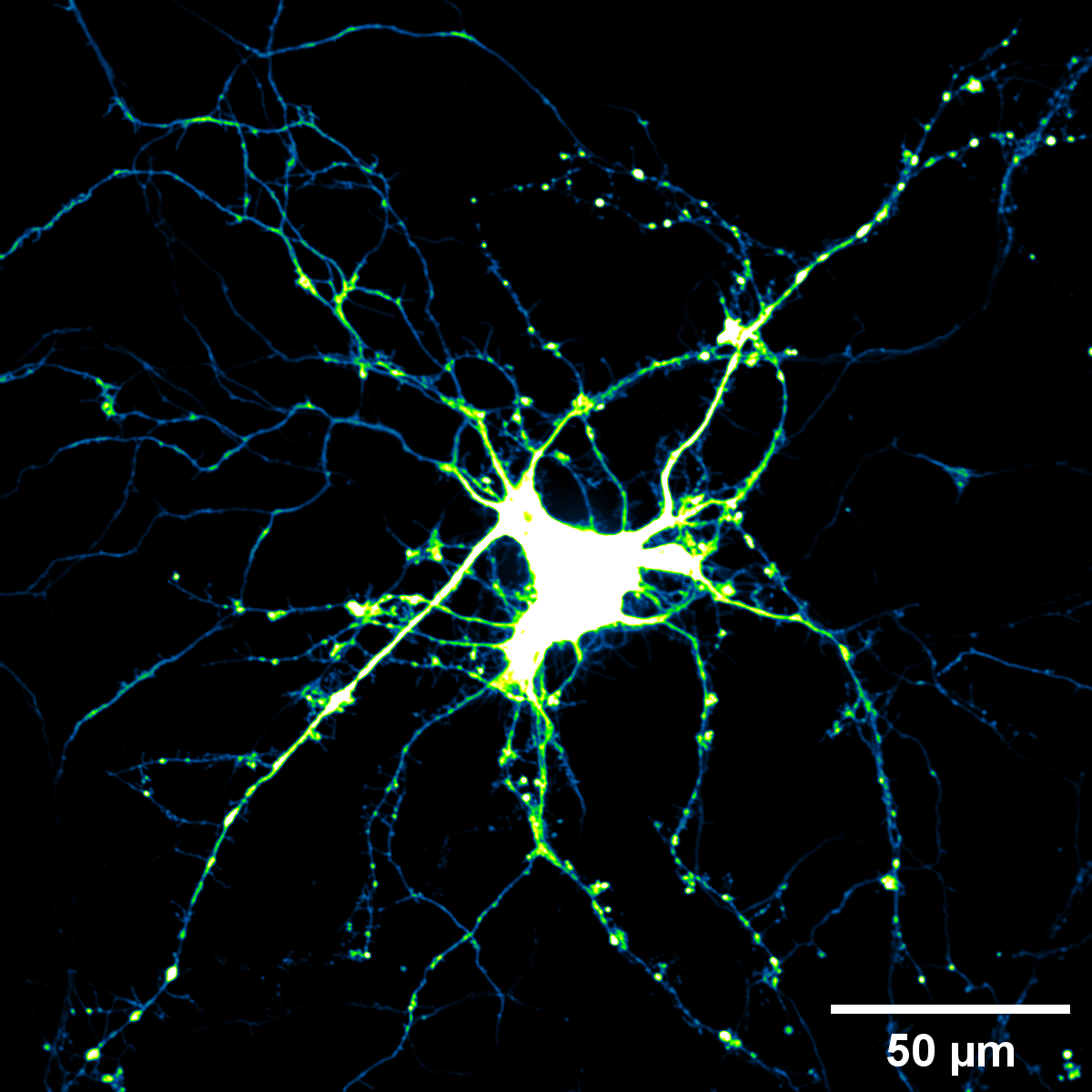

Supplement: Supplementary file 30 — Source Data For Expanded View [file 44318_2025_609_MOESM30_ESM.zip › SourceDataForExpandedView/Appendix figure/Appendix FigS3A/+ High K+ 10'.tif]

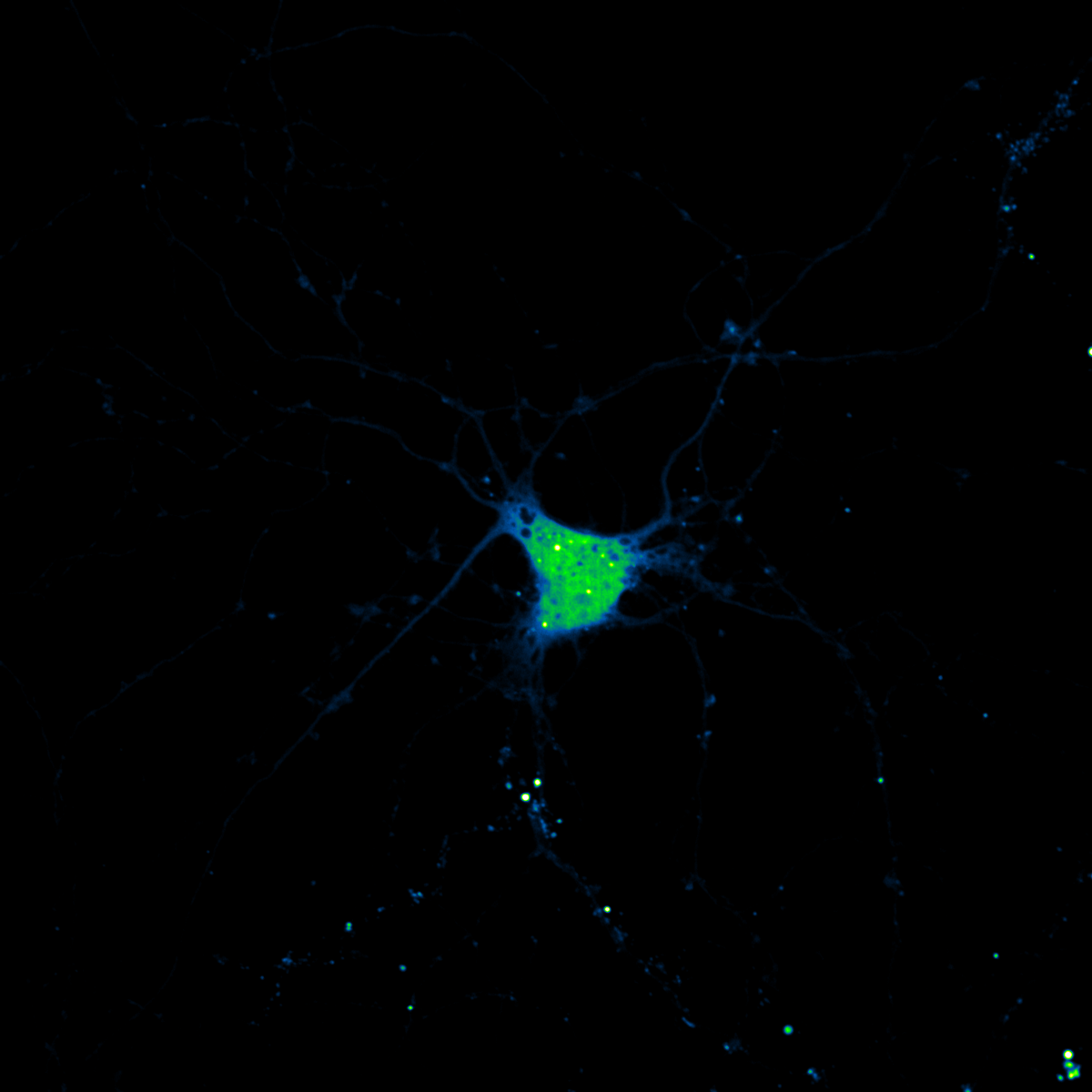

Supplement: Supplementary file 30 — Source Data For Expanded View [file 44318_2025_609_MOESM30_ESM.zip › SourceDataForExpandedView/Appendix figure/Appendix FigS3A/Before.tif]

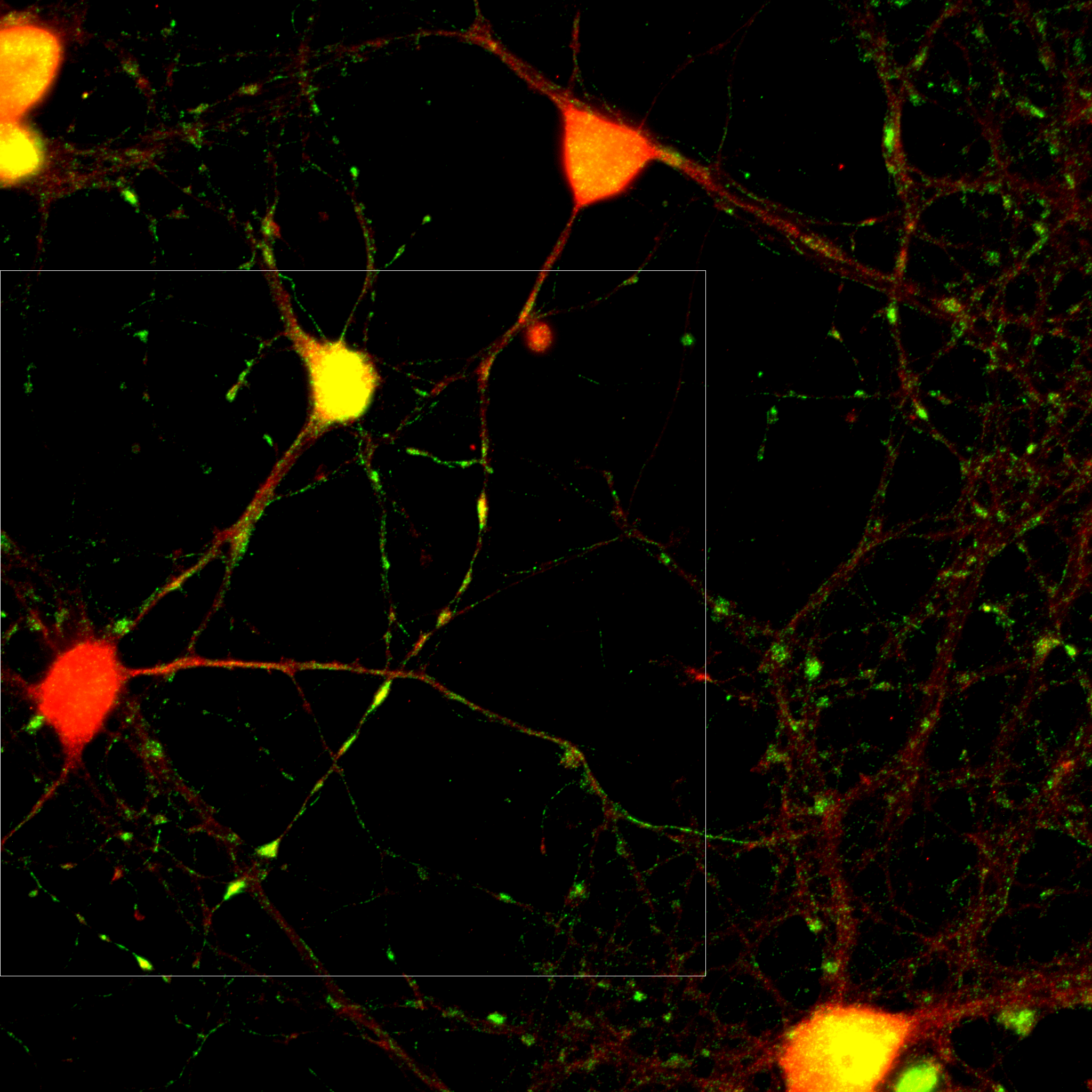

Supplement: Supplementary file 30 — Source Data For Expanded View [file 44318_2025_609_MOESM30_ESM.zip › SourceDataForExpandedView/Appendix figure/Appendix FigS3E/High K+.tif]

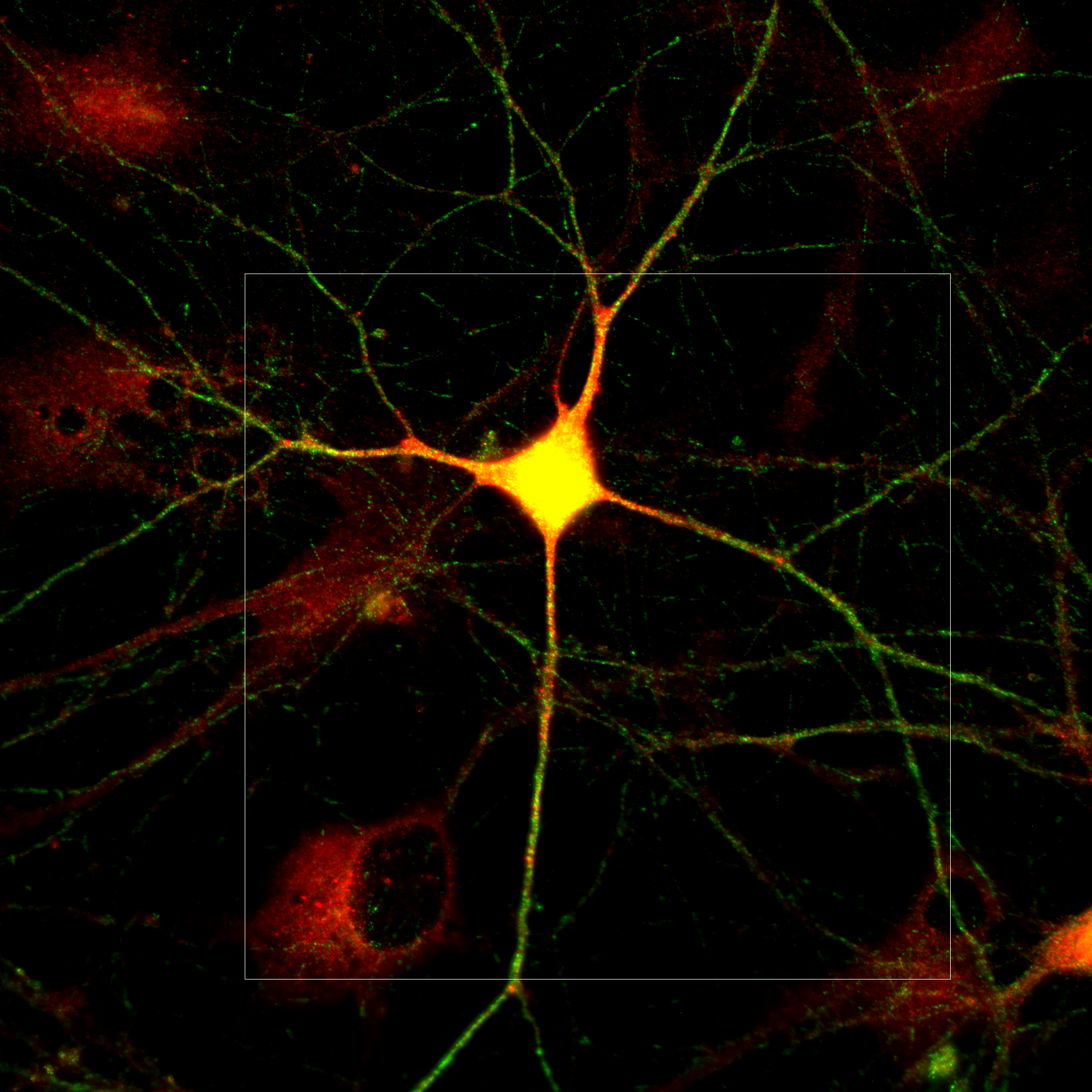

Supplement: Supplementary file 30 — Source Data For Expanded View [file 44318_2025_609_MOESM30_ESM.zip › SourceDataForExpandedView/Appendix figure/Appendix FigS3E/Low K+.tif]

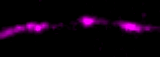

Supplement: Supplementary file 30 — Source Data For Expanded View [file 44318_2025_609_MOESM30_ESM.zip › SourceDataForExpandedView/FigureEV1/EV1A/1_Cy5-UTP+TIA1_Cy5-UTP.tif]

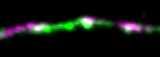

Supplement: Supplementary file 30 — Source Data For Expanded View [file 44318_2025_609_MOESM30_ESM.zip › SourceDataForExpandedView/FigureEV1/EV1A/1_Cy5-UTP+TIA1_Merge.tif]

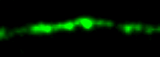

Supplement: Supplementary file 30 — Source Data For Expanded View [file 44318_2025_609_MOESM30_ESM.zip › SourceDataForExpandedView/FigureEV1/EV1A/1_Cy5-UTP+TIA1_TIA1.tif]

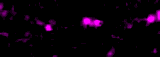

Supplement: Supplementary file 30 — Source Data For Expanded View [file 44318_2025_609_MOESM30_ESM.zip › SourceDataForExpandedView/FigureEV1/EV1A/2_Cy5-UTP+Stau1_Cy5-UTP.tif]

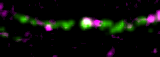

Supplement: Supplementary file 30 — Source Data For Expanded View [file 44318_2025_609_MOESM30_ESM.zip › SourceDataForExpandedView/FigureEV1/EV1A/2_Cy5-UTP+Stau1_Merge.tif]

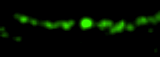

Supplement: Supplementary file 30 — Source Data For Expanded View [file 44318_2025_609_MOESM30_ESM.zip › SourceDataForExpandedView/FigureEV1/EV1A/2_Cy5-UTP+Stau1_Stau1.tif]

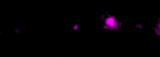

Supplement: Supplementary file 30 — Source Data For Expanded View [file 44318_2025_609_MOESM30_ESM.zip › SourceDataForExpandedView/FigureEV1/EV1A/3_Cy5-UTP+DCP1A_Cy5-UTP.tif]

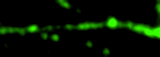

Supplement: Supplementary file 30 — Source Data For Expanded View [file 44318_2025_609_MOESM30_ESM.zip › SourceDataForExpandedView/FigureEV1/EV1A/3_Cy5-UTP+DCP1A_DCP1A.tif]

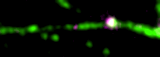

Supplement: Supplementary file 30 — Source Data For Expanded View [file 44318_2025_609_MOESM30_ESM.zip › SourceDataForExpandedView/FigureEV1/EV1A/3_Cy5-UTP+DCP1A_Merge.tif]

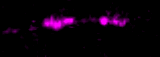

Supplement: Supplementary file 30 — Source Data For Expanded View [file 44318_2025_609_MOESM30_ESM.zip › SourceDataForExpandedView/FigureEV1/EV1A/4_Cy5-UTP+FMRP_Cy5-UTP.tif]

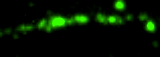

Supplement: Supplementary file 30 — Source Data For Expanded View [file 44318_2025_609_MOESM30_ESM.zip › SourceDataForExpandedView/FigureEV1/EV1A/4_Cy5-UTP+FMRP_FMRP.tif]

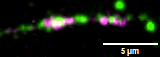

Supplement: Supplementary file 30 — Source Data For Expanded View [file 44318_2025_609_MOESM30_ESM.zip › SourceDataForExpandedView/FigureEV1/EV1A/4_Cy5-UTP+FMRP_Merge.tif]

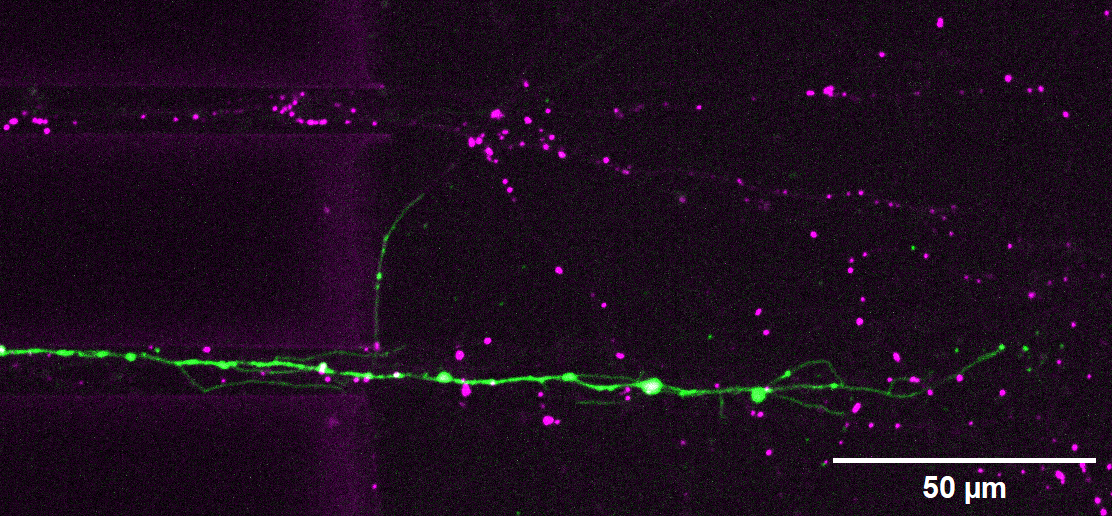

Supplement: Supplementary file 30 — Source Data For Expanded View [file 44318_2025_609_MOESM30_ESM.zip › SourceDataForExpandedView/FigureEV1/EV1B/EGFP-TIA1 + LysoTracker.tif]

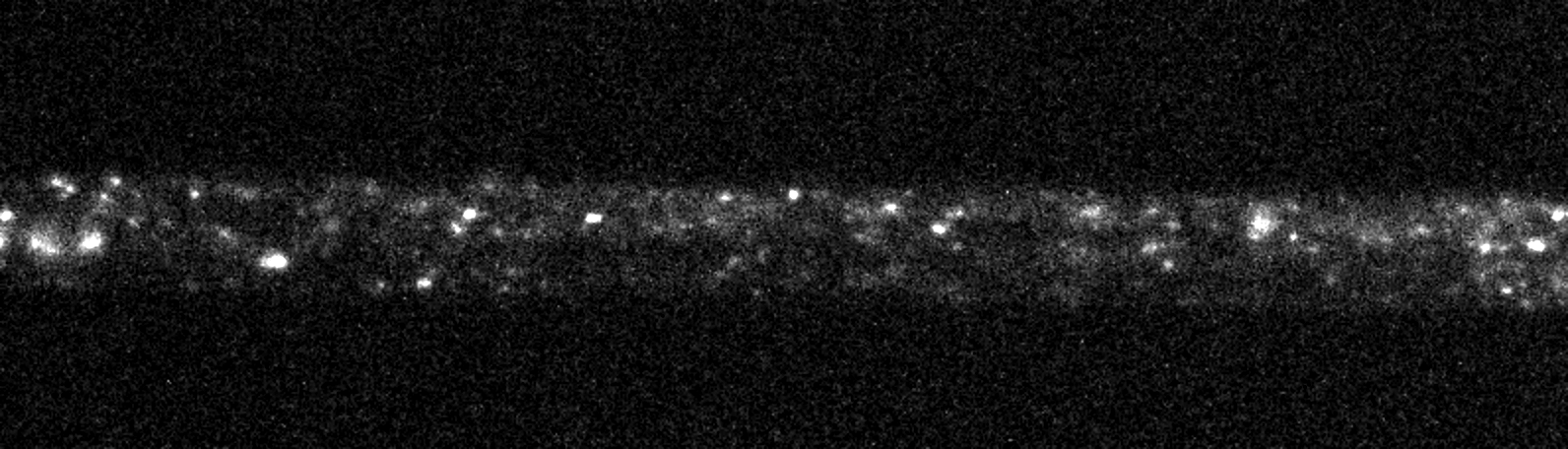

Supplement: Supplementary file 30 — Source Data For Expanded View [file 44318_2025_609_MOESM30_ESM.zip › SourceDataForExpandedView/FigureEV1/EV1C/0s_CTB.tif]

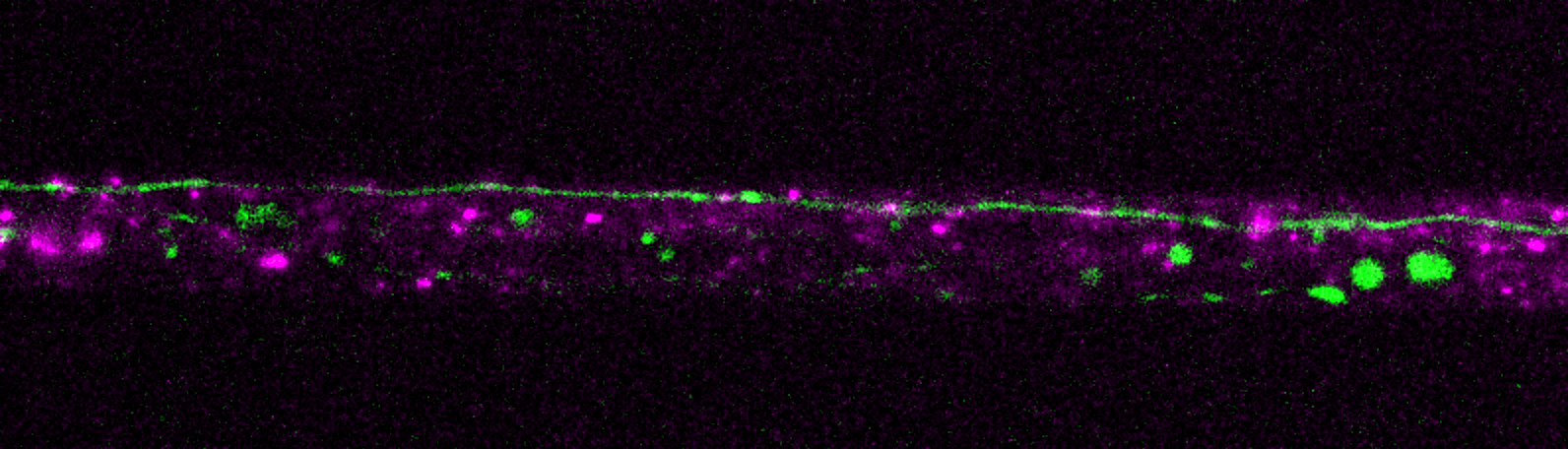

Supplement: Supplementary file 30 — Source Data For Expanded View [file 44318_2025_609_MOESM30_ESM.zip › SourceDataForExpandedView/FigureEV1/EV1C/0s_Merge.tif]

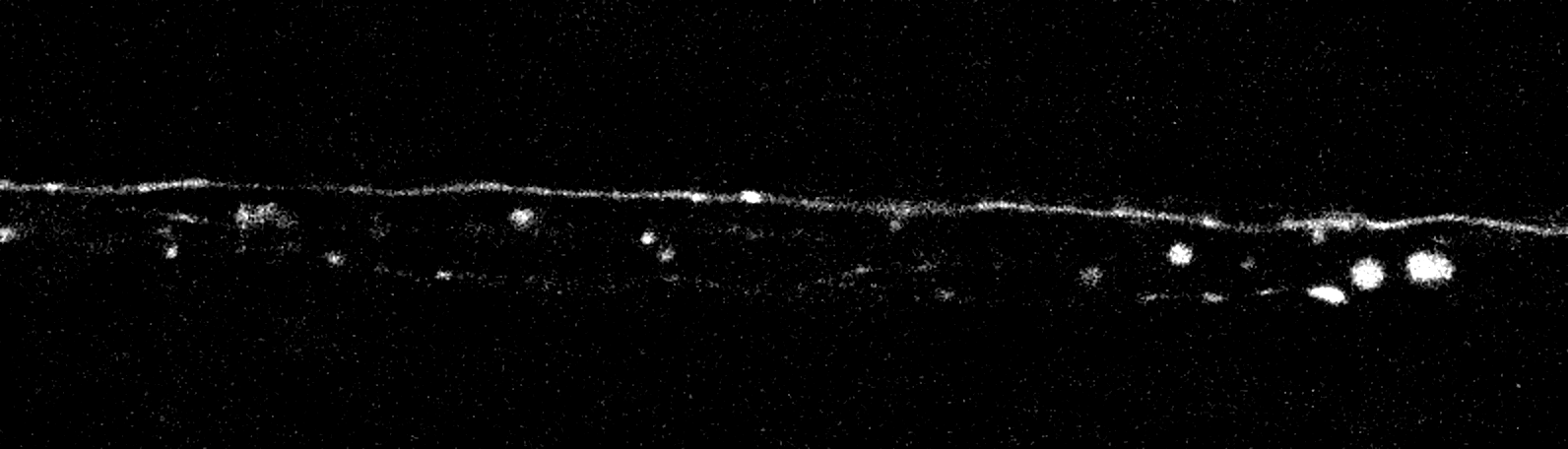

Supplement: Supplementary file 30 — Source Data For Expanded View [file 44318_2025_609_MOESM30_ESM.zip › SourceDataForExpandedView/FigureEV1/EV1C/0s_TIA1-mCherry.tif]

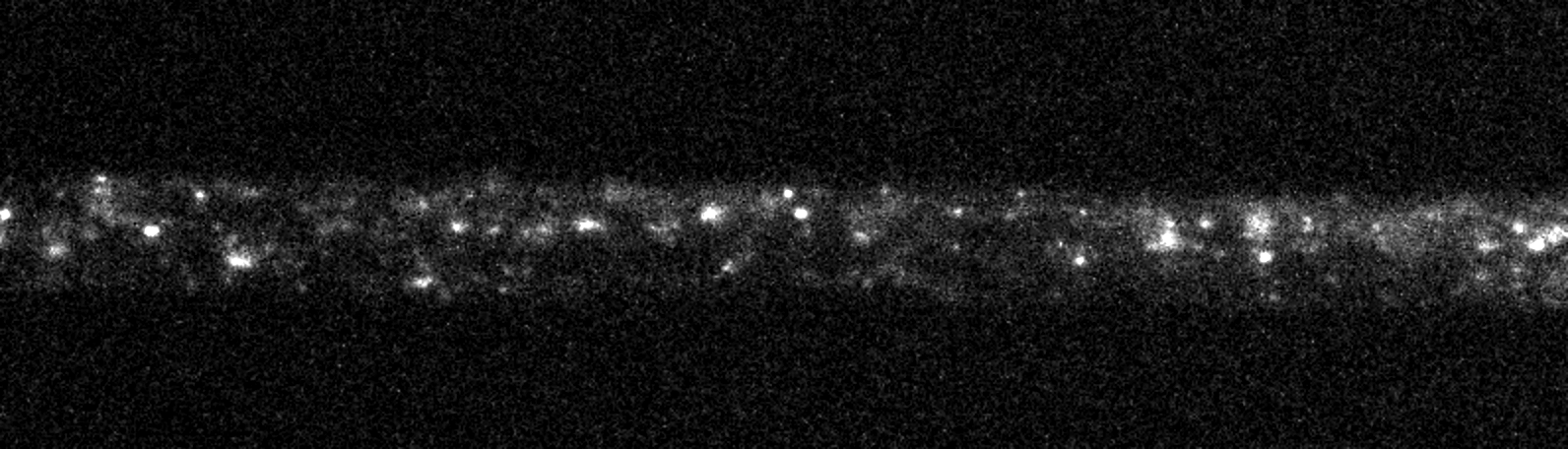

Supplement: Supplementary file 30 — Source Data For Expanded View [file 44318_2025_609_MOESM30_ESM.zip › SourceDataForExpandedView/FigureEV1/EV1C/100s_CTB.tif]

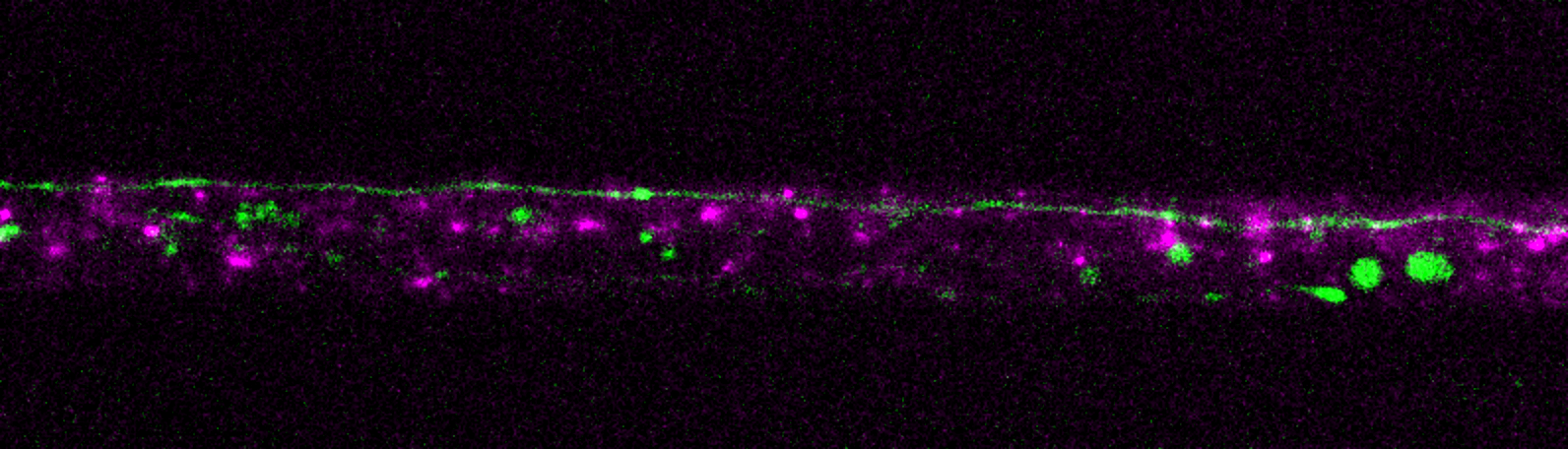

Supplement: Supplementary file 30 — Source Data For Expanded View [file 44318_2025_609_MOESM30_ESM.zip › SourceDataForExpandedView/FigureEV1/EV1C/100s_Merge.tif]

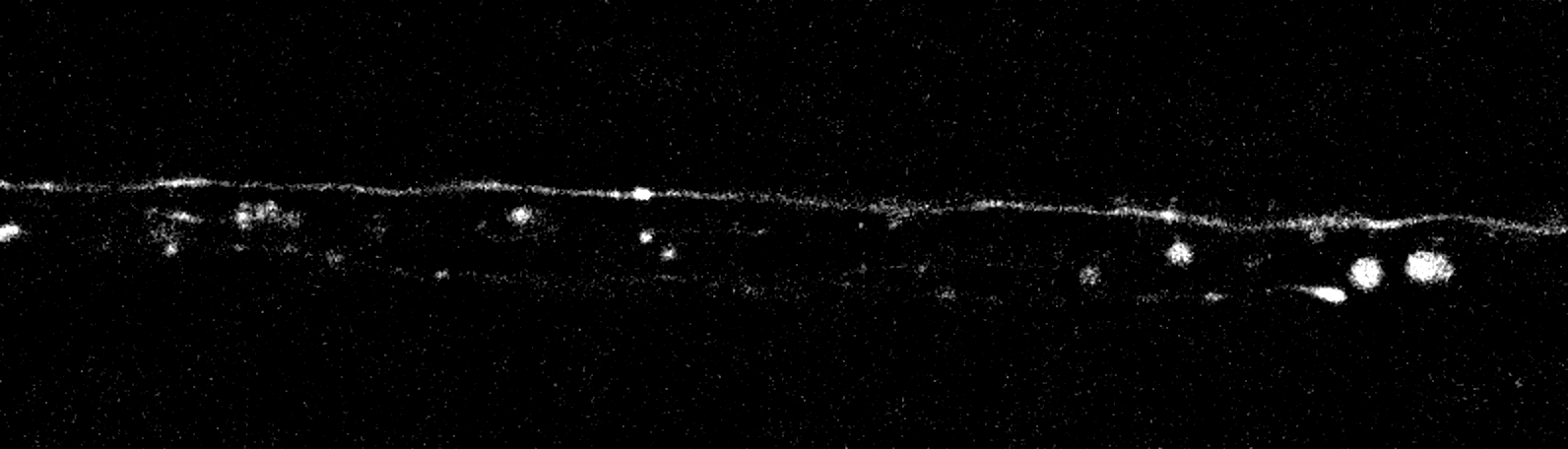

Supplement: Supplementary file 30 — Source Data For Expanded View [file 44318_2025_609_MOESM30_ESM.zip › SourceDataForExpandedView/FigureEV1/EV1C/100s_TIA1-mCherry.tif]

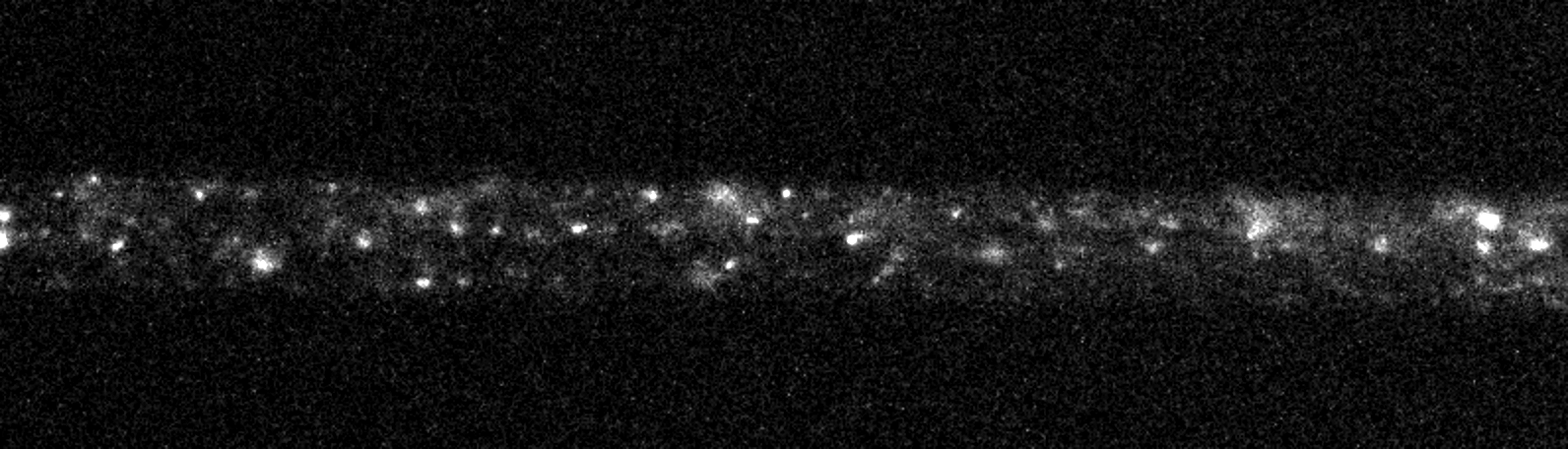

Supplement: Supplementary file 30 — Source Data For Expanded View [file 44318_2025_609_MOESM30_ESM.zip › SourceDataForExpandedView/FigureEV1/EV1C/200s_CTB.tif]

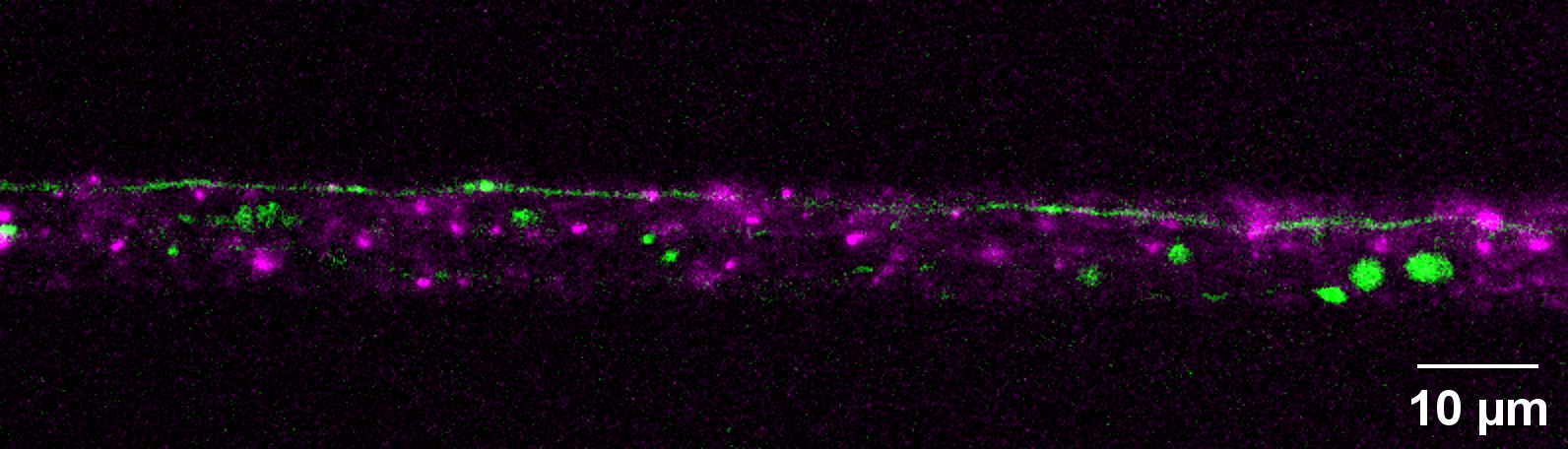

Supplement: Supplementary file 30 — Source Data For Expanded View [file 44318_2025_609_MOESM30_ESM.zip › SourceDataForExpandedView/FigureEV1/EV1C/200s_Merge.tif]

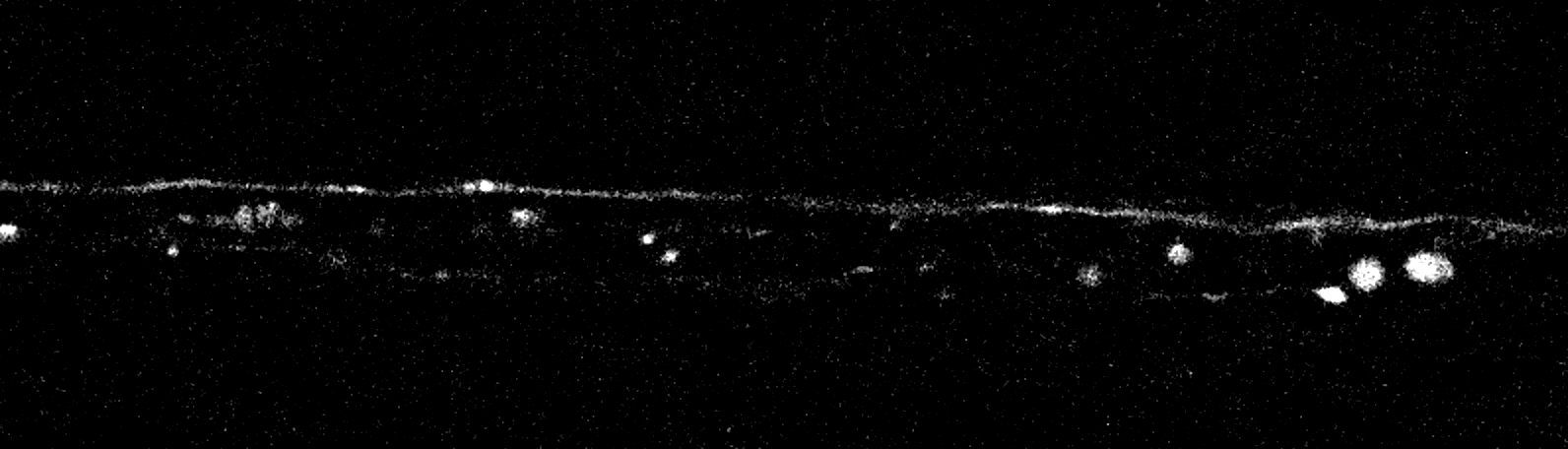

Supplement: Supplementary file 30 — Source Data For Expanded View [file 44318_2025_609_MOESM30_ESM.zip › SourceDataForExpandedView/FigureEV1/EV1C/200s_TIA1-mCherry.tif]

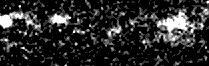

Supplement: Supplementary file 30 — Source Data For Expanded View [file 44318_2025_609_MOESM30_ESM.zip › SourceDataForExpandedView/FigureEV1/EV1D/0s_BoNT A-Hc.tif]

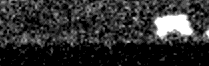

Supplement: Supplementary file 30 — Source Data For Expanded View [file 44318_2025_609_MOESM30_ESM.zip › SourceDataForExpandedView/FigureEV1/EV1D/0s_EGFP-TIA1.tif]

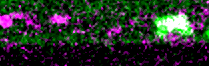

Supplement: Supplementary file 30 — Source Data For Expanded View [file 44318_2025_609_MOESM30_ESM.zip › SourceDataForExpandedView/FigureEV1/EV1D/0s_Merge.tif]

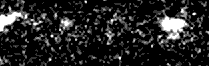

Supplement: Supplementary file 30 — Source Data For Expanded View [file 44318_2025_609_MOESM30_ESM.zip › SourceDataForExpandedView/FigureEV1/EV1D/24s_BoNT A-Hc.tif]

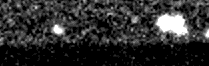

Supplement: Supplementary file 30 — Source Data For Expanded View [file 44318_2025_609_MOESM30_ESM.zip › SourceDataForExpandedView/FigureEV1/EV1D/24s_EGFP-TIA1.tif]

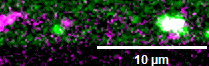

Supplement: Supplementary file 30 — Source Data For Expanded View [file 44318_2025_609_MOESM30_ESM.zip › SourceDataForExpandedView/FigureEV1/EV1D/24s_Merge.tif]

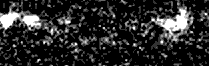

Supplement: Supplementary file 30 — Source Data For Expanded View [file 44318_2025_609_MOESM30_ESM.zip › SourceDataForExpandedView/FigureEV1/EV1D/8s_BoNT A-Hc.tif]

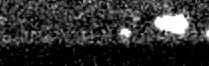

Supplement: Supplementary file 30 — Source Data For Expanded View [file 44318_2025_609_MOESM30_ESM.zip › SourceDataForExpandedView/FigureEV1/EV1D/8s_EGFP-TIA1.tif]

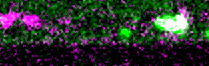

Supplement: Supplementary file 30 — Source Data For Expanded View [file 44318_2025_609_MOESM30_ESM.zip › SourceDataForExpandedView/FigureEV1/EV1D/8s_Merge.tif]

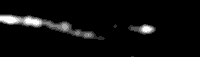

Supplement: Supplementary file 30 — Source Data For Expanded View [file 44318_2025_609_MOESM30_ESM.zip › SourceDataForExpandedView/FigureEV1/EV1E/0s_EGFP-TIA1.tif]

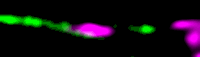

Supplement: Supplementary file 30 — Source Data For Expanded View [file 44318_2025_609_MOESM30_ESM.zip › SourceDataForExpandedView/FigureEV1/EV1E/0s_Merge.tif]

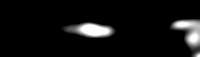

Supplement: Supplementary file 30 — Source Data For Expanded View [file 44318_2025_609_MOESM30_ESM.zip › SourceDataForExpandedView/FigureEV1/EV1E/0s_MitoTracker.tif]

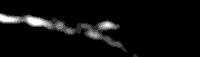

Supplement: Supplementary file 30 — Source Data For Expanded View [file 44318_2025_609_MOESM30_ESM.zip › SourceDataForExpandedView/FigureEV1/EV1E/136s_EGFP-TIA1.tif]

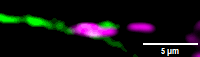

Supplement: Supplementary file 30 — Source Data For Expanded View [file 44318_2025_609_MOESM30_ESM.zip › SourceDataForExpandedView/FigureEV1/EV1E/136s_Merge.tif]

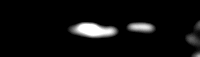

Supplement: Supplementary file 30 — Source Data For Expanded View [file 44318_2025_609_MOESM30_ESM.zip › SourceDataForExpandedView/FigureEV1/EV1E/136s_MitoTracker.tif]

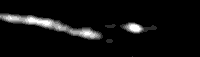

Supplement: Supplementary file 30 — Source Data For Expanded View [file 44318_2025_609_MOESM30_ESM.zip › SourceDataForExpandedView/FigureEV1/EV1E/68s_EGFP-TIA1.tif]

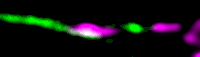

Supplement: Supplementary file 30 — Source Data For Expanded View [file 44318_2025_609_MOESM30_ESM.zip › SourceDataForExpandedView/FigureEV1/EV1E/68s_Merge.tif]

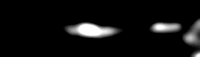

Supplement: Supplementary file 30 — Source Data For Expanded View [file 44318_2025_609_MOESM30_ESM.zip › SourceDataForExpandedView/FigureEV1/EV1E/68s_MitoTracker.tif]

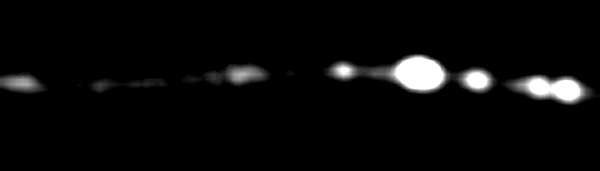

Supplement: Supplementary file 30 — Source Data For Expanded View [file 44318_2025_609_MOESM30_ESM.zip › SourceDataForExpandedView/FigureEV1/EV1F/0s_EGFP-Rab5.tif]

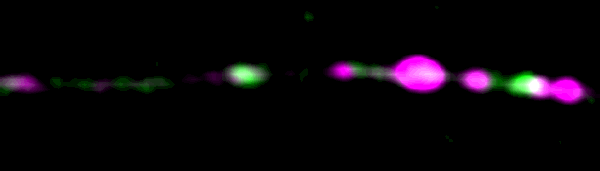

Supplement: Supplementary file 30 — Source Data For Expanded View [file 44318_2025_609_MOESM30_ESM.zip › SourceDataForExpandedView/FigureEV1/EV1F/0s_Merge.tif]

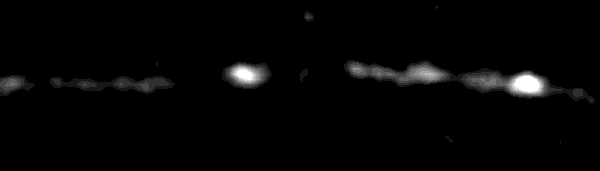

Supplement: Supplementary file 30 — Source Data For Expanded View [file 44318_2025_609_MOESM30_ESM.zip › SourceDataForExpandedView/FigureEV1/EV1F/0s_TIA1-mCherry.tif]

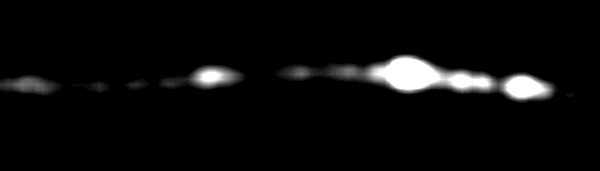

Supplement: Supplementary file 30 — Source Data For Expanded View [file 44318_2025_609_MOESM30_ESM.zip › SourceDataForExpandedView/FigureEV1/EV1F/36s_EGFP-Rab5.tif]

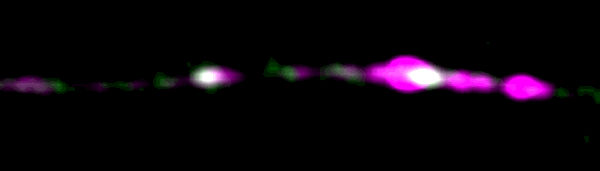

Supplement: Supplementary file 30 — Source Data For Expanded View [file 44318_2025_609_MOESM30_ESM.zip › SourceDataForExpandedView/FigureEV1/EV1F/36s_Merge.tif]

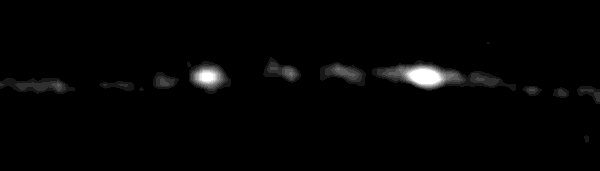

Supplement: Supplementary file 30 — Source Data For Expanded View [file 44318_2025_609_MOESM30_ESM.zip › SourceDataForExpandedView/FigureEV1/EV1F/36s_TIA1-mCherry.tif]

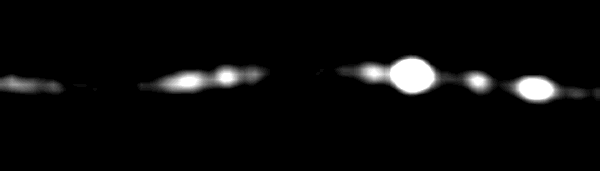

Supplement: Supplementary file 30 — Source Data For Expanded View [file 44318_2025_609_MOESM30_ESM.zip › SourceDataForExpandedView/FigureEV1/EV1F/72s_EGFP-Rab5.tif]

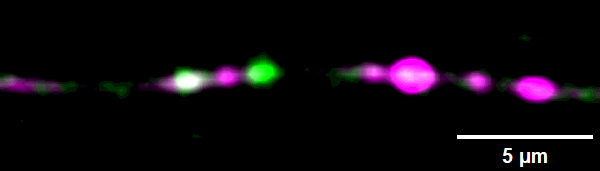

Supplement: Supplementary file 30 — Source Data For Expanded View [file 44318_2025_609_MOESM30_ESM.zip › SourceDataForExpandedView/FigureEV1/EV1F/72s_Merge.tif]

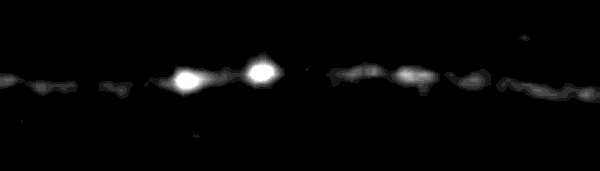

Supplement: Supplementary file 30 — Source Data For Expanded View [file 44318_2025_609_MOESM30_ESM.zip › SourceDataForExpandedView/FigureEV1/EV1F/72s_TIA1-mCherry.tif]

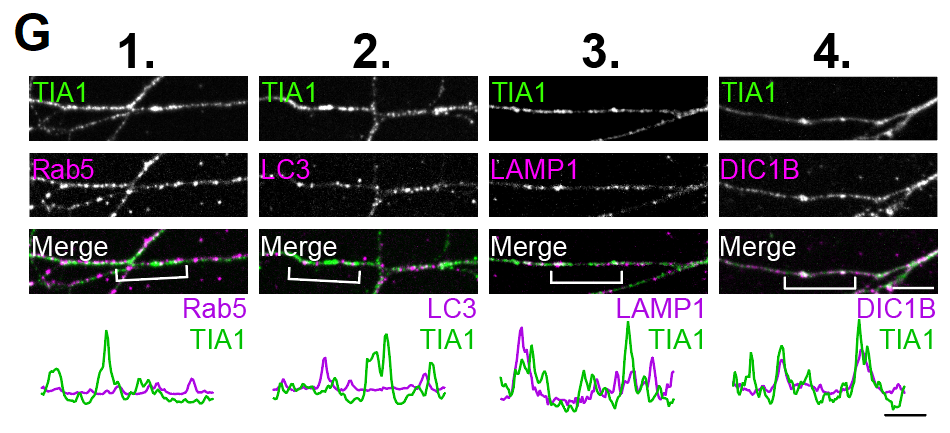

Supplement: Supplementary file 30 — Source Data For Expanded View [file 44318_2025_609_MOESM30_ESM.zip › SourceDataForExpandedView/FigureEV1/EV1G/0-EV1G.tif]

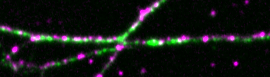

Supplement: Supplementary file 30 — Source Data For Expanded View [file 44318_2025_609_MOESM30_ESM.zip › SourceDataForExpandedView/FigureEV1/EV1G/1-Merge.tif]

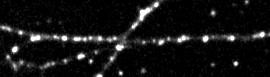

Supplement: Supplementary file 30 — Source Data For Expanded View [file 44318_2025_609_MOESM30_ESM.zip › SourceDataForExpandedView/FigureEV1/EV1G/1-Rab5.tif]

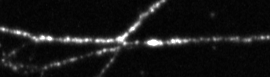

Supplement: Supplementary file 30 — Source Data For Expanded View [file 44318_2025_609_MOESM30_ESM.zip › SourceDataForExpandedView/FigureEV1/EV1G/1-TIA1.tif]

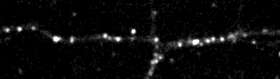

Supplement: Supplementary file 30 — Source Data For Expanded View [file 44318_2025_609_MOESM30_ESM.zip › SourceDataForExpandedView/FigureEV1/EV1G/2-LC3.tif]

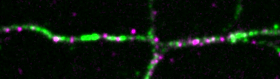

Supplement: Supplementary file 30 — Source Data For Expanded View [file 44318_2025_609_MOESM30_ESM.zip › SourceDataForExpandedView/FigureEV1/EV1G/2-Merge.tif]

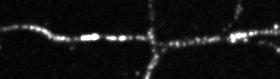

Supplement: Supplementary file 30 — Source Data For Expanded View [file 44318_2025_609_MOESM30_ESM.zip › SourceDataForExpandedView/FigureEV1/EV1G/2-TIA1.tif]

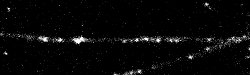

Supplement: Supplementary file 30 — Source Data For Expanded View [file 44318_2025_609_MOESM30_ESM.zip › SourceDataForExpandedView/FigureEV1/EV1G/3-LC3.tif]

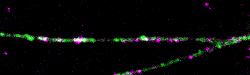

Supplement: Supplementary file 30 — Source Data For Expanded View [file 44318_2025_609_MOESM30_ESM.zip › SourceDataForExpandedView/FigureEV1/EV1G/3-Merge.tif]

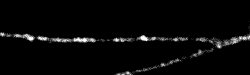

Supplement: Supplementary file 30 — Source Data For Expanded View [file 44318_2025_609_MOESM30_ESM.zip › SourceDataForExpandedView/FigureEV1/EV1G/3-TIA1.tif]

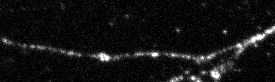

Supplement: Supplementary file 30 — Source Data For Expanded View [file 44318_2025_609_MOESM30_ESM.zip › SourceDataForExpandedView/FigureEV1/EV1G/4-DIC1B.tif]

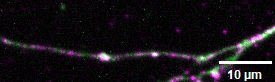

Supplement: Supplementary file 30 — Source Data For Expanded View [file 44318_2025_609_MOESM30_ESM.zip › SourceDataForExpandedView/FigureEV1/EV1G/4-Merge.tif]

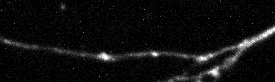

Supplement: Supplementary file 30 — Source Data For Expanded View [file 44318_2025_609_MOESM30_ESM.zip › SourceDataForExpandedView/FigureEV1/EV1G/4-TIA1.tif]

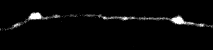

Supplement: Supplementary file 30 — Source Data For Expanded View [file 44318_2025_609_MOESM30_ESM.zip › SourceDataForExpandedView/FigureEV1/EV1I/+Nocodazole_0s.tif]

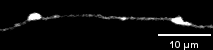

Supplement: Supplementary file 30 — Source Data For Expanded View [file 44318_2025_609_MOESM30_ESM.zip › SourceDataForExpandedView/FigureEV1/EV1I/+Nocodazole_144s.tif]

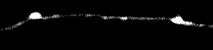

Supplement: Supplementary file 30 — Source Data For Expanded View [file 44318_2025_609_MOESM30_ESM.zip › SourceDataForExpandedView/FigureEV1/EV1I/+Nocodazole_48s.tif]

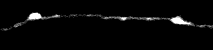

Supplement: Supplementary file 30 — Source Data For Expanded View [file 44318_2025_609_MOESM30_ESM.zip › SourceDataForExpandedView/FigureEV1/EV1I/+Nocodazole_96s.tif]

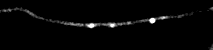

Supplement: Supplementary file 30 — Source Data For Expanded View [file 44318_2025_609_MOESM30_ESM.zip › SourceDataForExpandedView/FigureEV1/EV1I/Control_0s.tif]

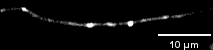

Supplement: Supplementary file 30 — Source Data For Expanded View [file 44318_2025_609_MOESM30_ESM.zip › SourceDataForExpandedView/FigureEV1/EV1I/Control_144s.tif]

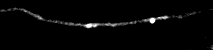

Supplement: Supplementary file 30 — Source Data For Expanded View [file 44318_2025_609_MOESM30_ESM.zip › SourceDataForExpandedView/FigureEV1/EV1I/Control_48s.tif]

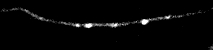

Supplement: Supplementary file 30 — Source Data For Expanded View [file 44318_2025_609_MOESM30_ESM.zip › SourceDataForExpandedView/FigureEV1/EV1I/Control_96s.tif]

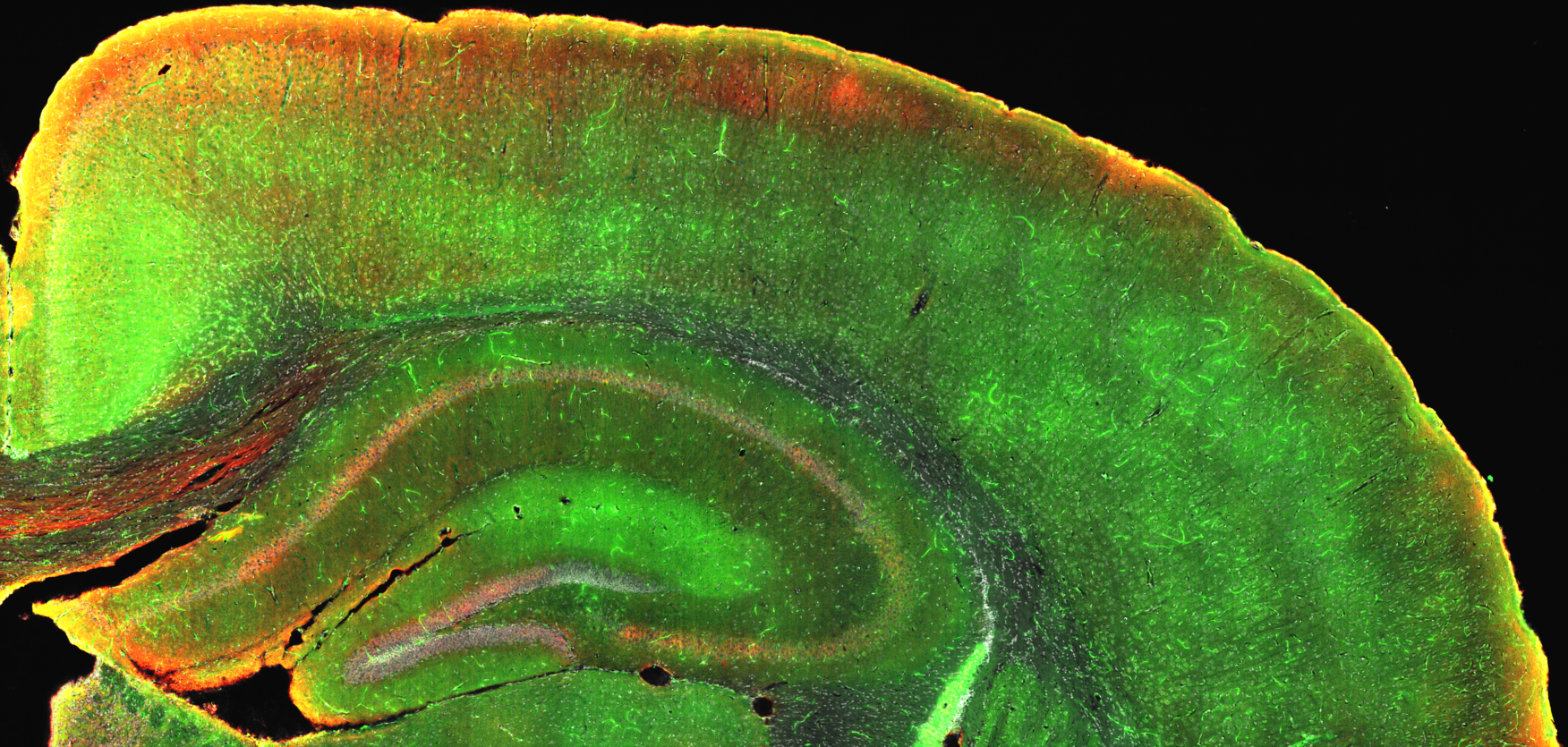

Supplement: Supplementary file 30 — Source Data For Expanded View [file 44318_2025_609_MOESM30_ESM.zip › SourceDataForExpandedView/FigureEV2/EV2B/ANXA7-TIA1-DAPI/ANXA7-TIA1-DAPI_large image.tif]

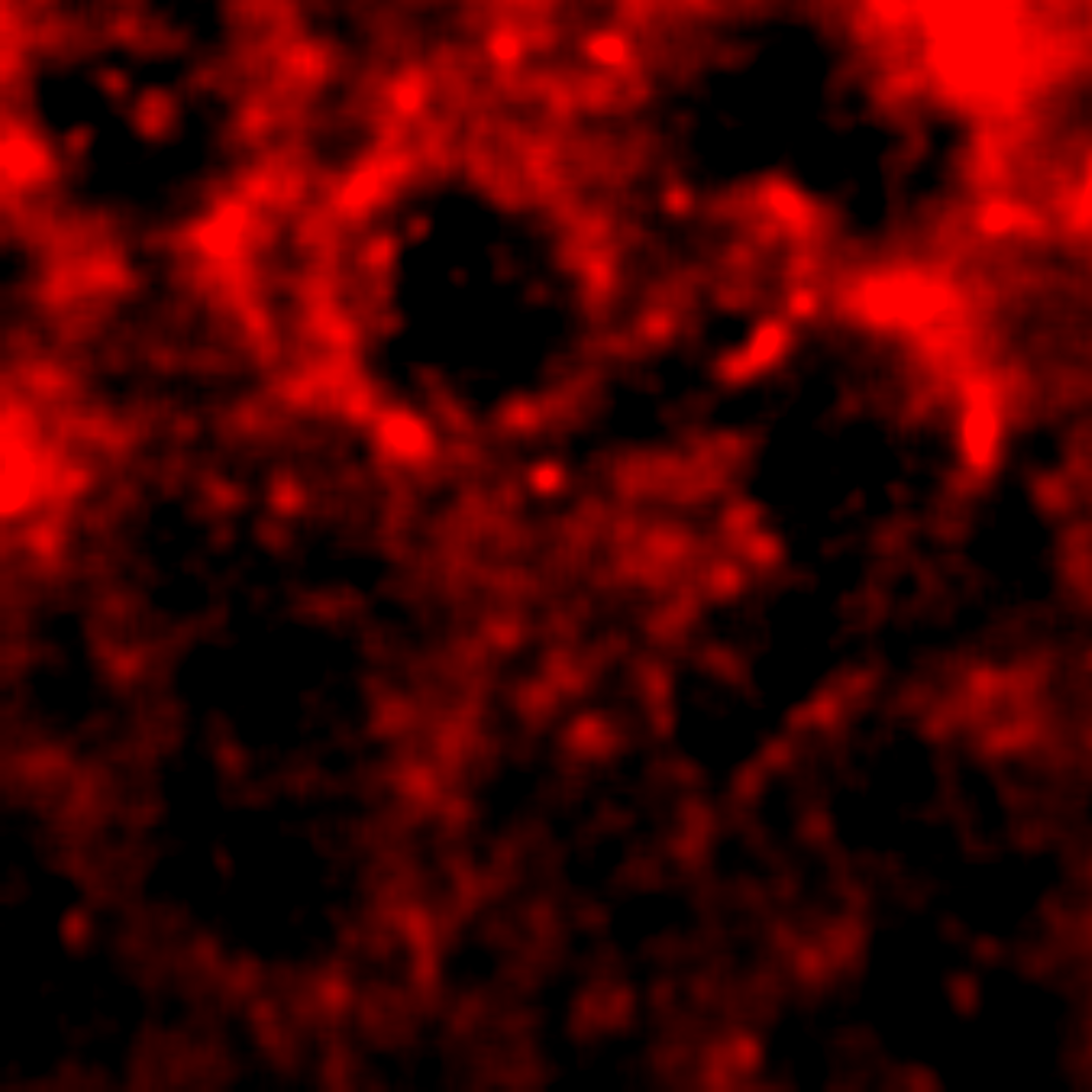

Supplement: Supplementary file 30 — Source Data For Expanded View [file 44318_2025_609_MOESM30_ESM.zip › SourceDataForExpandedView/FigureEV2/EV2B/ANXA7-TIA1-DAPI/CA3 ANXA7.tif]

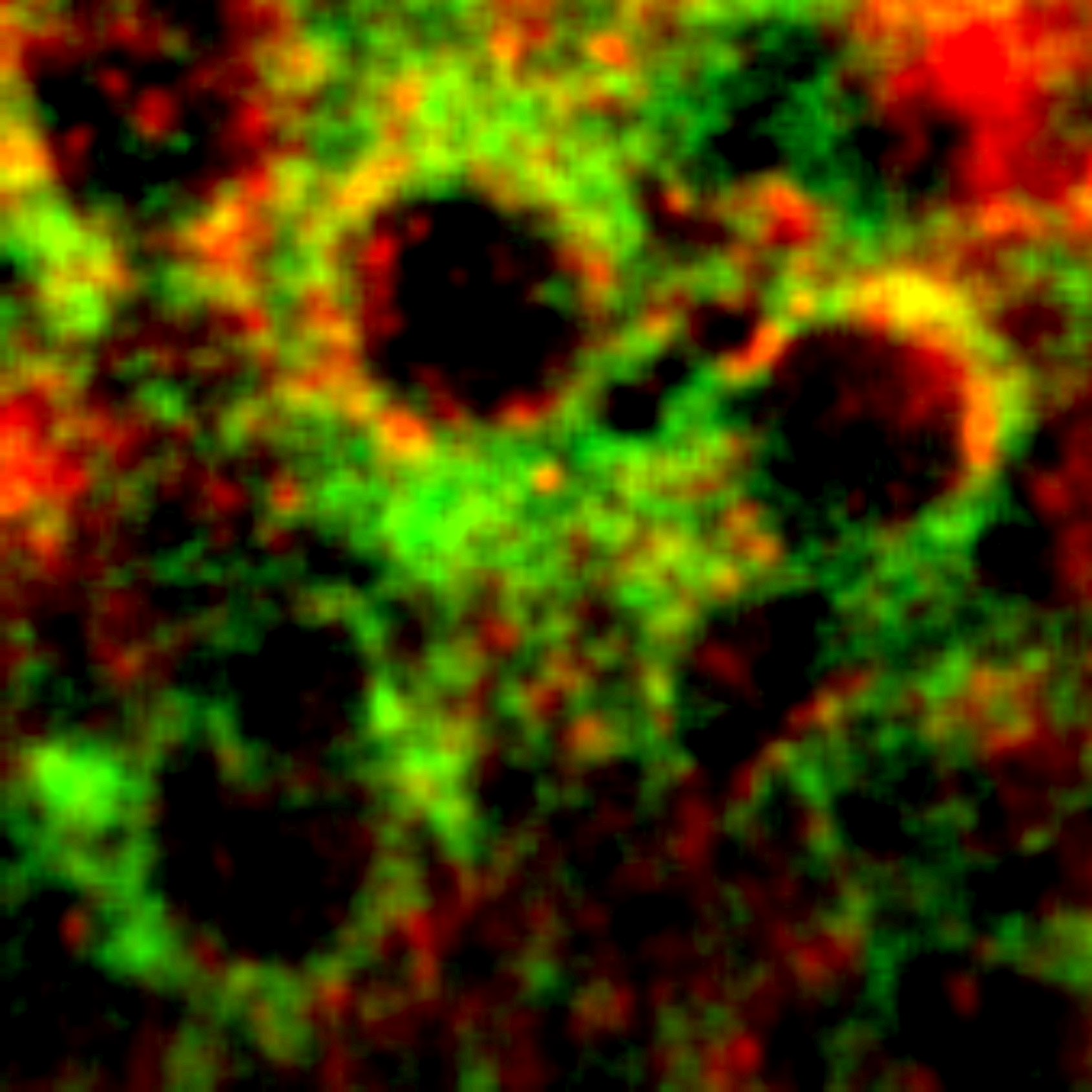

Supplement: Supplementary file 30 — Source Data For Expanded View [file 44318_2025_609_MOESM30_ESM.zip › SourceDataForExpandedView/FigureEV2/EV2B/ANXA7-TIA1-DAPI/CA3 Merge.tif]

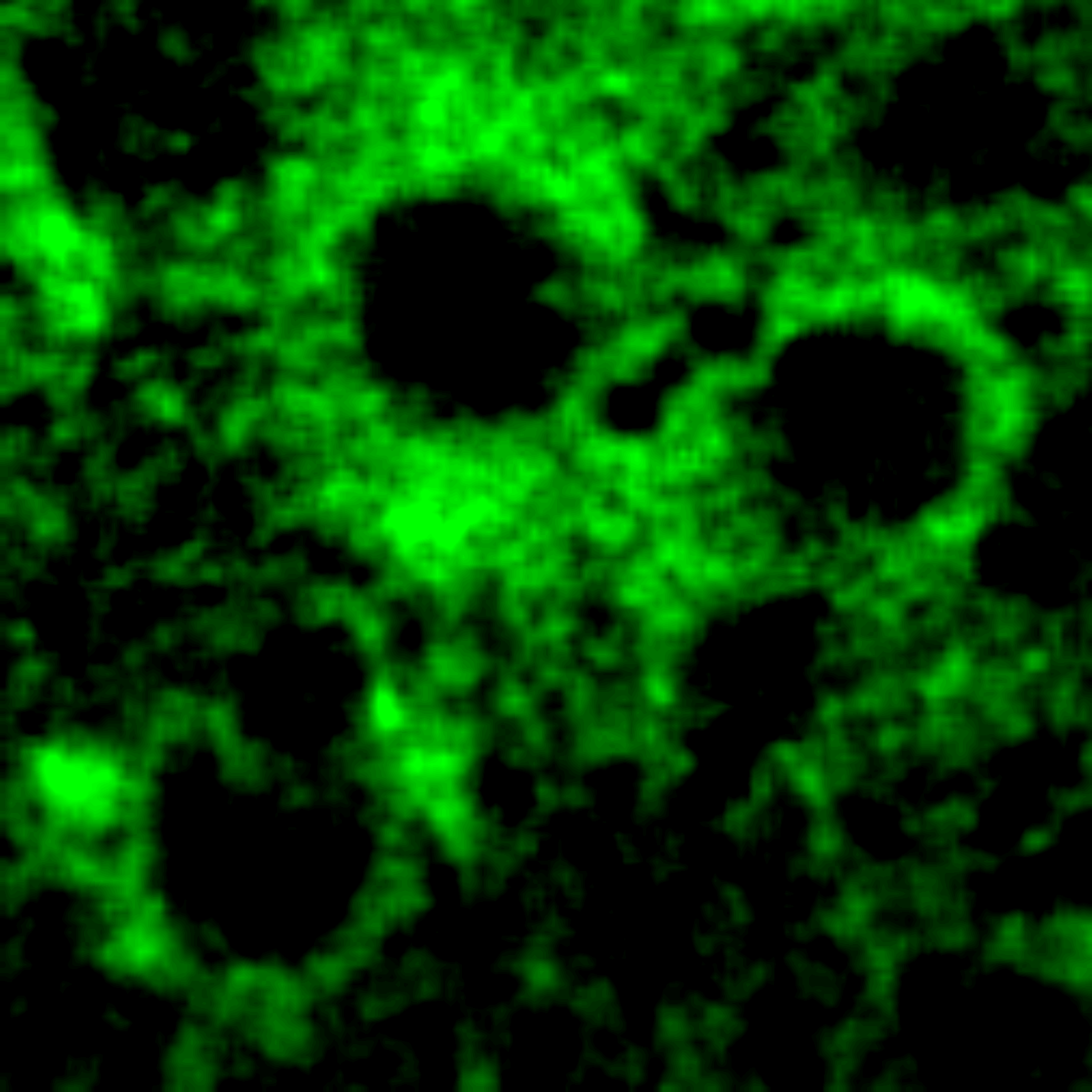

Supplement: Supplementary file 30 — Source Data For Expanded View [file 44318_2025_609_MOESM30_ESM.zip › SourceDataForExpandedView/FigureEV2/EV2B/ANXA7-TIA1-DAPI/CA3 TIA1.tif]

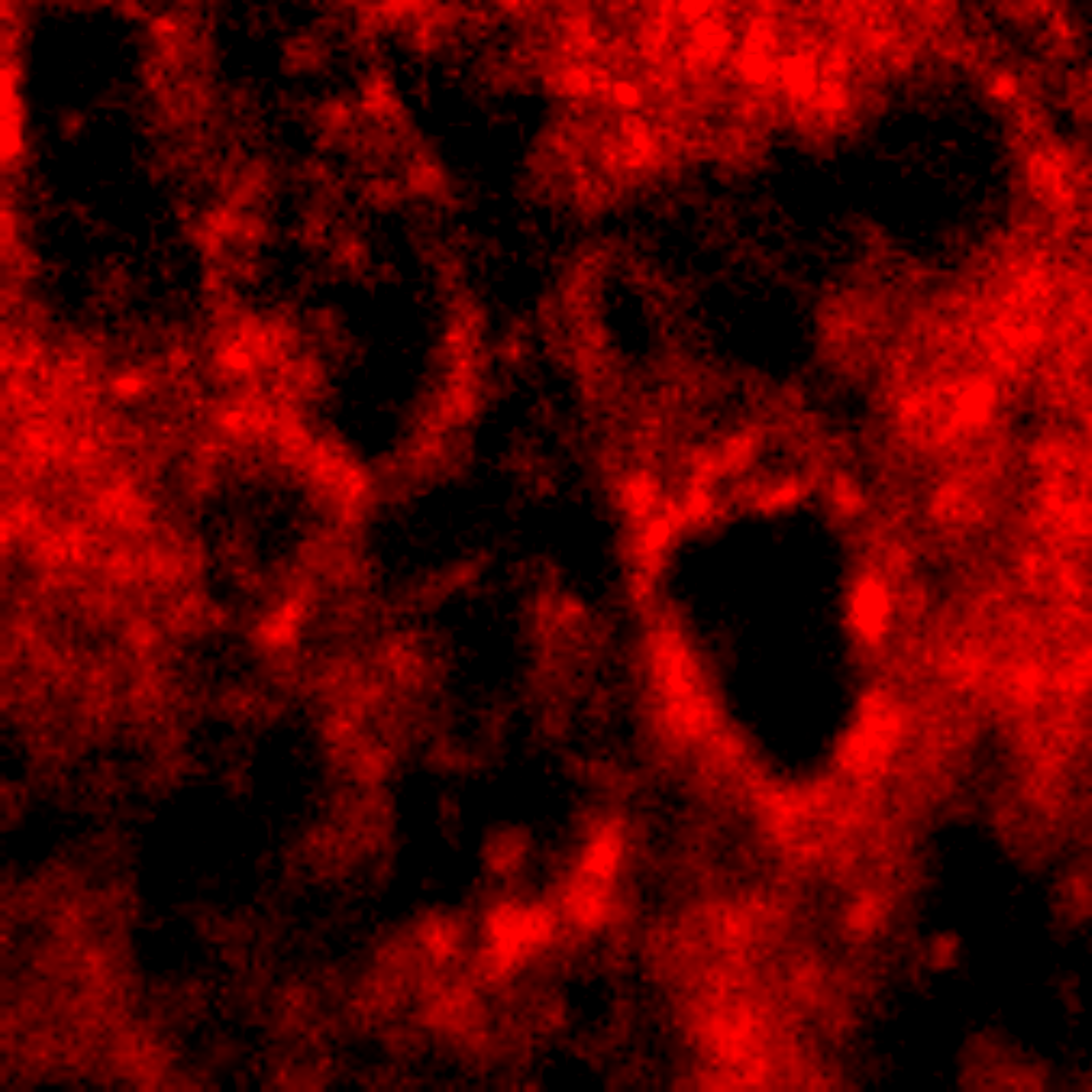

Supplement: Supplementary file 30 — Source Data For Expanded View [file 44318_2025_609_MOESM30_ESM.zip › SourceDataForExpandedView/FigureEV2/EV2B/ANXA7-TIA1-DAPI/motor cortex ANXA7.tif]

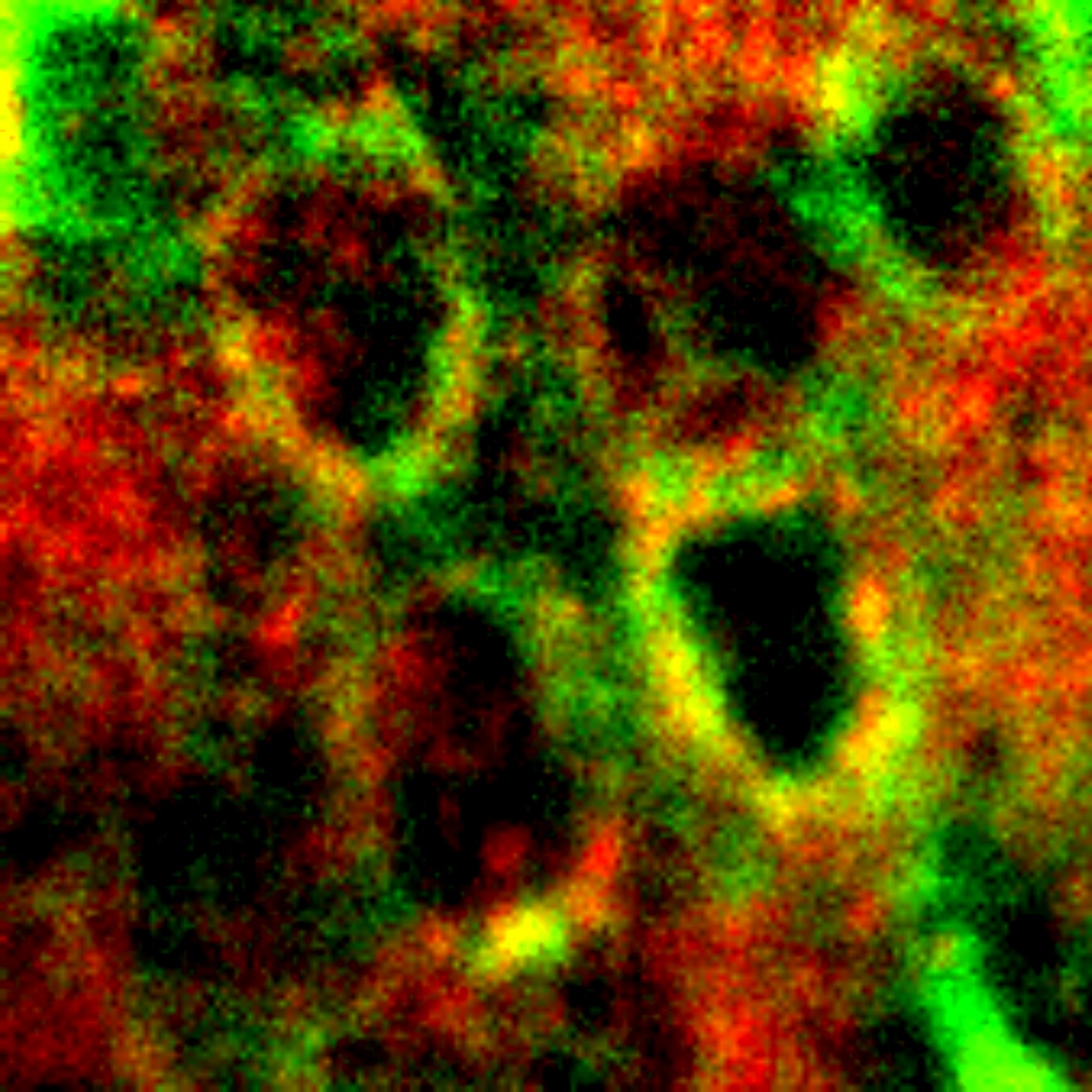

Supplement: Supplementary file 30 — Source Data For Expanded View [file 44318_2025_609_MOESM30_ESM.zip › SourceDataForExpandedView/FigureEV2/EV2B/ANXA7-TIA1-DAPI/motor cortex Merge.tif]

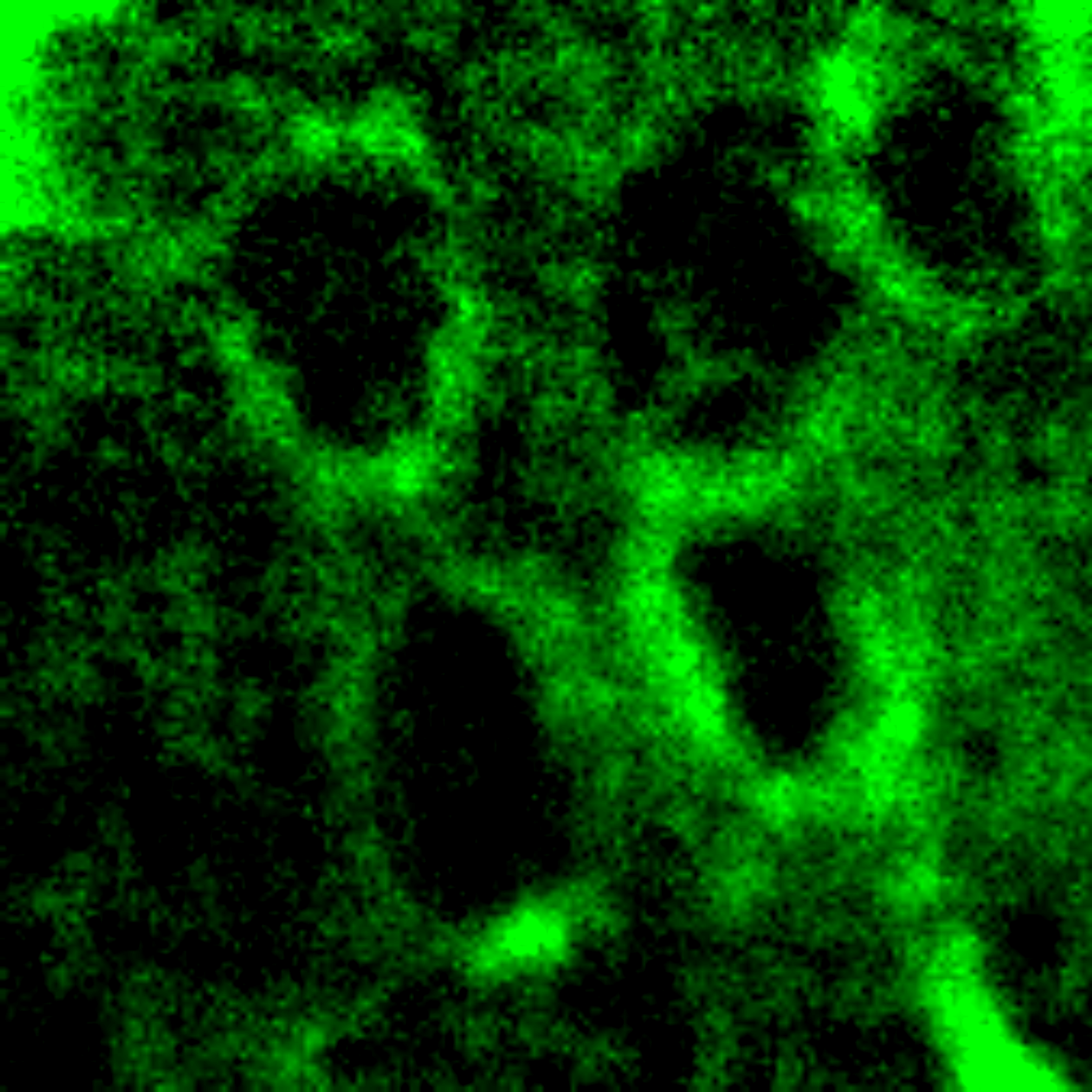

Supplement: Supplementary file 30 — Source Data For Expanded View [file 44318_2025_609_MOESM30_ESM.zip › SourceDataForExpandedView/FigureEV2/EV2B/ANXA7-TIA1-DAPI/motor cortex TIA1.tif]

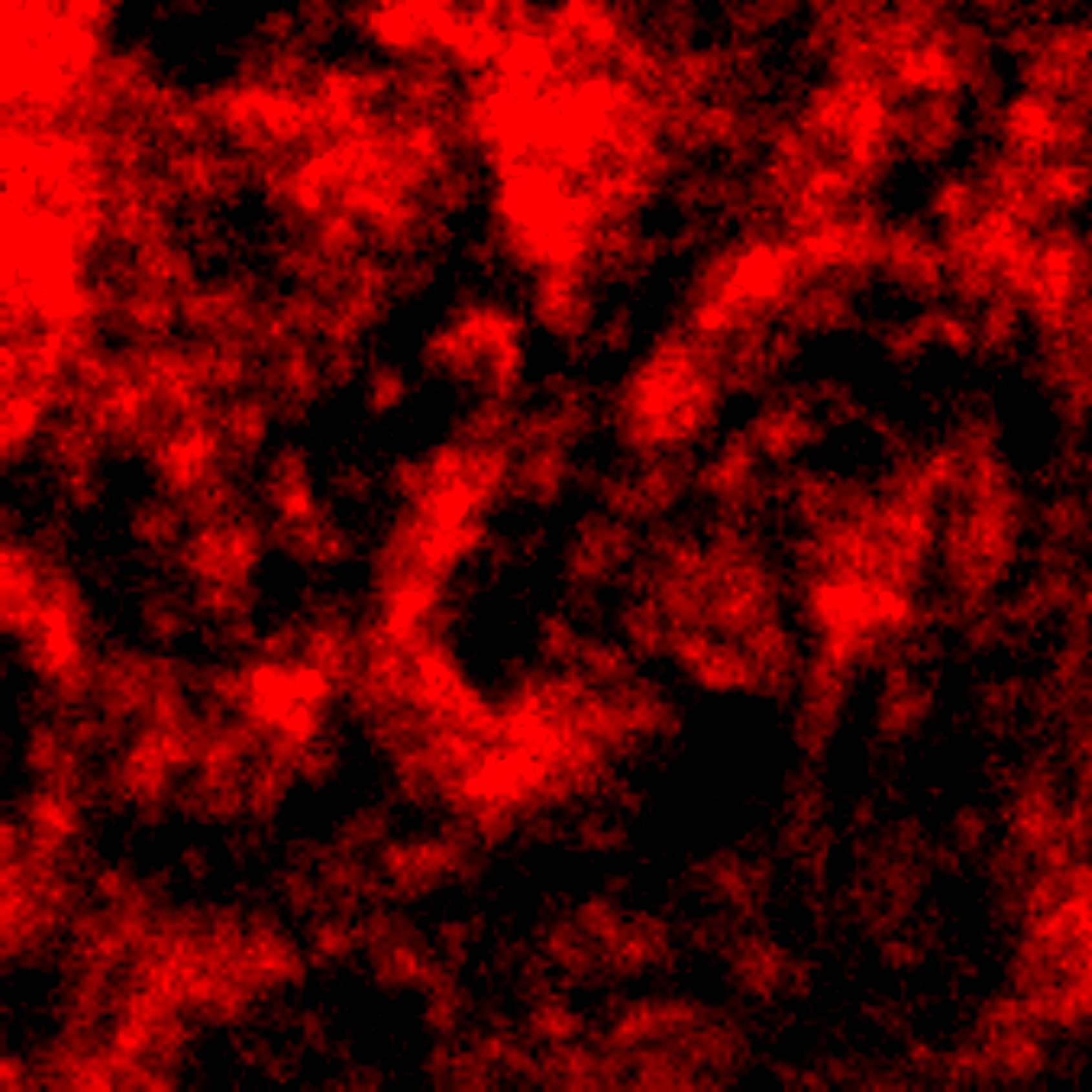

Supplement: Supplementary file 30 — Source Data For Expanded View [file 44318_2025_609_MOESM30_ESM.zip › SourceDataForExpandedView/FigureEV2/EV2B/DIC1B-TIA1-DAPI/CA3 DIC1B.tif]

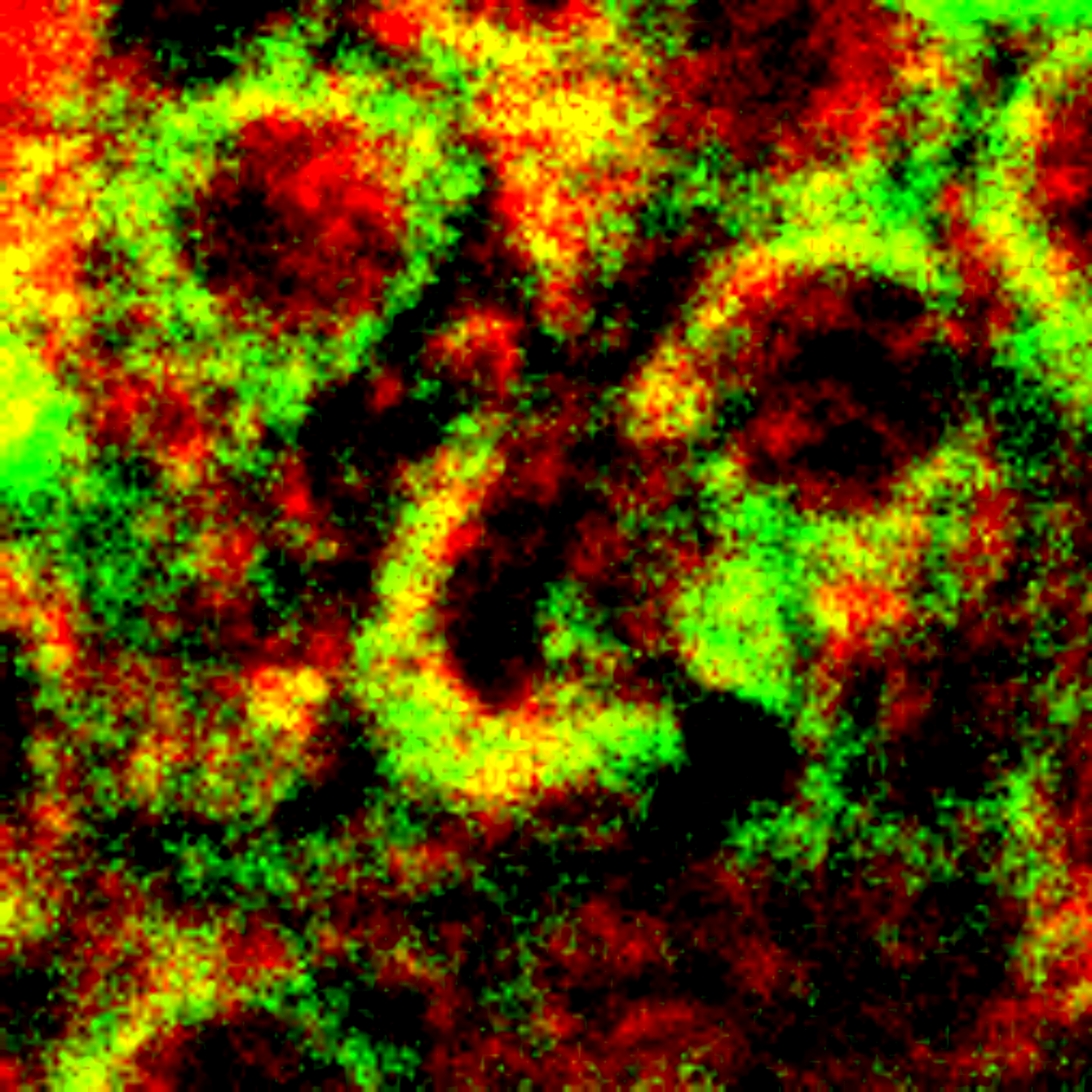

Supplement: Supplementary file 30 — Source Data For Expanded View [file 44318_2025_609_MOESM30_ESM.zip › SourceDataForExpandedView/FigureEV2/EV2B/DIC1B-TIA1-DAPI/CA3 Merge.tif]

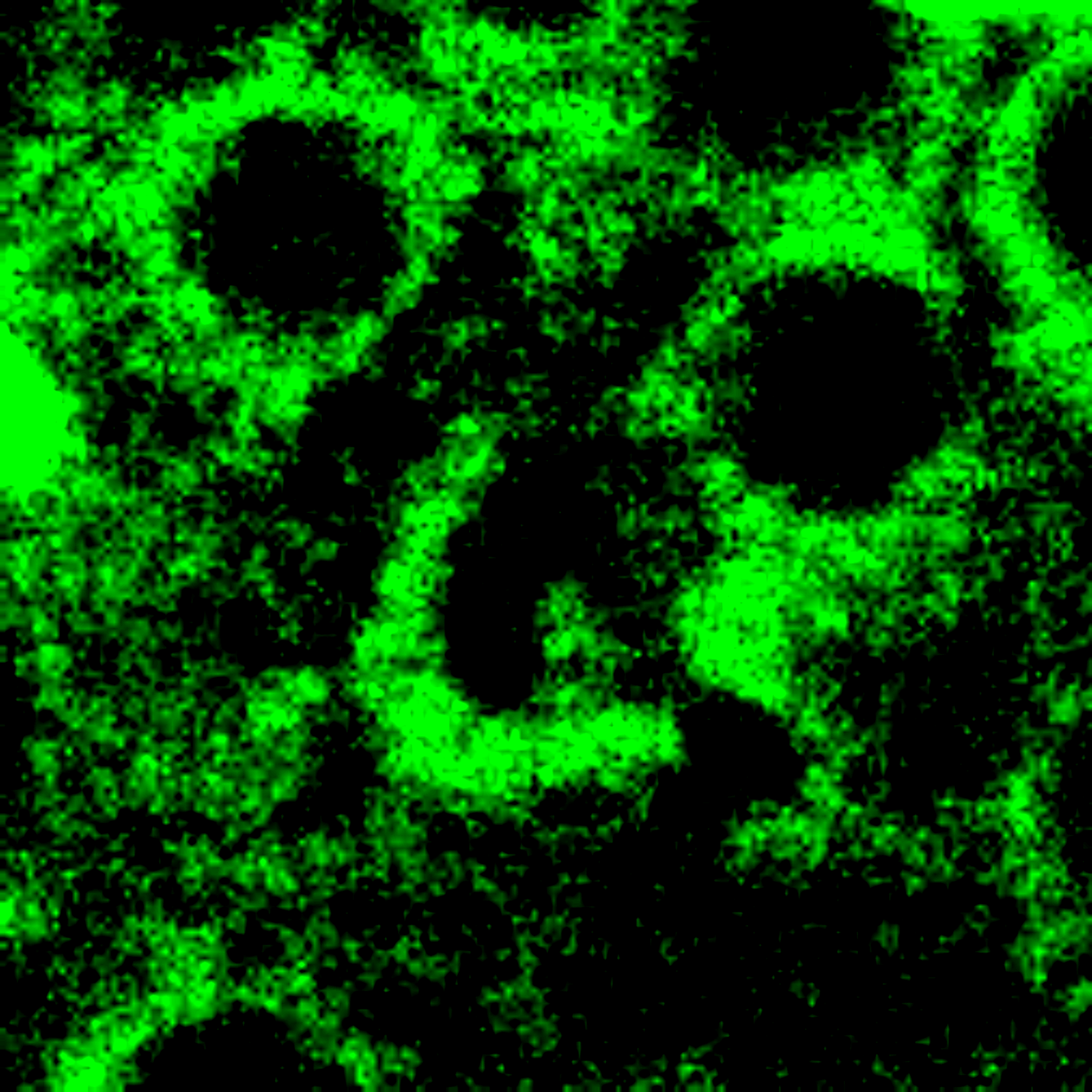

Supplement: Supplementary file 30 — Source Data For Expanded View [file 44318_2025_609_MOESM30_ESM.zip › SourceDataForExpandedView/FigureEV2/EV2B/DIC1B-TIA1-DAPI/CA3 TIA1.tif]

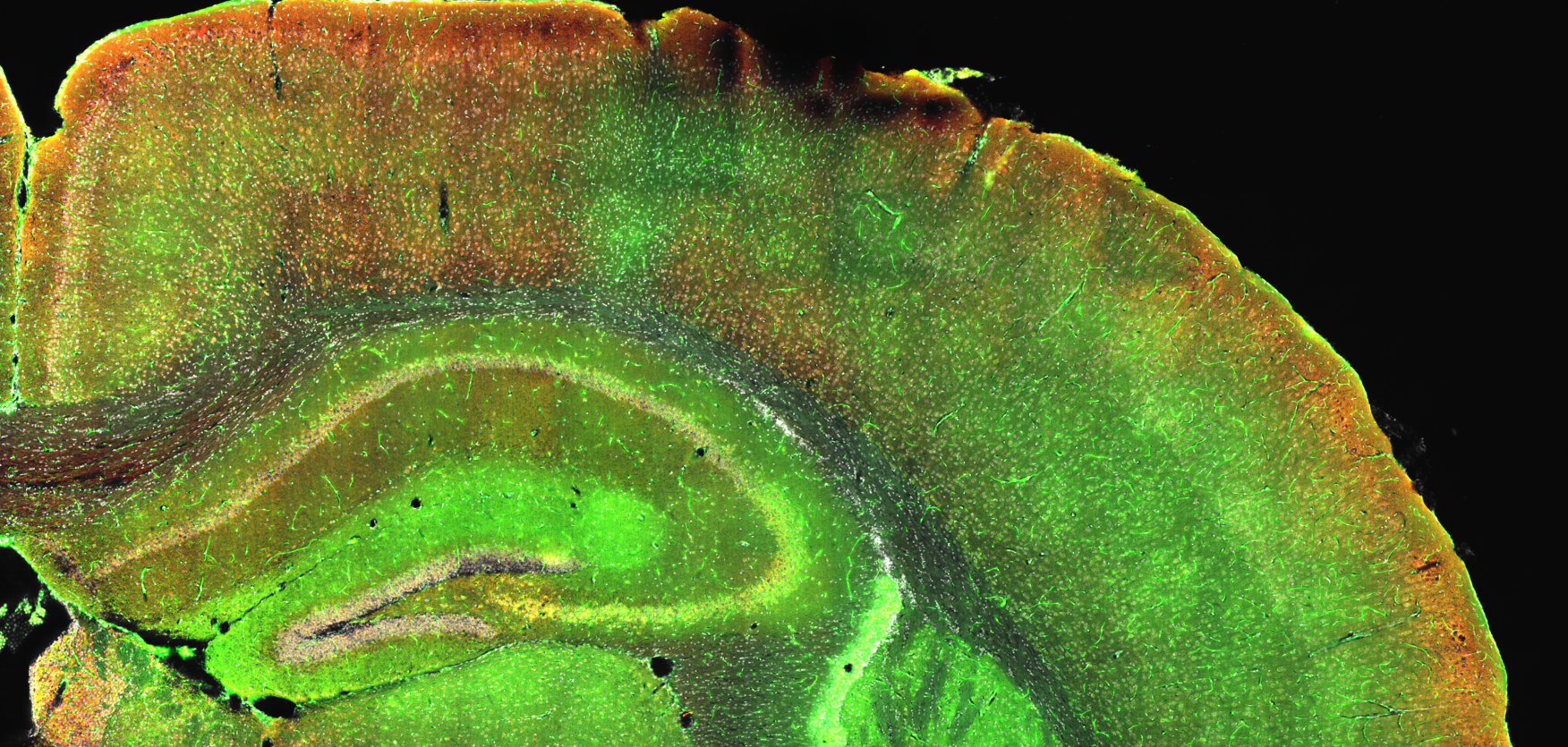

Supplement: Supplementary file 30 — Source Data For Expanded View [file 44318_2025_609_MOESM30_ESM.zip › SourceDataForExpandedView/FigureEV2/EV2B/DIC1B-TIA1-DAPI/DIC1B-TIA1-DAPI_large image.tif]

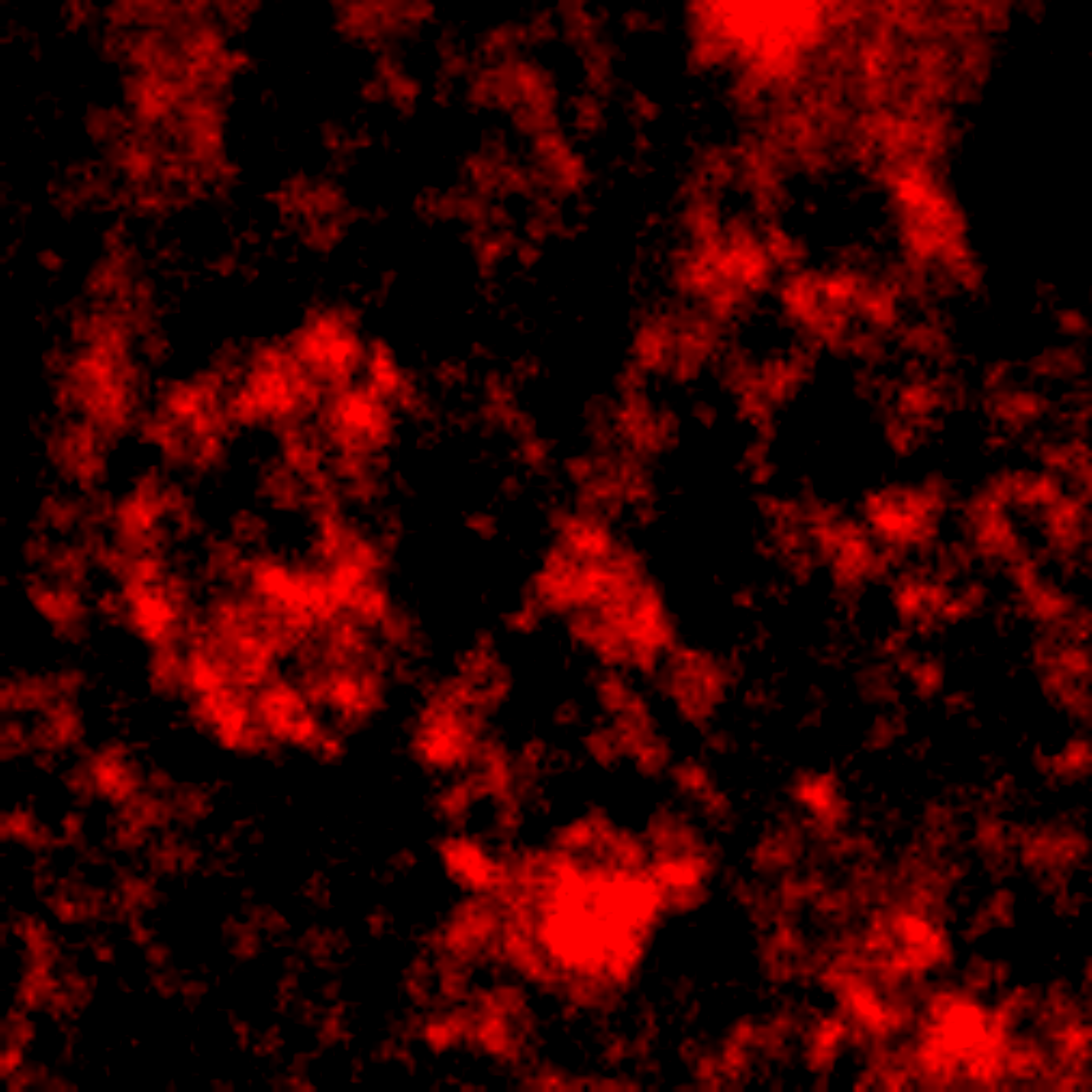

Supplement: Supplementary file 30 — Source Data For Expanded View [file 44318_2025_609_MOESM30_ESM.zip › SourceDataForExpandedView/FigureEV2/EV2B/DIC1B-TIA1-DAPI/motor cortex DIC1B.tif]

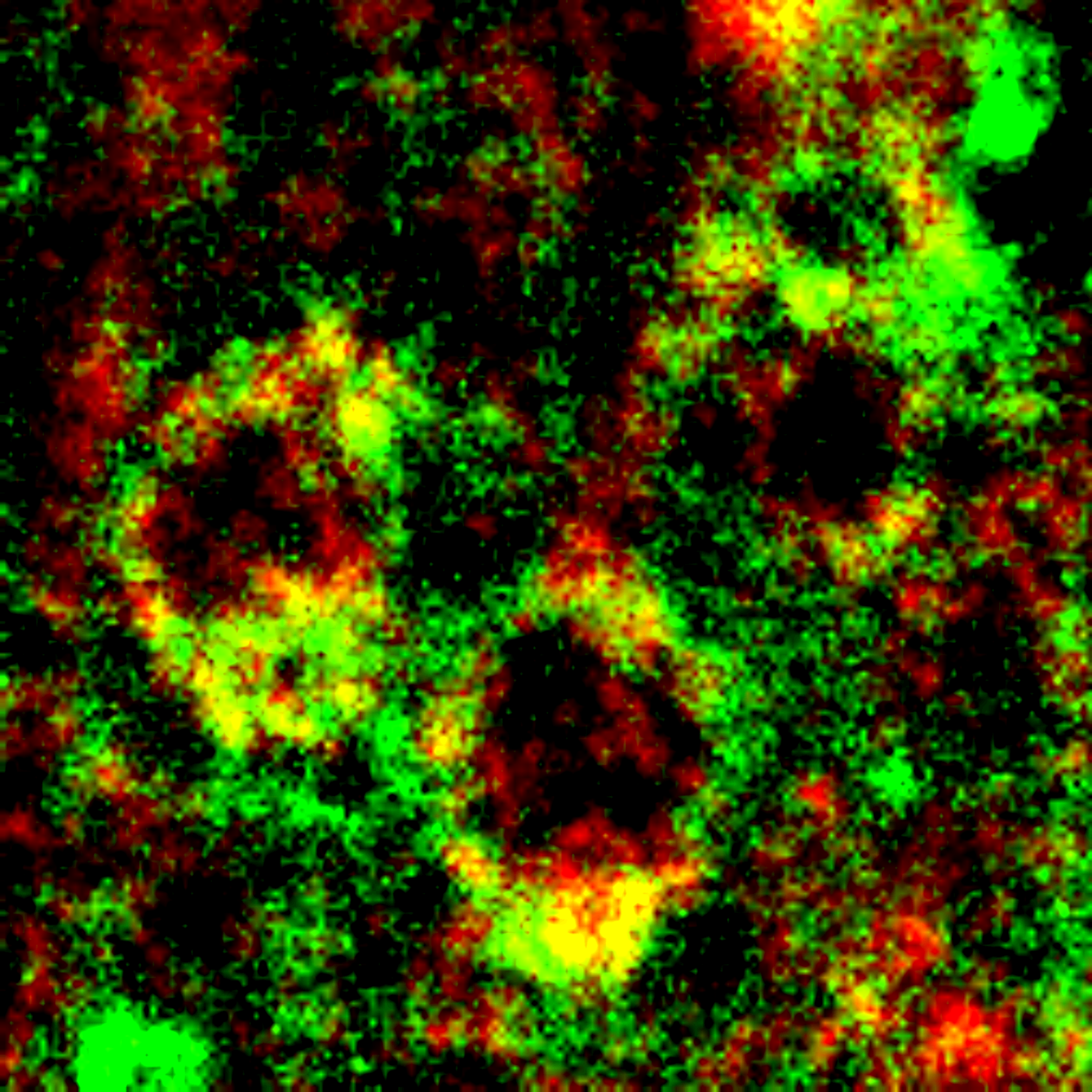

Supplement: Supplementary file 30 — Source Data For Expanded View [file 44318_2025_609_MOESM30_ESM.zip › SourceDataForExpandedView/FigureEV2/EV2B/DIC1B-TIA1-DAPI/motor cortex Merge.tif]

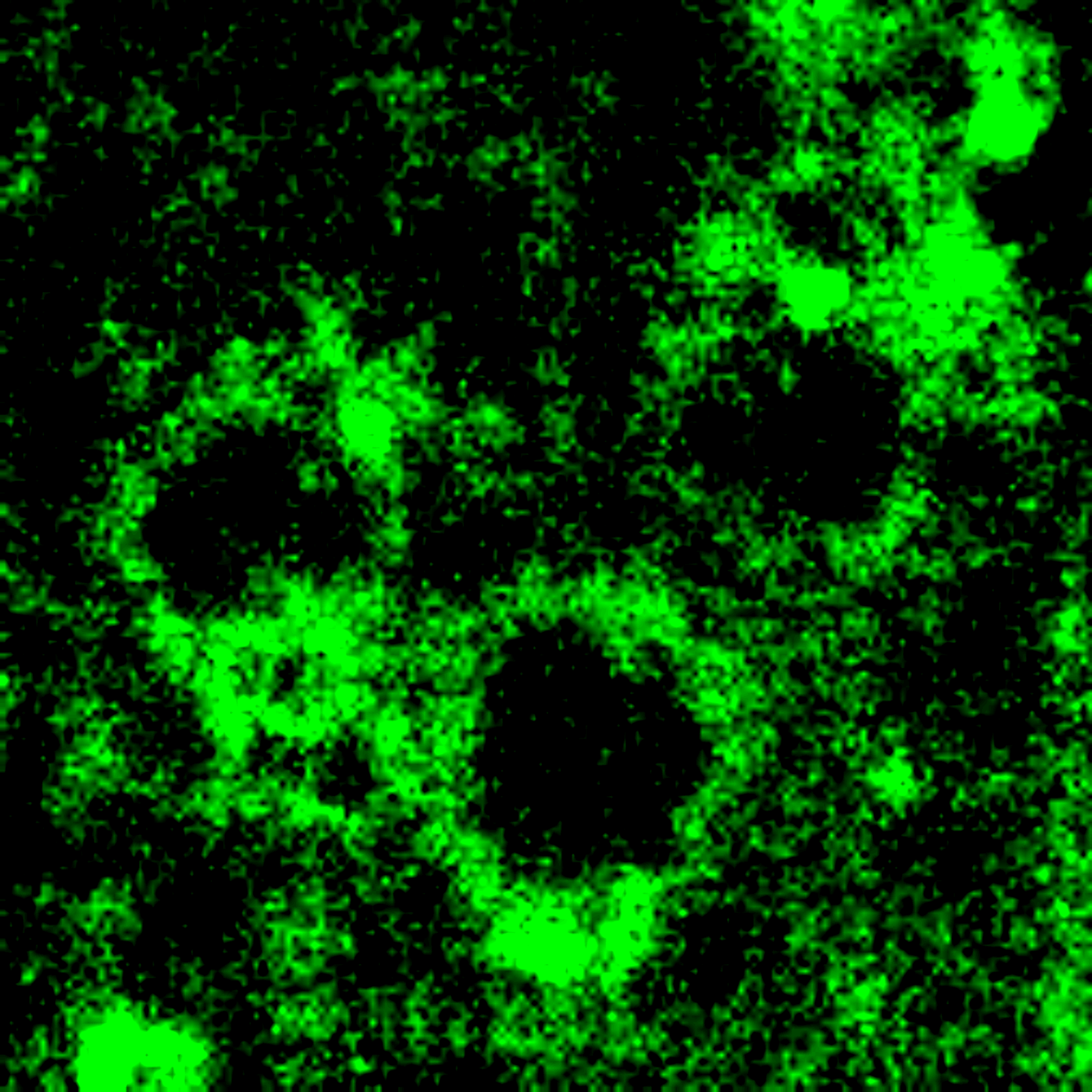

Supplement: Supplementary file 30 — Source Data For Expanded View [file 44318_2025_609_MOESM30_ESM.zip › SourceDataForExpandedView/FigureEV2/EV2B/DIC1B-TIA1-DAPI/motor cortex TIA1.tif]

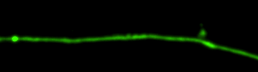

Supplement: Supplementary file 30 — Source Data For Expanded View [file 44318_2025_609_MOESM30_ESM.zip › SourceDataForExpandedView/FigureEV4/EV4G/Control-EGFP.tif]

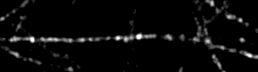

Supplement: Supplementary file 30 — Source Data For Expanded View [file 44318_2025_609_MOESM30_ESM.zip › SourceDataForExpandedView/FigureEV4/EV4G/Control-TIA1.tif]

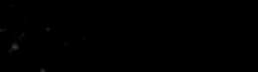

Supplement: Supplementary file 30 — Source Data For Expanded View [file 44318_2025_609_MOESM30_ESM.zip › SourceDataForExpandedView/FigureEV4/EV4G/Control-TDP43.tif]
