# Supplementary material for: Annexin A7 enhances TIA1 axonal trafficking to counteract pathological aggregation in neurons
Source: EMBO J. 2025 Nov 3;44(24):7477–512. doi: 10.1038/s44318-025-00609-8 (PMC12706091; doi:10.1038/s44318-025-00609-8)
Supplement: Supplementary file 31 — Expanded View Figures [file 44318_2025_609_MOESM31_ESM.pdf]

Expanded View Figures

Figure EV1. TIA1 granules undergo retrograde trafficking in axons.

(**A**) Cy5-UTP co-localization with indicated RNP markers. Arrowheads indicate RNPs with these markers. Scale ba = 5 µm. (**B**) Schematic diagram of the pulse-chase labeling assay to specifically label retrograde membranous axonal organelles in neurons cultured in a microfluidic device. See also the Methods for details. Scale bar = 50 µm. (**C**, **D**) Key frames from time-lapse images showing TIA1 granules trafficking with axon-derived CTB (**C**) or BoNT/A-Hc (**D**) in a microfluidic device. Arrowheads indicate moving TIA1 granules. Scale bar = 10 µm. (**E**, **F**) Key frames from time-lapse images showing TIA1 granules trafficking with whole cell stained MitoTracker (**E**) or co-expressed EGFP-Rab5 (**F**). Arrowheads indicate moving TIA1 granules and organelles. Scale bar = 5 µm. (**G**) Representative confocal images of endogenous TIA1 with organelle markers (Rab5 for endosomes, LC3 for autophagosomes, LAMP1 for lysosomes, DIC1B for dynein) in axons, with intensity profiles shown below. Scale bars = 10 µm (top), 5 µm (bottom). (**G′-G‴**) Quantification of (**G**), with (**G′**) showing the Pearson's coefficient of endogenous TIA1 granules with indicated markers, and (**G″**) showing the ratio of TIA1 co-localized with the indicated markers, and (**G‴**) showing the ratio of the markers co-localized with TIA1 ($n = 56, 56, 53, 56$ axons from three biological replicates). (**H**) Screening of shDIC1B constructs for knockdown efficiency in cultured rat cortical neurons. shRNA sequences of 1# and 2# are available in Table EV1. (**I–I′**) Key frames from time-lapse images showing axon trafficking of TIA1-mCherry granules with or without 10 µM, 15 min nocodazole treatment, with arrowheads in different colors marking distinct granules (**I**), and corresponding quantification (**I′**) ($n = 334$ and 497 granules from three biological replicates). Scale bar = 10 µm. Data represent mean ± SEM; two-tailed unpaired *t*-test in (**I′**); one-way ANOVA in (**G′**, **G″**, **G‴**); $^{**}P < 0.01$, $^{***}P < 0.001$. See appendix for exact *P* values. Source data are available online for this figure.

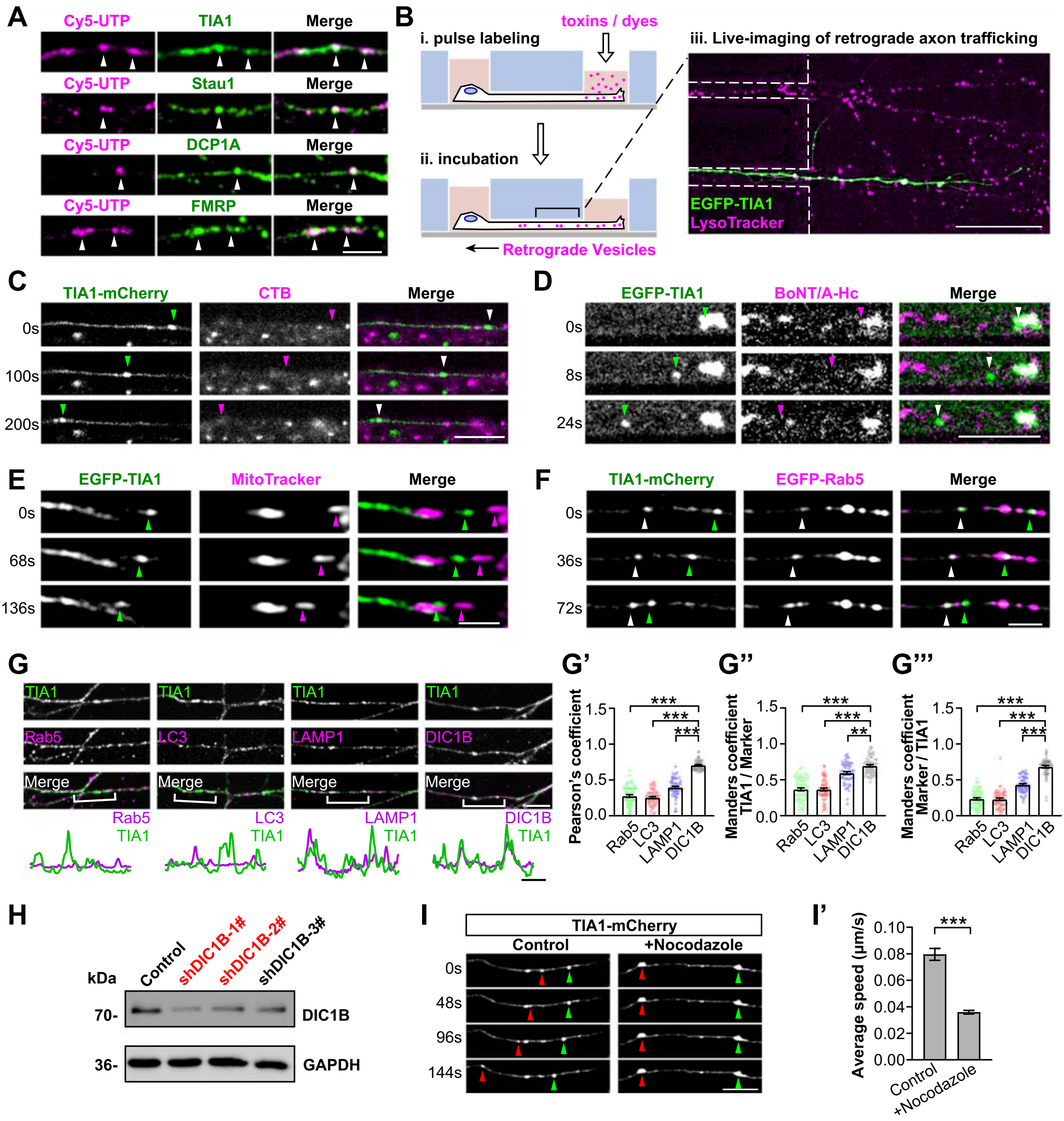
A
Cy5-UTP TIA1 Merge
Cy5-UTP Stau1 Merge
Cy5-UTP DCP1A Merge
Cy5-UTP FMRP Merge
B
i. pulse labeling
toxins / dyes
ii. incubation
Retrograde Vesicles
iii. Live-imaging of retrograde axon trafficking
EGFP-TIA1
LysoTracker
C
TIA1-mCherry CTB Merge
0s
100s
200s
D
EGFP-TIA1 BoNT/A-Hc Merge
0s
8s
24s
E
EGFP-TIA1 MitoTracker Merge
0s
68s
136s
F
TIA1-mCherry EGFP-Rab5 Merge
0s
36s
72s
G
TIA1
Rab5
LC3
LAMP1
DIC1B
Merge
G'
Pearson's coefficient
G''
Manders coefficient TIA1 / Marker
G'''
Manders coefficient Marker / TIA1
Rab5 LC3 LAMP1 DIC1B
H
kDa
Control
shDIC1B-1#
shDIC1B-2#
shDIC1B-3#
70-
DIC1B
36-
GAPDH
I
TIA1-mCherry
Control
+Nocodazole
0s
48s
96s
144s
I'
Average speed (μm/s)
Control
+Nocodazole

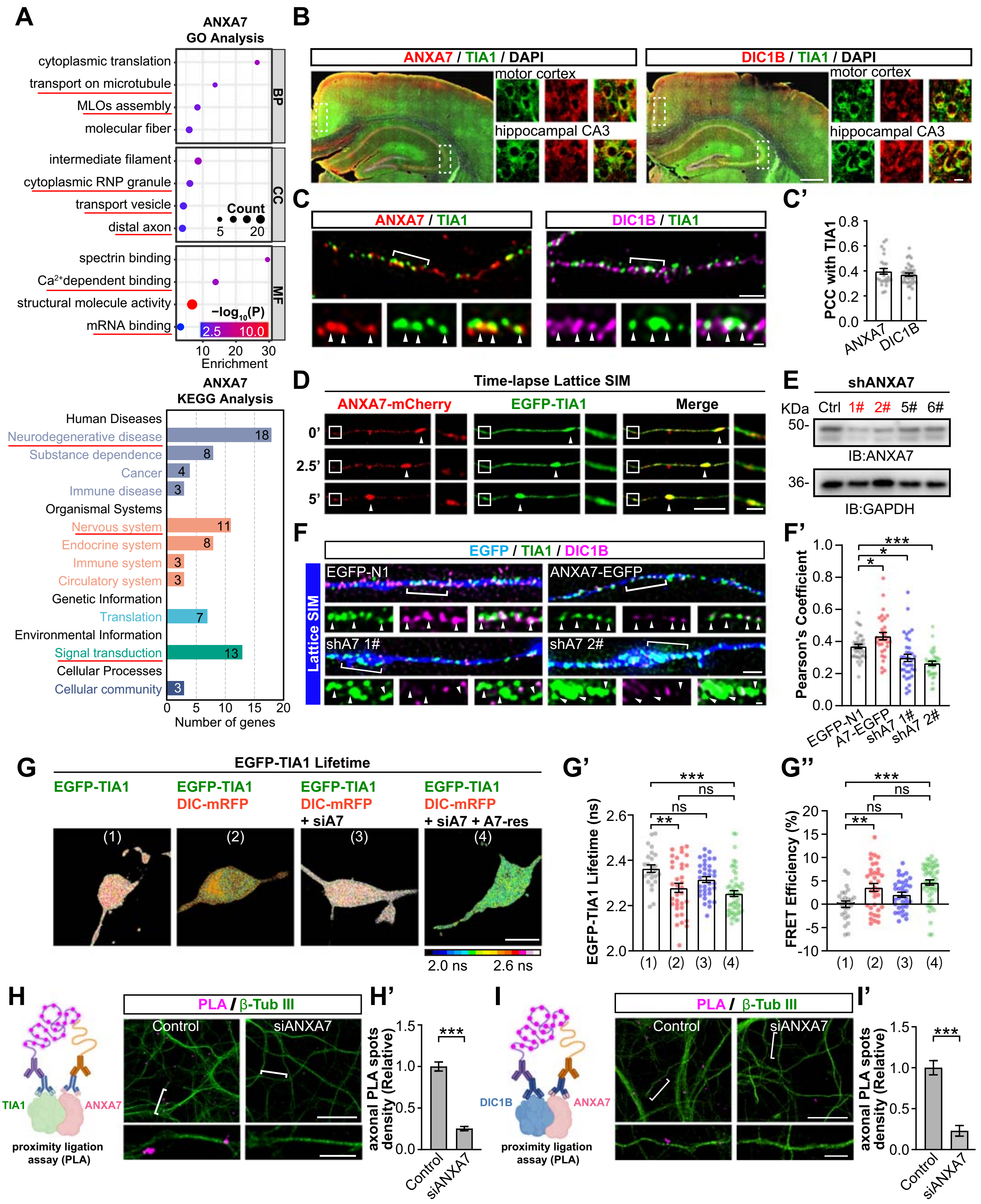
A
ANXA7 GO Analysis
cytoplasmic translation
transport on microtubule
MLOs assembly
molecular fiber
BP
intermediate filament
cytoplasmic RNP granule
transport vesicle
distal axon
CC
Count
5 20
spectrin binding
Ca2+dependent binding
structural molecule activity
mRNA binding
MF
−log10(P)
2.5 10.0
10 20 30
Enrichment
ANXA7 KEGG Analysis
Human Diseases
Neurodegenerative disease 18
Substance dependence 8
Cancer 4
Immune disease 3
Organismal Systems
Nervous system 11
Endocrine system 8
Immune system 3
Circulatory system 3
Genetic Information
Translation 7
Environmental Information
Signal transduction 13
Cellular Processes
Cellular community 3
0 5 10 15 20
Number of genes
B
ANXA7 / TIA1 / DAPI
motor cortex
hippocampal CA3
DIC1B / TIA1 / DAPI
C
ANXA7 / TIA1
DIC1B / TIA1
C'
PCC with TIA1
ANXA7
DIC1B
D
Time-lapse Lattice SIM
ANXA7-mCherry
EGFP-TIA1
Merge
0'
2.5'
5'
E
shANXA7
KDa Ctrl 1# 2# 5# 6#
50-
IB:ANXA7
36-
IB:GAPDH
F
EGFP / TIA1 / DIC1B
Lattice SIM
EGFP-N1
ANXA7-EGFP
shA7 1#
shA7 2#
F'
Pearson's Coefficient
EGFP-N1
A7-EGFP
shA7 1#
shA7 2#
G
EGFP-TIA1 Lifetime
EGFP-TIA1
EGFP-TIA1 DIC-mRFP
EGFP-TIA1 DIC-mRFP + siA7
EGFP-TIA1 DIC-mRFP + siA7 + A7-res
(1) (2) (3) (4)
2.0 ns 2.6 ns
G'
EGFP-TIA1 Lifetime (ns)
(1) (2) (3) (4)
G''
FRET Efficiency (%)
(1) (2) (3) (4)
H
TIA1
ANXA7
proximity ligation assay (PLA)
PLA / β-Tub III
Control
siANXA7
H'
axonal PLA spots density (Relative)
Control
siANXA7
I
DIC1B
ANXA7
proximity ligation assay (PLA)
PLA / β-Tub III
Control
siANXA7
I'
axonal PLA spots density (Relative)
Control
siANXA7

Figure EV2. ANXA7 promotes the recruitment of TIA1 granules to dynein.

(**A**) GO and KEGG pathway analysis of GST-ANXA7 interactors, including biological processes (BP), cellular components (CC), and molecular functions (MF). *P* values were computed using the hypergeometric test, and *q* values (adjusted *P* values) were derived using the Benjamini–Hochberg FDR method. Only categories with $q < 0.05$ are shown, ranked by descending enrichment score ($-\log_{10}$(P)). (**B**) Confocal images of endogenous TIA1 (green) and ANXA7 or DIC1B (red) in the cortex and hippocampus of P34 mouse brain. Scale bars = 500 and 10 µm. (**C**) In DIV12 rat hippocampal neurons, 3D-Lattice SIM images of TIA1 (green) and ANXA7 (red) or DIC1B (magenta) along axons. Arrowheads indicate co-localization. Scale bars = 1 µm and 200 nm. (**C′**) Pearson's coefficient for co-localization between TIA1 and ANXA7 or DIC1B from (**C**) ($n = 26$, 38 axons from three biological replicates). (**D**) Arrows in key frames from time-lapse SIM images showing co-trafficking of granules in DIV13 hippocampal neurons. One newly formed TIA1/ANXA7 granule (boxed) are amplified in the right panels. Scale bars = 5 and 1 µm. (**E**) Western blot showing shRNA-mediated knockdown of endogenous ANXA7 in rat cortical neurons. shRNA sequences (#1 and #2) are listed in Table EV1. (**F**) 3D-lattice-SIM images showing the distribution of endogenous TIA1 (green) and DIC1B (magenta) in axons of DIV12 cultured rat hippocampal neurons under conditions of endogenous ANXA7 knockdown (shA7 1# and 2#) or ANXA7-EGFP overexpression. Arrowheads indicate co-localized TIA1 and DIC1B spots. Scale bars = 1 µm (top) and 200 nm (bottom). (**F′**) Pearson's coefficient quantifying co-localization between TIA1 and DIC1B ($n = 38$, 32, 37, 35 axons from three biological replicates). (**G**) Color-coded EGFP-TIA1 lifetime in the soma of transfected neurons, with lifetime (**G′**) and FRET efficiency (**G″**) quantified and compared across the indicated groups ($n = 25$, 35, 35, 53 neurons from 4 biological replicates). Scale bar = 10 µm. (**H–I′**) Left: schematics illustrating PLA detection of endogenous TIA1/ANXA7 (**H**) and DIC1B/ANXA7 (**I**) interactions. Right: Confocal images showing PLA signals ANXA7 knockdown (siANXA7) neurons, with bracketed axons enlarged below. Scale bars = 50 µm (top), 10 µm (bottom). Quantification of axonal PLA density shown in (**H′**) and (**I′**) ($n = 100$, 72 ROIs for (**H′**) from four biological replicates; $n = 60$ ROIs for (**I′**) from three biological replicates). Data represent mean ± SEM; one-sample *t*-test in (**H′**, **I′**), one-way ANOVA in (**F′**, **G′**, **G″**); $^{*}P < 0.05$, $^{**}P < 0.01$, $^{***}P < 0.001$, ns non-significant. See appendix for exact *P* values. Source data are available online for this figure.

 © The Author(s)

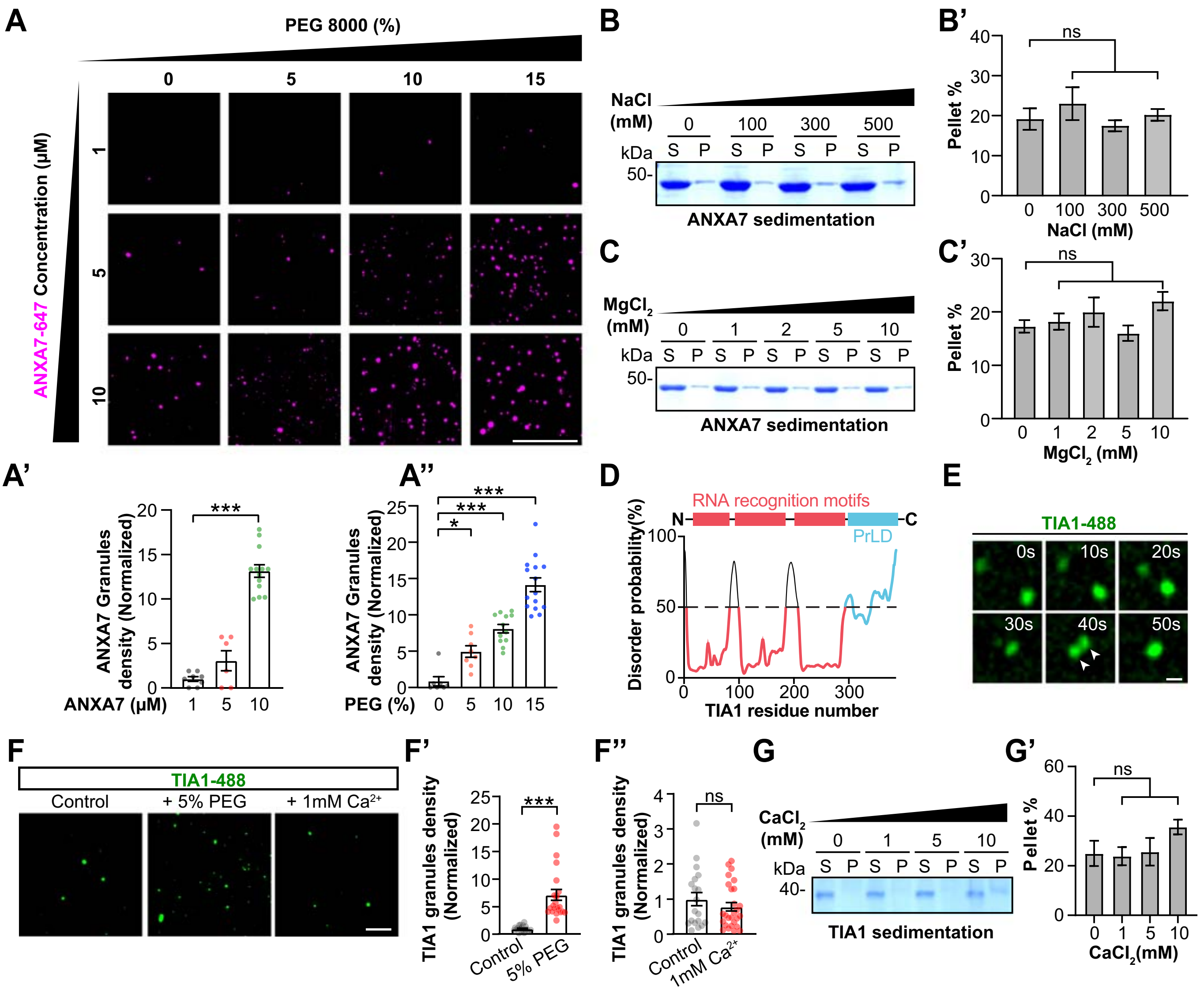

Figure EV3. The TIA1 phase separation is not affected by Ca^{2+} elevation.

(**A**) In vitro assay demonstrating the phase separation of purified ANXA7-647 induced by PEG-8000 addition. The concentrations of PEG and ANXA7-647 are indicated. Scale bar = 50 μm. (**A′-A″**) Quantification of (**A**), showing the ANXA7 granules density under different concentrations of protein (**A′**) and PEG (**A″**) (for (**A′**): PEG concentration = 0%, *n* = 8, 6, 13 ROIs; for (**A″**): ANXA7 concentration = 5 μM, *n* = 7, 8, 12, 15 ROIs from three biological replicates). (**B–C′**) In vitro sedimentation assays detected by SDS-PAGE showing the distribution of purified ANXA7 (5 μM) between supernatant (S) and pellet (P) fractions at the indicated concentrations of NaCl (**B**) or $MgCl_2$ (**C**). Quantification shown in (**B′**) and (**C′**) (for (**B′**): *n* = 5 technical replicates from three biological replicates; for (**C′**): *n* = 4 biological replicates). (**D**) Schematic diagram of the TIA1 protein domain structure with PrDOS analysis, showing the C-terminal PrLD. (**E**) Key frames from time-lapse images showing the in vitro LLPS process of purified TIA1 protein (TIA1-488), with fusion events of droplets indicated by arrowheads. Scale bar = 2 μm. (**F**) In vitro LLPS assay using purified TIA1 protein (TIA1-488), illustrating the effects of adding 5% PEG or 1 mM Ca^{2+} on phase separation. Scale bar = 10 μm. (**F′-F″**) Quantification of (**F**), with the effect of 5% PEG (**F′**) or 1 mM Ca^{2+} (**F″**) on TIA1 granules density compared to those of control groups, respectively (for (**F′**): *n* = 20, 23 ROIs; for (**F″**): *n* = 19, 27 ROIs from three biological replicates). (**G**) In vitro sedimentation assay detected by SDS-PAGE showing the distribution of purified TIA1 protein (5 μM) in the supernatant (S) and pellet (P) fractions at the indicated concentration of $CaCl_2$. (**G′**) Quantification of the sedimentation assay results in (**G**) (*n* = 3 biological replicates). Data represent mean ± SEM; two-tailed unpaired *t*-test in (**F′, F″**); one-way ANOVA in (**A′, A″, B′, C′, G′**); $^*P < 0.05$, $^{***}P < 0.001$, ns non-significant. See appendix for exact *P* values. Source data are available online for this figure.

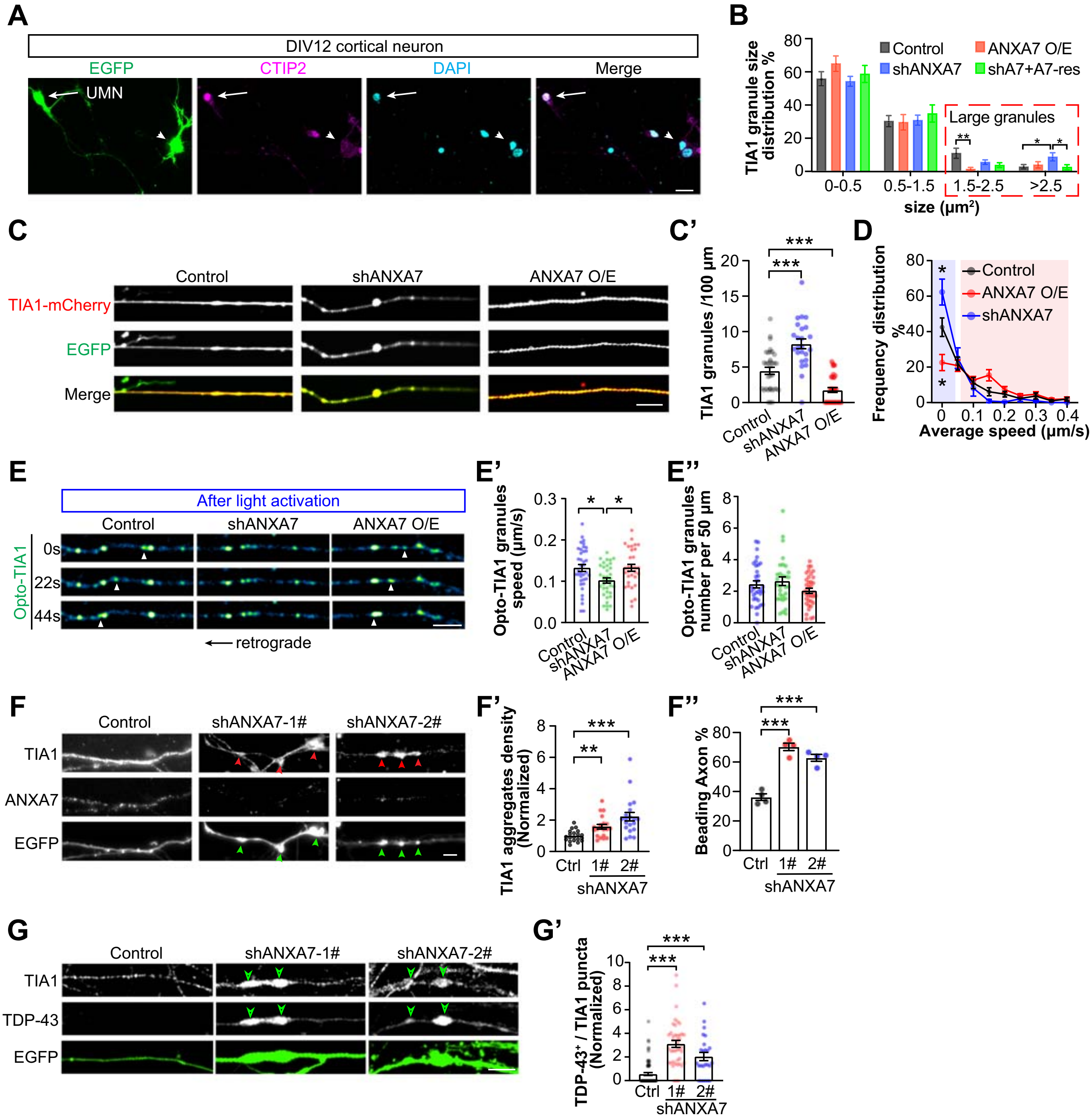
A
DIV12 cortical neuron
EGFP
CTIP2
DAPI
Merge
UMN
B
TIA1 granule size distribution %
Control
ANXA7 O/E
shANXA7
shA7+A7-res
Large granules
0-0.5
0.5-1.5
1.5-2.5
>2.5
size (μm²)
C
Control
shANXA7
ANXA7 O/E
TIA1-mCherry
EGFP
Merge
C'
TIA1 granules /100 μm
D
Frequency distribution %
Average speed (μm/s)
E
After light activation
Opto-TIA1
0s
22s
44s
retrograde
E'
Opto-TIA1 granules speed (μm/s)
E''
Opto-TIA1 granules number per 50 μm
F
shANXA7-1#
shANXA7-2#
TIA1
ANXA7
F'
TIA1 aggregates density (Normalized)
Ctrl
1#
2#
F''
Beading Axon %
G
TDP-43
G'
TDP-43⁺ / TIA1 puncta (Normalized)

 © The Author(s)

Figure EV4. ANXA7 regulates TIA1 axon trafficking and LLPS.

(**A**) Identification of upper motor neurons (UMNs) in DIV12 cortical cultures. CTIP2-positive UMNs are indicated by arrows; non-UMNs by arrowheads. Scale bar = 20 µm. (**B**) Related to Fig. 6A,A′. Quantification of TIA1 granule size from (Fig. 6A, **HN**). The red dotted box indicates large granules (≥2 µm^2, circularity 0.6–1) (*n* = 43, 43, 46, 40 axons from three biological replicates). (**C**) Distribution of TIA1-mCherry in axons of DIV11 rat hippocampal neurons with endogenous ANXA7 knockdown (shANXA7) or ANXA7-EGFP overexpression (ANXA7 O/E). Scale bar = 10 µm. (**C′**) Quantification of TIA1 aggregates per 100 µm axon under conditions in (**C**) (*n* = 34, 24, 35 axons from four biological replicates). (**D**) Distribution of the EGFP-TIA1 granule speeds from (Fig. 6C). Blue shadow indicates stationary granules (≤0.05 µm/s), red shading indicates mobile (>0.05 µm/s) granules (*n* = 21, 23, 23 axons from three biological replicates). (**E**) Key frames from time-lapse images of Opto-TIA1 in DIV9 rat hippocampal neurons with endogenous ANXA7 knocked down (shANXA7) or ANXA7-EGFP overexpression (ANXA7 O/E), showing retrograde trafficking of light-induced Opto-TIA1 granules after 11–20 min blue light exposure. Arrowheads indicate mobile Opto-TIA1 granules, and arrows indicate the retrograde direction. Scale bar = 10 µm. (**E′-E″**) Quantification from (**E**), showing the speed (**E′**) and density (**E″**) of Opto-TIA1 granules in the axons of indicated groups (**E′**: *n* = 37, 36, 31 granules; **E″**: *n* = 34, 30, 36 axons; all from three biological replicates). (**F**) IF staining images of TIA1 and ANXA7 in axons of DIV9 rat HN with ANXA7 knockdown using two shRNA sequences (shANXA7-1# and 2#). EGFP shows axon morphology, with arrowheads indicating beading structures. Scale bar = 5 µm. (**F′-F″**) Quantification of (**F**), showing the density of TIA1 aggregates (**F′**) and the percentage of beading axons (**F″**) (**F′**: *n* = 20 axons from four biological replicates; **F″**: *n* = 4 biological replicates). (**G**) Distribution of endogenous TIA1 with TDP-43 in axons of HN. EGFP depicts axon morphology. Scale bar = 10 µm. (**G′**) Quantification from (**G**) showing the number of TIA1 puncta co-localized with TDP-43 per 100 µm axon (*n* = 70, 42, 25 axons from four biological replicates). Data represent mean ± SEM; one-sample *t*-test in (**F′**); two-tailed unpaired *t*-test in (**B**); one-way ANOVA in (**C′**, **D**, **E′**, **E″**, **F″**, **G′**); *$P < 0.05$, **$P < 0.01$, ***$P < 0.001$. See appendix for exact *P* values. Source data are available online for this figure.

© The Author(s)

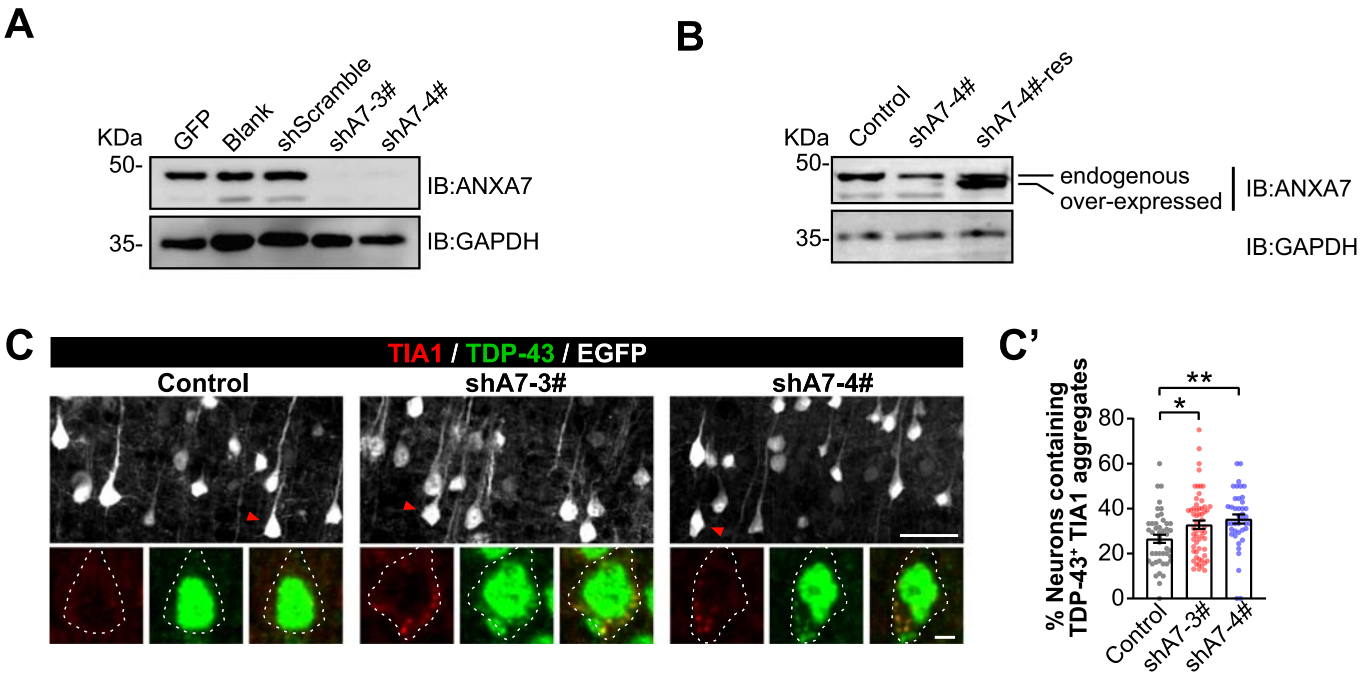

Figure EV5. ANXA7 knockdown leads to TIA1 aggregation in layer V neurons of the mouse motor cortex.

(**A**) Western blot validating the knockdown efficiency of two different shRNA sequences targeting ANXA7 in mouse brains (shA7-3# and 4#). shRNA sequences are available in Table EV1. (**B**) Western blot validating the rescue efficiency of shA7-4#-res, which overexpressing rat ANXA7 resistant against shANXA7-4# (targeting the mouse intron sequence) knockdown of endogenous ANXA7 in cultured mouse cortical neurons. (**C**) Confocal images of P60 mouse cortex showing TIA1 and TDP-43 IF in layer V neurons. Infected neurons marked by EGFP expression, the neurons pointed by red arrows are amplified in lower panels. Dotted lines depict soma shapes. Scale bars = 50 μm (top), 5 μm (bottom). (**C'**) Percentage of neurons containing TDP-43^{+} TIA1 aggregates (n = 45, 58, 41 ROIs from three mices). Data represent mean ± SEM, one-way ANOVA in (**C'**); *$P < 0.05$, **$P < 0.01$. See appendix for exact P values. Source data are available online for this figure.

 © The Author(s)
